# Comprehensive Identification and Abscisic Acid-Responsive Expression Profiling of NAC Transcription Factor in Triterpenoid Saponin in *Hedera helix*

**DOI:** 10.3390/biom15111557

**Published:** 2025-11-06

**Authors:** Xiaoji Deng, Feixiong Zheng, Zhangting Xu, Xiaoping Mao, Zhenming Yu, Xiaoxia Shen

**Affiliations:** 1 School of Pharmaceutical Sciences, Academy of Chinese Medical Sciences, Zhejiang Chinese Medical University, Hangzhou 310053, China; 13868867263@163.com (X.D.); feixiongz@163.com (F.Z.); 15168104031@163.com (Z.X.); 2 Key Laboratory for Collaborative Innovation and Sustainable Utilization of Dao-di Herbs, Songyang Institute, Zhejiang Chinese Medical University, Lishui 323400, China; 3 Zhejiang Conba Chinese Medicine Co., Ltd., Lishui 323400, China; maoxp@conbapharm.com

**Keywords:** *Hedera helix*, triterpenoid saponins, transcriptome, transcription factors, ABA

## Abstract

Triterpenoid saponins are important secondary metabolites in plants. Abscisic acid (ABA), as one of the indispensable regulatory hormones in plants, promotes the accumulation of bioactive components in various plants, including triterpenoid saponins; however, its induced mechanism in *Hedera helix* remains unclear. In this study, the treatment of *H. helix* leaves with 100 μM ABA led to the identification of 7108 differentially expressed genes (DEGs) within 6 h post-treatment through transcriptomic and bioinformatic analysis. Enrichment analyses of GO terms and KEGG pathways indicated significant enrichment of DEGs in terpenoid backbone biosynthesis pathways. Analysis of DEGs revealed the NAC transcription factor, which is crucial for plant growth regulation, stress response, and secondary metabolite biosynthesis. A total of 182 HhNACs were identified at the genome-wide level, named *HhNAC1* to *HhNAC182* according to their chromosomal positions. Numerous ABA-responsive cis-regulatory elements (CREs) were presented at upstream promoters of *HhNAC1* to *HhNAC182*. They demonstrated diversified tissue-specific expression profiling among stems, roots, and leaves of *H. helix*. Notably, *HhNAC93* was predominantly expressed in *H. helix* leaves. Correlation analysis unveiled a markedly positive relationship among ABA-induced *HhNAC93* expression, triterpenoid saponin accumulation, and the expression of essential saponin biosynthetic genes. HhNAC93 likely functions as a candidate regulator in triterpenoid saponin biosynthesis. These findings provide crucial evidence for further exploring the biological role of HhNAC transcription factor in *H. helix*.

## 1. Introduction

*Hedera helix*, a member of the Araliaceae family, is an evergreen climbing plant native to Europe. Due to its high horticultural value, it is widely distributed around the world nowadays [1]. In addition to its horticultural value, *H. helix* shows substantial medicinal potential, with bioactive compounds vividly demonstrating pharmacological efficacy in traditional and modern therapy. Continuous deepening research on *H. helix* has revealed its potent anti-inflammatory, antioxidant, and anti-neoplastic properties, including proliferation inhibition, apoptosis induction, and autophagy modulation in malignant cells [2,3], which have been scientifically verified and widely recognized in European medical practice. *H. helix* is currently recorded in the European Pharmacopoeia, with its leaf extracts showing the ability to treat respiratory diseases such as bronchitis and asthma [4]. Pharmacopeial quality standards specifically designate hederacoside C as the primary component, and its content directly impacts the quality of *H. helix*. Pentacyclic triterpenoid saponins, such as α-hederin, hederacoside B, hederacoside C, and hederacoside D, are the predominant specialized metabolites in *H. helix* [5]. Therefore, the biosynthesis of these bioactive triterpenoid saponins is of paramount importance for further anticancer development.

In planta, triterpenoid saponins biosynthesis consists of three stages: the mevalonic acid (MVA) pathway, the biosynthesis of 2,3-squalene oxide, and chemical modifications, for example, glycosylation, oxidation, and substitution of 2,3-squalene oxide, which collectively influence the structural diversity and bioactivity of the resulting saponins [6]. Triterpenoid saponin biosynthesis initiates in glycolysis, where glucose is converted to acetyl-CoA in the presence of 3-hydroxy-3-methylglutaryl-CoA reductase and 3-hydroxy-3-methylglutaryl-CoA synthase [7]. The resulting acetyl-CoA serves as a precursor in the MVA pathway, which synthesizes 3-isopentenyl diphosphate (IPP), and IPP is a universal C5 precursor for all terpenoid biosynthesis [8]. The biosynthetic pathway of squalene oxidation initiates with farnesyl pyrophosphate synthase, catalyzing the condensation of IPP and dimethylallyl diphosphate to form geranyl pyrophosphate [9]. The head-to-tail condensation of 2 farnesyl pyrophosphate produces squalene by reaction of squalene synthase. It is epoxidized by squalene epoxidase to yield 2,3-oxidosqualene and is the pivotal branch-point intermediate that determines the metabolic flux towards either sterol or triterpenoid saponins [10]. The committed step involves β-amyrin synthase, which catalyzes the diversification of the scaffold through the cyclisation of 2,3-oxidosqualene. Subsequent structural elaboration is achieved through a series of coordinated tailoring reactions, which are mediated by cytochrome P450 monooxygenases for oxidation, UDP-glycosyltransferases for glycosylation, and various acyltransferases [11]. These reactions collectively generate the structural diversity that is characteristic of triterpenoid saponins.

Under drought conditions, plants synthesize abscisic acid (ABA), and an ABA-dependent signaling pathway serves as a central hub for drought stress responses [12]. Emerging evidence demonstrates that this pathway orchestrates metabolic reprogramming to facilitate triterpenoid saponins accumulation, potentially mediated by ABA-responsive transcription factors (TFs) and key biosynthetic genes. After treating *Bupleurum chinense* with 100 μM ABA for 12 h, the content of saikosaponin A and saikosaponin C increased to varying degrees and began to decrease after 24 h [13]. A greater enhancement in saponin accumulation was shown by the 100 μM ABA treatment in comparison to the results that were obtained after treatment with 200 μM ABA. Transcriptome analysis revealed that BcIPPIs, BcFPSs, and BcBASs were downregulated to varying degrees within 1–3 d. In contrast, most bHLHs and biosynthetic genes were highly expressed within 12 h but downregulated after 24 h. At the same time, multiple TFs have been demonstrated to participate in the regulatory network controlling saponin biosynthesis. Overexpression of *DzHDZ32*, an HD-ZIP I transcription factor in *Dioscorea polystachya*, leads to significantly upregulated transcriptional levels of this transcription factor in leaves and elevated synthesis levels of diosgenin and its biosynthetic intermediates, such as cholesterol and β-sitosterol [14]. The ginsenosides Rh2 and Rg3 were significantly reduced in *Panax ginseng* roots overexpressing *PgERF120*, while the content of ginsenosides Rd and Rc were slightly increased. It is predicted that overexpression of *PgERF120* could increase the expression of *PgUGT1*, which might lead to an increased conversion of Rh2 and Rg3 into Rd, which is then converted to Rc, resulting in reduced levels of Rh2 and Rg3 [15]. PnMYB4 binds directly to the promoters of crucial saponin biosynthetic genes, such as PnSSs, PnSEs, and PnDSs, to inhibit saponin accumulation. PnMYB4 and PnMYB1 also act as positive regulators of saponin biosynthesis through interaction with the core transcriptional regulator PnbHLH. PnMYB4 inhibits the activation of the saponin biosynthetic gene by competing with PnMYB1 for binding to PnbHLHs, thereby disrupting the PnMYB1-PnbHLH complex [16].

The NAC family is a group of plant-specific regulators characterized by an N-terminal NAC domain, typically comprising approximately 150 amino acids [17]. The NAC domain is divided into five subdomains, with subdomain A potentially playing a role in forming functional light regulators, and subdomains C and D possessing positive charges. These three domains are highly conserved and are DNA binding sites. Subdomains B and E can be linked to multiple functions of the NAC gene. NAC TFs are crucial for plant growth and development, participating in various biological processes such as stress response and secondary metabolism [18]. *PgNAC41-2* expression levels are significantly positively correlated with Re, Rb2, and total saponin contents in *P. ginseng* [19]. Furthermore, heterologous expression of *PgNAC41-2* results in elevated accumulation of these saponins. Concurrently, the expression levels of key saponin biosynthetic genes, including *Pgβ-AS-1*, *PgSS-1*, *PgSE2-4*, and *PgFPS-22*, are markedly upregulated. The interaction between reactive oxygen species (ROS) and ABA plays a significant role in drought responses and tolerance. Under drought stress, ROS within plants has been shown to induce the synthesis of endogenous ABA. Furthermore, under the induction of ABA signaling, the RcNAC091 has been observed to bind to the promoter region of the *RcWRKY71*, thereby significantly enhancing its expression levels [20]. This enhances oxidative enzyme activity, protecting roses from ROS damage and improving drought tolerance.

The influence of NAC on triterpenoid saponin biosynthesis has been studied in multiple medicinal plants. However, its downstream genes and hormone-induced responses in *H. helix* remain unclear. It is crucial to excavate the candidate NAC for triterpenoid saponins in order to enhance *H. helix*’s tolerance to abiotic stresses and the accumulation of medicinal compounds. This study utilized whole-transcriptome sequencing to investigate the effects of ABA on *H. helix*. It aimed to identify TFs involved in the biosynthesis or regulation of triterpenoid saponins. Additionally, the expression of genes associated with ABA and key enzymes in the triterpenoid saponin biosynthetic pathway was analyzed, offering insights into the mechanisms of *H. helix*’s response to abiotic stress.

## 2. Materials and Methods

### 2.1. Acquisition and Experimental Treatments of H. helix Plants

The plants were gathered in November 2024 from the research base of Songyang Institute, Zhejiang Chinese Medicine University (119.57° E, 28.36° N), and were identified as *Hedera helix* by Professor Xiaoxia Shen (Zhejiang Chinese Medicine University), and belonged to the Araliaceae family, genus *Hedera*. Healthy *H. helix* plants with similar growth patterns that were free of diseases were collected. Organ-specific expression was performed using samples harvested from roots, leaves, and stems. Appropriately timed exogenous ABA treatment can regulate plant physiology, thereby enhancing their capacity to respond to drought [21]. Exogenous ABA was used to study their impact on the HhNACs’ expression levels. According to research reports, treatment with 100 μM ABA significantly increases the content of saponins within a short period after treatment compared to treatment with 200 μM ABA [13]. Therefore, 100 μM ABA was adopted as the reference concentration in this experiment. To further investigate the effect of different treatment durations with 100 μM ABA solution on the accumulation of saponins in *H. helix*, preliminary experiments were conducted; thereby, 100 μM ABA was uniformly sprayed on the *H. helix* plants, with water containing 0.5% ethanol serving as the control group, and samples were collected at 0, 6, and 12 h, respectively. Samples with three replicates were stored in liquid nitrogen and preserved at −80 °C for RNA extraction.

### 2.2. Quantification and Composition of Triterpenoid Saponins in H. helix

*H. helix* leaves were collected at 0, 6, and 12 h subjected to 100 μM ABA, dried at 55 °C, ground, and sieved through a 50-mesh screen. The powdered leaves were accurately measured to 1 g, extracted with 10 mL of high-performance liquid chromatography (HPLC) grade methanol (Macklin, Shanghai, China), and an ultrasonic extractor (KQ-100DE, Supmile, Kunshan, China) was used to extract ultrasonically for 1 h at 50 °C. After ultrasonication, the supernatant was obtained by centrifuging at 10,000 rpm for 10 min using a NO.2 rotor in a high-speed centrifuge (H1850R, Cence, Changsha, China). The total saponin was quantified using the vanillin-perchloric acid colorimetric method with a Cary 60 UV-vis spectrophotometer (G6860A, Agilent, Santa Clara, CA, USA) at 560 nm, employing oleanolic acid as a standard reference [22]. The linear equation of oleanolic acid was y = 0.3186x − 0.0174, *R^2^* = 0.9995.

The powdered *H. helix* leaves (0.5 g) were implemented using 5 mL methanol-assisted ultrasonic extraction at 80 kHz for 40 min at 50 °C. The filtrate was cooled to 25 °C, brought to a constant volume through an HPLC-grade methanol, and was ready for the determination of α-hederin and hederacoside C employed on an Agilent 1260 HPLC instrument with an Agilent ZORBAX StableBond column (SB-C18) at 205 nm following a published manual [23]. The standard α-hederin (Sigma-Aldrich, Saint Louis, MO, USA) and hederacoside C (Sigma-Aldrich) were adopted and quantified against linear equations, y = 0.1051x − 0.0997 (*R*^2^ = 0.9997) and y = 1.6949x + 0.0097 (*R*^2^ = 0.9993), respectively. Data were expressed as mean ± standard deviation (SD). Statistical analysis was performed using IBM SPSS 27.0 and was assessed using Student’s *t*-test. *** indicates significant differences between the control and treatment groups at the *p* < 0.001 level.

### 2.3. Transcriptome Sequencing and Bioinformatic Analyses

Total RNA was extracted from each *H. helix* sample using a rapid RNA extraction kit (Huayueyang Biotech, Beijing, China). The concentration of RNA was measured using a NanoDrop 2000 spectrophotometer (Thermo Fisher, Wilmington, NC, USA). The sequencing library of each *H. helix* sample was prepared from 1 μg of the corresponding RNA through the mRNA Library Prep Kit (Yeasen, Shanghai, China) following the instructions. The library concentration was first evaluated on a Qubit Fluorometric Quantification (Thermo Fisher) and required more than 1 ng·μL^−1^. Quantitative PCR was employed to accurately measure the library’s effective concentration, ensuring it exceeded 2 nM and achieved the sequencing quality standards. The BMKCloud platform (www.biocloud.net, accessed on 13 June 2025) was used for quality control preprocessing of raw data, including removing adapter sequences, low-quality reads, and excessive poly-N ratios to acquire high-quality clean data. All bioinformatics analyses were performed with high-quality clean data.

### 2.4. Identification of DEGs and PCA Plot

DEGs identification and PCA plot were integrated within DESeq2 v4.11 and online BMKCloud (www.biocloud.net/, accessed on 13 June 2025) as described [24]. A PCA plot was perpetrated on the transcript matrix after a variance stabilizing transformation to examine the differences in overall expression between samples and the clustering of groups. DEGs analysis between two different treatments was executed with DESeq2 v4.11, obeying the negative binomial distribution for modeling the RNA seq data and identifying DEGs. The Benjamini–Hochberg procedure adapts discrete *p*-values to control the false discovery rate (FDR). The DEGs were delimited with |log2FC| ≥ 2 (FC, fold change) and randomized *p*-values < 0.01.

### 2.5. Functional Enrichment and Pathway Analysis

The DEGs were synchronously implemented through gene ontology (GO) and the Kyoto Encyclopedia of Genes and Genomes (KEGG) analyses. GO annotation used clusterProfiler, employing the Wallenius distribution for modeling preferences, while KEGG enrichment was integrated within KOBAS v2.8.3 and clusterProfiler [25]. Hypergeometric tests were used to evaluate the GO and KEGG enrichment significance, with FDR ≤ 0.05 and *p*-values < 0.01.

### 2.6. Comprehensive Identification of NAC Family Members in H. helix

The *H. helix* genome assembly is publicly available from the NCBI Genbank under accession number GCA_947179155.2. The *Arabidopsis thaliana* NACs were retrieved on the TAIR server (www.arabidopsis.org, accessed on 12 June 2025), and searched against the chromosome-level *H. helix* genome to identify NAC members through the TBtools toolkit v2.331 that meet the criteria of an E-value < 1 × 10^−10^ [26]. Two hidden Markov models for NAM (PF01849) and NAC (PF02365) were recruited from PFAM v37.4 (http://pfam.xfam.org/, accessed on 23 July 2025), and targeted against the aforesaid *H. helix* NAC family members. They were ulteriorly validated using SMART (https://smart.embl.de/, accessed on 23 July 2025). The obtained GFF annotation file facilitated the visualization of HhNAC members on the *H. helix* chromosome. After checking their conserved motifs, they were eventually defined as *HhNAC1-HhNAC182*.

### 2.7. Phylogenetic Tree, and Multi-Species Collinearity Analysis of HhNAC Proteins

The *A. thaliana* and *O. sativa* genomic information was retrieved from Ensembl Plants (https://plants.ensembl.org/, accessed on 12 June 2025). A phylogenetic tree of *H. helix*, *O. sativa*, and *A. thaliana* NAC proteins was conducted through a neighbor-joining algorithm, running 1000 bootstrap repetitions within MEGA11 analyzer [27].

Using MCScanX v1.0.0 software, we performed collinearity analysis on genomic and annotation data from *H. helix*, *A. thaliana*, *P. ginseng*, *P. notoginseng*, and *O. sativa* with an E-value threshold of 1 × 10^−5^, resulting in gene duplication events within NAC members and their intraspecific or interspecific collinearity relationships. Results were visualized using the Advanced Circos module in TBtools. Key thresholds defining collinearity blocks were set as follows: each block must contain at least five gene pairs, with no more than 25 gene positions separating adjacent gene pairs.

### 2.8. Exon-Intron Structure, Conserved Motifs, and Cis-Regulatory Elements Analyses

HhNAC1-HhNAC182 proteins were employed to excavate conserved motifs using the Simple MEME Wrapper module in TBtools, v2.371, with a motif amount of 10, motif widths of 6–50, and an E-value threshold of 1 × 10^−10^. Structural domains for *HhNAC1*-*HhNAC182* were excavated from NCBI (www.ncbi.nlm.nih.gov, accessed on 26 July 2025). They were combined with annotation files and their exon and intron information were visualized in TBtools. The PlantCare portal (http://bioinformatics.psb.ugent.be, accessed on 27 July 2025) was utilized to discover CREs within the initial 2000 bp upstream of the 182 *HhNAC* genes promoter sequences in *H. helix*. TBtools was adopted, which screened out the proposed CREs and finally visualized the results.

### 2.9. Total RNA Extraction and qPCR Analysis

Total RNA of ABA-inducible *H. helix* leaves was achieved as mentioned above. A total of 1 μg RNA was transcribed into first-strand cDNA with a Reverse Transcription Kit (Accurate Biology, Changsha, China) and subsequently stored at −80 °C. Specific primers of 24 *HhNAC* genes were designed at the PrimerQuest portal (https://sg.idtdna.com, accessed on 28 July 2025) and synthesized by Zhejiang Sunya Biotech (Hangzhou, China) (Table S7). The Quantitative real-time polymerase chain reaction (qRT-PCR) assay was implemented in a qRT-PCR instrument (ABI7500, Waltham, MA, USA) in triplicate as previously reported [28]. The β-actin (accession number KU942510) was utilized as a reference control due to its stable expression [29]. The 2^−ΔΔCt^ protocol was employed for calculating the relative expression of *HhNAC* genes [30], which were visualized through TBtools and GraphPad Prism v9.5.1. A correlation analysis was implemented with IBM SPSS 27.0.

## 3. Results

### 3.1. Saponin Accumulation in H. helix Under Exogenous ABA Treatment

**Figure 1:**
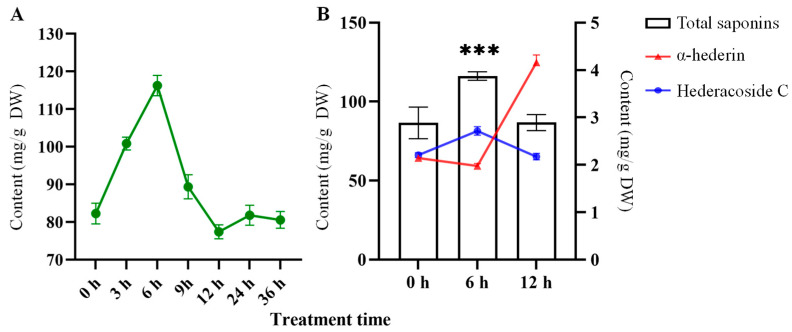
Total saponin accumulation in *H. helix* leaves at various time points with treatment with 100 μM ABA (**A**) and contents of total saponins (**B**), α-hederin, and hederacoside C in *H. helix* at 0, 6, and 12 h after ABA treatment. Significant differences: *** (*p* < 0.001).

The 100 μM ABA was sprayed onto the leaves of *H. helix*, which were collected at 0, 3, 6, 9, 12, 24, and 36 h. Total saponin accumulation increased significantly between 0 and 6 h after ABA treatment. Accumulation accelerated rapidly during the initial 0–3 h period, slowed slightly between 3 and 6 h, peaked at 6 h, and then declined. At 12 h, total saponins had reached a trough, and afterwards, they gradually rebounded. Therefore, we selected 0, 6, and 12 h as the optimal timing for the ABA treatment (Figure 1A). To investigate saponin accumulation in *H. helix* after treatment with 100 μM ABA, the total saponin, α-hederin, and hederacoside C in *H. helix* leaves were measured at various intervals. Total saponin showed a significant increase from 0 to 6 h, achieved a peak at 6 h, and gradually declined and returned to baseline by 12 h (Figure 1B). HPLC was utilized to quantify the dynamic changes in α-hederin and hederacoside C in *H. helix* leaves after ABA treatment at various times. The results revealed distinct accumulation patterns between the two saponin components after ABA treatment for 6 h. The α-hederin decreased slightly at 6 h and then increased at 12 h; however, hederacoside C showed the opposite trend, reaching a peak at 6 h and then gradually decreasing. It is suggested that hederacoside C and α-hederin may be converted into each other in a dynamic equilibrium. Hence, the results demonstrate that ABA treatment may promote the accumulation of triterpenoid saponins in *H. helix*, and ABA is beneficial for the synthesis and accumulation of the active component, hederacoside C.

### 3.2. Transcriptome Analysis of H. helix Under Exogenous ABA Treatment

**Figure 2:**
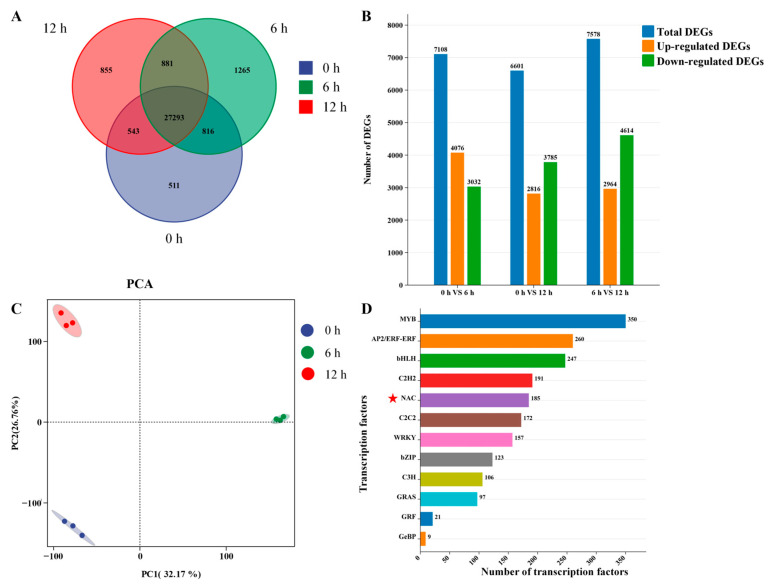
Transcriptomic profiling of ABA-treated *H. helix*. (**A**) Number of DEGs at 0, 6, and 12 h after ABA treatment. (**B**) DEGs among 0, 6, and 12 h after ABA treatment. (**C**) Principal component analysis. (**D**) Number of transcription factors under ABA treatment.

To study the transcriptional regulation mechanism under exogenous ABA treatment, a 100 μM ABA solution was evenly sprayed onto *H. helix* leaves, and RNA sequencing was performed on samples treated for 0, 6, and 12 h (Table S1). The experiment generated a total of 293.13 GB of clean data, containing an average of 23,517,142 high-quality sequencing reads (Q30 values ranging from 93.00% to 93.82%). In the ABA samples from the control (0 h), a total of 29,163 genes were identified, whereas at 6 and 12 h post-treatment, 30,255 and 29,572 genes were detected, respectively (Figure 2A). The three experimental groups showed unique transcriptional profiles, with biological replicates within each group clustering closely, indicating high reproducibility across conditions. This study examined the impact of exogenous ABA on *H. helix*’s biological accumulation by identifying differentially expressed genes (DEGs) at various time points. ABA treatment for 6 h resulted in 7108 DEGs, with 4076 upregulated and 3032 downregulated, while 12 h of treatment led to 6601 DEGs, comprising 2816 upregulated and 3785 downregulated, compared to the control (Figure 2B). Principal component analysis (PCA) showed that gene expression changed significantly after ABA treatment (Figure 2C), with a clear boundary between the control (0 h) and the treatment groups (6 and 12 h). Among them, the 6 h treated group not only exhibited the highest number of DEGs, but also had a significantly greater number of upregulated DEGs compared to those at 12 h. These transcriptional findings are consistent with the HPLC-based metabolic profiling data. Crucial genes involved in the synthesis and accumulation of *H. helix* saponins may be highly expressed in *H. helix* leaves for 6 h after ABA treatment. The functions of all screened DEGs were revealed by this study through the performance of GO enrichment analysis.

**Figure 3:**
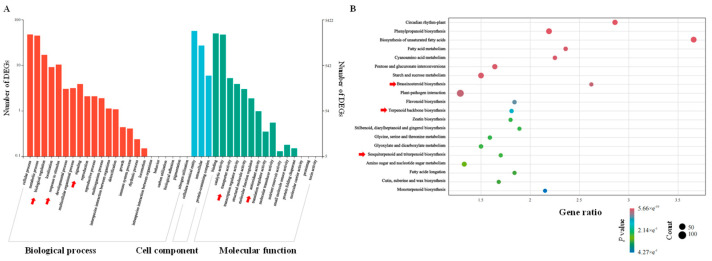
GO (**A**) and KEGG (**B**) enrichment analysis of DEGs under ABA treatment.

The aforementioned DEGs can be classified into three primary categories: biological processes, cellular components, and molecular functions (Figure 3A). Cell processes and metabolic processes rank first and second among biological processes, and genes involved in biological regulation and responses to external stimulation showed high expression under ABA induction, indicating that ABA significantly increased cell biological activity and responses to external stimulations. Among the molecular functional categories, binding functions and catalytic activities were most notable, and genes related to transcriptional regulation and antioxidant activity were also significantly enriched. Triterpenoid saponins can act as reactive oxygen species cleansers, and the enrichment of signaling pathways suggests that the accumulation of triterpenoid saponins induced by abscisic acid in *H. helix* may involve complex regulatory networks, thus having antioxidant effects, and this ability is consistent with the enrichment results. In addition, KEGG pathway enrichment analysis of DEGs revealed that these genes participated in 20 different pathways (Figure 3B), among which fundamental metabolic pathways essential for plant growth and development were significantly enriched (*p* < 0.05). KEGG enrichment analysis showed that DEGSs were predominantly enriched in biosynthetic pathways, including phenylpropanoid biosynthesis, brassinosteroid biosynthesis, terpenoid backbone biosynthesis, sesquiterpenoid biosynthesis, and triterpenoid biosynthesis. After ABA treatment, the terpenoid skeleton biosynthesis and sesquiterpene and triterpene biosynthesis pathways were highly enriched (*p* < 0.05). This finding suggests that the two pathways under scrutiny are of significant importance in the response of *H. helix* to ABA signal induction. The terpenoid skeleton biosynthesis is crucial for forming the basic structures of sesquiterpenes and triterpenoid saponins, and it also plays a role in synthesizing secondary metabolites such as plant sterols and monoterpenes. The specific accumulation demonstrates that ABA regulates the accumulation and synthesis of *H. helix* saponins. ABA can activate the synthesis network of triterpenoid saponins compounds in *H. helix* in response to hormone stimulation, thereby inducing the directed synthesis of triterpenoid saponins.

### 3.3. Transcription Factors Exhibiting Differential Expression in Response to Exogenous ABA

TFs are crucial regulators of plant growth and development. This study set out to examine the impact of exogenous ABA on the expression of TF in *H. helix* and identified gene differential expression among various TFs under different treatment times. A total of 2578 TFs were identified and categorized into 20 families, such as AP2/ERF, bHLH, MYB, NAC, WRKY, and bZIP (Figure 2D). The transcriptome shows that there are 185 NAC TFs, and the expression of these TFs showed specificity under different treatment times. Three new genes were discovered in the transcriptome: NewGene_10257, NewGene_16559, and NewGene_17048. 24 NAC TFs were differentially expressed in *H. helix* leaves after 0 h of treatment, 31 after 6 h, and 34 after 12 h. Since the NAC TF is closely related to triterpenoid saponin synthesis, this study focuses on bioinformatics analysis of the NAC TF and analyzes DEGs under ABA treatment.

### 3.4. Identification and Chromosome Localization of NAC TF in H. helix

**Figure 4:**
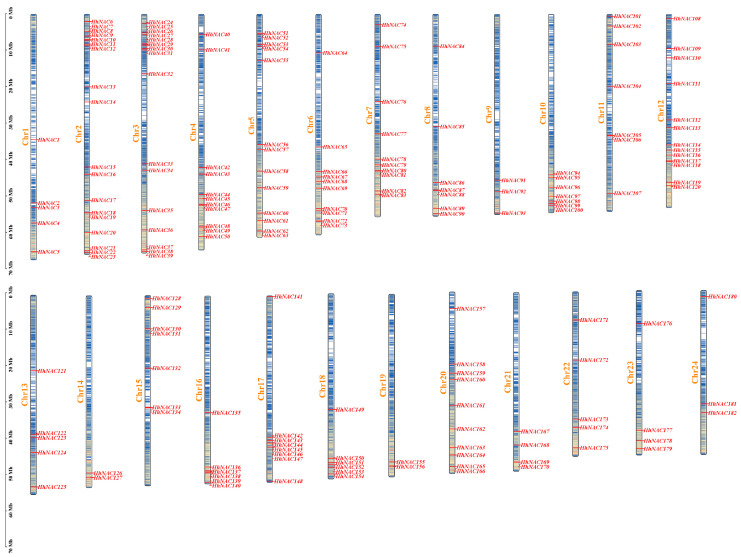
Localization of *HhNAC* genes on the 24 chromosomes in *H. helix*.

A thorough investigation was conducted on the *H. helix* genome, leading to the identification of 182 NAC proteins (Table S2). According to their distribution on 24 chromosomes, these 182 HhNACs were named *HhNAC1* to *HhNAC182* in order (Figure 4). The mass of the HhNACs ranged from 23.18 kDa (HhNAC78) to 75.18 kDa (HhNAC66), and theoretical isoelectric points (pI) ranged from 4.9 (HhNAC161) to 10.2 (HhNAC78). Instability index (II) ranged from 51.54 (HhNAC104) to 74.71 (HhNAC93), while aliphatic index (AI) ranged from 34.29 (HhNAC38) to 54.54 (HhNAC93). The grand average of hydropathy (GRAVY) ranged from −0.825 (HhNAC164) to −0.413 (HhNAC161). Instructively, all HhNAC proteins were hydrophilic, and all HhNAC proteins were localized in the nucleus.

### 3.5. Collinearity Analysis of HhNAC TF in H. helix

**Figure 5:**
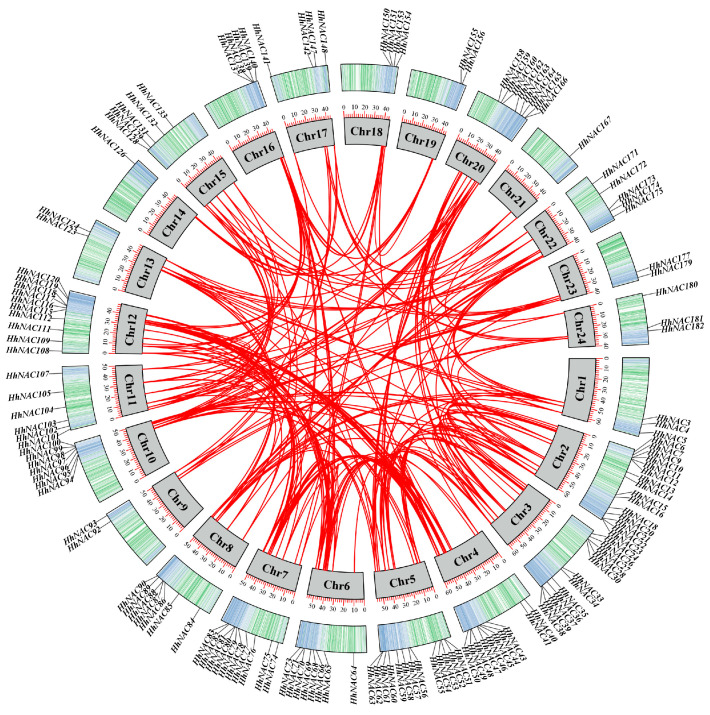
Gene duplication events of HhNACs in *H. helix*.

**Figure 6:**
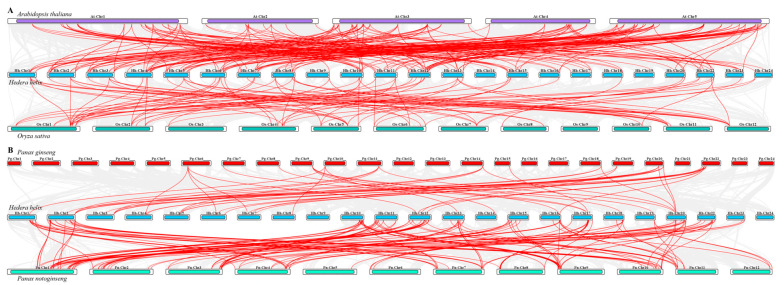
Collinearity analysis of NAC genes among *H. helix*, *A. thaliana*, and *O. sativa* (**A**), and among *H. helix*, *P. ginseng*, and *P. notoginseng* (**B**).

The intra-species collinearity relationships of HhNACs revealed 250 gene duplication events among these genes in *H. helix* (Figure 5). Additionally, the inter-species collinearity relationship of *NAC* genes among *H. helix*, *A. thaliana,* and *O. sativa* was evaluated, finding a notable evolutionary divergence (Figure 6A) with more shared conserved homologous gene pairs with *A. thaliana* (187 pairs) than with *O. sativa* (69 pairs), suggesting greater genomic conservation of *NAC* genes in dicots compared to monocots. Among the same Araliaceae family, a collinearity analysis of NAC genes among *H. helix*, *P. ginseng,* and *P. notoginseng* was performed (Figure 6B). It was interesting to observe that PgNACs involved in gene duplication events were localized on chromosomes 6, 7, 8, 9, 10, 14, 15, 18, 19, 20, and 22 of *P. ginseng*, whereas PnNACs were distributed across all chromosomes of *P. notoginseng*. Conversely, HhNACs distributed on chromosomes 3, 4, 5, 6, 7, 8, and 14 participated exclusively in gene duplication events with PgNACs. NAC genes distributed on chromosomes 10, 11, 15, 22, and 23 specifically participated in gene duplication events with PnNACs. From the results, it was evidenced that NAC genes on chromosomes 9 and 24 of *H. helix* did not undergo gene duplication events with those of *P. notoginseng* or *P. ginseng*. In the quantitative view, compared to PgNACs, HhNACs, and PnNACs presented more duplication events, suggesting that PnNACs and HhNACs shared a closer phylogenetic relationship during the evolutionary process of the Araliaceae family.

### 3.6. Phylogenetic Analysis of HhNACs and Other Homologous NAC Proteins

**Figure 7:**
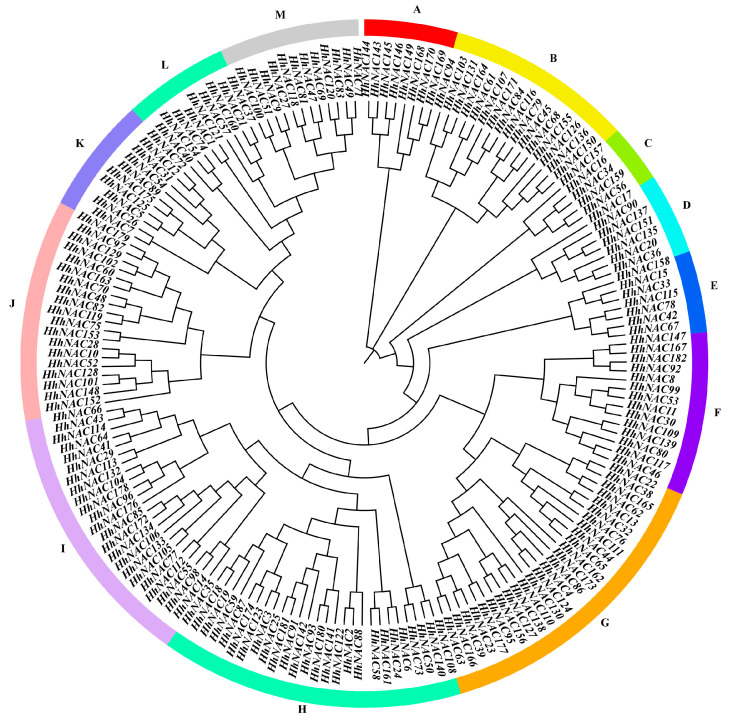
Phylogenetic analysis of NAC members in *H. helix*. The phylogenetic tree was generated using the neighbor-joining method and 1000 bootstrap values in MEGA 11 software.

A phylogenetic tree was constructed for 182 HhNAC proteins (Figure 7). HhNACs were classified into 13 subgroups, designated A through M. Subsequently, a phylogenetic tree was constructed from NAC sequences of *H. helix*, *A. thaliana* (175), and *O. sativa* (175), revealing that HhNACs were classified into 16 distinct clades (Figure S1; Table S4). According to the previous studies on AtNACs and OsNACs, the 16 subgroups were defined as follows: ANAC023, VOZ, ANAC006, New, SND, NAM, NAC084, ONAC129, ANAC013, NTL, VND, TIP, CUC, COG4, ATAF, and NAP. Considering the grouping results, it was obvious that most HhNAC proteins were in the same branch as AtANAC proteins, compared with OsNAC proteins. Therefore, it can be inferred that *H. helix* shares a closer evolutionary relationship with the dicot plant *A. thaliana* than with the monocot plant *O. sativa*. These findings strongly suggested that HhNACs displayed a high conservation within dicots compared to monocots during the evolutionary process.

### 3.7. Conserved Motifs and Gene Architecture of HhNAC Family Members

**Figure 8:**
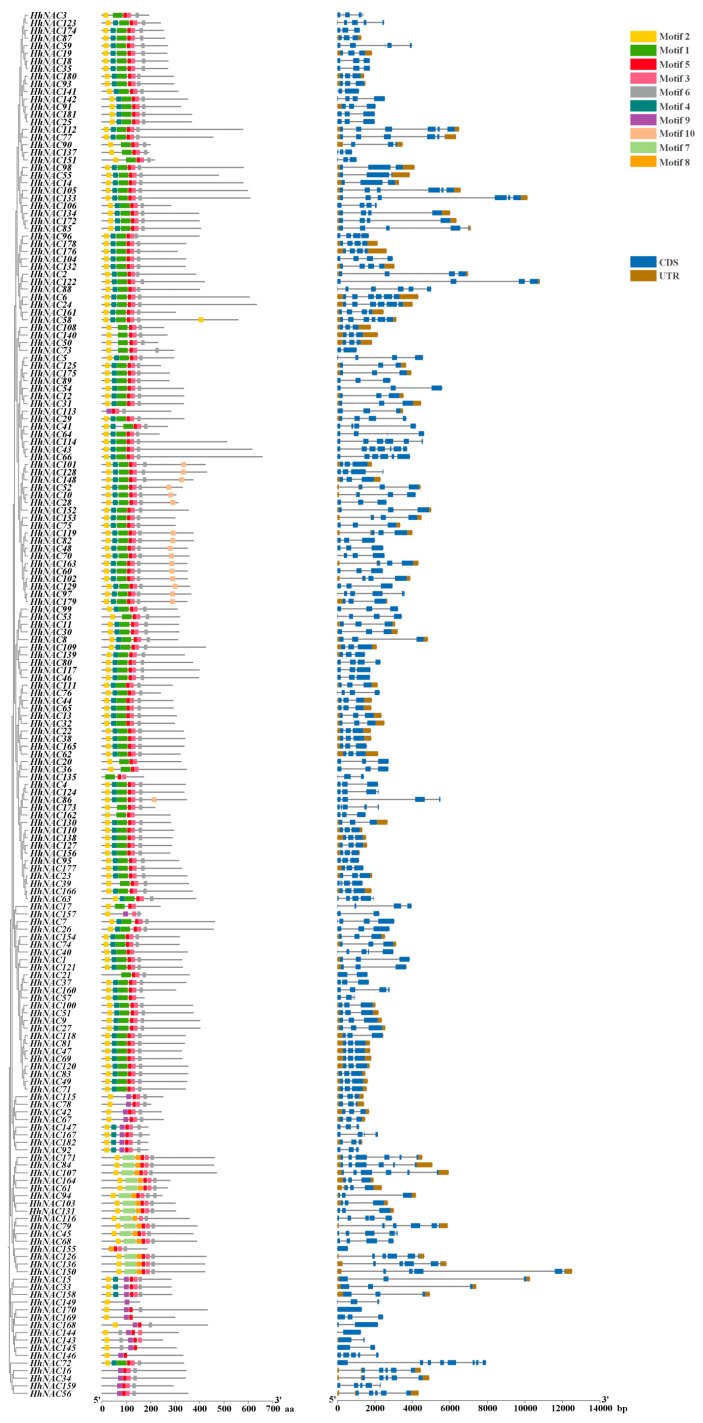
Conserved motifs and gene structure of HhNAC members in *H. helix*. The blue and brown correspond to the coding sequence (CDS) and untranslated region (UTR), respectively. The remaining colored blocks represent different conserved domains.

A domain prediction analysis of 182 HhNAC proteins confirmed the presence of a conserved NAM domain in all HhNAC family members, in accordance with the NAM domain exhibiting a consistent spatial architecture across NAC proteins in other plants (Figure 8). When comparing the exon–intron structures of 182 HhNACs, the amount of coding sequences (CDS) varied from three to seven. To further elucidate the structural features of HhNAC proteins, a total of 10 conserved motifs were extracted and analyzed (Figure S2). HhNAC proteins presented 4 to 7 conserved motifs, with motif 5 harbored in all HhNAC proteins. Motifs 1–4 were frequently observed in HhNAC proteins, suggesting their fundamental role in the plant NAC family and highlighting the evolutionary conservation of motifs 1–5. The NAM domain comprised five conserved subdomains, designated A–E (Figure S3). Among these, subdomains A, C, and D exhibited high evolutionary conservation across different species. Analysis of conserved motifs in HhNACs indicates that motifs 3 and 1 correspond to NAC protein subdomains A and C, respectively, while motif 5 encompasses domains D and E, and observing the conserved motifs in 182 HhNAC proteins reveals that the vast majority of genes simultaneously contain 2–3 of the aforementioned motifs. This suggests that NAC genes have evolved relatively conservatively in *H. helix*, although the absence of modicum motifs may be due to amino acid site mutations that occurred during evolution. Phylogenetic analysis indicated that HhNAC proteins within the same clade possessed similar gene architecture and conserved motif compositions. Overall, the exon–intron structures of HhNACs were diverse, but HhNACs within the same branch exhibited highly similar characteristics, providing further molecular evidence for the basic structural conservation of the HhNAC family.

### 3.8. Analysis of Cis-Regulatory Elements in the Promoter Sequences of HhNACs

**Figure 9:**
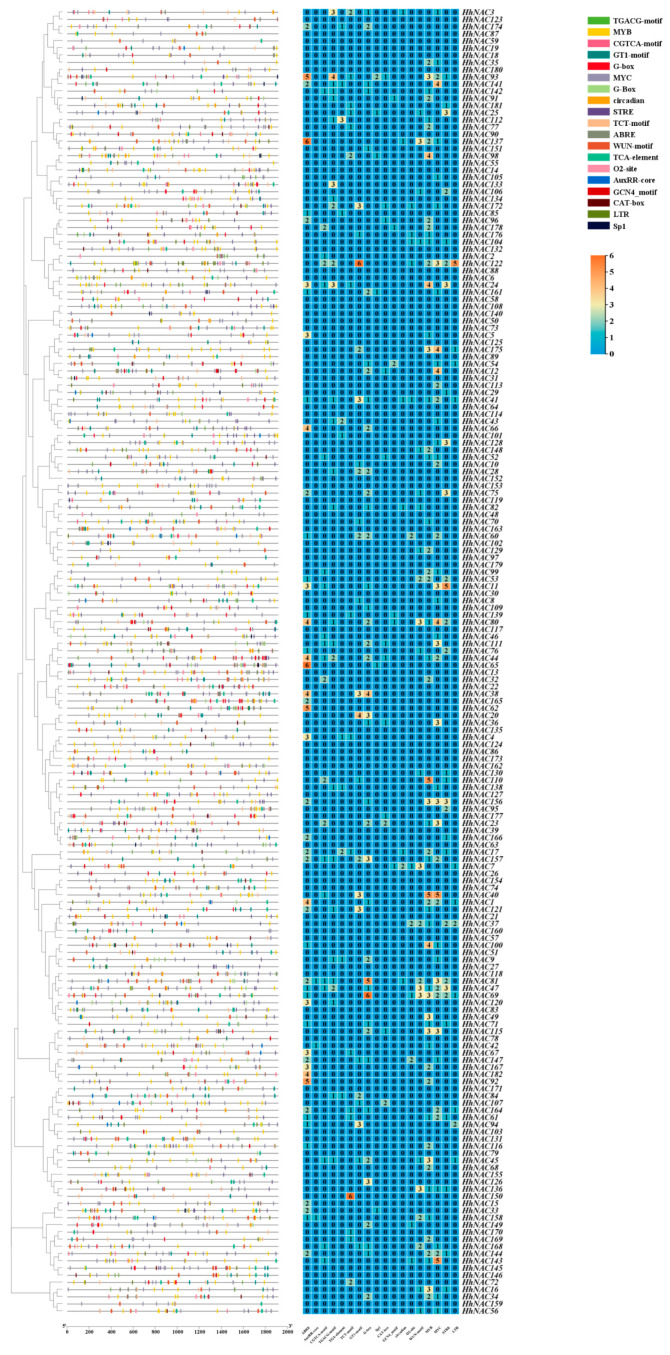
Cis-regulatory elements analysis of HhNACs in *H. helix*. The colored blocks on the left represent the different cis-regulatory elements within the promoter region of the HhNACs. The orange and blue colors in the heatmap represent high and low relative gene expression, respectively.

The role of CREs in the modulation of gene transcription levels during the processes of plant growth, development, and stress responses has been well-documented. Systematically discovering the upstream 2000 bp of 182 HhNACs revealed 18 distinct types of CREs. A total of 701 CREs were excavated and could be classified into four primary groups based on their annotated function, including light response (153), growth and development (44), stress response (317), and hormone response (187) (Figure 9). Hormone-responsive CREs were massively enriched, for instance, ABRE, AuxRR, CGTCA-motif, TGACG-motif, and TGA-element. Among these, ABRE accounted for a relatively high proportion, indicating that HhNACs were likely to be induced by ABA treatment. The promoter sequences of HhNACs also harbored several CREs that were responsive to methyl jasmonate (MeJA) signaling, such as CGTCA-motif, TGACG-motif, and MYC, suggesting their transcriptional regulation might be influenced by MeJA. HhNACs comprised numerous CREs responsive to abiotic factors like low temperature, drought, mechanical wounding, and anaerobic induction. The presence of diverse CREs in HhNACs enhanced the potential of *H. helix* to cope with adverse environment and to confer stress resistance, thereby acting as an essential function in modulating growth and development, hormone transduction, and stress adaptation in multivariate environments.

### 3.9. Expression Profiling of HhNACs in H. helix Different Organs

**Figure 10:**
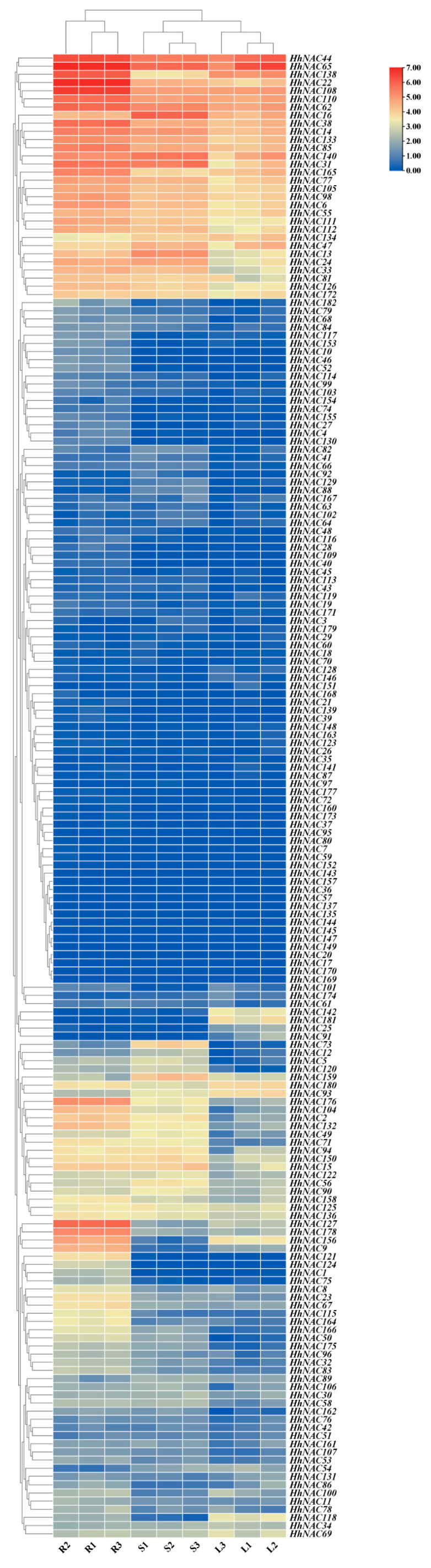
Expression profiles of 182 HhNACs at different organs in *H. helix*. R, S, L represent roots, stems, leaves, and the numbers 1–3 represent biological replicates. The red and blue colors in the heatmap represent high relative expression and low relative expression, respectively. The red and blue colors in the heatmap represent high and low relative gene expression, respectively.

A heatmap analysis of transcriptomic data for *HhNAC1*-*HhNAC182* genes in *H. helix* roots, leaves, and stems demonstrated distinctly tissue-specific expression profiling (Figure 10). These genes were predominantly expressed in roots and stems, with fewer showing dominant expression in leaves (Table S5). The expression levels can be ranked as follows: roots > stems > leaves. Further analysis of tissue-specific expression profiling indicated that *HhNAC22*, *65*, *108*, and *44* were primarily expressed in roots. Genes preferentially expressed in stems included *HhNAC16*, *13*, *140*, and *31*. Intriguingly, *HhNAC93*, *180*, and *134* were specifically expressed in leaves. Depending on the accumulation levels of *H. helix* saponins in different organs, leaves > stems > roots, these three genes that were highly expressed in leaves might participate in saponin biosynthesis in *H. helix*, which should receive more attention. Additionally, it was noteworthy that multiple genes, such as *HhNAC16*, *65*, *44*, *138*, *108*, *110*, and *62*, maintained a stable high expression level in these examined organs. The findings suggested that they might play a central role in regulating the fundamental growth processes in *H. helix*.

### 3.10. Expression Profiling of HhNACs Under ABA Treatment

**Figure 11:**
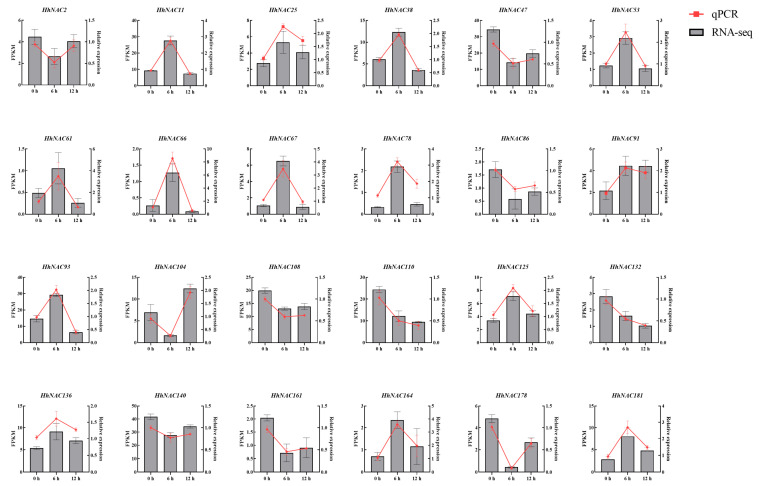
Expression profiling of 24 differentially expressed HhNACs in *H. helix* at 0, 6, and 12 h after ABA treatment.

Since there was an abundant ABA-responsive CREs presentation, expression patterns of HhNACs treated with ABA for 0, 6, and 12 h were examined using transcriptome sequencing. The results demonstrated that, in comparison with the control (0 h), 24 and 31 *HhNAC* genes exhibited differential expression for 6 and 12 h under ABA treatment, respectively. Considering that, total saponins content showed a significant rise from 0 to 6 h, peaked at 6 h, and followed that with a decrease in *H. helix* leaves exposed to ABA treatment. According to this trend, it could be inferred that the candidate HhNACs associated with saponin biosynthesis were likely to appear at 6 h. As shown in the results (Figure 11), among the 24 differentially expressed HhNACs, 14 genes (*HhNAC11*, *HhNAC25*, *HhNAC38*, *HhNAC53*, *HhNAC61*, *HhNAC66*, *HhNAC67*, *HhNAC78*, *HhNAC91*, *HhNAC93*, *HhNAC125*, *HhNAC136*, *HhNAC164*, and *HhNAC181*) were highly consistent with the ABA-induced saponin accumulation. The upregulation trends of *HhNAC66*, *HhNAC67*, *HhNAC93*, *HhNAC11*, and *HhNAC38* were more significant. Combining the expression patterns of these genes with the CARs in their promoter regions revealed that *HhNAC66*, *HhNAC67*, *HhNAC93*, *HhNAC11,* and *HhNAC38* had more ABRE elements than other *HhNAC* genes, typically 3–4 elements. *HhNAC93* possessed the greatest ABRE elements (5), providing evidence for its significant expression following ABA treatment. Notably, *HhNAC93* had a high basal expression at 0 h, and its relative expression increased the most after 6 h of ABA treatment, reaching 3.03 times than that at 0 h. The expression of these HhNACs reached its maximum at 6 h post-treatment and subsequently declined gradually. This finding indicated a positive correlation between the accumulation of triterpenoid saponins and the expression of these genes, suggesting a regulatory role for these genes in the process of ABA-induced saponin biosynthesis in *H. helix*. The consistent results between qRT-PCR and RNA-Seq confirmed the data’s reliability, strongly supporting the crucial role of HhNACs in connecting ABA signaling to saponin accumulation.

### 3.11. Expression Profiling of Saponin Biosynthetic Genes Under ABA Treatment

**Figure 12:**
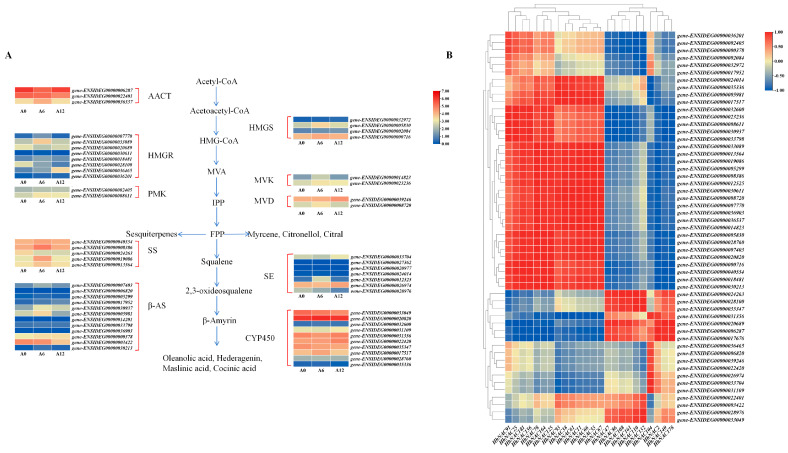
Expression proofing of triterpenoid saponin biosynthetic genes under ABA treatment (**A**) and correlation analysis between *HhNAC* genes and triterpenoid saponin biosynthetic genes (**B**). Red and blue blocks represent high and low relative gene expression, respectively. A significant positive correlation was identified between *HhNAC* genes and the triterpenoid saponin biosynthetic genes (Pearson’s r = 0.80, *p* < 0.001).

Crucial enzymes like HMGR, HMGS, MVK, PMK, and MVD associated with the triterpenoid saponins biosynthesis were pinpointed (Table S6). These genes were integral to the mevalonate pathway, supplying necessary reaction precursors (Figure 12A). The biosynthesis pathway also included enzymes such as SS, SE, and FPS, which participated in the biosynthesis of 2,3-oxidosqualene, a compound that was crucial for the biosynthesis of various triterpenoid compounds. β-AS, UGT, and CYP450 were key enzymes involved in the biosynthesis of *H. helix* saponins, and β-AS was an essential enzyme for formatting the saponin skeleton, primarily acting to cyclize 2,3-oxidosqualene, thereby creating the conditions for subsequent modification of the saponin skeleton by UGT and CYP450. The FPKM values of these 53 enzymes associated with the triterpenoid saponin synthesis at 0, 6, and 12 h were examined (Table S7). Following 6 h of ABA treatment, 38 genes increased, suggesting their potential function in the biosynthesis of triterpenoid saponin in *H. helix*. Notably, genes involved in the mevalonate pathway, including HMGR, MVK, and PMK, exhibited rapid induction at 6 h. Conversely, expression levels of the saponin biosynthetic genes, such as β-ASs and CYP450s, peaked at 12 h. The findings indicated that transcriptional activation of the metabolic pathway occurred sequentially. Additionally, a significant correlation was found between the ABA-responsive HhNACs (*HhNAC93*, *38*, *61*, *11*, *66*, *53*, and *67*) and the saponin biosynthetic genes (AACT, HMGS, HMGR, MVK, PMK, MVD, SS, SE, β-AS, and CYP) at 6 h. This strong correlation indicated that the aforementioned HhNACs were almost associated with the saponin biosynthesis. When combined, the tissue-specific expression of HhNACs, the ABA-treated expression profiling, and the correlation with saponin biosynthetic genes, suggested that *HhNAC93* was likely a highly effective regulatory factor in the accumulation of ABA-induced triterpene saponins in *H. helix*.

## 4. Discussion

Triterpenoid saponins are crucial plant secondary metabolites with significant roles in liver protection, immune regulation, anticancer, diabetes prevention, antiviral, antioxidant, anti-inflammatory, and cardioprotective activities. The pentacyclic triterpenoid saponin α-hederin and hederacoside C are the most abundant saponin constituents in *H. helix*. Notably, the European Pharmacopoeia has designated hederacoside C as the official quality control marker for *H. helix*, making it a critical parameter for evaluating the medicinal quality of this botanical material. The investigation revealed a highly dynamic correlation between α-hederin and hederacoside C in *H. helix*. Hederacoside C exhibited a temperature-dependent bipolar response; it initially increased but then decreased further as environmental temperature rose. Conversely, α-hederin exhibited an opposite accumulation pattern; under severe stress conditions, the content of α-hederin was significantly elevated, suggesting a potential role of triterpenoid saponins in stress response [31]. The concentration of α-hederin in *H. helix* leaves showed a biphasic response to varying watering schedules, initially decreasing and then increasing. By contrast, the accumulation of hederacoside C exhibited a distinct drought-dependent pattern; it increased sharply during moderate stress, but declined progressively under severe drought, ultimately reaching minimal levels [32]. Effectively increasing the saponin in *H. helix*, especially the accumulation of hederacoside C, was a significant highlight of this research.

Hormones are crucial in managing plant growth, development, and stress responses, allowing plants to quickly adapt to environmental stress variation. Drought stress acts as an inducer for the hormone accumulation, and ABA is crucial for plants coping with abiotic stress, primarily facilitating drought adaptation through significant physiological changes. Exogenous ABA application mitigated drought-induced growth inhibition, enhanced root vitality, strengthened the antioxidant defense, upregulated the expression of PgHMGRs, and promoted ginsenoside accumulation in *P. ginseng* under drought stress [33]. Exogenous ABA inhibited the expression of JA biosynthetic genes in *B. chinense*, thereby reducing JA levels and leading to a decrease in saikosaponin biosynthesis. *BcbHLH4* expression peaked at 6 h post-ABA induction and declined to its lowest at 24 h, mirroring the saikosaponin accumulation trends of biosynthetic genes (BcHMGR and BcBAS). The triterpenoid saponin in *H. helix* leaves underwent ABA-induced accumulation, and a transcriptome analysis identified a time-dependent rise in DEGs, with 7108, 6601, and 7578 DEGs observed at 0, 6, and 12 h after ABA treatment, respectively, suggesting that the activation of complex adaptive mechanisms in plants follows hormonal stimulation. Functional enrichment and pathway analysis offered a limited understanding of the mechanisms behind ABA-induced triterpenoid saponin biosynthesis in *H. helix*. GO enrichment analysis revealed the activation of signaling pathways and the upregulation of specific metabolic processes, notably triterpenoid glycoside biosynthesis, indicating a targeted reprogramming of secondary metabolism. KEGG analysis indicated significant enrichment in the pathways of terpenoid backbones, sesquiterpenoids, and triterpenoids. Instructively, 56 genes, including HMGS, HMGR, FPS, β-AS, UGT, and CYP, exhibited differential expression, and these genes collectively explained the accumulation of triterpenoid saponins. The systematic activation, from upstream signal transduction to metabolite biosynthesis, demonstrated that ABA could regulate the operation of triterpenoid saponin biosynthesis in *H. helix*.

NAC plays a role in various developmental processes, including fiber cell development, leaf senescence, fruit maturation, and secondary metabolite production. Studies show that NAC TFs influence plant growth and development by engaging in various hormone metabolic pathways. In *O. sativa*, OsNAC29/31 regulated OsMYB61, which subsequently activated cellulose synthase genes (CESA), and the NAC-MYB-CESA module could be disrupted by gibberellin-induced SLENDER RICE1 (SLR1)-NAC29/31 [34]. *ANAC096* partnered with bZIP TFs, such as ABRE-binding factors and proteins (ABF/AREB), to stimulate ABA-responsive gene expression, enhancing plant tolerance to dehydration and osmotic stress [35]. Overexpression of the *PgNAC41-2* significantly stimulated the production of mono-saponins ginsenoside Re, Rb2, and total saponins in *P. ginseng*. *PgNAC41-2* was notably upregulated and strongly co-expressed with the biosynthetic genes, including *PgSE2-4*, *CYP716A52v2-1*, *CYP716A52v2-3*, *CYP716A47-1*, and *UGT71A27-2*, suggesting PgNAC41-2 has a close association with saponin biosynthetic genes and supports a potential regulatory role in triterpenoid saponin biosynthesis.

Transcriptome sequencing of ABA-treated *H. helix* leaves revealed that 24 and 31 *HhNAC* genes were differentially expressed at 6, and 12 h, respectively. The results indicated that some HhNAC members were induced by ABA signaling. A genome-wide analysis found that 182 HhNAC family members were randomly distributed across 24 chromosomes in *H. helix*, significantly higher than the number of NAC family members in other plants, such as 175 in *A. thaliana*, 175 in *O. sativa*, and 171 in *P. ginseng*. A synteny analysis of HhNACs revealed that 250 gene duplication events occurred in the HhNAC family, suggesting that the lineage-specific expansion of the HhNAC family might result from genomic duplication events during evolution, conferring great adaptability to *H. helix*. It was worth noting that the evolutionary relationship between NACs in *H. helix* and *A. thaliana* was closer than that with monocots. The NTL subfamily, characterized by a transmembrane domain, was essential for enhancing plant stress resistance and drought tolerance [36]. COG4, a NAC TF, was bound to promoter regions of hormone-responsive genes, activating their expression to combat cold stress [37]. The intimate phylogenetic relationship suggested that HhNACs located within the same subgroup might exert similar transcriptional regulation. A phylogenetic tree of HhNAC proteins was constructed, dividing them into 16 subgroups. Combined with their gene structure, motifs 1-5 were widely presented in HhNAC proteins, with motifs 1-3 containing the most conserved A, C, and D subdomains of the NAM domain. Similarly, *HhNAC* genes within the same branch exhibited a high degree of similarity in terms of gene structure and the distribution and numbers of conserved motifs, providing molecular evidence to further demonstrate the basic structural conservation of the NAC family.

The CREs within the promoter of HhNACs identified numerous ABA-responsive elements, indicating their possible role in ABA signal transduction. Indeed, ABA treatment for 6 h showed an upward trend in saponin content. Combined with the transcriptomic results, 24 HhNACs showed significant dynamic changes in expression under ABA treatment, with 14 genes showing upregulation at 6 h. Interestingly, *HhNAC93* exhibited a relatively high basal expression level at 0 h; its relative expression increased most significantly at 6 h, followed by a decline in expression, which perfectly aligned with the time-dependent accumulation of triterpenoid saponins. In the future, knocking out and silencing the *HhNAC93* in *H. helix* is necessary to prove its role in saponin accumulation. Simultaneously, the expression of 30 saponin biosynthetic genes displayed a synchronous change with *HhNAC93*, exhibiting a synergistic induction, which strongly indicated its regulatory role in the biosynthesis of ABA-induced triterpenoid saponins in *H. helix*.

## 5. Conclusions

This study utilized HPLC and transcriptomic analyses to examine the ABA-induced saponin accumulation in *H. helix*. Total saponin content initially increased, peaking at 6 h, before declining in *H. helix* leaves after ABA treatment. Transcriptome analysis revealed dynamic gene expression under ABA treatment, with DEGs significantly enriched in hormone signaling and phenylpropanoid metabolism pathways, identifying 182 HhNACs. Through bioinformatics analyses such as phylogenetic tree, gene structure, physicochemical properties, and collinearity analysis of the HhNACs, results showed that the HhNAC family members were evolutionarily conserved in *H. helix*. The promoter upstream of HhNACs was enriched with stress-responsive CREs, which could stimulate the expression of HhNACs, thereby helping *H. helix* leaves adapt to abominable conditions and accumulate specialized metabolites. According to comprehensive transcriptomic data, the expression profiling of 182 HhNACs at different organs or under ABA treatment was investigated. HhNACs sensibly influence saponin accumulation and stress response in *H. helix*. ABA-inducible *HhNAC93* was perfectly expressed in *H. helix* leaves, aligned with the time-dependent accumulation curve of triterpenoid saponins, and was synchronously expressed with the 56 biosynthetic genes associated with triterpenoid saponins. The findings provide a theoretical basis for further investigation into the transcriptional regulation of triterpenoid saponins in *H. helix*.

## Data Availability

The original contributions presented in this study are included in the article/Supplementary Material. Further inquiries can be directed to the corresponding authors.

## Supplementary Materials

The following supporting information can be downloaded at https://www.mdpi.com/article/10.3390/biom15111557/s1: Figure S1: Phylogenetic analysis of NAC members in *H. helix*, *A. thaliana* and *O. sativa*; Figure S2: The information of motifs 1–10 using MEME server; Figure S3: Sequence alignment of NAC domains; Table S1: Summary of RNA-seq data in *H. helix* after ABA treatment; Table S2: The protein sequences of the HhNAC proteins in *H. helix*; Table S3: Physicochemical properties of NAC proteins in *H. helix*; Table S4: The protein sequences of NAC family in *A. thaliana*, and *O. sativa*; Table S5: The protein sequences of triterpenoid saponin biosynthetic enzymes in *H. helix*; Table S6: The FPKM value of HhNACs and triterpenoid saponin biosynthetic genes; Table S7: Primers used for quantitative real-time PCR.
