# Supplementary material for: Comprehensive Identification and Abscisic Acid-Responsive Expression Profiling of NAC Transcription Factor in Triterpenoid Saponin in Hedera helix
Source: Biomolecules. 2025 Nov 6;15(11):1557. doi: 10.3390/biom15111557 (PMC12649841; doi:10.3390/biom15111557)
Supplement: Supplementary file 1 [file biomolecules-15-01557-s001.zip › biomolecules-3908702-supplementary.pdf]

Supplementary Materials

Table legends

Table S1 Summary of RNA-Seq data in *H. helix* after ABA treatment.

Table S2 The protein sequences of the HhNAC proteins in *H. helix.*

Table S3 Physicochemical properties of NAC proteins in *H. helix.*

Table S4 The protein sequences of NAC family in *A. thaliana,* and *O. sativa*.

Table S5 The protein sequences of triterpenoid saponin biosynthetic enzymes in *H. helix*.

Table S6 The FPKM value of HhNACs and triterpenoid saponin biosynthetic genes.

Table S7 Primers used for quantitative real-time PCR.

Figure legends

Figure S1 Phylogenetic analysis of NAC members in *H. helix, A. thaliana* and *O. sativa*.

Figure S2 The information of motifs 1-10 using MEME server.

Figure S3 Sequence alignment of NAC domains.

Table S1 Summary of RNA-Seq data in *H. helix* after ABA treatment.

Samples	Clean reads	Clean bases	GC Content	%≥Q30
A0-1	21,636,488	6,454,654,393	43.16%	93.40%
A0-2	22,139,773	6,607,533,840	43.80%	93.46%
A0-3	23,192,108	6,923,674,115	43.31%	93.21%
A6-1	29,964,670	8,937,951,298	44.07%	93.59%
A6-2	22,443,452	6,696,102,514	44.12%	93.23%
A6-3	21,976,793	6,555,020,837	44.08%	93.08%
A12-1	23,005,734	6,853,565,520	43.59%	93.82%
A12-2	24,464,426	7,299,790,946	43.14%	93.75%
A12-3	22,381,577	6,679,685,093	42.98%	93.16%

Table S2 The protein sequences of the HhNAC proteins in *H. helix*.

Name	Sequences
HhNAC1	MEREVKDETLPPGFRFHPTDEELITCYLINKISDASFTGRAITDVDLNKCEP<br>WDLPGKAKMGEKEWYFFSLRDRKYPTGVRTNRATNTGYWKTTGKDKEI<br>FNSVTSELIGMKKTLVFYRGRAPRGEKTNWVMHEYRIHSKSSFRTTKQDE<br>WVVCRVFQKSAGGKKYPSNNSRAVNPYNLEIGAPSAMPSQIIQSDPFHQF<br>PVGRTYMTNVELAELTRAFGGGSTSMNMPMPIQSQLNYPVVAGGGGGS<br>FTISGLNLNLGGGTSTQPFFRQPAPPPPPSMNQQDVTSSMLTGGDIGGDA<br>AAYAVDMNNANVLNSRFMNMEHCADLDNYW
HhNAC2	MTKEMEISIGSFPGFKFSPTEEELILYYLKKKIEGFDKCVELIAEIDITQFEPW<br>DLPAKSIMQSDNEWFFFSARGRKYPRGLQSRRATESGYWKATGKERNVK<br>TGSNIIGTKRTLVFHQGRAPKGERTEWIMHEYCMSGTSQDSLVVCRLRRN<br>CEFHFHDTLRKESRHSSPGDSSISTPRKESSHSSPGDSSTSALAEGLQHKGV<br>DGIYRDHSSRDNSHSVEHINFECESGNKPANEIFQDGCSSLQKGYDAEDD<br>CFADILNDDIVKLDESSLLPSTQPKIDVKFKEPVQRNMPVMLPSQGTANR<br>RIKLWREKEINSITNLEASKVGDENFSGGADRQTQPPHCLMSTLSDMLAN<br>HHSIILLLIPTLLVLFVFVGMTLASQRFWS
HhNAC3	MEEVPPGYRFFPTEEELIAFYLHNKLEGNDSQALARVIPVVNIYEQEPWQ<br>LPNNLIQEREARGGRPNRTTASGFWKATGSPSYVYSSSNKVIGMKKAMV<br>YYHGKAPTGKKTKWKMNEYRAIEEEVLTSTSSSTSAAPRLRHEMCLCRV<br>YVISGSFRAFDRRPLGTVIREASSHQADGNKDTATSSQNFPNIN
HhNAC4	MEIEQNSSSHYPPGFRFHPSDEELIVHYLLKKLGSRPLPAAVVAEIELYNY<br>NPWDLPDKSLFGEDEWYFFTPRERKYPNGERPSRTAGLGYWKATGTDKP<br>ILSASGSRSIGVKKALVFYKGRPPKGLKTDWIMTEYRLPDTMTRSARSKGS<br>MRLDDWVLCRIRKKGNMSKNNWAGDESPSKSVNFLPKIEELPSTYTKTN<br>TEITTDYPLKDCHILASMQAYQDLPPIENISNSTFPQRNYSNNSDSGFYDH<br>TSGKGNSFVTSSFFESFPNPTKAKLNEGNRFDTLVGSNEMVTERNGNEVL<br>IPGNMMTTTAMNFYKQNQAQGNIFNPSQLDAIVNLLLSREDF
HhNAC5	MIEKKIMKNLSFISNIGAALPPGFRFYPSDEELVCHYLYKKIANEEVLKGTL<br>VEIDLHTCEPWQLPEVAKLNSDEWYFFSFRDRKYATGYRTNRATKSGYW<br>KATGKDRTVVDRRTNGIVGMRKTLVFYKNRAPNGIKSGWIMHEFRLETP<br>HLPPKEDWVLCRVFHKAKMTTENNNQLSPRDVFEAEAIAGDTSPIPHQI<br>LPLGHQHHQSPAPSQYQNPNNLLELSTLNPNFLQLSQVHTCVNELMMI<br>NSKCQEDEYGYLFDMSFEESNLGSDGIMATNMEDMRFEDDDHSLVFL
HhNAC6	MAVLPLGTFPVGYRFRPTDEELINYYLKLKIKGFEKEVTVIREVDVCKCEP<br>WDLPDLSMIESNDNEWFFFCPKDRKYQNGQRSNRATEAGYWKATGKD<br>RFIRSSKGRNVIGRKKTLVFHTGRAPSGKGTQWVIHEYWATDEALNGTH<br>PGQTAFVLCRLFKKHDGKLDGMAESSNCDKVEQNVSSTVVKSYAEDKSE<br>PETPMLSGQPQMQSASTEGCADGNYIKTIHDYSLTMDQYSNSCTADEAE<br>GQVLDPIQPDPYLEYELRAFYDSPQPPDSKIFSPLHSQMISEFGSSYLHSPTT<br>FSNDNKEVQFQYGTNSMDIAELLNSIIVSSDEDSATHHISGVESESCSKSDV<br>EVTEGQRPACFYEAQFDPSKMARHPGHMPQEQGYIPNFINNEAQRNLRS<br>LPNWPYVSSAVSVSDQVYNLGSPEGSFTSCNAVGNDDNIGTGIKIRTRQR<br>NVPATFQGGLVTQGTAPKRIRFQTKLQVGSVSCSKPKDLSYGEENCEIKPL<br>VIEAEEATEKHNSTTATDAASTTDETEEVSLVKPYRSGEVAQEPSLNVKTK<br>GGFPFGGRIKVLSEFLKAPSVLRSISSSLRIPGTLLTGGLVIIVVGICGHLLF
HhNAC7	MFQLTTMLALEEILCELNGQVEMNNEQGLPPGFRFHPTDEELITFYLASK<br>VFNPTFSAGVNIAEVDLNRCEPWELPDVAKMGEREWYFFSLRDRKYPTG<br>LRTNRATGAGYWKATGKDREVYSTSSSNGGAGSASNGGQLLGMKKTLV

	FYKGRAPRGEKTKWVMHEYRLDGDFSCRHTCKEEWVICRIFHKMGEKK<br>NGVALQGQSSHYLQEASITSSSPSKTGLLELQPHKPIQSLQNQNPNPFLIN<br>DYHHENDLKSLITINNISSSIGDAAVSQAFPINPINGGYQTSFSPTRNNKEK<br>EDTNNKQSPSSFFKSLLTNQQDYYYYYNYLTSFKEQQIAPPISNIIPKHCKT<br>EPNISHVDHFSSNDHNAAILRCPNPPDYNNNYKIQQQNPTSSSYYQSPFL<br>FGTMDTNGGFPSAAAGADANLHDMSTSSSSTSGVLAFNRAGFHQMLLD<br>SSIKLAAGESWPFHF
HhNAC8	MDEKMIMSIWDKDNNYKEDDDEEEEEVLVLPGFRFHPTDEELIGFYLRRK<br>VEKKPLSIELIKQVDIYKYDPWDLPKVGMVGEKEWYFFCMRGRKYRNSV<br>RPNRVTESGFWKATGIDKPIFATSTTHDQETHACIGLKKSLVYYIGSAGKG<br>TKTEWMMHEFRLPPTNWKNKNPQINAKNKDITLEAEVWTLCRILKRNA<br>SFRKYTPPYRKQDTSEQITKDGSPIRFSLESDENMNSRNLLFQQKREKLDIN<br>HVEERNKKIRNQQNSTAQTPFTVSQLSYGNPNKDDFGGGNWDELRTVV<br>ELAVDPFSLNQFR
HhNAC9	MEGTPVVVNEGDQELLNLPPGFRFHPTDEEIITHYLTQKVVDDKFSATAI<br>GEVDLNKCEPWDLPKKAKMGEKEWYFFCQRDRKYPTGTRTNRATESGY<br>WKATGKDKEIYSKEKGNQQLVGMKKTLVFYKGRAPKGEKTNWVMHEF<br>RLDGKFSYYNFPKTAKDEWVVCKVFHKNTGMIKRSNTMTELMRMNSFV<br>EGLLDSPLPPLMDSPYSNNINGIISNEDDFKGKVITATSAKSSGGNSFSYLP<br>KGQIQMQQQQQNNYMASFNPPNYQTSTPSYQIPNSIFYSQNGIPIPNANF<br>LYQCSSTPPGYLHQQRINNSISSFSGSPNFEQASANQDPERQLKVEQFSSN<br>QSMVSRSQDTGLSTDLTAEISSKQEVNRSKMSYGDIEGRPNSVAPLSDLDS<br>FWNF
HhNAC10	MTRELGGNGQVVSVPPGFRFHPTDEELLYYYLRKKVSYEAIDLDVIREVD<br>LNKLEPWDLKEKCRIGSGPQSEWYFFSHKDKKYPTGTRTNRATAAGFWK<br>ATGRDKVIHLNNNSKRIGMRKTLVFYTGRAPHGQKTDWIMHEYRLDDP<br>YNAEVQEEGWVVCRVFKKKTHTRTYEPDIHSQEHHHYLGGTTISSLIDPK<br>QNHQAIYDHQNGTIFDGSMQLPQLFSTESSPSTVHHHHHQPPFLCPGPA<br>MNNLIDMECSQNFLRLTSDGLGLAPVPERFMADWSFLDKLLTGHHTICN<br>PSSIALDLG
HhNAC11	MDVEKKSSIDNCGKQHEDDDVPLPGFRFHPTDEELVGFYLRRKVEKRTIS<br>LELIKHIDIYKYDPWDLPKGSNVGEKEWYFFCRRGRKYRNSIRPNRVTGS<br>GFWKATGIDKPVYSEGRRDSTVIGLKKSLVYYRGSAGKGTKTDWMMHEF<br>RLPPPEHHPGKNTTKHILLVNDGFQEAEVWTLCRILKRNTSNRKCIPDW<br>RENPTTKIRNNPVIDASSKTCSVESDNRQSYNISFRTTPITKYLHDEKKVLF<br>SNDLHVNIGSNNLVMGPGMGSIYQAPSRFVTSYSNFSSPEVNELTQYGN<br>WDDLRSVVQFAGDTIF
HhNAC12	MSNLKMVEAKLPAGFRFHPRDEELVCDYLTKKITRSEQPPILMEVDLNKS<br>EPWDIPEYACVGGKEWYFYSQRDRKYATGLRTNRATDSGYWKATGKDR<br>PVSHRNSLVGMRKTLVFYQGRAPKGKKTDWVMHEFRLEGPLGPPKLSSS<br>KEEDWVLCRVFYKNKQVDHNHSPGSKQGMAGNRYEDTISLSSTYLPPL<br>MDDPPPPPPLYNEYQIHDDYHQSSLCEQVPCFSIFNPMNIISSKFITTTTNS<br>SVPNNTFAGGGMAPDQLINGNTSCEDKMVIDAVLNHLMDNINVNNSD<br>QHHPTNNMVKMMMKDSPCSFGEAASTSESFLSEVETIWNNY
HhNAC13	MKSEQIELPPGFRFHPTDEELVMHYLCRKCASQEIAVPIIAEIDLYKFDPW<br>QLPGMALYGEKEWYFFSPRDRKYPNGSRPNRAAGTGYWKATGADKPIG<br>KPKTVGIKKALVFYAGKAPRGVKTNWIMHEYRLANVDRSAGKRNNNLR<br>LDDWVLCRIYNKKGPLEKHDTKLIKYPEMDETEERKPEFATFDQVQSVPQ<br>PPQQSAVPLQMMKDDYSAFDTSESGARLHTDSSCSEHELSPEFIYERERER

	EVQSEPKWNELENVLSSSLDFPFYMDDFPDDPFAPQVQYTDHQLSPLQDI<br>FMFMQKPY
HhNAC14	MTVPESSCFGGAKFFPPGFRFHPTDEELILYYLKRKICRRRLKFDIIVETDVY<br>KWDPEDLPGLSKLKTGDRQWFFFSPRDRKYPNGARSNRATMHGYWKTT<br>GKDRTISCNSRSVGVKKTLVYYKGRAPSGERTDWVMHEYTLDEEELKRC<br>LSAKDYYALYKVYKKSGPGPKNGEQYGAPFVEEDWADDECVNFNADQ<br>MNSVKEVDISCVDNNRANDQPLCLVNDLEELLNHITEMPVLASPLDVDY<br>THALDQLIGVEENASALVDHSSRETNLPEGSMVLHQSQQYNVQASFDYT<br>HSATSQLQLYEASEVTSAPNICEQKLGENFLEDFLEMDDLISSEPTVQNFR<br>EQPEPLSFDEIDGLIDLLYNDENMFLCDMVPGEQGQISQPYANNPESGMV<br>NVAPNSYLNNENGEVNHQKEPYRNNANLMSSQLWIEDEGCYIFNPAEA<br>NQAPVSQQISGVECNDNNGNVSTGATQNQSGKVDTDSWFSSALWAFVE<br>SIPTTTASASESTLVNRTFERMSSFGRVRINTGKTNDAAGNLGATSKGSGN<br>FKGGMLCFSVLGIFCAILWMLIATSIKLLGKYVSP
HhNAC15	MGDPKNPNPPSFSLLPPGCRFFPSDEQLLCYYLSLKNKGDRSSGFDVIKEIE<br>FYNFEPFDLPDSVCFRFGRGGRKMHYYCYVARVLREEGRRRAGSGYWKR<br>SGRVRNVLGGSAGKVVVGTRRSFVFYLGDSPTNAVRTDWIMYEYAPTDH<br>HAASFVLCRVFIKSHRNNRSEHPLSSVGEESAATVRHIGIQHDGTVRQVIG<br>EANTLDDNLPDHKNEVLRFPVGLVGDLDDKVTNGPVGLQMRSPGLSIG<br>RDSIVDSLTAEQLVALLEEDFLELDDLVSPLPGID
HhNAC16	MCPPAPASPISAVVLTDEELILLLEDIIHGYPSYPLPNNVIADLNPFNYPPS<br>NLPDGIVYFLRAEVKKNTKLGSWKAKGEPCEIFTNSVITGWRTTLEFHEG<br>QAPNDQKTDWVMQEYRITQKGLCNNRNTKDSGSLYRLFRSEECIPDHVV<br>HPKHGTALLTDSTTGQGLTEAQKKNEDDEIGSLTAAERLNCLLQGDFLE<br>LDDLNGLQPSSSSSENSSRRSFASDEWFDSEALLRNLDHKNIEQQGRDASF<br>KYTVSTSIIPNEVVICPATLGKLSAEDTRKLDYSLPIEGTIDKRVLKCKQVN<br>ETASSSQNAAASSSHNKAVKEKKKKDVSRMKKLKKKYFCLMPF
HhNAC17	MPFSKMVELVDANFPPGYRFVPTDEELVKYYLQNLVLVWNIKCTHHSG<br>MYMEHCENVWYFFTPRNRKYPNGRRPDRAAGTGYWKATTVEKEVLKS<br>GPDDQELPNKNRVKIILGFKRGLDYYEGKHPEGKKTDWKMNEYRFNSTS<br>NLPITNNGENDKEVRSIRPLFFIAKKSTEEEVAGGLGGPGHDQSGPPASID<br>FSSNQNNSNSTVNINAVPSNSNGENIKRVRLFYFISFLSWIQVK
HhNAC18	MDYPPPGFRFYPTEEELVSFYLQNKLLDGSRQDLDRVIPVLHIYDFSPWDL<br>PKYAGEFCQGDPEQWFFFIPRQDKETRGGRPNRLTSSGYWKATGSPGLV<br>YSSYDNRVIGMKRTMVFYEGRAPNGTKTEWKMNEYKAASTINDHQPPN<br>SSSSSSSTSTTSSTLQLRQELSLCRVYKRSKCLRSFDRRPSSGVVTIGQGGTSV<br>EAGTTSVSADQNQNQNQNMMMMMRSSSAADDHDHSSSSGDYSNNNI<br>VPSHRLDTDDQPMWEWDWEPMFS
HhNAC19	MDYPPPGFRFYPTEEELVSFYLQNKLLDGSRQDLDKVIPVLHIYDFSPWDL<br>PKYAGEFCQGDPEQWFFFIPRQDKEARGGRPNRLTSSGHWKATGSPGFV<br>YSSHDNQVIGMKRTMVFYKGRAPNGAKTEWKMNEYKAAATTNDHHQ<br>PPSSSSSTSTTSSTLQLRQELSLCRVYKRSKCLRSFDRRPSGVLTIGQGGAVE<br>AGTTSVSADYHQNQNMTMMRSSSPADDHDHSSSSGDHSNNNNNNNTDP<br>SNHLDTYDLPMLDWDWEPMFS
HhNAC20	MQQNPTDDGVAAAQSYFDMIPPGYRFKPTDGELVFYYLQKKINNEPLPL<br>NQINEYNIYLKNPQELAEEYKSSNEELYFFTPRQRKYKNGGRPNRAAGD<br>GYWKATGADKPITHNGEEVGFRKALVFYMGKPPTCTKTCWIILNEESYIF<br>SHEMASGYNGNDEASIGYFIPFDNHHGFADHFQNEFTGTPQHDIAHFNP<br>NQDMVPFGFDYSNQDMVPSVNYLNQPMAPFDHFDYSNHMQQMIEPSS

	SLIDQLQPFSTQNDIEPNKINNHYPCHSSVNPEGHDQEEVREETKEVLGH<br>FSAQSYINTDQSHINECSKIPSPDPDSTTD
HhNAC21	MHICLLASGSTQLMKSSSLITSSRKFLTTTSAAGPLLKLTSTNANRGSFLVR<br>NLDNNLDVVILNYSMLFGYDLTEKAKMGEKEWYFFSLRDRKYPTGLRTN<br>RATEAGYWKATGKDREIYSSKTGCLVGMKKTLVFYRGRAPKGEKSNWV<br>MHEYRLEGKFAYHHLSRTSKDEWVISRVFQKIGGGATLAAGGGKKRLTS<br>SINFYPDISSPSSVSLPPLLDSSPYATATSGGGATTDHESCSCDANFTTKEH<br>VPCFSTPAAPSCFNHHSVFDILPPAFTSAMVDPSSSSHFQSNIGVSAFPSLR<br>SLQENLQLPFFFSAVAPTPVHGGGEMGNNYSSAAENMPAPETQKAGPTE<br>LDCIWRF
HhNAC22	MGVPETDPLSQLSLPPGFRFYPTDEELLVQYLCRKVAGHHFSLQIIGDIDL<br>YKFDPWVLPSKAMFGEKEWYFFSPRDRKYPNGSRPNRVAGSGYWKATG<br>TDKIITTEGRKVGIKKALVFYIGKAPKGTKTNWIMHEYRLFEPQRKSGSAR<br>LDDWVLCRIYKKNSSAQKPVMGISSSKEHSHSHGSSSSSSSQFDDVLESLPE<br>IDDRFFTLPRMNSLNLGSGNFDWASLAGLNSMPEQFPGGGQAPMQTQA<br>QGVIMQNNNNDQNDVYAPARSPLGHADTRFGRNIEEEVESGVRNQRV<br>DNSGFLSSNNYAQGFTNSIDPFAIRYPTQPGNLMFRP
HhNAC23	MESTDSSMGSQQQPQLPPGFRFHPTDEELVVHYLKKKATSAPLPVAIIAE<br>VDLYKFDPWELPAKASFGEQEWYFFSPRDRKYPNGARPNRAATSGYWK<br>ATGTDKPVLTSGGSQKVGVKKALVFYGGKPPKGIKTNWIMHEYRLDDK<br>KSSMKPPGCDVANKKSSLRLDDWVLCRIYKKNNTQRAMDHESSDSMND<br>NMISPSIHPKASTTNYGTTTTSSIILENEQNMFQGMEYNADGSISLYGTSKS<br>QPSVPCSSNFLSVKPNLQTSTLYWNDEAAIGRGSSSAGSTKRFFADGGRTE<br>DQNSSIASLLSQLPQNAQTLHHQQPAMLENISDGVFRQPPYQQLPGGMN<br>WYS
HhNAC24	MAVLPLETLPVGFRFRPTDEELINHYLKLKINGFEKEVSVIREVDVCKCEP<br>WDLPDLSMVKSIDNEWFFFCPKDRKYQNGQRANRATRAGYWKATGKD<br>RSIKSSKGRNVIGRKKTLVFYTGRAPKGERTRWVIHEYCATDEALNGTHP<br>GQSAFVLCRLFKKHDGKQDEMAESSNCGEIEQNVSSPTVVKSYAEDTQSE<br>LETPMLSGLPQIQSVSTEGCADENAKKEILRYSLTMDQYSNGCTADEAEG<br>QVIYSIQPDPYLEDALRGFYDPPQPPDSKIFSPLRSRMMDELRSSYFDSPTTF<br>SNDEVQYESGTNAMDIAGFLDSILASSDEDSATHHISGVESGTPKYINTIEF<br>ANKDSGSCSESDVKVGQAQLYPGTFLEALNTTSLLQMGHAVSQGECINP<br>NFRKEAGAVFQGEWITPNFRKDAPAVFQGECITPNFSNDEAQRNLRFLPI<br>RPDVLSAVSIGEQDNNLLSLEESYPGSSAVGSRNNIGTGIKIRTRQPHSQAS<br>AQNIVMQGTAPKRIRLQTKLKVGSVSCSKPKDLSYGEENCEVKPLVIEAE<br>EATEKHNFATATDAASTTDETEEVFLLKPYCNGEVAQEPSLNVKTRLLLC<br>SISSSLRIPRTLLTVGLVIVVVGICKCILF
HhNAC25	MAIAFTMNLQENHDHENKNNNIDEHEHDMVMPGFRFHPTEEELVEFYL<br>RRKVEGKRFNVELITFLDLYRYDPWELPALAAIGEKEWFFYVPRDRKYRN<br>GDRPNRVTTSGYWKATGADRMIRTENFRSIGLKKTLVFYSGKAPKGIRSS<br>WIMNEYRLPHHETERLQKAEISLCRVYKRAGVEDHPSLPRSLPTRATTSRS<br>NQKHQDVTQHAIEKFQTFGELQTQQINDKKISETSGNSTTDDVGTALGLS<br>NHNIYIPLAPINALLSLQNCNTIYNTASSSLVTTTNPNNSIDDLHRLISYQQ<br>DASVNSQQQFYHNNSHINVQPVQHSQSLTLSMLPGSVQAAFSDRLWEW<br>NAITEAAAITKDYGSAFK
HhNAC26	MLALEDILCELNGELEMNNEQGMPPGFRFHPTDEELITFYLASKVFNPTF<br>SAGVDIAEVDLNRCEPWELPDVAKMGEREWYFFSLRDRKYPTGLRTNRA<br>TGTGYWKATGKDREVYSSSNGGAGSASASKGGQLLGMKKTLVFYKGRA

	PRGEKTKWVMHEYRLDGDFSCRHTCKEEWVICRIFHKMGEKKIAVVLQGQSSHYMQEASITSSSPSKTGLLELQPHKPIQSLQNQNPFLINDHHHENDLKSLIPINNNSSSVGDAAVSQAFPINPINGGYQTSFSPTRTNKEKEDTDKKQSPSSLFKSLLSNQQDYYYYYNCPTSFKEQQTAPPTSTIIPKHCKTETNFSHFDRFTSNDHNDAILRCPIPPDYSNNYKIQQQNPTSSSYYQSPFLFGTMDTNGGFPSAAAAGADANLHEMSTSSSSTSGVLAFDRAGFHQLLLDSSIKLAVGESWPFHF
HhNAC27	MEGAPIVVDEGDQEILNLPPGFRFHPTDEEIITHYVTQKVVNKKFSATAVGEVDLNKCEPWDLPKKAKMGEKEWYFFCQRDRKYPTGMRTNRATESGYWKATGKDKEIYSKGKGNRQLVGMKKTLVFYKGRAPKGEKTNWVMHEFRLDGKFSYHNFPKSAKDEWVVCKVFHKNTGMIERSNTMTELMRMNSFVEGLLDIPSLLPPMDSPYFNNTNGIISNEDDFKGTIITSTTSAKSSGGNSFSYFPKDQIQMQPQQNNYMTSFNPTNYQTSTPSYQIPNSIFYSQNGIPIPNANFPNQGSSSPLGYLHQQRINNSISSSSGSPNFEQASANQALQRQLKVEQFSSNQSMVSRSQDTGLSTDLTAEISSKQEADRRTISYGDIGGRLNSVAPLSDLDSFWNY
HhNAC28	MMKELGGNGQVVSSVPPGFRFHPTDEELLYYYLRKKVSYEAIDLDVIREVDLNKLEPWDLKEKCRIGSGPQNDWYFFSHKDKKYPTGTRTNRATAAGFWKATGRDKVIHLSNNSKRIGMRKTLVFYTGRAPHGQKTDWIMHEYRLDDLDNAEAQEEGWVVCRVFKKKTHTRSYEPDIHSQEEHHHLGATTVSSLSEPKQNHQSIYDHQNGTIFDGSMQLPQLFSTELLPSTVHHHQQQQQQHQHQPPFLCPGSAMNNFTEMECSQNLLRLTSGGPGLAPVPERFTADWSFLDKLLTDHHTRCNPSSQALDL
HhNAC29	MAPMALPPGFRFHPTDEELVAYYLDRKISGHTIELEIIPEVDLYKCEPWDLPDKSYLPSKDMEWYFYSPRDRKYPNGSRTNRATRAGYWKATGKDRLVQSQKRPVGMKKTLVYYRGRAPHGIRTNWVMHEYRLTESLYGTTTAATLKDSYALCRVFKKVIHIPKSSSKEAEQMNEVSENNKSVYESDEHLLFTEEETGGLTGISAELGDDHDRDQDYSKFPSDASSNSDVTQGTPLEAAGTTVTDCLQAAFASDEANSSANMYSSYTLNCPSSNIFQDIHTPNYSSMQYEDACYPPLALEDFPQIDIAAETTSSKPSNPADQALDDHLMYNVYASG
HhNAC30	MDVEKKTSIDSCGKQREDDDVPLPGFRFHPTDEELVGFYLRRKVEKRTISLELIKHIDIYKYDPWDLPKGSNVGEKEWYFFCRRGRKYRNSVRPNRVTSSGFWKATGIDKPIYSEGRRDSTLIGLKKSLVYYRGSAGKGTKTDWMMHEFRLPQPAHHLDKNTTKHIDLMNDGFQEAEVWTLCRILKRNTSNRRCLRDWREISTTKIRNNPVLDASSKTCSVESDNRQGYNISFRTTPITKHVQDETKPVLFSNDLLHVNIGSNNLVMGPGMGSVYQAPSSVVTSYSNFSSPEVNELAQYGNWDELGSIVEFAGDTYF
HhNAC31	MSNLKMVEAKLPAGFRFHPKDEELVCDYLTKKITRSEQPPILMEIDLNKSEPWDIPEYACVGGKEWYFYSQRDRKYATGLRTNRATVSGYWKATGKDRPVSRRNSLVGMRKTLVFYQGRAPKGKKTDWVMHEFRLEGPLGPPKLSSSKEDWVLCRVFYKNKEVDHHHSPGSKQGMAGNRYEDTISSSTYLPPLMDDPRPPPLPPLYNEYQTYHYQSLYEQVPCFSIFNPMNIISSKFITTTNSSAPNNTFGGGMAPDQLINGNTSCEDKMVVDAVLNHLTKMDNININNNNNHHHPTNNMVMKKMMMNDSPCSFGEAASTSESFLSEVETIWNNY
HhNAC32	MKGEQIELPHGFRFHPTDEELVMHYLCRKCASQQIGVPIIAAIDLYKFDPWQLPGMALYGEKEWYFFSPRDRKYPNGSRPNRAAGTGYWKATGADKPIGNPKTVGIKKALVFYAGKAPRGVKTNWIMHEYRLANVDRSACKRNNNLRLDDWVLCRIYNKKGTLEKHDTKLIKYPEMDEMEERKPEFATFDQTQTVQSVPQPRQQSAVSLQMMKDDSGFDTSESGARLQTDSSSDHVLSPEFTY

	ERERERESEPKWNELENFLSISLGFPFYMDDFPDDPFAPQDQYTDHQFMQKPC
HhNAC33	MGDPKNPNLPSFSLLPPGCRFFPSDEQLLCYYLTLKNKGDHSFGFDVFKEIEFYNFEPFDLPDSVCFRFGQGGRKRHYYCYVARVLREGGRRRAGSGYWKTSGRVRDVLGGGAEKVVVGTRRSFVFYLEESPKNAVRTDWIMYEYAPTDHHAGSFVLCRIFIKSRRNNRSEHPLSSVGEESAATVRHIGVQHDGTVRQVIDEVNMLNNNFPEHKNEVLRFPMGVVGDLDDKVTSGPVGLQMRSSGLSVGRDLIVDSLTAEQLVALLEEDFLELDDLVSPLPGID
HhNAC34	MCSPAPASLIPIVVLTDVELILLLEEIIRRYPSYPLPNNVIADLNPFNYPPSNLPDGIVYFLRSEVKKDTKLGSWKAKGEPCEIFTNSVITGWRTTLEFHEGQAPNDQKTDWAMQEYRITKKGLCNNRNTKDSGSLYRLFHIVDCIPDHMVPPKHVGALPTDSTTGQGSTSEAQNEDDEIGSLTAAERLSCVLQGDFLELDDLNGLQSSSSSSDNSSCPSFASDEWFDSLALLRDLDHKNIKQQGKDASFKYTVSTSVISNEVVICPATLGKLSAEDIQKLDYSLPIEGTIDKRVLKCKPGNETASSSQNAAASSSHTKAVKEKKKDVGGMKKLKKYFCLVPF
HhNAC35	MDYPPPGFRFYPTEEELVSFYLQNMLLDESRQDLDRVIPLLHIYDFSPWDLPRYAGEFCRGDPEQWFFFIPRQDKEARGGRPNRLTSSGYWKATGSPGFVYSSHDNRVIGMKRTMVFYEGRAPNGTKTKWKMNEYKAVATINDHQPPNSSSSSSTSTTSTTLQFRQELSLCRVYKRSKCLRSFDRRPSGVVTIGEGGTSVEAGTTSVTADQNQNQNQNMMMMMMRSSSPADDHDHSSYSGDHSNNNTDPSNHLDTDDLPMWEWDWEPMFS
HhNAC36	MEDQNIQENFTDDGVAATQSYFDMIPPGYRFKPTDQELIVHYLQRKINNERLPLNQINECNIYLKNPQELAEEYKSSNEKELYFFTPRQKKYKNGGRPNRAAGDGYWKATGADKPIKHNGEEVGFRKALVFYLGKPPDGNKTSWLMHEYRVKNPPAFQRTEKDDFQNQVLDNNAQTLNSAIQPASMPSNQIVPSGYNDNALLNEESYIFSHRMASGYNGNDETSMGDFIPLDNHHGFADHFQNEFTGAPQYDIAHFIPNQAMVPIGFDYSNQAMGPFDHFDYSNHTQQQMIGPSPSSIDQLQIGPSPSSIDQLQIRPSPSSIDQLQQPYSTQNDVNPNKINNHYPYH
HhNAC37	MDIFYHHFDNSTDAHLPPGFRFHPTDEELITYYLLKKVLDHNFSGRAIAEVDLNKCEPWELPEKAKMGEKEWYFFSLRDRKYPTGLRTNRATEAGYWKATGKDREIYSSKTNSLVGMKKTLVFYRGRAPKGEKSNWVMHEYRLEGKFAYHHLSRTSKDEWVISRVFQKSGAGATSTAGGGKKRLTSGINLYPDISPPSSVSLPPLIDSSPYATATYVGGATTNHESCSYDGNFTTKEHVPCFSTPAAPSCFNHRSLLDGLPPAFTSSMVDPSSSSRFQSNIGVSAFPSLRSLQENLQLPFFSAVDPPLVHGGGEMGNSYPSAAGNWPVPETQKAGPAELDCIWSF
HhNAC38	MGVPETDPLSELSLPPGFRFYPTDEELLVQYLCRKVAGHHFSLQIIGDIDLYKFDPWVLPSKAMFGEKEWYFFSPRDRKYPNGSRPNRVAGSGYWKATGTDKIITTEGRKVGIKKALVFYIGKAPKGTKTNWIMHEYRLFEPQRKNGSARLDEWVLCRIYKKNSSAQKPVMGISSSKEHSHSDSRGSSSSSSCQFDDVMESLPEIDDRFFTLPGMTSVNDKLNLGSGNFDWASLAGLNPIPEQFPGGGQFPMQTQAQGVIMQNNNNNQNDVYAPTMSPLGHVDTRFGRNIEEEVESGLRNQRVDNSGFLNSNNYGRGFTNSIDPFAIRYPTQPGNLGFRQ
HhNAC39	MESTDSSTGSQQQPHLPPGFRFHPTDEELVVHYLKKKAASFDPWELPGKYICVCVHVFIFLFCDLFNWITKASFGEQEWYFFSPRDRKYPNGARPNRAATSGYWKATGTDKPVLTSGGSQKVGVKKALVFYGGKPPKGIKTNWIMHEYRLADNKKSSMKPPGCDVANKKGSLRLDDWVLCRIYKKNNTQRAMDHESSDLMNDNMILPSIHPKASTTNYGTTTSSMLENEQNMFQGMECNTDGSISLCGPSKSQLSLPCSSNFLSIKPNLQTSTLYWNDEGATGRGSSAAASTKRF

	YTDGGRTDDENGSIASLLSQLPQTPQTLHHQQAAMLGNIGDGVFRQQPYQQLPGGMNWYS
HhNAC40	MEKDYKNLPPGFRFHPTDEELITYYLGHKVLDFNFTSRVITDVDLNKSEPWDLPAKASMGEKEWYFFSLRDRKYPTGMRTNRATEAGYWKTTGKDKEIFRGGVLVGMKKTLVFYLGRAPKGEKTNWVMHEYRLENKQAFKPTKARLAWFLNFLRERRKKKTIFQKTTVKKPQQTTSSQHSLDSPCDTNTMISEFGDFDQLQNLNNAANSSNEISNISLQNLNNLVANSSSRISNISLPNLNNVANSSSGISNISLQNYTPDNNLNTSINSWPSCLPSTSLLMNSLLLKALQLTSNGFDYASFMPQGMLPYGIDFTSNFAAASSISKALDNSVQQHQQQHQEQPFNLDSMW
HhNAC41	MAPIGLPPGFRFHPTDEELVNYYLKRKIHGLDIELDIIPEVDLYKCEPWELAGQFSFIFNVSYSISVLLCLYLIDLTTKSRNFDTDIFRDPEWYFFGPRDRKYPNGFRTNRATRAGYWKSTGKDRKVSSQNRAIGMKKTLVYYRGRAPQGIRTDWVMHEYRLDDKECDQDTSGIQDSYALCRVFKKNGICGSELEDQGQPSIPLIEYSQGVIINEYETMSPADVTLASSSCMDEEDKDDSWMQFITDDAWCSSNNPIGGEEGSQVTITN
HhNAC42	MANINNNGNGNGIQVPIGYRFRPTDEELLLHYLKPKVHSLPLPASVIPDHFHLFRSPPSLLPGDSKEKRYFFCKRNWNNVQKCRITTISDGSGYWKAIGKDKAINVTHAAVGTKKSFVFYQGTRPRALKTRWLMFEYRLVPSQATTKSTQELEDWVVCCIYQRKRKTKNHGNSNKTRRNMEEVTVPSCDGMMDFMILDSSDLFGPPPPSPSYSSGNSTFSNNELLDQEEEECSSNSFGTYFSCF
HhNAC43	MAPVSLPPGFRFHPTDEELVAYYLKRKINGRKIELEVIPEVDLYKCEPWDLPGKSLLPSKDLEWYFFSPRDRKYPNGSRTNRATRAGYWKATGKDRKVNSQMRAVGMKKTLVYYRGRAPHGSRTDWVMHEYRLDERECETALGLQDAYALCRIFKKSLNEPKITNHYGITASDHSSSNIEIYSCEDNMENSDHPMPFSTYPLDTTSIMHGSPHNMGGTSTNDAKWTQYLSEEAFSFTNPSFPDCNAIAYPPSKVDIALECARLQHRLSLPPLQVQDFPQAGFVDLKMPQTSNSMYGNTGDQQDILQEIKSVAQVSQELMTQNSCIGNNSSEDDFSFLPHGNQIQGMSSTVSVEIGENMDEQFKTEKAVENLRWVGMSNKDLEKSFPEDYKTVPIENISSFQREEHEVQGNHHNKFHEFNEKEGNEHFSLGFANDDPNDQSFLDDGDLDDFSSTQSFEVYEKFEVNHGLLVSTRQVPNTFFHQIVPSETVKVHLNPVIDHNFPISKLDLPTMPENTNLFDKFKAFATTKLVGNTKSLKPWRKIETDGIKVVKRRRENGCHSLEKIESTTVKWDYEEKKVGFVGMTEWGSVVLIKMWPCLTLALASYFYHLGAAHFT
HhNAC44	MTAVEVQLPPGFRFHPTDDELVMHYLCRKCASQQIAVPIIKEIDLYKHDPWDLPELALYGEKEWYFFSPRDRKYPNGSRPNRAAGSGYWKATGADKPIGNPKAVGIKKALVFYAGKAPRGEKTNWIMHEYRLADVDRSARKKNNSLRLDDWVLCRIYNKKGTLEKHNVGSRKVSNPTGIEEKKPEIITPITENSPATFGDMMYLDTSDSVPKLHTDSSCSEHVVSPEFTWDREVQSEPKLEEWENTTLDFPLNYMDAPVDNNVNLIYGSPPQMQGNFQTPHWQDMFMYLPR
HhNAC45	NATLPFANQIRDCGANRECPKCHHRIDNSDVSHAWPGLPAGVKFDPSDAEILEHLAAKCRIANCVPHMLIDEFIPTLDGKNGICYTHPENLPGAKEDGSNVYFFHRTINAYATGQRKRRKIQTEDSLTKEHVRWHKTGKTRPVMENGTQKGCKKIMVLYRSSVKGSKPGKSNWVMYQYHLGTEEDEKDGQYVVSKIFYQQHKQADKSNNFFAITEHDKGTMCTSPKMPKTNTPNPPRSRKSDACDDVMDDYILESPVQDISVPSSSQAHFKDEIEYPFLAGESQAIDLDSTEALLLCNEILDSYVPLDNSGLYHGPSPACVRDINNAPSAERSESCGIAELENIELETPPDFQLSNLQFSSQDGIFNYLDLLL

HhNAC46	MDGNNETDKSDDVMLPGFRFHPTDEELVGFYLKRKIQQRPLPIELIKQVD IYKYDPWDLPNLASTGEKEWYFYCPRDRKYKNSARPNRVTGVGFWKAT GTDRPIYSSHGTKCIGLKKSLVFYRGRAAKGLKTDWMMHEFRLPYIPDAG TTKKFLDKNFLPHDSWAICRIFKKANSMAQRALSHSWVSPLPETTTSDIFT QGLHSTQFSSENMSCMTETSSVIQLCNELQQNSSASFSAQDIPSYRPINPTF YKPFSLGVPNGDLHNSLVFSPPDFSGPSTKSTVDISNMLFNLSPALIGDAN KTTDCITPQQEFNNFSMSSPEEMQGSIGTEEVDAGSTKNHSATHGNNQW GNIQPTGFPFSLPSSLPDAWKPNLEWDSPPCPSEVSPTYSTNKCYT
HhNAC47	MENLCDEQIELPPGFRFHPTDEELITHYLSPKVYNSSFSATAIGEVDLNKV EPWDLPWRAKMGEKEWYFFCVRDRKYPTGLRTNRATEAGYWKATGKD KEIYRAKKLTGMKKTLVFYKGRAPRGEKTNWVMHEFRLEGKFSVNNLP KTLKNEWVISRIFHKSSGGKKIHISGLVRNGNLGDELQPSNHPPLLDISITT VAATPHVTCFSENLEDRKPLDEIVLSSSILSPKLPSPSSSLSTQTSLQYRESFL MQDPYLLRLFLEINGAETEIKQYSKTELSQDTGGMSTDISSVVSNHEMGKS TYEDQQYPISSGGPVDLDCLWNY
HhNAC48	MMDTMESCVPPGFRFHPTDEELVGYYLRKKIALQKIDLDVIRDIDLYRIEP WDLIERCRIGYEEQNEWYFFSHKDKKYPTGTRTNRATMAGFWKATGRD KAVYDKSKLIGMRKTLVFYKGRAPNGQKTDWIMHEYRLESDDNAPPLEE GWVVCRAFKKRATSQMKNIETWDSTYFHDEPSSVTSVVDPLPLDYIKRQ QPSSFLNQNFMCKQEVEAENLIFAQSDHHFVQLPQLQSPSLPSLKKPTSSI SLISENIEEEQHISKRCKNNGTNKMATDWRDLDKFVASQLSQEEERGDQ RPGNYGNLSSFGGHENYSSDMALLLLQSGSDEDGNKLNEFLDSSCDIGICI FDK
HhNAC49	MENLSDDQMILPPGFRFHPTDEELITHYLSKKVVDSNFSATAIGEVDMNK IEPWELPRRAKMGEKEWYFFCVRDKKYPTGLRTNRATAAGYWKATGKD KEIFRGKPLVGMKKTLVFYRGRAPKGEKSNWVIHEYRLEGKFSLQNLPKT AKNEWVICRVFKKSSGGKKVHISGLMRLNSIENETGPNFLPPLMDSSLHG DRTTYTDSLHVPCFSNTIEIQKSQKEMISYFNNPVYPFTPNASDITIFQRISL PNSSFYGVQSAPIPGNLSYPGALPMQEQSILRCLFENYGSNVKKENDKISV SQETGLSTDMNTEISSVVDMGKRSFEDQEAPSTSVGPLGFDCLRNY
HhNAC50	MEKKVMNFVKDGAIKLPPGFRFQPTDEEIVFQFLARKITSCPLPASVIPDV DDNICKYDPWNLPGDLEQDKYFFSKKEAKYRNGSRSNRATGSGYWKAT GLDKQIVCPKRKQNVVIMGMRKTLVFYKGKPPHWSRTNWIMHEYRLVQ TNQSASTTNQNSMIQMGDWVLCHIFLKTGSSAETVDEVIQPTFSHDSSPT SSSSSSSSFSGTSSVITEVSSYGSDCEETRGRN
HhNAC51	MEGATAIVVNNGDNQDELLNLPPGFRFHPTDEEIITHYLSQKASNSNFTA TAIGEVDLNKCEPWDLPKKAKMGEKEWFFFSQKDRKYPTGMRTNRATE SGYWKATGKDKEIYSKGKDRHQLIGMKKTLVFYKGRAPKGEKSNWVM HEFRLEGKFSSYNFPKSPKDEWVVCRVVHKNTGTIKTSPTSDLTSLNSFVE GLLDPSSLPPLTESPYFNNNERPNDSAYTKISGTNTEDDFKGSVLVNPSTN NHQVQMQPQNYTFSPTTYYQTAPSTNYQVPNSILYQNAICYQATSSTLGY SHQQRINNSIPNISGSGNSMQTANLAQLKVEQFSSNQSIVSRSQETGLSTD MTTEISSKKEVDEGNRYEDLESFWSY
HhNAC52	MMSTGNGQQVVSVPPGFRFHPTEEELLYYYLRKKVSYESIDLDVIREVDL NKLEPWDLKDKCRIGSGPQNEWYLFSHKDKKYPTGTRTNRATTAGFWK ATGRDKAIHHSSYSQRIGMRKTLVFYTGRAPHGQKTDWIMHEYRLDDN NAEVQEDGWVVCRVFTKKTHTKIYEPEIHSQEHHHHRHKFTSISNLMDP KQNIQAMDDQHTTFDGSMHLPQLFSNESSSLSVLHPNTFLFPNSLSNTD

	MECSQNLLRLTSGGSGLIPVPERFTADWSFLDKLLTSHQNMDQQIKLSNNPSTQGLDHVGSSTTSVPRFPLQYLGCETTHNFKFSK
HhNAC53	TKPPTITSQSRIMDIEKIDNFIKLDDDEDDQALPGFRFHPTDEELVGFYLRRKVDKRPISLELIKHIDIYKYDPWDLPSSNMGEKEWYFFCRRGRKYRNSIRPNRVTGSGFWKATGIDKPIYCEGRRDCIGLKKSLVYYCGSAGKGTKTAWMMHEFRLPPPDYHHNTTTKNNIHFNDEFQEAEVWTLCRILKRNTSQRKCMGNWREISTSKRNKPTLDANSKPCSVESDNLQGYNISFTSTTSPVQIRQNNEHKSAAIYFSNHANELNQLAMTSVSQAPSSVTSSYSNISGPEVSEFEWYGAWDELGSIMEFVNDPFLF
HhNAC54	MSLSSLKMVEERLPPGFRFHPKDEELVCDYLMKKVTRCEHPPFLMEVDLNKSEPWDIPDSASVGGQERYFYSQRDRKYAIGLRTNRATISGYWKATGKDRPVSRRGSLVGMRKTLVFYQGRAPRGKKSDWVMHEFRLEGPLGAPRLSSKEDWVLCRVFCKSKEVMVAKQGIGISGCCYEDATTSSTKYYDLPPLMDIAPPPYITVGQTHDEQVHCFSNIFTQNQTNCPTLFSNFNSNSHHIMTRITKNMEQRPNISSKSTTLSPTTYLNNFNNNNTNNYNNTNTNNNNYYNNSPPPLNTMMIEDSATSFGEGSNSDISFLSEVGGFPTIWQNNY
HhNAC55	MTVQDASSCFGDAKFFPPGFRFHPTDEELILYYLKRKICRRRLKLDMIGETDVYKWDPEDLPELSILKTGDRQWFFFSPRDRKYPNGARSNRATMHGYWKATGKDRTISCNSRSVGIKKTLVYYIGRAPSGERTDWVMHEYTLDEEELKRCPSAQDYYALYKVFKKSGPGPKNGEQYGAPFKEEDWADDECVNFSGIVDQGNSMREPDVSCVDNSKTNGQAQFPLNDLVKEFKNQITDEPVVVPPLDVDHTYALDQLIGKEENESTLVNHCSREVSLPDQSLVLDHHSQQCYVQASFDYTQSATSQLQLHEASDVTSGPNICEQELGENFLEDFLEMDDLIGPELNVQNVEKPPEAFTFGEIDGLTELDLYNGATMFLQDIGTDEAGKILQPYMNNNLESGMFNMASHSYSNNNENEMVNYQVQPYLNDADQMSSQLWAQDQRYQALTPADANQAAACQPISDGWFLQTMSSSTWQEL
HhNAC56	MCPPPASPIAIKLWTDEALIVLLGDIILGHPLPSNVIGEFNPYNYLPSNLPDGIGYFISSEVKKDTELGSWKAKGDPCEIFTNSDITGWRTTLEFCEGQAPDERRTDWVMQEYRITQKGLCNNSNIKDSGTLCRLFHNEEQIPDHVIHPKNGGSLPQADSNTGQGSTSEAKEKNEDDETGPPLSVAERRKCVLNGDFLELDDLVDPQSPSSSSDNSSRPTFTSDELFDSVALLRDLEDENIGQQGKGESYKYTVSASVKPNEVVMCPATLGKLSAGGSQKSGYSLPDSAIERNIDNRVLIHAINCSNQITSASQNAVASGSHYAAINQVKEKKRKNGVDRMKKLKKYFCLIPL
HhNAC57	MDIFYHHFDSSESQLPPGFRFHPTDEELITYYLLKKVLDHNFTGRAIAQVDLNKCEPWELPEKAKMGEKEWYFYSLRDRKYPTGLRTNRATEAGYWKATGKDREIYSSKTCFLVGMKKTLVFYRGRAPKGEKTNWVMHEFRLDGKFAYHYLSSSSKVCSQLHVISHQITICTN
HhNAC58	MAGTLSVGFRFRPTDEELINHFLKLKINGLEEDVSVIGEVDVCRYEPWDLPGLSVVESIDNEWFFFCPKDRKYQNGNRSNRATVAGYWKATGRDRLIKASNGMNVIGRKKTLVFHRGRAPKGRGTPWVIHEYSATEEALNGTHPGQVAFVLCRLFKKRDEKQEEMAESSKCEEVEQYVSSPTVVKSSAEGTQSEPASLILSQQHEMQSISIEVIADEIANKTILDTPLPIADDAEGKGLDTSIQMMNELGPSYLHSSTTPCNDNKGVQLQDGTNSVDLDEFLNSLLVSSAEYSCENSGAVEQGFVKESGQLDTGFACGGFFTEAQKVSPLHLATPPGDCITPDFSNDKAQRDLHFLQNRWDALSAIPVGDQIYNMLNLEESCTSEDIGTGIRIRSHKPRFQPSEQGFVTQGIAPRRINLQMKLCLPKGFSYSEENSKVKPSASETLQAEEAKENYNSSATDAASTTTDAASTTTDAASNTDEIEEVSLFKPYKNEVIQELSLNA

	RQKGNCPISDRIMVPSVSLNAPILCSISSTQYIPWKLMVVGFVIVVVGIWG<br>CLLL
HhNAC59	MDYPRAPGFRFYPTEEELVSFYLHKKLLDHNRQDLDNVIPLLDIYEFSPW<br>DLPREVCDGDAEQWFFFIPRQEKEAQGGRPNRLTPSGYWKATGSPGFVY<br>SSSNRVIGLKKTMVFYKGRAPNGNKTEWKLNEYRAAAAATSAIEGLQLH<br>SSSAASTTLPPDHFRQELSLCRLYKRSKSLRSFDRRPLLTIGEGSGRVDHQQ<br>APPRSDHHEAATTSHHENQNSSSPILMVDSSSPSADAIALILLPSKKKKLGI<br>YFYRNRINQLIPFLISY
HhNAC60	MNTFSHVPPGFRFHPTDEELVDYYLRKKISSRRIDLDVIKDIDLYKIEPWDL<br>QELCRFGTGEQNEWYFFSHKDKKYPTGTRTNRATAAGFWKATGRDKAI<br>YSKHDLVGMRKTLVFYKGRAPNGQKSDWIMHEYRLETDENATTSQQEE<br>GWVVCRVFKKRIATVRRMSEHESPIWYDDQVSFMPDMDSPKQQPYMTA<br>YHYPYPCKKEQDFQYQIPPDHFLQLPLLESPKLLQAPPVTMTSCNSMPIY<br>GLNMNHAKHLQSSSSVLTQEQNASHPTYDQAVDQSVTDWRVLDKFVA<br>SQLSHEDVLKQNNDYANVFHSDDHSNMSLIRNLNKQEMAPVQNASTST<br>SSCQIDLWK
HhNAC61	MTWCNDCSDESGAIVRKSPPVPITDNNVISENHKHNHIEACPSCGHQIK<br>WQEKAGIHNLPGLPAGVKFDPNDQELLEHLEAKVRLDDHKLHPLIDEFI<br>PTLEGENGICYAHPEKLPGVSKDGFVRHFFHRPSKAYTTGTRKRRKVHA<br>DIDGNETRWHKTGKTRPVCTVQGKVIKGYKKILVLYTNYGKQRKPEKTN<br>WVMHQYHLGNNDNEKEGELVVSKIFYQTQPRQCSTSSIIKDSHPTKPNT<br>GVLNIAKGGSSMKNNSTLFIDYLL
HhNAC62	MGVPETDPLSQLSLPPGFRFYPTDEELLVQYLCRKVAGQHFSLQIIGEIDLY<br>KFDPWDLPSKAIFGEKEWYFFTPRDRKYPNGSRPNRVAGSGYWKATGTD<br>KVITTEGRKVGIKKALVFYVGKAPKGTKTNWIMHEYRLSDPQRKNGSAR<br>LDDWVLCRIYKKNSTAQKPLLGDIASKEHSYSHGSSSSSSSQFDDVLESLPE<br>IEDRFFTLPRMNSLNDELNFQNLGSGNFDWAILAGLNSMPEHVPGTQAP<br>MQAQSRGIMNNNNNTQNYMCVPSISPLGHVDTRFGKSMEEEVESGLRN<br>HRLDNSWFLNPNAYGLGFYGLS
HhNAC63	CVAPWSLLTSPPGVNPSCFKARRPLQLMESTDSSAGSQQQPQLPPGFRFH<br>PTDEELVVHYLKKKAASTPLPVAIIAEVDLYKFDPWELPAKASFGEQEWY<br>FFSPRDRKYPNGARPNRAATSGYWKATGTDKPVVTSAGSGGTQKVGVK<br>KALVFYGGKPPKGIKTNWIMHEYRLADNTKSTSMKPPGCSDVANKKGSL<br>RLDDWVLCRIYKKNNSQRPMDHESSDMVLGSIPSPPPPTCIQQIEQQNLK<br>LLPGLKAYGTTAMLENEQNIFESGMISTNTDGSISISGSKSHLISLPPYNVLS<br>MKRSLYWNNNEEGTPTGGSSSSTKRFFTDHINNGRISTTADENGSISSFLS<br>QIPQTPHATTTLHQQQADADAAMLGNLEERSCDI
HhNAC64	MAPVGLPPGFRFHPTDEELVNYYLKRKIHGLEIELDIIPEVDLYKCEPWEL<br>AEKSFLPSRDPEWYFFGPRDRKYPNGFRTNRATRAGYWKSTGKDRKVSS<br>QNRAIGMKKTLVYYRGRAPQGIRTDWVMHEYRLDDKELLHIDSYALCR<br>VFKKNGICGSELEDQGQPSIPLIEYSQGVIINEYETMSPADVTLASSSCMDE<br>EDKDDSWMQFITDDAWCSSNNPIGGEEGSQVTITD
HhNAC65	MTAVELQLPPGFRFHPTDDELVMHYLCRKCASQQIAVPIIKEIDLYKYDP<br>WDLPGLALYGEKEWYFFSPRDRKYPNGSRPNRAAGSGYWKATGADKPI<br>GNPKPVGIKKALVFYAGKAPRGEKTNWIMHEYRLADVDRSARKKNNSL<br>RLDDWVLCRIYNKKGTLENHNVGSRKVSCPTGIEEKKPEIKTPATDNSPA<br>MFGDMMYLDTSDSVPKLHTDSSCSEHVVSPEFTCDREVQSVPKVEEWEN<br>SPLNFPLNYTDATVDNNVNLMFGSQPQQGNYQTSQWHDMFMYLQRSL

HhNAC66	MAPVSLPPGFRFHPTDEELVAYYLKRKINGRKIELEVIPEVDLYKCEPWDL PGKSLLPSKDLEWYFFSPRDRKYPNGSRTNRATRAGYWKATGKDRKVNS QMRAVGMKKTLVYYRGRAPHGARTDWVMHEYRLDERECETASGLQDA YALCRIFKKTLNAPKIINHYGITASDHSSSNIEIYSCEDNMENSDHPMPFST YPPNTTSIMHGSPRNIGGTSTNDAKWTQYLSEEAFSFTNPSFPDCGAIAYP PSKVDIALECARLQHRLSLPPLQVQDFPQDGFVDFQMPQSSNSMYGNTS DQQDILQEILSVAQVSQELMTQNSWIGNNSSEDDFSFLPHGNQIQGMSST RSAEIGENMDEQFKTERDVENLRWVGMSNKDLEKVITTPTIHSKNINLGF SRVFYLFIFCTWQSFLEDYKTVPIEDISSFQREEHEHFSLGFTNDDPNDQSF LDDGDLDDFSSTPSFEVYEKIEVNHGLFVSTRQVPNTFFHQIVPSETVKVH LSPVVHNFPISKLDSPTMPENTNLFDKVKAFTTTKLVGTTKSLRPWRKIES PIVGIIALILAYCLYMGEQRDDYKNLRDCDDLADGIKVVKRRREKGCHSL EKIESTTVKWDYEQKKVGFVGMTEWGIVVLNKMWPCLTLALASYFYHL GAAHFT
HhNAC67	MANVNNNGNGNGNGFQVPMGYRFRPTDEELLLHYLKPKVHSLPLPAS VIPDHFHLFQSHPSHLPGDSKEKRFFFCKRKWNNVHKCRITTISDGSGYW KAIGKDKAINVTPAAASAALGTKKSFVFYQGTRPHALKTRWLMVEYRLV PSQITTKSTQELEDWAVCCIYQRKRKAKNHGISSKTRRNMEEEVVIMPSC DGMMDFMILDSSDQFGPPPLPSPSCSSGNNTCSNNELLDQEEECSSNSFG PYFSCF
HhNAC68	CCRSWLVDSKGIASKVKNATLPSENQIKDCGANRECPKCHHRIDNSDGS HAWPGLPPGVKFDPSDAEILDHLAAKCRVANCVPHMLIDEFIPTLDGKN GICYTHPENLPGAREDGSNVYFFHKTINAYATGQRKRQKIQTEDSLTKEH VRWHKTCKTRPVIENGTQKGCKKIMVLYRSSVKGSKPCKSNWVMYQYH LGTEEYEKDGQYVVSKIFYQQHKQADKNSNFVAITEHDKGTMHTSPKTP KTNTPNPPRSRKSDACDDVMDDYLLESPIQVADNFHYFIAKVQMLNSYM PPNPSGESQAVDLDSTEDLLFCNEILDSYVPLNNSGLYHGQSPACVRDIN NAPSAERSESCGIAELENIELDTPPDFQLSVSFASYLSSISSFAF
HhNAC69	MENLSDQQMELPPGFRFHPTDEELITHYLSPKVHNSSFSATAIGEVDLNK VEPWDLPWRAKMGEKEWYFFCVRDRKYPTGLRTNRATEAGYWKATGK DKEIYRAKKLVGMKKTLVFYKGRAPRGEKTNWVMHEFRLEGKYSNNNL PKTSKNEWVISRIFHKSSGGKKIHISGLVRKGNFGDELQPPNLSPLLDISRT TAAATSHVTCFSEQLEDGKPQDEIVLPSSSSILSPKLPIPSLFMSTQISLQYPE SFLMQDPSLLRFFLEINGAEAEMKQNSKTEFSQDTGGMSTDISSVVSNQE MGRGTCEDQEYPISSGGPVALDCLWNY
HhNAC70	AKSVEMMDTMESCVPPGFRFHPTDEELVGYYLWKKIASQKIDLDVIRDID LYRIEPWDLIERCRIGYEEQNEWYFFSHKDKKYPTGTRTNRATMAGFWK ATGRDKSVYDKSKLIGMRKTLVFYKGRAPNGKKTDWIMHEYRLESDDN APPQEEGWVVCRAFKKRATRQTKNMETWDSTYFYDEPSSVTSVIDPLPLD YITRQQHSSFLNQNFMCKQEVEAENLIFSQSDHHFVPLPQLQSPSLPSLKR PTSSSVSLISEKNEEEQHISKRCKNNDTNKMATDWRDLDKFVASQLSQEE ERCGQGGGYGNLSSFGGHETYSSDMALLLLQSGSDEDGTKLNELLNSDSS CDIGICTFDK
HhNAC71	MENLSDDQMILPPGFRFHPTDEELITHYLSKKVVDSNFSATAIGEVDMNK IEPWDLPRWAKMGGKEWYFFCVRDKKYPTGLRTNRATAAGYWKATGK DKEIFRGKSLVGMKKTLVFYGGRAPRGEKSNWVIHEFRLEGQFSFQNLPK TAKNEWVICRVFKKSCGGKQVHISGLMRLNSIENEMGPNVLPPLMDSSL HGDRTTVTESLHVPCFSNTIEIQKETINCLNNPVYPFSPNASDITIFQRICLP NASFSGVQSAPIPGNLPYPGTLPMQDQSILRGLFENYGSNVREILKTEKDM ISVSQETGLSTDINTEISSVVDMGKRSFEGQEAPLDFDRLWDY

HhNAC72	MSNLKMVEAKLPAGFRFHPRDEELVCDYLTKKITRSEQPPILMEVDLNKS EPWDIPEYACVGGKEWYFYSQRDRKYATGLRTNRATDSGYWKATGKDR PVSHRNSLVGMRKTLVFYQGRAPKGKKTDWVMHEFRLEGPLGPPKLSSS KEEDWVLCRVFYKNKQVDHNHSPGSKQGMAGNRYEDTISLSSTYLPPL MDDPPPPPPLYNEYQIHDDYHQSSLCEQVPCFSIFNPMNIISSKFITTTTNS SVPNNTFAGGGMAPDQLINGNTSCEDKMVIDAVLNHLMDNINVNNSD QHHPTNNMVKMMMKDSPCSFGEAASTSESFLSEVETIWNNY
HhNAC73	MEKKVMNFVKDGAIKLPPGFRFQPTDEEIVFQYLARKITSCPLPASVIPDI DDNICKYDPWNLPGDLEQDKYFFSKKEAKYQNGSKSNRATGSGYWKAI GFDKQIVCPRRQQNVVIMGMRKTLVFHKGKPTHWSRTNWIMHEYRLLQ SNQSASTTNQVHNWHIYIYIYIYIYIYIYIYIYIYIYIKEIFKFTSLSCYINLFL FFFKYCFCNLQNSMIQMGDWVLCHIFLKTGSTLAETVDEIIQPMFSCDSM SNLSDDDSSPASSSSSFSGTTSVITEVSSYGSDFEQTGGRN
HhNAC74	MEENLPPGFRFHPTDEELITYYLVHKVSDFSFTSRAVTDVDLNKSEPWDLP AKASMGEKEWYFFSLRDRKYPTGLRTNRATEAGYWKTTGKDKDIFRGG VLVGMKKTLVFYRGRAPKGEKTNWVMHEYRLENKHAFKPTKDEWVVC RIFQKSATVKKPQQTTSSQQSLDSSCHTNTMVNEFGLFDHLPNLNNIVNS STMMNNISLQNYTNDENNLNMSMNSLLPPSLSWPSGLLSSNLSMNSLLL KALQFKNYQQREMTTSTNLDYASFMPQGISTPYGPGFTSNFAASSSSSKGL DNSVQQQQEHQEQPFNLDSIW
HhNAC75	MESESCVPPGFRFHPTEEELVGYYLRRKINSLKIDLDVIVDIDLYRLEPWDI QDRCKLGYEEQNEWYFFSHKDRKYPTGRRTNRATTAGFWKATGRDKAV LSKESIIGMRKTLVFYKGRAPNGMRSDWIMHEYRLQSSEHGPPQAKGWV VCRAFKKPTPNQKQGFEPWNNGYYFQNNNNFSSSPSFQEILNPLNAALT NQSTNFYQVPFANSNQQQFFPNDSHFDNEIVDLPQLDSPTLSVSLDPDQE NCNGWKNMDKLLAPKPVEHALFSFPNMPLVPCEDELDAQNHLNHFLG YFPEL
HhNAC76	MGYRHKMVAVELQFPPGFRFHPTDEELVIHYLCRKCASQQIAVQIIKEIDL YKYDPWDLPDLAVYGEKEWYFFTPRDRKYPNGSRPNRVAGSGYWKATG ADKPIGHPKAVGIKKALVFYAGKAPKGDKTNWIMHEYRLAGVDRSVQE KNNSLRLDDWVLCRIYNKKGKPEKNNAGSQKLSHPMEIEDKKPEIITPAT ETSPAVFGDLVHLDPSDSVPRLHTDLSCSEHVVSPEITCDREV
HhNAC77	MARMFLPLGFRFHPTDEELIMYYLKRKVMGKKLHFEAIAELDIYKFGPW DLPDESLLKSKDLEWYFFCPREKKYASGARTNRATKYGYWKTTGKDRSVI YKQRTVGMIKTLVFHKGHAPKGERTDWVMHEYRIEDKNMGDVVQEYM LCRVFKKSGPGPRNGAQYGAPFNEEEWSDEEICSESSPVDGRDSFHVLPS NMTNSAAVRLTVPGSSCSHSISKPGQSTAGPSNIDVSYDMPDLPEEYRGA LLNMLTEDNTMLPIENSIAEKFKIANNDKSIEALPCLDGNENEIYNELGDL NTLEELMDAEFNLPTNQDVGYPQNSMLLGDDSSFLELNDLDIPLRCPAE ASEFELVQTGNTYVHHTPYRNLDGSYCEGNYTSAVQYVSELNQHSVLLE GSHWLGDNLEVARKENGFGNFEAHGVDAVTCSDIPVSCMQPEGNNFTE QIQSREPVTRLH
HhNAC78	MEQNSVKVNGQGGGVGLQIRLPIGFRFRPTDEELLLHYLKPKVYSSPFPSS VIPVLHDDIIFHSHPSHLPGDPKEMRHFFCKRKWDAYVKNYSSSRIRITTS DGSGYWKPIGKEKTIHIVAPGLLTPLVGIKKSMVFYQGKPPHGLKTPWIM HEYRLLPPQTTHNSTNRELENWVVCRINQRKRKSKNHGSNKTTGIWKRR
HhNAC79	MPRSWLVDGRGIAKKVKNASLPYANQIKDCGGQRECPKCHHLIDNSDV SHSWPGLPPGVKFDPSDVEILEHLAAKCRVGNSTPHMFIDEFIPTLDGDN GICYTHPKNLPGAKEDGSFVYFFHKPTNAYATGQRKRRKIQAEDSLTKEH

	VRWHKTGKTKSVMENGNQKGCKKIMVLYRSSVKGSKPGKSNWIMHQY HLGTEEDEKDGQYVVSKIFYQQHKQLDENDNSIAIIESDKSMIRTSPKTPK TNTPNPPRLGKPDSCDDVMDDYILESPIQEAEFIKEISCPSSQLQFKDEIEYP SFLAGESQADDLDSIEDSLLCNEILDSYRFNDSGLHHDPSSSFVRNAHGAE KSETCGITELEDLELDTPPDFDLANLQFCSQDSIFDFLDRL
HhNAC80	MDDRNEVDKIDDVMLPGFRFHPTDEELVDFYLKSRVQQRPLPIELIKQVD IYKYDPWDLPNLASSGEKEWYFYCPRGRKYRNSARPNRVTGTGFWKATG TDRPIYPSNGTKCIGLKKSLVFYRGKAAKGIKTDWMMHEFRLPSIPDAGP PKKFLDKNLPPHDSWAICRIFKKANSMAHRALSHSWVSPLPETTSSDMFT QGSHSTQFSSENMSCTTETTSAIQLCNEWQQNSTASYYALDIPSYRPINPA FYKPFSLGAPNGDLHNDLVFSPPDMFGSSAKSTVDPVTYNYKLLPRFPTN YHNCCYPKIKTIMLLSSTTSCSIQITQHTNSGKLYLHSILYVFETKVGILRGK PLIHTQDNCWNISHKSYIR
HhNAC81	MNNFSEEQMELPPGFRFHPTDEELITHYLTPKVDNSSFSDVAIGEVDLNK VEPWDLPWKAKMGEKEWYFFCVRDRKYPTGSRTNRATESGYWKATGK DKEIHKAKTLVGMKKTLVFYKGRAPRGEKTNWVMHEYRLEGKYSMNN LPKTAKNEWVISRIFHKSSGGKKTHISGLVRLENYGDEFKPSNLPPLLDISR TTKAETSHVTCFSEQQMEDRKFQDKTVDTFNPCVLPSSSPSKPFDSSFLSP KIPISNSFFSTQISLQYPESISIQDQSFLRYFLEYNWEESEMKNSKTEFWGMS TDMSSTVSNHQMGQRSYEDQEYPITSGGPVDLDCLWNY
HhNAC82	MISTMDTMESSCVPPGFRFHPTDEELVGYYLRKKVASQKIDLDVIRDIDLY RIEPWDLIERCRIGYEEQNEWYFFSHKDKKYPTGTRTNRATMAGFWKAT GRDKAVYDKTKLIGMRKTLVFYKGRAPNGQKTDWIMHEYRLESDENGP PQEEGWVVCRAFKKRTTTSQTKNMEPWDSTYFYDEPSGVCSVVDPLDYI TRQPSSSFLNQSFMCKKEIEAENLIFVQSEHHQFVQLPQLESPSLPLLIKRPT SSVSLVSLNNEEEERNRGCNNNINESSTNKNKNKVAADWRDLDKFVAS QLSQDQEDQKYEGGGSDGILMTSFGGHEITGSSNHDDPMALLLLQSGDS EHQQGNYKLNEFFNSDSSCDIGICIFDK
HhNAC83	MENLSLKADDQMELPPGFRFHPTDEELITHYLSKKVVDSNFSATAIGEVD MNNIEPWDLPRRAKMGEKEWYFFCVRDKKYPTGLRTNRATAAGYWKA TGKDKEIFRGKSQVGMKKTLVFYKGRAPQGERSNWVIHEYRLEGQFSLH NLPKTTKNEWVICRVFKKTSGGKKVHISRLMSSSENEMAPTALPPLMDSL LPSGRTIVPTIKESIHVPFFSSTIDIQKSQNERNNYFNNPVHPFPSNASDILQ RISLPNSYYRFQSAPDPGILQYPGTFPMQNQAILRGLLENYDSNIRQNFKT EKETGSVSQETGLSTEISSVVSNLDMGRRSFEDQEAPLTSVGALDFDCLWS Y
HhNAC84	MNKSNTQLGSSSISSSDLIDAKLEEHQLCGSKHCPGCGHKLEGKPDWVG LPAGVKFDPTDQELIEHLEAKVEAKEFKSHPLIDEFIPTIEGEDGICYTHPE KLPGVTRDGLSRHFFHRPSKAYTTGTRKRRKIQTECDLQGGETRWHKTG KTRPVMLNGKQKGCKKILVLYTNFGKNRKPEKTNWVMHQYHLGMHEE EREGELVVSKIFYQTQPRQCNWSDRINSTSGLVIGGNEGNINSEPNSRRES GSGGCSSKEIATHRDEVLSVVGVGAPILSYNPVEMQQFKAHDSYSFVPFG KNFDDQVGTRGGEASTATESTTLTMTGTCEEHDLQRPHHHMTHQQLAT AANFHISRPSHSISTIISPPPLHHTTSIILDEDSFHVSRMMLLNENFQQHQQ QQHHKLGGRSSSGLEELIMGCTSTDIKEDSSITNPQEAEWLKYSTFWPDP HDPDNPDRHG
HhNAC85	MNQELVTAVPPVEIAAPPPPTKSLAPGFRFHPTDEELVRYYLTRKACGKP FRFQAVSEIDVYKSEPWELEGYSSLNSRDLEWYFFSPVDRKYGNGSRLNR ATGRGYWKATGKDRAVRHKGQTIGMKKTLVFHSGRAPDGKRTNWVM

	HEYRLTDIDLEKAGVAQDAFVLCRIFQKSGLGPPNGDRYAPFVEEEWDDDTALLVPGGEAEEDVVNADDARAVRNDLERDANKAPLRLSEPPIDSQTLPFACKRERTEDFPSNPEANPELVPLFEIKRAKHSDPNSSHANGSEDSTTTSQEPTAPSTNFALLEFPLLDSIENRGSRLPANPATFDAANLEKSVPPGYLKFISNLENEILNVSMERETLKIEVMRAQAMINILQSRIDILNKENEDLRRVAHGIEAEMAI
HhNAC86	MENEPISSSHFPPGFRFHPSDEELIVHYLLKKLCSRPLPAQVVAEIDLYSYNPWELPKKALFGEDEWYFFTPRDRKYPNGARPNRTASSGFWKATGTDKPILGASGSRRIGVKKALVFYTGRPPKCLKTDWIMNEYRLPDTMTRPSRSKGSMRLDDWVLCRVHQKGNMSKKTWAVEDSPSKLVSFMPKIGELPSAYTAANTGITTDYLLKDCQILASMLACQHLSPIKNISNATFPRRNNSNSENTSGKGNPFTTTSFFESFLNPTIAKFNEGKRFEDLIDSDKMDTMGNNNEVLLPGNMMTTNAMNFYNQNQAQGDIFNPSHSDSHLSNIFLFQNKTLISSDGSIK
HhNAC87	MEEFPPGFRFYPTEEELVSFYLHNKLQGISQSDLNRVIPVIDIYEVEPWHLPKFSGELCHMDMEQWFFFVPRQEREARGGRPNRTTASGYWKATGSPSYVYSTSNKVIGVKKTMVFYKGKAPTGRKTKWKMNEYRAIEEEVVTSSSSSSTNCAAPRLRHEMSVCRVYVVSGSSRAFDRRPFGTVIRETTVDDQEVHGNGVAKCAENAPKIEKTSSPEPESSYSGEKELTVDFIENEGTFNFEMINGLEWELEQLVWQ
HhNAC88	MKEDRQFISAVGEFPGFRFSPTEEELILYYLKKKIEGGFDKCVEVIAEIDMTQDEPWELPAKSIIQSDDEWFFFSARGRKYPRGLQSRRATESGYWKATGKERNVKSGSDIIGTKRTLVFHKGRAPKGERTEWIVHEYCMSGTSQDSLVVCRLRRNCEFHFHENQRKKSSDGSKSPSARDSSTSALAEEGLQHKDFFKGGMAADGCSKNCISSDNSQSVEHIDSECESDHKLASDVFQDGSCSMQKGCDGEDDWFADILNDDIIELDESSLSTSTNLLQPVATGSKVEVKSTQPEQGIEIGMLPSQGTADRRIRLRRQKEKKYTVKPLEVANLSGDRNSIELVLCASVFEDPFAFFKFSVQLVSFVPRYLRSLWSLEKSFSFHGTKYCSSSYSSSSLSNL
HhNAC89	MGLRDIGAALPPGFRFYPSDEELVCHYLYKKIANEDVLKGTDLLEIDLHTCEPWQLPEVAKLNSTEWYFFSFRDRKYATGFRTNRATKTGYWKATGKDRTVVDPRTNAIVGMRKTLVFYKDRAPNGIKTGWIMHEFRLENPLHPPKEDWVLCRVFHKAKTENNNTEVLSPQNYVFDAKVTAGDTSPPAAQTLPLGYHHQITSDFSQNHQNQNPNNNQELLSPLNPNFLQLSQAHTDALARVTEDEYGFLFDISFEESNLGDGMASNMEGVRFEDL
HhNAC90	MVNCAISSSSSAHDEEEEHMLMPPPPGYHFVPSHSQIIEIYLVNKILGCPSSSDMIKEMDLMQQLDPEHLPFDEFIYCRKNEAYFITPTKERNTEGSRTILTTTSGYWKASKDEVPVFNSERTVGYKKTYVFYQGNEPAGEKTLWRMNEYRVRLDIIPADALYEDVRSKIEHYVACKIKYKKPESPAEPLQEDEDEDEE
HhNAC91	MAIAFTMNLQENHDDENKNNNIEEHEHDMVMPGFRFHPTEEELVEFYLRRKVEGKCFNVELVTFLDLYRYDPWELPALAAIGEKEWFFYVPRDRKYRNGDRPNRVTTSGYWKATGADRMIRTENFRSIGLKKTLVFYSGKAPKGIRTSWIMNEYRLPHHETERLQKAEISLCRVYKRAGVEDHPSLPRSLPTRASSSRSNQKHQDATQHAIEKFQSLGELQTQQINDEKINETSGNSTTDEIGTALGLSNHNIYIPLAPINALLSPQNCNTIYNTGSSSLVTTMNLNNSIDDLHRLISYQQDASVNVQQQFYHNNSETNLQHV
HhNAC92	MGDDNVNLPPGFRFCPTDEELVVHFLQRKASLLPCHPDIIPDLDLYPYDPWDLDGKALAEGNKLYFYSRSSQNRITSNGYWDSMGGDEPIFTTDTTRRVGTKKYYAFYIGELSEGVKTNWIMQEYRLWNGGSCSKSSKKRNSKLDHSKWVVCRVYEHNYDDNGDETELSCLDEVFLSLDDDFDEISLPN

HhNAC93	MDASKTSKMGLPGFRFHPTEEELLDFYLKNTILGKKKSHYDIIGVLNIYRH DPWYLPGLAKIGEREWYFFVPRNIKHGNGGRPNRTTEHGFWKATGSDR KIVSLSDPKRVIGLKKTLVFYEGRAPRGTKTDWVMNEYRLPDSCPSHKDI VLCKIYRKATSLKVLEQRAAVEEEMKTTNTKTTPPPSPLTPPISMQTVSFC CQNEDSTAAAASLPLLKKEEEEEEEEEEPMITEEKNFLNDEEGNITKCLNL QLPTGMEKLELQLPKFGSQDPFYAPLCSPWLDNWLITYANVLDL
HhNAC94	MRSWCYEMEILICPSCSHIIQLQDQAGFEDLAALPAGVKFHPSDQEIVEHL EAKVTQDHSHKRHPLIDQFIPTLEGENGICYTHPEKLPGVNKDGQIRHFF HRPSKAYTTGTRKRRKVQTDAADAETRWHKTGKTRPILIDGTVKGYKKI LVLYTNYGSQKKPEKTNWVMHQYHLGDNEEEKDGELVVSKVFHQTQP RQCGSSVKELSPSEKASMSLSRVYHNPIIKNPSITKDKYFDSQNSRKEES
HhNAC95	MNSINSSATAQHPRLPPGFRFHPTDEELVVHYLKKKAALEPLPVTIIAEVD LYKFDPWELPSKASYGEQEWYFFSSRHRKYPNGTRPNRAATSGYWKATG TDKPILTSKGGHQKVGVKKALVFYRGKPPKDSKTNWIMHEYRLVENSNP SMKPPCTKKHSSRLDDWVLCRIFKKKSTISRPMERDNDDYSIEDNNNFPK LGNYVGLLTDHKESIFEGTTLHGNGMQKNSSMSRMEYPSSSKLSDISMLS TRGKRTLESQQQLWNIASLNATDHGNGSFNSLLINPFPQGSAPFQSNTSR TVLGDGLLHQPNINWN
HhNAC96	MGGTSLPPGFRFHPTDEELVGYYLKRKVERLEIELEVIPVIDLYKFDPWELP EKSFLPQGDMEWFFFCPRDRKYPNGSRTNRATKVGYWKATGKDRKVVC QSSVQGYRKTLVFYRGRAPAGDRTDWVMHEYRLSDDVPFALCRIIKKNE QALKTGDIYGDPRSKLVGSSSRNEDFTSRRIHNEPMIIPDDMPFQASYMGS VSNYSTPLSSPYQTTPIVESESVRMETNPSSLWVSPDFILDSSKVFLDSFLFLF FLMEFSKKVNPQFKGLSGYFPPNQFSTNLMTAWKQYDQFEISSSSSYSNFT EKVELADDHSQFGCMSPYSGHEDYMNFFGNGGMPHEGYDWSNSLNQK PFLKTNWRNHSISISSMQEWKLGTKLQFVDKAAVMGVLENWSVVARR
HhNAC97	MNLNMNTFSHVPPGFRFHPTDEELVDYYLRKKVALKRIDLDVIKDVDLY KIEPWDLEELCKLGTEDQNEWYFFSHKDKKYPTGTRTNRATKAGFWKA TGRDKAIYYKHNLVGMRKTLVFYKGRAPNGQKSDWIMHEYRLETNENE DGWVVCRVKKRLTPVRKMDAHEPLCWYDDQVSFMPDFDSPRQIPQPYT PYHNHHHPCKQELELQYYRTPHERFLQLPHLESPKVLQLSAANASCNSV TPFGFEKSSTLTQEEHMKQNSQVLNINALYGSNNIERAVDQVTDWRVLD KFVASQLLSQDQDDTKEGTCSNAQVAEQINMSKPWLLQKVKNWSDISQ MKIPPPTNSLLQIRTTQKMQKNPPHS
HhNAC98	MSSCFDDAKFFPLGFRFHPTDEELILYYLKRKICRRQLKLDMIGETDVYKW DPEDLPELSKLKTGDRLWFFFSPRDRKYPNGARSNRATVHGYWKATGKD RTISCNSRSVGIKKTLVYYIGRAPSGKRTDWVMHEYTLDEEELKRCPAQD YYALYKVFKKSGPGPKNGEQYGAPFKEEDWADDECVNFSGMVDQGNS VKELDVSCVDNNKANGQAQFPLNDLVEEFMNQIADEPVIVPPLDVDYT YVLDQLIGEEETENTLVNHCSREVNLPDQSLVLHHSQQCNVQASFDYTQ SATSQLLLYEASVVTSAPNICEQELGENIHEDFLEMDDLIGPEPNVQNFEK PPEAFSFGEIDGLTELDLYNDATMFLQDIGSVEAGKVLQPYMNNNLEMF NMASHSYSNNNENEMVNYQVQPYLNDADQMGSQLWTHDQRCTALTP ADADLAAACQPISGVICNGNPANLPTEATQCQSGEVDDGTYSWFSSAL WAFVESIPTTPASASETALVNKAFERMSSFGRLNMNSGKTNDAAGNPTA ASRRSGSSKAGIFCFSILGIFCAILWVLIGTSMNLLGRCISS
HhNAC99	MDIEKMDNFIKLNDDEDDLLPGFRFHPTDEELVGFYLRRKVDKRPIGLELI KHIDIYKYDPWDLPEGSNMGEKEWYFFCKRGRKYRNSIRPNRVTGSGFW KATGIDKPIYSEGHRDCIIGLKKSLVYYRGSAGKGTKTEWMMHEFRLPPP

	GYRQNTTTKNNIHFNDEFQEAEVWTLCRILKRNTSQRKGMAEGREITTSS<br>KRNNPTLDANSKTRSAESDDLQVYNISFTSTSPVQIRQNNEHKPAAIYFP<br>NHVNELNQLATTSISQAPSSVISSYSNFSSPEVSEFWRYGGEWDELRSIMEF<br>VNYPFLF
HhNAC100	MEGAAIVVNDGANQDELLNLPPGFRFHPTDEEIITHYLSQKVFTSNFTAT<br>AIGDVDLNKCEPWDLPKKAKMGEKEWFFFCRKDKKYPTGMRTNRATES<br>GYWKATGKDKEIHSKEKDRRQLIGMKKTLVFYKGRAPKGEKSNWVMHE<br>FRLEGKFFPKSPKDEWVVCRVVHKNTGTIKTSPTRDLTSINSFVEGLLDTPS<br>LPPLTDSPYFNNNERPNDSTFTKISGANTEDDFKGTVLVNPSMNNHQLQ<br>MQPQNYTFNPTACQTAPTTNYQVPNSLLYQNAIRYQATPNTLGYPHQQ<br>RMINSIPNVLGSSANPVQTADQALVRQLKVEQFSSNQSIVSPSQDTGPSA<br>DMTTEISLKQEVDEGNRYKDLDSFWSY
HhNAC101	MSEDMNLSVDGHSRVPPGFRFHPTEEELLHYYLRKKVAYQKIDLDVIPDV<br>DLNKLEPWDIQEKCKIGSTPQNDWYFFSHKDKKYPTGTRTNRATAAGF<br>WKATGRDKVICSSLRRIGMRKTLVFYKGRAPHGQKSDWIMHEYRLDDN<br>TTNHHETTSTDSTSLVVSEYSTSAEDGWVVCRVFKKKNYHRALESPQKSS<br>TMDSRRSHMYLTNSAKDEGVLDQILLYMGKSCKQEDHETVVVSNNNN<br>NLTTMQSRNPINNTLAAMITSTSADELVHGRFVHLPRLERSTSPTIPSLLRP<br>IQDCTTFKSLGPSIDEEVFSETQGNMVDDPKDGLINDWTALDRLVASQLN<br>GQAETSKQLSSCCYAGHPDREDGDFCFSSFDHHDLGQLSNPRCNRLNQA<br>SQVYNSEIELWSFVQPSSSSSSDPFCHLSV
HhNAC102	MNTFSHVPPGFRFHPTDEELVDYYLRKKVASKRIDLDVIKDVDLYKIEPW<br>DLEELCKLGNEDQNDWYFFSHKDKKYPTGTRTNRATKAGFWKATGRD<br>KAIYSKNSLVGMRKTLVFYKGRAPNGQKSDWIMHEYRLETDENGSPQEE<br>GWVVCRVFKKRLTTVRKMDEHEPLCWYDDQVSFLPDFDSPRQTTQPYTP<br>YRNHQYSCKQVLEFQYYMPQERFLQLPQLESPKFPQSTASVICNSVAPYG<br>FEHSSTLTQEEHLQQNSQALNFRAIYGSNNVNIEQAVDQVTDWQVLDKF<br>VASQLGQDQDHATKETNCSNAQISEHINMLCNDLKRPEVDSESMSISTLS<br>CQIDLWK
HhNAC103	MMAWCNESPSDEIAIITIDSKIERNRTLTCPSCNHSIELQDQGGVQDLPGL<br>PAGVKFDPSDQVILEHLEAKMLYDHGHKLHPLIDEFIPTLEGENGICYTH<br>PEKLPGVNKDGQIRHFFHRPSKAYTTGTRKRRKVHTDADGGETRWHKT<br>GKTRPVLVDGTVKGYKKILVLYTNYGRQKKAEKTNWVMHQYHLGDNE<br>EEKDGELVVSKVFYQTQPRQCGGSSVKAINESPNETSSKSLSSRHHDINPM<br>IKNPGFIEFYNPNFISYDAGSQNREIPHQLIPNLTFQGDDSSFFNLSTGASK<br>GS
HhNAC104	MAGASLPPGFRFHPTDEELVGYYLKRKVEGLEIELEVIPVIDLYKFDPWEL<br>PEKSFLPKRDMEWFFFCPRDRKYPNGSRTNRATRAGYWKATGKDRKVV<br>CQSSVTGYRKTLVFYRGRAPAGDRTDWVMHEYRLSDEVSQGSPTSKGPF<br>ALCRIIKKNEQALKTSDTSGHPKVKQVGNCSSHGDFSSNRSLHEPVIVPD<br>GMPFQASYTGSESNYSSALTSPYQTTTMVETEKAPVGTSPSSLWVSPDFIL<br>DSSKGYPQGQEGLSGYFPHNEFPNSMTPRQPYEHFEISPSSSHSNFTEKVE<br>LNDYFSHFSSMSPYSGQGDYTGLFGNGSISQEGYGWSNSLNPPF
HhNAC105	MDSELASSKSGSLAPGFRFHPTDEELVFYYLRRKICGRPFRFDAISEIDIYKV<br>EPWDLPGKSRLKSRDLEWYFFSVLDKKYGNGSKTNRATDRGYWKTTGK<br>DRAVYHRTQIVGMKKTLVYHNGRAPRGERTNWVMHEYRLIDEAMQKA<br>GIGQDAFVLCRIFRKSGSGPKNGEQYGAPFIEEEWEDDELVLVPKDEYSEE<br>MPVSDDACPDAYLDENDLEQILSAVDPSHEAPLAYGDENHYVDESTHSF<br>DDAQEVDLSNEAPLPLNVAYGDENHYVEETTDFFGDAQEPLVGVDENY

	VQERPDGRTLFNLPVQYGMDAKSVKREYIGEPSYTGESSNAENPVDVDYF LDESFLDAMDNPQFGDGTFIETNDLKQPAVVDPSSFDMLDEYLQFFNAT DENVQHRDFDYSNMMASEDLLSDPASLLTYKDVNEGIKQETVEGKQQL EDHSNDVPSSSKQEPTKFGSDFHYPFTKHASRMLGNIPAAPAFASEFPTK DRTLRLNSASSSSIDVTAGIIQIRNVTLSGSGMDLSFGKHGRVNIILSFGLPS GNDYSADSQPAYSILSDKANSAISRGWFYFMFMWVLFLTLSLKIGSYICAN
HhNAC106	MDQELVTATPPAAIAAAPIAKSLAPGFRFHPTDEELVTYYLRRKACGKPF RFQAVSEIDVYKSEPWDLAGYSPLRDMEWYFFSPVDRKYGNGSRLNRAT GRGYWKATGKDRSVRHKGQTIGMKKTLVFHGGRAPDGKRTNWVMHE YRLVDIELEKAGVAQDVFVLCRIFQKSGLGPPNGDRYAPFVEEEWDDDS ALLVPGGEAEDDVANGDDARAGENDLEQVFSFQDICICTIVFILYREITSF PRHSHCILHNQYLRNFSLMWLAELVNQNNNKIKTTNKP
HhNAC107	MNKTSNYTISSSDLIDAKLEEHQLCGSKHCPGCGHKLEGKPDWVGLPAG VKFDPTDQELIEHLEAKVEAKELKSHPLIDEFIPTIDGEDGICYTHPEKLPG VTRDGLSRHFFHRPSKAYTTGTRKRRKIQTECDLQGGETRWHKTGKTRP VMVNGKQKGCKKILVLYTNFGKNRKPEKTNWVMHQYHLGQHEEEREG ELVVSKIFYQTQPRQCNWSDRVNSTSGLITGGNEGNNISEPVNSRRDSTGS GSCSSREISINHRDDQMSAGVGAPISSYNPMDIHQLKVHDHYSFVPFRQIF DEVGTTGGEASTRETQVTTTGGTCEEHDLQRPHHHMTHHPHHQPHQQ QLATAAFHISRPSHSISTIISPSLHHTTSINILDEDSFHVSRLMLQNENFQQQ PQQQQLQHQQQQHHNKLGGRSASGLEELIMGCTSTDIKEESSITNPQEA EWLKYSPFWPDSHEPDHPDHHG
HhNAC108	MEKHDDKLFVSNGALRLLPGFRFHPTDQELVLDYLKPKVLAFPLPASIIPE VHLCNSDPWDLPGDLEQERYFFSTKEVKYPNGNRSNRAAASGYWKATG PDKKILTTRAASNQVVGMKKTLVFYRGKPPHGSRTDWIMHEYRLLTPPP QGSLDNWVLCRIFLKKRSTTGNDNHVEIPRSPPHTLKANRAPSSVFYDFM ANNNRRRTTTTDLNLAPASSSSSGSSGITEASSDDPEETTSCNTLSTSTNKR TLA
HhNAC109	MQVKERYISMDERNDQAVVDKVDEVMLPGFRFHPTDEELVGFYLKRKIQ HRPLSIELIKQLDIYKYDPWDLPKLATTGEKEWYFYCPRDRKYRNSARPN RVTGAGFWKATGTDRPIYSSEGSKCIGLKKSLVFYKGRAAKGIKTDWMM HEFRLPSLTDSVPPKRFIDKNNIPANDSWAICRIFKKANSNAQRALSHSW VSPVLPDTTTTITSDMVTPHFHTTTYTDQFSSDTTRPSSLIQYGSCSNYINDL QNSSITSLSPNFGLPPYKPYNQMASTAYTFVSPETTNHHDHPTKCSIDTSS LLFNMSSSSIFGDFDKASECLDHYKGIQDQYVNNGFLVNLPQEVEENVG QGGETSVALMKEQCSNVAFFDDQWGINIRSSSVGFPYTLPMSMPEAWKS NLLCESSSPCPSDQMSTSYSTNKCNT
HhNAC110	MTITGEINSYLPPGFRFHPTDEELIVFYLRNQATSQPCPVSIIPEVDIYKFDP WELPEKAEFGENEWYFFSPRDRKYPNGARPNRAAVSGYWKATGTDKAI HSGGKYVGVKKALVFYQGKPPKGVKTDWIMHEYRLSESRSTPRKHHGS MRLDDWVLCRIYKKKNPARASEEVPKIDDFRTKTLTAGDDGSNDDDVQI MEFPRTSSISHLWELEYVGSISQLLNENSYNSSSYDNQNTMSSNGGSMAP ARNVHLGEMSHPHADLMKFQMNNQNSILYQPVFVNSMFEFQTLEIEK
HhNAC111	MAVELQFPPGFRFHPTDEELVMHYLCRKCASQQVAVPIIKEIDLYKYDPW DLPDLALYGEKEWYFFSPRDRKYPNGSRPNRAAGSGYWKATGADKPIG HPKAVGIKKALVFYSGKAPKGGKTNWIMHEYRLAGVDRSVRHKSNSLR LDDWVLCRIYNKKGTLEKKDGSRKVSHPLEIEEKKPEIMTPVLETSPAVFD DLVHLDPSDSVPRMHTDSSCSEHVVSPEITCDREVESQPKLEDWEKSALD FPFNYVDATVNNTNVNAMFGSQQGNYQMSPLQDMFMYLQRPC

HhNAC112	MARMPLPPGFRFHPTDVELIMYYLKRKVMGKQFHYFEAIAELDIYKFGP WDLPDKSVLKSKDLEWYFFCPKKKKYASGARTNRATDYGYWKTTGNDR SITYKQKTVGMIKTLVFHNGHAPKGQRTDWVMYEYRIEDENLADVVQE YMLCKIFQKSGLGPKNGAQYGAPFNEEEWSDVEELCSESSPVDGRALYH VLPSDMNNSAAVSLTVPGSSISHSISEPGPSTAGPSNFDMSNDMVDLAEE DIGALLDILTEDSTMLPVENSIAEKFNIANNDKNIEALPCLDGNEMYNDL QNLNWAELMDTGFNLSTYQDVGYAQNPMLLGDGSAFLELNDLDIPLRC HAEAGEFELVQTGSTYAPQTSYRNLDGSYCEGNYTSAVQNVSELNQHSA LPGGSHWLGDNLEVARKDNGFGSFEAHGVDTFPCNEVPVSFMQESSFAE QIQRRGEQEAKPLPRLQLLLESISAQPASAAELHAHLIRAEKLNDSISYCGS SIHFKAEVTLKVGECTKDALSHCLSHCLGESTCMCIYWGKLPIGDLRKNV TCVFYFVVIWIFMHATVQFFVDMMIMVDNCCLVFG
HhNAC113	MEWYFYSPRDRKYPNGSRTNRATRAGYWKATGKDRSVQSQKRGVGMK KTLVYYRGRAPHGIRTNWVMHEYRLTESLYRTTNAATLKDSYALCRVFK KAIHIPKSSSKEAEQINEISENNKSVYESDEHLLLTEEENGLTGISTELDYSK FPSDASSNSDVTEGTPLEGGGTTVTDCLQAPFASDEANSSADMYAYTAN FPSSNIFQDIHIPNYSSMQYEEACYPPLALEDFPQIDIAAETTSSKPSNPADE ALDDRLMYNVYASGTMMLEEILSLCSSQENSK
HhNAC114	MAPVSLPPGFRFHPTDEELVAYYLKRKINGHEIELEVIPEVDLYKCEPWDL PAKSLLPSKDLEWYFFSPRDRKYPNGSRTNRATRAGYWKATGKDRKVNS QMRAVGMKKTLVYYRGRAPHGARTDWVMHEYRLDERECETASSLQDA YALCRIFKKTLNAPKIADHYDIAASDHSSSNIEIYSEGRCEDNIESSDHHP MPFPTYPPNNNNLIMHGSPHNICGTSTDSKWMQYLSEEAFSFTNPSFQDY GAISSYPPSKVDISLECARLQHRRSLPPLQVQDFPQAGFVDLKMQPTSSMY GNTSDHDQQDILNEILSVARVSQELMDQNSWLGNNAAEDGDFSFLPNI NQNQGTSSSSRSIQIGDLDEPFKSDQRMVENLQWVGGMSNKDLEKVIDY SQTYFLDDGDLDDFSITPSFEVFDKIEINHGLLVSTRQLPETFFHQLVPSETV KVHLNSVISRNFPMPKQISPTRPKDRTLFDKPMALGTKKLVGHTGDKWN AIFTKHNLRKIRNS
HhNAC115	MEQNPVNVNGNGSGGGVGLQIQLPIGFRFRPTDEELLLHYLKPKVHSSPF PSSVIPVLDHDSIIFHSHPSHLPGDPKERRHFFYKRKRDSYVKICSRIRITTSD GSGYWKPIGKEKTIHIVAPGLLTPLVGIKKSMVFYQGKPPHGLKTPWIVH EYRLIPSQTTHNSTHQELENWVVCRINQRKRKSKNHGSNKKTTRNLEEK VVRPNNDNVMDFFMILDSSESVGPPPSSPSPSCSSGGTISFTPYGFV
HhNAC116	NASLPFANQIKDCGAQRECPKCHHLIDNSDVSHSWPGLPAGVKFDPSDV EILEHLAAKCRAGNSMPHMFIDEFIPTLYGDNGICYTHPKNLPGAKEDGS VVYFFHIPTNAYATGQRKRRKIQTEDSLTKEHVHWHKTGKTKSVMENG NQKGCKKIMPSKSNWIMHQYHLGTEEDEKDGQYVVSKIFYQQHKQPVE KDNPIAIIESDKSMIRTSPKTPKTNTPNPPRLGKSDSCDDVMANYILESPIQI PCPSSQLQFKDEIEYPSFLAGESQPADDLDSIQDSLLCNEILDSYPLYNSGL HHGPSSSFVRNTNNAPGAEKSETCGITELEDLELDTPPDFQLAVSFSSYFYS ISSFAF
HhNAC117	MDDRNEVDQIDDVMLPGFRFHPTDEELVGFYLKSKIQQRPLPIELIKQVDI YKYDPWDLPNLASTGEKECYFYCPRDRKYRNSARPNRVTGAGFWKATG TDRPIYSSNGTKCIGLKKSLVFYRGRAAKGIKTDWMMHEFRLPSIPDAGPP KKLLDKNLPPCDSWAICRIFKKANSMAHRALSHSWVSPLPKTSSNMFTQ GSHSTQFSSENTSCTAETSSAMQLCNELQQNSTASYSALDIPSYRPINPAFY KPFSLGVPNGDLHNGLVFSPPDMFGPSTKSTVDVTSMLFNMSPALIGDAS KTSDSIDFGGPQQQLNFSMSSPEDMQGSIGTGEDDAGSTKNHSATHGNN QWGNIHRSIGFPFNLTSSLSDAWKPNLPWDSPPCPSEMSTTYPQNNCYT

HhNAC118	MDNFSEEQMELPPGFRFHPTDEELITHYLTPKVDNSSFSAAAIGEVDLNK VEPWDLPWKAKMGEKEWYFFCVRDRKYPTGLRTNRATESGYWKATGK DKEIYKAKTLVGMKKILVFYKGRAPRGEKTNWVMHEYRLEGKYSMNNL PKTAKNEWVISRIFHKSSGGKKIHMSGLARLGNYDDELKPSNLPPLLDISR TTKAEPSHVTCFSEQMEDRKFQDKNVDTFNPCVLPSSSPSKPFDSSFLSPK NPITNSFFSTQISLQYPESLSIQDQSILRYFPEYNGEESEMKKNSKAEFSQET GGMSTDMSSVVSNQEMSQWSYEDQEYPITSGGPVDLDCLWNY
HhNAC119	MMSTMDTMESSCVPPGFRFHPTDEELVGYYLRKKVASQKIDLDVIGDIDL YRIEPWDLIERCRIGYEEQNEWYFFSHKDKKYPTGTRTNRATMAGFWKA TGRDKAVYDKTKLIGMRKTLVFYKGRAPNGQKSDWIMHEYRLESDENG PPQEEGWVVCRAFKKRTTTSQTKNMEAWDSTYFYDEHSTGVSSVVDPLD YITRQQPSSSFLNQSFMCKKEIEAENLIFAQSEHHQFVQLPQLESPSLPLLV KRPTSSLSLVSQNNEEEEKNRGCNNNINDSSTNNNNKVTADWRDLDKF VASQLSQDLEKKYEGVGDGILMTSFGGHELTNRSDHDDHMALLLLQSG DSDRQQGNDKLNEFFNSDSSCDIGICIFDK
HhNAC120	MENFPVKADDQMILPPGFRFHPTNEELITHYLSKKVVDSNFSATAIGEAD MNNIEPWDLPRRAKMGEKEWYFFSVRDKKYPTGLRTNRATAAGYWKA TGKDKEIFRGKSLVGMKKTLVFYKGRAPKGEKSNWVIHEYRLEGQFSLR NLPKIVKNEWVICRVFNKGSGGKKVHISGLISSTENEMAPTALPPLMDSSL HGGRTIVPSVTESIHVPCFSNTIDIQKERNNYFNNPVYPFPSNASDIFQRISL PNSYYRVQSDPIPGILQYPGAFPMQDQAILRGVVENYDSNRRQNFKTEKE MGSVSQETGLSTEMNTEISSVVSNHDMGRRSFENQEAPSTSVGALDFDRL WSY
HhNAC121	MEREVKDETLPPGFRFHPTDEELITCYLINKISDASFTGRAITDVDLNKCEP WDLPGKAKMGEKEWYFFSLRDRKYPTGVRTNRATNTGYWKTTGKDKEI FNSVTSELVGMKKTLVFYRGRAPRGEKTNWVMHEYRIHSKSSFRTTKQD EWVVCRVFQKSAGGKKYPSNNSRAVNPYNLEIGAPSAMPSQVMQSDPY HQFPMGRTYMTNTEMAELTRAFRGGSTSVNMPMPIQSQLNYPVVAGGG GGSFTISGINLNLGGGTSTQPFFRQPAPPPPPMNQQDVTSSMLTGSDIGNE AAAYAVHMNNANVLNNRFMNMEHCADLDNYWPPY
HhNAC122	MSKETEISIGSFPGFKFSPTEEELILYYLKKKIEGFDKCVEVIAEIDITQFEPW DLPAKSIIQSDNEWFFFSARGRKYPRGLQSRRATESGYWKATGKERNVKT GTNIIGTKRTLVFHKGRAPKGERTEWIMHEYCMSGNFSGNPCYRYLRILI KKKIIWQDSLVVCRLRRNCEFHFHDAPRKSKESRHLLPGDSSTSALRKESR HSLPGDSSTSALAEGLQHKGSDGSYKDRSSSDNSHSVEHMNSEYESDHKP ANEIFQDGCSSRQKGCDTADDCFADILNDDIVKLGESSLLTTPDLLPPTVP RIDVKFKEPVQRNMSGMLPSQGTANRRIKLWREKEINYLTNPEASNVGD ENFSGEADIQTQSPESPHCLMRTLSDMLANHYSIVLLLILTLLVLFLSLLAR LWQVKDFGRNFLF
HhNAC123	IVLTRSGYKKMEESPPGFRFYPTEEELVAFYLHNKLGGNYSQALARVIPIV NIYEQEPWQLPKFAGELCREDSEQWFFFVPRQEREARGGRPNRTTASGF WKATGSPSYVYSSSNKVIGVKKAMVYYIGKAPTGKKTKWKMNEYRAIVE EVLTSSSSSTSAAPRLRHEMCVCRVYVISGSCRAFDRRPLGRIIRETSSHQA DGNEDIATSSQNRSRDCRSIMTLLSSNFSLVDKNPHTNN
HhNAC124	MEIEHISSSYYPPGFRFHPSDEELIVHYLLKKLGSRPLPAAVVAEIELYNFN PWELPKKSLFGEDEWYFFTPRDRKYPKGKRPNRTAGSGYWKATGTDKPI LSASGSRSIGVKKALVFYKGRPPKGEKTDWIMTEYRLPDTMTRAARSKGT MRLDDWVLCRIRQKGNMSKNNWAGDESPSKSVDFLPEIEELPSAYTKTN TEITTDYLLKDCHILASMLACQDLPPIENISNATLPRRNYSNNSGSGFYDH

	TSGKGNAFMTSSFFESFLSPTKAKLNEGNRFDNLIGSNKMVTEIKENDILIP GNMTTTTMSFYNQNQAQGNIFNPTGRTPKLKCV
HhNAC125	MGLRDIGAALPPGFRFYPSDEELVCHYLYKKIANEEVLKGTLEEIDLHTCE PWQLPEVAKLNSNEWYFFSFRDRKYATGYRTNRATTSGYWKATGKDRT VVDPRTNGIVGMRKTLVFYKNRAPNGIKTGWIMHEFRLETPHLPPKEDW VLCRVFHKARTTTENSNQLSPQDVFEAEATAGDTSPVPHQILPLGYHHH QSPAPPQYQNQNPNNIIELSTLNPNFLQLSQVHTLANELMMIN
HhNAC126	MAGRSWLVDGNRFATKIKSASGACDPGRISWKSNPTKACPNCEHVFDN SDVTQQWPGLPRGVKFDPSDQEIIWHLLAKVGVENSKPHPFINEFIPTVD EEDGICYTHPQNLPGVMQDGSVSHFFHRAVKAYNTGTRKRRKIHGDDL GDVRWHKTGRTKPVILDGVQKGCKKIMVLYISQFRGGKAEKTNWVMH QYHLGIGEDEKEGEYVISKVFYQQQQIKQTEKSEEHSPEGIDATILKVDAIS PKSVTPELSCVERRFPDYNEGKDLTITCASSPAKMECLKDGVHTAFEDPD NQDHHATENDAYEMGDAIENQAGEDMNGWDSGSQYLLDSQQLVEAM SLCDEFLHSQSPNRNGVEGREIEGKPRLSDYARLGPENFKRDLEECQALIL DPSNIETDTPPDFRLSQLEFGSQESFVPWGAVKEA
HhNAC127	MTIIGKTNSNLPPGFRFHPTDEELIMFYLRNQATSQPCPVSIIPEVDIYKFDP WQLPEKAEFGENEWYFFSPRDRKYPNGARPNRAAVSGYWKATGTDKAI YSGSKYVGVKKALVFYKGKPPKGVKTDWIMHEYRLNGSRSTPNKQHGS MRLDDWVLCRIYKKKNTARVSEDPILEDFGTKTSTAGDDAGYDLQTMEF PRTSSISHLWELDYLGSISQLLNENSHNLSYDDQNTMTTNAAGLFEPART VELCEMSHPYYADSMKFQVNNQSSILNQPVFVNPVFEF
HhNAC128	LMSEDMNLSVDGHSRVPPGFRFHPTEEELLHYYLRKKVAYQKIDLDVIPD VDLNKLEPWDIQDKCKIGSTPQNDWYFFSHKDKKYPTGTRTNRATAAGF WKATGRDKVICSSLRRIGMRKTLVFYKGRAPHGQKSDWIMHEYRLDDN TTNHHETTTVRLYSTSAEDGWVVCRVFKKKNYHRALESPQNSSTMDSRR SHMYLTSSSKDEGVLDQILLYMGKSCKQEDHETAVVSNNNNNNITSMQSH NPINNTLAAMITSTSTSADELVHGRFVHLPRLERSTSPTIPSLLRPINHDQD CTTFKSLGPSIDEEVFSGTQANMLDDPKDGLINDWAEVDRLVASQLNGQ AETSKQLSSCCYADHPDHDNGDFCFSSFDHHDLGQLSSNPRCNRLNQA YQVYNSEIELWSFVRPSSSDPLCHLGNGPLCWSIVL
HhNAC129	MQGFNRYSENMNTFSHVPPGFRFHPTDEELVDYYLRKKVASKRIDLDVIK DVDLYKIEPWDLEELCKLGTEDQNDWYFFSHKDKKYPTGTRTNRATKA GFWKATGRDKAIYSKSSLVGMRKTLVFYKGRAPNGQKSDWIMHEYRLET DENGSPQEEGWVVCRVFKKRLTTVRKMDEHEPLCWYDDQVSFMPDFDS PRQTTQPYTPYRNHQYSCKQELELQYYTPQERFLQLPQLESPKFPQSTASL SCNSVAPYVFEQSSTLTQEEHLQQNSQALNLSALYGSNNINIEQAVDQVT DWQVLDKFVASQLGQDQDATKETNCSNAQISDHINMLCHDLKRPEVAS ESMSISALSFQIDLWK
HhNAC130	MEQDNIVTHFLPAGFRFHPTDEELIIHYLKMKVTSSSNPIVSIIADINLYKF DPWELPDKASFGENEWFFYTPRDRKYPNGARPNRATASGYWKATGCDK PILSSKGSQYIGVKKALVFYVGHPPKGTKTSWMMYEYRLPNHSIHSPRLR GSMRLDDWVLCRIRQKSNKIVAKENRKISSTSSSSSDIEPFEEKDTMENIN YFKDYGLIPQELNEVTDKFGHVNLQGQTSGNLVSDGNFNSDIEMASSVK ETLATIRRMLSLANLDEQIQFVSNESMFTHF
HhNAC131	MMAWYNESPSVDERAIVTIDSKIKTTRTLTCPSCSHSIELQDQGGVQDLPG LPAGVKFDPSDQVILEHLEAKVLYDHRHNLHPLIDEFIPTLEGENGICYTH PEKLPGVNKDGQLRHFFHRPSKAYTTGTRKRRKVNTDADDGGETRWHK TGKTRPVLVDGTVKGYKKILVLYTNYGRQKKPEKTNWIMHQYHLGDNE

	EEKDGELVVSKVFYQTQPRQCGGSSVKAIDELPNETSSKSSSSRHHDINPKIKNPGCIEFYNPNFISYNAGSQNREIPHQLIPNLTFQGDGSSFFNLSTGASEGS
HhNAC132	MAGASLPPGFRFHPTDEELVGYYLKRKIKGLEIELEVIPVIDLYKFDPWELPEKSFLPKRDMEWFFFCPRDRKYPNGSRTNRATRVGYWKATGKDRKVVCQSSVIGYRKTLVFYRGRAPAGDRTDWVMHEYRLSDDVSQGSPCSKGPFALCRIIKKNEQTLKSSDIYGDPKAKQVGSCSSNGDFSSTRTLNEPVIIPDDIPFQTNYMGSESNYSNALTSPYQTTTVVETEQAPMGTSPWVSPDFILDSSKEYPQGQEGLSGYCPHNEFPNSMTPWQPFEHFEISPSSSHSNFSEKVELIDDFSPFSSMSPYPGQGDYTGFFGHGSISHEGYGWSNSLNTNPF
HhNAC133	MDSELASSKSGSLAPGFRFHPTDEELVFYYLRRKICGKPFRFDAISEIDIYKVEPWDLPGKSRLKSRDLEWYFFSVLDKKYGNGSRTNRATDRGYWKTTGKDRAVYHRTQIVGMKKTLVYHNGRAPRGERTNWVMHEYRLIDEAMQKAGIVQDAFVLCRIFRKSGSGPKNGEHYGAPLIEEEWEDDELVLVPKDEYAEELPVSDDACPDACPDAYLDEDDLEQILNAVDPLHEDPLPLNVAYGDENHYVAESTHSFGDAQEVDPSNEAPLPLNVAYGDENHYVEESTDFIGEAQELLVGMDENNHMQEQLDGRTLFNLPVQYDMDAKSVKHEYIGEPSYTGESSNTENPVDVDYFLDESFLDAMDYPQFGDGTFIETNDLKQPAVVDPSSFDMLDEYLQFFDATDENVQHLDFDYSNMMASEDLPSDPASLLTYKDVNEGIKQETVEGKQQLEDHSNDVPSSSKQEPTKFGSDFHYAFTKHASRMLGNIPAAPAFASEFPTKDATLLHLNSASSSSIHVTAGMIQIRNVALSGSEMDLSFGKHGRVNIILSLGLPRGNDYSADLQPGSSILSDKANSAKSRGWFYFMFMWVLFLTMSFKIGSYICAN
HhNAC134	MDQELVTATPPAAIAAAPIAKSLAPGFRFHPTDEELVTYYLRRKACGKPFRFQAVSEIDVYKSEPWDLAGYSSLNSRDMEWYFFSPVDRKYGNGSRLNRATGRGYWKATGKDRLVRHKGQTIGMKKTLVFHGGRAPDGKRTNWVMHEYRLVDIELEKAGVAQDVFVLCRIFQKSGLGPPNGDRYAPFVEEEWDDDAALLVPGEAEEDVANGDDDARAGENDLEQEANKALLSLNQMPISSQTLPFVCKRERTEDCPLNCEADPESVSLFEIKRSKHSDPNSSQANGSEDSTTTSQEPTAPSTSALLEFPLLESIENRASHPPANPPTFDAANLEKSVPPGYLKFISNLENEILNVSMERETLKIEVMRAQAMINILQSRIDLLNKENEDLRKVVQGK
HhNAC135	MEFGHAEMYESPLEETEWYFFTSRNKKYPNGTRPNRSVCATTIGDDNDPGGFWKASGADTKIYSHGKLVGYKKVLVFHEGRPKNRADVKKTNWIMYEFRVADDYIIPPIRAPGDTKVCHRSSTRSLSKSPRYCHGSKYKPRYCHGSKYKPRYCHGSKYKPRYYHGSKYKPS
HhNAC136	MAGPSWLVDSHRIATKIKSASGAYDPGRVSWKSNPTKACPNCQHVVDNSDVKQQWPGLPRGVKFDPSDQEIIWHLLAKAGADNFKPHPFITEFIPTVDEDDGICYTHPRNLPGVKQDGSVSHFFHRAIKAYNTGTRKRRKIHGDDLGDVRWHKTGRTKPVILDGMQKACKKIMVLYISQVRGAKAEKTNWVMHQYHLGTGEDEKEGEYVISKVFYQQQQVKQSEQSEHLPKGIDDATLKVDSLSPKSLTPETPHAERRLPNYDEEKDLTVTCIDPHLKHYETECLKNEVHTSSEKPDNQDQLVAENDAYQLGDDDENYAGEDANGWDSASQYLLDSQQLVEAMSLCDELLRSQSPNRNGVESREIVGKACLSDYAHLGTENFKRDLEECQALVLDPANIETDTPPDFRLSQLEFGSQESFGL
HhNAC137	MSEHAWIVSKSVDKCQIEDGEGCLSFYVPPGHRFVPTDCELVEHYLLNKILRRQLPCNIIKYIDSLHKYDPEQLPVSQFTHGKKNEAYFFTHANCSYSPGAEQIKNTKNGYWKGCNGADDVICCRNQIIGYKKTYVFHQKKEPEGDEACWMMYEYRVNPNIILPQELNDIIRAERSCVICKVQYKINNALED

HhNAC138	MTSIGKTNSYLPPGFRFHPTDEELIVFYLRNQAMSKPSPVSIIPEVDIYKFDPWELPEKTEFGEKEWYFFSPRDRKYPNGARPNRAAVSGYWKATGTDKAIYSGGGKFVGVKKALVFYQGKPPKGVKTDWIMHEYRSSESRSTPKKHHGSMRLDDWVLCRIYKKKNPARASEEPKSDDVRTKTLTAGDDASNGDVQIMEFPKTSSISHLWELGYVDSISQLLNDNSYNSSSCDNKNTMSSNGGLIAPARNVQLGEMSHPRADLMKFQPNNQNSILYQPVFVNSMFEFQT
HhNAC139	MDERNDQAVADKVDEVMLPGFRFHPTDEELVGFYLKRKIQHRPLSIELIKQLDIYKYDPWDLPKLATTGEKEWYFYCPRDRKYRNSVRPNRVTGAGFWKATGTDRPIYSSECSKCIGLKKSLVFYRGRAAKGIKTDWMMHEFRLPSLTDSVLPKQFIDKNNIPANDSWAICRIFKKANAQRALSHSRVSPVLPDTTTPITSDMLTPHFHTTTCTQFSTDTTRPSSLIQYGSCSNYINDLQNSSITSLSHNFDLPPYKPYNQMASTAYTFLSPETTNHHDQPTKCIIDTSSLLSNMSSSSIIGDFDKASECLDRYKGIQDQFVNSVFLANLPQQAEENV
HhNAC140	MEKHDDKLFVKNGVLRLLPGFRFHPTDQELVLDYLKPKVLAFPLPASIIPEVHLCNSDPWDLPGDLEQERYFFSTREVKYPNGNRSNRAAASGYWKATGLDKKILTTSANSNQVAGMKKTLVFYRGKPPHGSRTDWIMHEYRLLASPPQPQGSLDNWVLCRIFLKKRGATGTKNDNHVEIPQSRPHTLKANRAPSQVFYDFMANNNHSRRRRRRTTTTDLILAPASSSSGSSGITEASSDDPEETTSCNTLSTLQINARFSKYKYPSR
HhNAC141	MEAQINQAKTMDESSIKLGLPGFRFHPTEEELLNFYLKNAILGKNSHYHVIGILNIYRHDPWFLPGLAKIGEREWYFFVPRDRTHGSGGRPNRTTEHGFWKATGSDRKIVSSSEPRRVIGLKKTLVFYKGRAPRGTKTDWIMNEYRLPPDSNIIKDNKNKDIVLCKVYRKATSLKVLEQRAAKEEEEMNMTRTNNAITNPPPPPPLPISMDHRYTITYSPSQHELDLVLKKEEDEDENEGMLIIPEDHFLNDDEAAATEETTKSTVLQLPTGMENVGLQLPKLSTQVDPFHTPLCSPWLDNWLIPYANILNL
HhNAC142	MAVSSTMNQEDNPHHDDKKGDDEHEHDMVMPGFRFHPTEEELIEFYLRRKVEGKRFNLELITFLDLYRYDPWELPALAAIGEKEWFFYVPRDRKYRNGDRPNRVTTSGYWKATGADRMIRENFRSIGLKKTLVFYSGKAPKGIRTSWIMNEYRLPHHETERLQKAEISLCRVYKRAGVEDHPSLPRFLPTKASSSSSKSDKNDQDHATQHAIQTEQTDIKIGESNNINSTSNDIGTALGLCNNNSNYNIALAPISATLLPANCATIYTGSSSLVPMPNAIDDLHRLISYQQASVNQPQFYHNTHSNMQPVPQSQSLTLSMLPGSVQAGFPDRMWEWNSITEAASKDYSSAFK
HhNAC143	MGRDDEFKVPVGFRFMPTEREIINSYLRPKIMGQKLPCNIVEEEQLYGGNPWKVLDDEKQWQVNEIDKGDLTKKTIYVFTKLTKKAGATTAGKMRKNGGNAGGKEHFVRAAGCGTWDGQTGPREIKNGNDEVIGFRRMFVFKIRDLNPNSAPTKIGHWIMHEYSLWGDESDYVLCEITRYLRREKRAVQENNKSLRIEVDPHKHEAPSHGIEHPLAIQYPAGPIQVLDLYIAMVNYDKLHISFFLHINS
HhNAC144	MVMPAGFRFCPTEEELINSYLRPKIMGQILPCNIVEEKQLYGTEDPWKVLHDGNQWMVTETDELGKIVKKIIYVFTKLTKKAITGKMKKNGGAGGKEQFVRAAGCGTWDGQTTPREVKNRNGEVIGFRKMLVFEIRGALNPSPNPSSSAESSSANPGHWIMHEYSLLGGESEYVVCEIILDLRKKSKWKRGVVEEGEENVINPKRLIKNIDEEKKDEENVIICCADVPLVHEEAGPSGMESQVLPLGVEYPNSSTTDHELIPMMESQEQALEWEWNDLVKLDELLSSDVPKFDMIMHEFEEILRHDGVMQLKDI
HhNAC145	MEEIKAMKMPTGFRFRPSEEELINYYLKPKNLGQALPCNIVQEKPLYSGNPWQVLDIRDQENKWQVSETKKVIKMTIYVFTKLTKKKHAAGAGERKKE

	AAGGGGREQFVRAAGCGTWDGQTGPKEIKNGKGGVIGFKKMLVFKIGG LNPSTSTSSVDETNNPGHWISHEYSLPGGEWEYVLCEITLHLKKSSEERKSS EDLEEIIFRKSSSPRVEFGTGNCEGDSTSQTIILSFESNDHEIKPHFTTLKFYL NNFQPNKHVIILSLLPIIFLNFSSPLYHLLKFLHSIIFLNFGNTKRGVTAHYY
HhNAC146	MEEIKAMKMPTGFRFRPSEEELINYYLKPKNLGQALPCNIVQEKPLYSGN PWQVLDIRDQENKWEQFVRAAGCGTWDGQTGPKEIKNGKGGVIGFKK MLVFKIGGLNPSTSTSSVDETNNPGHWISHEYSLPGGEWEYVLCEITLHLK KSSEERKCSEDLEEIIFRKKSSTRIEGASNGEVSAGADCNYMEVIPQPIAAIG RLYGDGGQPAAVEDDNELMLMMIEELGGSQIQQPLAGFGYDNGGQYQ ANGDGNDDNQIQQPLAAFGYVNGGQYQANDEQLMMMSHTGIWMSP GYLHIWHHLFQLATLFCLSCVELGICRLYHPFMIGFPYL
HhNAC147	MGDHNNVNLPPGFRFCPTDEELVVHFLQCKASLLPYHPNIIPDLNLYPYY PWDLDGKAMAEGNKWYFYSRRTQNRITSNGYWNPLGGDEPIFSNNTSK RVGTKKYYMFYMGDPSQGVKTNWIMEEYRLSDGASSSRSSKRRQTKVDY SKWVVCRVYDDNYDDDDGRELSCLDEVFLSLEDDFDEISFPN
HhNAC148	MPENMSISVNGQSKVPPGFRFHPTEEELLQYYLRKKVSFKNIDLDVIQDV DLNKLEPWDIQEKCKIGSTPQNDWYFFSHKDKKYPTGIRTNRATVAGFW KATGRDKVIFSNSRRIGMRKTLVFYKGRAPHGQKSDWIMHEYRLEDNTIS DTDTNTDHVSNIGVVGERTQKEGWVVCRIFKKKNHYKSLDSGYSPRMNI ISASSITTATTRTQFLTSSSSNNNSNETTGSLEQILYQYMGRSCKVDEDDAK YMNSTGTVLYERDQRFMELPNLESPNSCRSLSNYYQRPAVHVQMLNNDI QQLVSDHPNSIYDHQSQSELTDLSNWAALDQLVASHLHGQKYYDTTTT MSSSKQQLSCFNDDIDHDHPPINLWS
HhNAC149	NLGLPKACDIPPGFRFAPTEEQLVGFFLLNKVMEPSWGYFDCFIEHKNIY DHAPWNIFTDNQDWSKLNRESAVNNKKTQFYAFTKLKKKGKNVIRSSG CGKWHGQTGPKPIKYYGEIMGWEKMFTKQACIDKNESNKMFTVNNLPY FHYIVCQVK
HhNAC150	MAGPSWLVDGHRIATKIKSASGACDPGRVSWKSNPTRACPNCQHIVDN SDLTQQWPGLPRGVKFDPSDQEIIWHLLAKAGTVNFKPHPFINEFIPTVD EGDGICYTHPRNLPGVKQDGSVSHFFHRAIKAYNTGTRKRRKIHGDDLG DVRWHKTGRTKPVVLDGMQRGCKKIMVLYMSQGRGSKAEKTNWVMH QYHLGTGEDEKEGEYVISKVFYQQQQVKQSEQSEHLPEGIDDTTLKVDSF SLKSVTPEMPRAERLSPNHDEEKELTVTCIDLHLKHYETECLKDEVHTSSE KPDNQDHLVAENDADQMGDDNENYAGEDANGWDSASQYLLNSQQL VEAMSLCDELLRSQSPNRNGVESRELAGKACLSDYAYLGTENFKRDLKE CQALVLDPANIETDTPPDFRLSQLEFGSQESFGL
HhNAC151	LKSSKSKSKSFSSSTTMADYSSFSSSTDGQKKIEDGEGGYLSFYVPPGHRFVP TDCELVELYLLNKILRRQLPCNIIEYIDSLHEYDPEQLPISQFTHGKKNEAY FFTHANCSYSPGAEQIMNTKNGHWKGYNGADDAICCGNQIIGFKKTYV FHRKKEPEGEEACWMLYEYRVNPNITPPQEFNDIERSCVICKVQYKKIKH ALADSSFNREEDD
HhNAC152	MGSSNGGVPPGFRFHPTDEELLHYYLKKKISFQKFEMEVIREVDLNKIEP WDLQEKCKIGSTPQNEWYFFSHKDKKYPTGSRTNRATNAGFWKATGRD KCIRNSFKRIGMRKTLVFYRGRAPHGQKSDWIMHEYRLEDGEHDPQGNL TTSSSEDGWVICRVFKKKNLFKIGNEGGASSSASDQLNNINSATNNNQPR SLSHRENHHHQYLLNQHHNQQNFDLGLNYSHIPVALPQYPHNIQAQNF MIPTHKQPIGYDDFPTLHSEAPLLVKQLMTNPREQCDSGSENIRYQTGET GLEIGTCEPSSQNMVTGGREESLNEWGMIDRLEDPNASSMHQINQLSLR GEMDFWGYGK

HhNAC153	MEAESCVPPGFRFHPTEEELVGYYLKRKINSLKIDLDVIIDIDLYRIEPWDIQ DRCKLGYEEQNEWYFFSHKDRKYPTGKRTNRATTAGFWKATGRDKAVL SKESIIGMRKTLVFYKGRAPNGMKTDWIMHEYRLQSSDHGPPQEEGWVV CRAFKKPTPNQKQGFEPRNNGYYFQNNNNFSSPSFQEILNPLHGALTNQ STNFYQVPFANSNQQQIIPNDSRFDNETVDLPQLDSPTLSVSLDPDQENC NGWKNMDKLLSPKPVEHALFSFPNMPLVPCEDELDAQNHLNHFLGYFP EL
HhNAC154	MEENLPPGFRFHPTDEELITYYLVHKVSDFSFTSRAITDVDLNKSEPWDLP AKASMGEKEWYFFSLRDRKYPTGLRTNRATEAGYWKTTGKDKEIFRGGV LVGMKKTLVFYRGRAPKGEKTNWVMHEYRLENKHAFKPTKEEWVVCRI FQKSAMVKKPQQTTSSQQSLDSPCDTNTMVNEFGHFDHLPNFNNIANSS TMMNNISLQNYTNENNLNMSMNSLLPSSSLSWPSSLLSSNLSMNSLLLKA LQFKNYQQREITTSTNLNYASFMPQGISTPYGTDFTSNFAASSSTSKGLDN SVQQQQQEHQEQPFNLDSIW
HhNAC155	MQDGSVSHFFHRAIKAYNTGTRKRRKIHGDDLGDVRWHKTGRTKPVIL DGVQKGCKKIMVLYMSQVRGGKAEKTNWVMHQYHLGTGEDEKEGEH VISKVFYQQQQIKQTEKSEEHSPEGIDAKILQEDAICPKSVTPELSCVERRFP DYDEGKDLTITCTSSPAKVHQTSPSILTVLFYCKSKIL
HhNAC156	MTIIGKTNSNLPPGFRFHPTDEELIMFYLRNQATSQPCPGSIIPEVDIYKFDP WQLPEKAEFGENEWYFFCRRDRKYPNGARPNRAAVSGYWKATGTDKAI YSGSKFVGVKKALVFCKGKPPKGIKTDWIMHEYRLNESRSTPNTQHGSTG LDEWVLCRIYKKKNTARVSEDPIVEDCGTKTLTSNDDADNDLQMMEFPR TSSISHLWELDYLGSISQLLNESSYNSSYDDQNGLFEPAQTVELGEMSRPC YADSMKFQAKNQSSMLNLPVFVNPMFEF
HhNAC157	MDTTNCEMVIEMPIGYRFLPTDEELIKHYLSNKVFYRPLPAQAIQEIDSEK FYDKHPKTLVEDEDKFEWFFFIHENGNIYCKRKGIRMVGNGTGFWKSAQ NEDPVFNSDGDVFAFKIHLTYFSGSLNKAKKTHWKMEEYRLPTKHGDE WVLGRLKRGTDYI
HhNAC158	MGDPNNPNPSPLSLLPPGCRFFPSDEQLICCYLTNKNNNSDHGFGFDVIK EIDLYNFDPFDLPGTACFRFGRGGRKSHYYCYVARVSREEGRRRAGGGY WKRRGRVRDVVGGGAGKVVVGTRKSFVFYLGDSPKSALRTDWVMHEY ALIKHHLASFVLCRVFVKSHRNNRSEHPLSSCGEHSVATVRHIGVQHDGI NNLVTGDTNMVDNKSPDHKNKVSRFPMGLVSELDDGVTSGPGGLQMR SSELSSGREMIVDSLTAQQLVAILEEDFIELDDLLFPLPGIDQS
HhNAC159	MCPPPASPITIDLWTDEALIVLLDDIIHGHPLPSNVIADFNPYNYLPSNLLD GIGYFISSEVKKDTKLGSWKAKGDPCEIITNSDITGWRTTLEFCEGQAPDE RRTDWVMQEYRITRNGLCNNSNRNDSRTLCRLFHSVEQIPDHVTHPEHG DAFPQFDSNTSQGSTSEEKNGDDETGPPLSAAERRKCVLNGDFLELDDLA DSQSTSSISDNSSLPTFTSDEWFDSVALLRDLEDENLEQQGKGASYKYTVS ASVNPNEVIMSPVTLGMSEFHTSQFIFSYAYLMSMLFSV
HhNAC160	MDIFYHHFDSNESQLPPGFRFHPTDEELITYYLLKKVIDHNFTGRAIAQVD LNKCEPWELPEKAKMGEKEWYFYSLRDRKYPTGLRTNRATEAGYWKAT GKDREIFSSKTCCLVGMKKTLVFYRGRAPKGEKTNWVMHEFRLDGKFAY HYLCSSSKDEWVISRVFQKSGAGVTSAGGWATKRLTSGMNLYPDISSSSS ASLPQLLDSTPYAISTSGVATFTDRDSCCSDDGTVTKEHVPCFSTSAAPGSF NHHSLFDNLNPACNAVMVDPASSSRFPSLRSLNWEAPETQKPGPTELDC IWSF
HhNAC161	MTGTLPVGFRFRPTDEELINHYLNLKINGHEEKVSVIREVDVCKCEPWDL PGLSVVESVDNEWFFFCPKDRKYQNGNRTNRATVAGYWKATGRDRFIK

	ASKGMNVIGSKKTLVFHIGRAPTAQRTPWVIHEYSATEEALNGTHPGQG AFVLCRLFKKQDEMAKSSNCEEVEQYVCSPTVFKSSAEDTLSDPVSPILSQ QPEMQSISIEGIVDEIANKTIPDTPLPIADDTEVKGLDTPIQPDPYMENAFS DFYGLAEPLDSNIFSPFNLQMMNGLGSSYLHSSTTFSNDSRECSFQDVVQI L
HhNAC162	MSYSSNSNVQLPPGFRFHPSDEELVVHYLKNKATSNPLPASLMSEVELYK FTHGKKAMFGKEEWFFFTTRDRKYPNGVRPNRMAASGYWKATGTDNPI FSSHEVLIIGVKKGLVFYKGHPPRGVKTDWTMQELENWVLCRLEQKSSTP RNNREDQHSLQNDYFPRVDKLSSKNRNPNLGKVRGSLLKDCPMLPFLFD SQLNFPCMGAISSLSLESTVTSTPVHHNYSNGESFQVSLTKRQRKVVEIINE LDREKLNRENQEDELLSQSNITTDMKFYGED
HhNAC163	MNTFSHVPPGFRFHPTDEELVDYYLRKKISSRRIDLDVIKDIDLYKIEPWDL QELCRLGTEEQNEWYFFSHKDKKYPTGTRTNRATAAGFWKATGRDKAI YSKHDLVGMRKTLVFYKGRAPNGQKSDWIMHEYRLETDENATTTQEEG WVVCRVFKKRIATVRRMSEHESPIWYDDQVSFMPDMDSPKQQPNMTTY HYPYPCKKEQDFQYQIPPDHFLQLPLLESPKLLQAPPVTMTSCNSMPIYGL NMNHAKHLQSSSSVLTQEQNAGHPTYDQAVDQSVTDWRVLDKFVASQ LSHEDVLKQNHDYANVFHSDDHSNMLLIRNLNKQEMAPDQNASTSTSI CQIDLWK
HhNAC164	MTWCNDCSDESAAFVKKSPPAPITDNNVISENQKNNHIKACPSCGHQIK WQEKAGIHNLPGLPAGVKFDPNDQELLEHLEAKVRLDNHKLHPLIDEFI PTLEGENGICYAHPEKLPGVSKDGFVRHFFHRPSKAYTTGTRKRRKVHA DIDGNETRWHKTGKTRPLCTVQGKVIKGYKKILVLYTNYGKQRKPEKTN WVMHQYHLGNNDDEKEGELVVSKIFYQTQPRQCSTSSIIKDSHPTKPNN GALNIAKGGSSMKNNSTLFIDYYNQSLISFNQTT
HhNAC165	MGVPETDPLSQLSLPPGFRFYPTDEELLVQYLCRKVAGQHFSLQIIGEIDLY KFDPWVLPGKAIFGEKEWYFFSPRDRKYPNGSRPNRVAGSGYWKATGTD KVITTEGRKVGIKKALVFYVGKAPKGTKTNWIMHEYRLSDPQRKNGCAK LDDWVLCRIYKKNSSAQKPVLGEIASKEHSHSHGSSSSSSSQFDDVLESLPE IEDRFCALPRMNSLNDKLNFQYMGSGNFDWEILAGLYSIPEHVPGTQAP MQAQTQGLVNNTNQNYTCDPSIAPLGHVDTRFGKSTEEEVESGRRDQR VDNSGLLDCDPYGPGFRGSVDPFALWNPIQSRNMGFN
HhNAC166	MESTDSSAGSQQQPQLPPGFRFHPTDEELVVHYLKKKAASAPLPVAIIAE VDLYKFDPWELPAKASFGEQEWYFFSPRDRKYPNGARPNRAATSGYWK TTGTDKPVVTSAGSGGTQKVGVKKALAFYGGKPTKGIKTNWIMHEYRL ADNTKSTNMKPPGCSDVANKKGCLRLDDWVLCRIYKKNNSQRPIDHES SDMVLGSIRSPPPTCIQQIGQQNPKLLPGLKAYGTTAMQENEQNIFESGMI STNVDGSNSISGSKSHLISLPPNNVLSMKRSLYWNNDEEGKPAGGSSSTK RFFMDHINNGRTSTTADDNGSISSFLSQIPQTPRATTTQNEAAAAVMLGN LEGGDDGFFRQQAYHQLAAGMNWYS
HhNAC167	MGDHNNVNLPPGFRFCPTDEELVVHFLQRKASLLPYHPNIIPDLDLCTYD PWDLDGKAMQEGNKWYFYSRRTQNRITSNGYWNPLGGDEPIFSNSTSKR VGTKKYYMFYMGDPSQGIKTNWIMEEYKLSDGTSSKRRQTKVVIELFWSK SGLNCKWVVCRVYDHNCENDDGRELSCLDEVFLSLEDDFEEISFPN
HhNAC168	LKVKTNFHNYHHLFIIMYSLSLPSPQIRVQETFVMGIEELVNVPSGFKFDPT NEELIMYYLLPKVNGMALPCEGVVTEKVVYGENSTPWEVFDEEKDPWVS GRCKNKKTIYVFTKLKRKNKGKKQIERIAGCGTWDGQTASRIIRNGAGQI IGSRKYFSFESRKFGNENYIKFHGHWGMHEFSVSDGLKVGGGDDDYVLC AITRDDSKRILKELIISAPLVDAVQNNYRTNGEEEVVASGSINQVLEQEVA

	RGSVVHQPLDLVEVASEYGDPMLFKEDVALQQVHGINYSDDAAVNFIESHVSAMIEYNGSGNLGEFQSSAMIEYNGNFGNSSDFQVSTMIEYNGFGYPGEFQGSTTMGYGQLADLGVNNNLQHQQGEAYYWDDSFDFGNPDAIGSIDGRSEALSWENQWLGKRKLLEEAENPCRKKCCGNHN
HhNAC169	MDIEKLVPVGFKFEPTDDELITYYLLPKLYGMALPCGIVCEKVVYGENSTPWEIFDDEKDPWIQSPKCKNKKIIYVFTKLKEKSKGKKQKERTAGCGTWDGQTAPRLVNNGEGKVIGSRKYFSFEPRKIVVVEDVIQSNINDLDGDQRTNGEESASRSIYPVALEELVISASSEFHQPLEELDAPLEEVASEFDQKLLKEDTAPLQVLNEILDQKDFTDQLTFLYIYIYIYFILVKILQLESEGQCYRSLPCPWTSSSSAGNTSFPMHPSQSNPMTCDPEPVLQIGYSLIMYLCDNIPS
HhNAC170	MDIEKLVPPGFKFEPTDEELIAYYLLPKLHGMALPCGIICEKVVYGKNSTPWEIFDYEKDPWIQSPKCKNKKIIYVFTKLKEKSKGKKQKERTAGCGTWDGQTAPRLIRNGEGKVIGSRKYFSFEPRQFGNDKQDYIRNYGHWGMHEFSVSGGGDYVLCAITRDDSKRIVKELHQETPLVIQSNIISDLDSDQRTNGEESASRSVYQVALEELVISVSSEVHQPLEELEVPLEEEVASEFDPKLLKEDTTPLQVLNEVLDQYNVFGNSSESQGSTMIEYQGFGNPGELQGSTMIFENLGEFQESTIIEFNGFQNLGEFQGLAMMDYGQLGDLALNNLQNQGEGFWDDDIFDLTFLDAMPFNMEDDSFDLSFLDAMPISMDDDIFDLSFLDGISMDEVSEPLFLEKQVLGKRKFLKEDENPCGKKLCRNFLSF
HhNAC171	MNKSNHTRLEPSSISSSDLIDAKLEEHQLCGSKHCPGCGHKLEGKPDWVGLPAGVKFDPTDQELIEHLEAKVEAKEFKSHPLIDEFIPTIEGEDGICYTHPEKLPGVTRDGLNRHFFHRPSKAYTTGTRKRRKIQTECDLQGGETRWHKTGKTRPVMVNSKQKGCKKILVLYTNFGKNRKPEKTNWVMHQYHLGQHEEEREGELVVSKIFFQTQPRQCNWSDRINSTSGLVIGGTEGNINSEPNSRRESGSGSCSSKEIATHRDEVLSVVGVGAPILSFNPMEMQQFKAHDSYSFVPFGKNFDDQVGTGGVEASTARESTSLATTGLCEEHDLQRPHHHMTHQQLETAANFHISRPSHSISTIISPPPLYHTTSIILDEDSFHVSRMMLQNENFQLHQQQQQQQHHKLGGMSSSGLEELIMGCTSTDIKEESSITTPQEAEWLKYSTFWPDPHDPDNPDHHG
HhNAC172	MDQELATAVQPVAIAAPPPPTKSLAPGFRFHPTDEELVRYYLTRKACGKPFRFQAVSEIDVYKSEPWELEGYSSLNSRDLEWYFFSPVDRKYGNGSRLNRATGRGYWKATGKDRAVRHKGQTIGMKKTLVFHSGRAPDGKRTNWVMHEYRLTDIDLEKAGVAQDAFVLCRIFQKSGLGPPNGDRYAPFVEEEWDDDRALLVTGGEAEEDLTNADDARAVRNDLEQDANKAPLCLSEPPIDSQTLPFACKRERTEDCPSNREADPESVRLFASKRAKHSYPNSNHANGSEDSTTTSQEPTAPSTNFALLEFPLLDSIENRESRPPANRATFDAANLEKSVPPGYLKFISNLENEILNVSMERETLKIEVMRAQAMINILQSRIDLLNKENEDLRRFAHGK
HhNAC173	MENEPIPRSHFPPGFRFHPSDEELIVHYLLKKLGSRSLPAQVFSIIYKLIQTYYITFLSSNIYKDEWYFFTPRDRKYPKGARPNRTASSGFWKATGTDKPILGASGSKRIGVKKALVFYTGRPPRGLKTDWIMNEYRLPDTMTRSSRSEGSMRLDDWVLCRVHQKGNMSKKTWAVEDSPSKLVSFMPKIEELPSAYTASIMCGEPLNSGFMCDSFDI
HhNAC174	MEEFPPGFRFYPTEEELVSFYLHYKLQGISQAHLNRVIPVIDIYEVEPWHLPKFSGELCHKDTEQWFFFVPRQEREARGGRPNRTTASGYWKGTGSPNYVYSTCNKVIGMKKTMVFYKGKAPSGQKTKWKMNEYRAIEEEVVTSSSSSSSTNCLRHEMSVCRVYVVSGSSRAFDRRPLGTVIRQTIVDDQEAHGNGVAKCAENAPMIEKTSSPESSYSGEKDPTVDFIENEGTSNSEIVNGLGWELEQFDWQ

HhNAC175	MGLRDIGAALPPGFRFYPSDEELVCHYLFKKIANEDVLKGTDLLEIDLHT CEPWQLPELAKLNSTEWYFFSFRDRKYATGYRTNRATKTGYWKATGKD RTVVDPRTNATVGMRKTLVFYKNRAPNGIKTGWIMHEFRLENPLLPPKE DWVLCRVFHKAKTESNNIEVLSPQNYVFDAKVIAGDTSPPAAQILPLGH HHQITSNFSQNHQNQNPNNNQELLSPLNPNFLQLSEAHTESLARVGEDE YGFLFDMSFEESNLGDGIASNMEGVRFDHSLY
HhNAC176	MGCTSLPLGFRFHPTDEELVGYYLQRKIEGLEIELEVIPVIDLYKFEPWDLP EKSFLPKGDMEWLFVCPGDRKYPKGSRTDRTTRAGYWKATGKDREVVC QSSVLGYRKTLVFYRGRAPEGVRTNWVMHEYRLSDDVSQESPSSQEPFAL CRIKKKKQQAFKTGDINGDPKSKQVRSSSRNEEFTSTRISNEPVIVLDDMP FQASYMGSVSNYSSPLTSPYQTTPMMESESVPMETNRSSLWLSPDLILDLS KEYPQGQEGLSGYFPQNEFSNSITRTIVNFFRKERDLKVKLILFLAQNFIPR MRGFGG
HhNAC177	MNSINASATAQHPRLPPGFRFHPTDEELVVHYLKKKVASVPLPVTIIAEV DLYKFDPWELPSKASFGEQEWYFFSPRDRKYPNGARPNRAATSGYWKAT GTDKPILTSKGAHEKVGVKKALVFYGGKPPKGIKTNWIMHEYRLVDNSK PSMKPPCTKKNSSRLDEWVLCRIFKKKSTISSRPMERDNDDYSILEDNINF PKLGNYVGLLEDHKESIFEGTLNGNGMQKYSFMSQVEEYPSISKASDMSL ASLTRGKRTLESHDQLLWNIESGNKFYNDDNTSFNATDHGNGSFSSLLIN QFPQGSAPFQYSNTSTVLGDGLLHQPN
HhNAC178	MGGTSLPPGFRFHPTDEELVGYYLKRKIEGLEIELEVIPVIDLYKFDPWELP EKSFLPKGDMEWFFFCPRDRKYPNGSRTNRATRAGYWKATGKDRKVVC QSSVLGYRKTLVFYRGRAPGGDRTDWVMHEYRLSDDVSQESPSSQVPFA LCRIIKKNEQALKTGDINGDPKSKQVISSSRNEEFTSSRIPNEPVIIPDGMPF QASYMGSVSNYSSPLTSPYPTTPMMESESVPMETNPSSLWVSPDFILDSSKE YPQGQEGLSGYFPQNQFSNSITPWKPYDQFEISSSSSYSNFTESVELADDLG QFGCMSPYSGHGDYMTFYGNGSLPHEGYNWSNSPNRKPF
HhNAC179	MNTFSHVPPGFRFHPTDEELVDYYLRKKVASKRIDLDVIKDVDLYKIEPW DLEELCKLGTEDQNEWYFFSHKDKKYPTGTRTNRATKAGFWKATGRDK AIYSKHSLVGMRKTLVFYKGRAPNGLKSDWIMHEYRLETNENATPQEEG WVVCRVFKKRLTIVRKMDEHEPLCWYDDQVSFMPDFDSPRQIPQPYTPF HNHQYPCKQELELQYYTPHERLLELPHLESPKVPQLSAASASCNSVTPYG FEQSSTLTQEEHMHQNRQAQNINALYGSNNIEHAVDQATDWRVLDKFV ASQLSQDEDDTKEGTCSNAQVAEQINMLCNDLKRPDVDSKSTSISTLSCQ IDMWK
HhNAC180	MDEPKTSKMGLPGFRFHPTEEELLDFYLKNIILGKKKLHFDIIGVLNIYRH DPWYLPGLAKIGEREWYFFVPRDTKHGNGGRPNRTTEHGFWKATGSDR KIVSLSDPKRVIGLKKTLVFYTGRAPRGTKTDWVMNEYRLPDSCPSHKDI VLCKIYRKATSLKVLEQRAAMEDEMKTTNTNTNTTPPPSPLTPPISMPAV SFCSQNEDSKAAAASHPVFKKEEEEEEPMLTEEANFLNDEAGNITRCLNL QLPTGMEKLELQLPKFGSQDPFYAPLCSPWLDNWLIPYANVLNL
HhNAC181	MAIAFTMNLQENHDHENKICNIDEHEHDMVMPGFRFHPTEEELVEFYL RRKVEGKCFNVELITFLDLYRYDPWELPALAAIGEKEWFFYVPRDRKYRN GDRPNRVTTSGYWKATGADRMIRTENFRAIGLKKTLVFYSGKAPKGIRTS WIMNEYRLPHHETERLQKAEISLCRVYKRAGVEDHPSLHRSLPTRASSSRS NQKHQDSTQHAVEKFQTFGELHAQQINDEKISETSGNSTTDDVGTALGL SNQNIYMPLAPTNALLSPQNCNTIYNTASSSFVTMANPNNSIDDLHRLIS YQQADAPVNSQQQFYHNNSHINVQPVQQSQSLTLSMLPGSVQAAFSDK LWEWNAITEAAASTKDYGSAFK

HhNAC182	MGDNNVNLPPGFRFCPTDEELVVHFLQRKASLLPYHPDIIPDLDLYPYDP<br>WDLDGKALAEGNKWYFYSRRTQNRITSNGFWNSLGGDEPIFTTNTTRRV<br>GTKKYYAFYMGELSEGVKTNWIMQEYRLCNGGSSSKSSKRKNSKIDHSK<br>WVVCRVYEHNYDDNDEGTELSCLDEVFLSLDDDFDEISLPN

Table S3 Physicochemical properties of NAC proteins in *H. helix*.

Protein name	Number of amino acids	Molecular weight /kDa	Isoelectric point (pI)	Aliphatic index	Instability index (II)	Grand average of hydropathicity	Subcellular localization
HhNAC1	328	36.56	8.54	57.68	39.14	-0.582	Nucleus
HhNAC2	385	43.58	6.25	70.16	54.43	-0.587	Nucleus
HhNAC3	192	21.68	9.2	63.02	57.73	-0.662	Nucleus
HhNAC4	343	38.96	7.69	62.27	46.16	-0.718	Nucleus
HhNAC5	294	34.00	5.89	72.96	38.12	-0.553	Nucleus
HhNAC6	604	66.90	5.42	68.58	42.12	-0.534	Nucleus
HhNAC7	463	51.90	6.57	62.2	48.32	-0.63	Nucleus
HhNAC8	311	36.66	8.22	64.53	41.55	-0.922	Nucleus
HhNAC9	401	45.49	7.6	55.66	39.17	-0.794	Nucleus
HhNAC10	304	34.90	6.83	68.62	36.4	-0.665	Nucleus
HhNAC11	315	36.38	9.18	66.13	38.15	-0.774	Nucleus
HhNAC12	335	38.08	6.07	60.21	50.85	-0.695	Nucleus
HhNAC13	306	35.67	5.51	60.26	52.33	-0.804	Nucleus
HhNAC14	578	64.84	4.72	70	43.76	-0.534	Nucleus
HhNAC15	284	31.72	6.08	80.56	59.52	-0.38	Nucleus
HhNAC16	346	38.63	6.04	78.09	48.79	-0.58	Nucleus
HhNAC17	239	27.44	9.04	63.56	43.62	-0.748	Nucleus
HhNAC18	271	30.84	5.58	47.82	60.44	-0.866	Nucleus
HhNAC19	267	30.24	6.05	47.49	60.96	-0.894	Nucleus
HhNAC20	324	37.17	4.8	50.9	50.19	-0.896	Nucleus
HhNAC21	358	39.09	9.05	65.73	51.26	-0.317	Nucleus
HhNAC22	334	37.51	8.68	62.49	39.12	-0.643	Nucleus
HhNAC23	349	38.74	8.73	57.34	41.48	-0.732	Nucleus
HhNAC24	634	70.22	5.24	75.36	41.8	-0.454	Nucleus
HhNAC25	367	42.18	6.96	72.32	44.51	-0.696	Nucleus
HhNAC26	456	51.09	6.32	60.15	42.84	-0.638	Nucleus
HhNAC27	402	45.39	8.49	56.99	36.34	-0.754	Nucleus
HhNAC28	312	35.90	6.67	63.43	40.66	-0.818	Nucleus
HhNAC29	336	37.86	4.95	61.04	41.59	-0.712	Nucleus
HhNAC30	317	36.51	8.92	67.57	40.73	-0.735	Nucleus
HhNAC31	334	38.19	8.09	59.52	54.19	-0.711	Nucleus
HhNAC32	297	34.49	6.03	55.86	46.16	-0.846	Nucleus
HhNAC33	284	32.02	5.93	79.15	56.87	-0.364	Nucleus
HhNAC34	343	37.76	5.5	82.16	42.55	-0.401	Nucleus
HhNAC35	270	31.01	5.51	45.48	53.18	-0.879	Nucleus
HhNAC36	347	39.62	5.25	56.83	44.1	-0.939	Nucleus
HhNAC37	345	38.33	7.69	60.26	46	-0.531	Nucleus
HhNAC38	340	38.26	8.29	62.21	34.29	-0.655	Nucleus

Protein name	Number of amino acids	Molecular weight /kDa	Isoelectric point (pI)	Aliphatic index	Instability index (II)	Grand average of hydropathicity	Subcellular localization
HhNAC39	355	39.53	8.76	58	34.74	-0.639	Nucleus
HhNAC40	350	39.85	9.18	68.86	41.76	-0.636	Nucleus
HhNAC41	269	30.91	4.8	70.67	36.87	-0.567	Nucleus
HhNAC42	244	27.81	9.02	60.7	53.11	-0.638	Nucleus
HhNAC43	615	70.29	5.64	65.28	44.35	-0.66	Nucleus
HhNAC44	291	33.48	6.26	65.67	40.03	-0.728	Nucleus
HhNAC45	373	41.83	5.54	68.79	55.4	-0.654	Nucleus
HhNAC46	397	44.63	6.32	57.98	43.06	-0.625	Nucleus
HhNAC47	326	37.10	6.47	74.14	41.34	-0.592	Nucleus
HhNAC48	351	40.62	5.42	66.95	55.69	-0.753	Nucleus
HhNAC49	348	39.46	8.75	68.05	36.86	-0.568	Nucleus
HhNAC50	230	25.92	8.89	59.7	42.39	-0.602	Nucleus
HhNAC51	374	42.24	6.41	55.78	32.41	-0.844	Nucleus
HhNAC52	330	38.04	7.33	62.88	36.74	-0.763	Nucleus
HhNAC53	319	36.94	7.57	61.44	44.52	-0.756	Nucleus
HhNAC54	334	38.05	8.52	57.75	40.1	-0.707	Nucleus
HhNAC55	478	54.42	4.52	65.08	43.84	-0.689	Nucleus
HhNAC56	353	38.70	5.08	76.8	40.27	-0.561	Nucleus
HhNAC57	173	20.38	9.07	63.7	38.68	-0.645	Nucleus
HhNAC58	559	61.54	4.94	79.53	36.57	-0.367	Nucleus
HhNAC59	269	30.62	9.21	73.68	55.35	-0.608	Nucleus
HhNAC60	350	40.88	6.39	61.83	57.26	-0.799	Nucleus
HhNAC61	269	30.47	9.03	71.34	40.13	-0.771	Nucleus
HhNAC62	321	36.28	7.06	68.04	32.78	-0.61	Nucleus
HhNAC63	385	42.21	8.74	65.4	44.9	-0.586	Nucleus
HhNAC64	235	27.07	5	68.04	42.58	-0.691	Nucleus
HhNAC65	292	33.41	7.04	63.77	45.77	-0.714	Nucleus
HhNAC66	657	75.18	5.68	70.61	50.2	-0.532	Nucleus
HhNAC67	252	28.42	8.81	60.36	50.42	-0.565	Nucleus
HhNAC68	389	43.58	6.21	65.68	51.03	-0.64	Nucleus
HhNAC69	328	37.12	6.68	67.77	45.21	-0.605	Nucleus
HhNAC70	358	41.31	5.43	61.82	55.21	-0.796	Nucleus
HhNAC71	343	38.78	6.62	73.29	39.52	-0.448	Nucleus
HhNAC72	651	72.41	5.39	92.63	48.38	-0.159	Nucleus
HhNAC73	295	34.31	8.01	76.64	42.74	-0.237	Nucleus
HhNAC74	317	36.22	7.74	60.88	39.86	-0.69	Nucleus
HhNAC75	300	34.92	5.79	62.4	44.5	-0.76	Nucleus
HhNAC76	240	27.38	8.17	73.92	38.81	-0.634	Nucleus
HhNAC77	455	51.53	4.89	69.21	35.79	-0.601	Nucleus

Protein name	Number of amino acids	Molecular weight /kDa	Isoelectric point (pI)	Aliphatic index	Instability index (II)	Grand average of hydropathicity	Subcellular localization
HhNAC78	201	23.18	10.2	74.58	41.08	-0.673	Nucleus
HhNAC79	391	44.10	5.34	67.08	47.1	-0.744	Nucleus
HhNAC80	372	42.58	9.09	66.85	34.64	-0.556	Nucleus
HhNAC81	338	39.18	6.28	54.47	44.04	-0.81	Nucleus
HhNAC82	375	43.12	5.22	63.15	51.82	-0.776	Nucleus
HhNAC83	352	40.11	8.56	68.41	47.2	-0.621	Nucleus
HhNAC84	458	51.35	6.29	61.29	45.11	-0.819	Nucleus
HhNAC85	405	45.48	5.23	73.75	52.24	-0.633	Nucleus
HhNAC86	347	39.20	9.03	64.12	37.58	-0.584	Nucleus
HhNAC87	257	29.47	5.56	59.84	56.53	-0.628	Nucleus
HhNAC88	400	45.06	5.76	65.07	56.35	-0.612	Nucleus
HhNAC89	275	31.48	5.61	69.53	33.23	-0.624	Nucleus
HhNAC90	199	23.08	4.81	66.13	65.68	-0.737	Nucleus
HhNAC91	323	37.32	6.24	72.17	47.71	-0.766	Nucleus
HhNAC92	189	21.82	4.63	67.51	43.18	-0.711	Nucleus
HhNAC93	295	33.76	5.96	74.71	54.54	-0.611	Nucleus
HhNAC94	247	28.37	8.67	66.68	50.33	-0.861	Nucleus
HhNAC95	315	35.73	9.53	62.54	38.36	-0.807	Nucleus
HhNAC96	399	46.08	6.68	61.5	40.07	-0.561	Nucleus
HhNAC97	365	42.83	8.35	66.74	42.55	-0.852	Nucleus
HhNAC98	579	64.78	4.58	71.61	41.75	-0.444	Nucleus
HhNAC99	309	36.05	7.7	62.78	44.92	-0.808	Nucleus
HhNAC100	372	42.17	7.65	60.27	33.34	-0.774	Nucleus
HhNAC101	424	48.15	6.1	65.5	46.18	-0.709	Nucleus
HhNAC102	351	41.05	5.74	63.56	48.45	-0.811	Nucleus
HhNAC103	301	33.90	6.73	67.34	47.13	-0.762	Nucleus
HhNAC104	344	38.62	6.32	51.54	43.06	-0.706	Nucleus
HhNAC105	597	67.38	4.56	67.47	40.31	-0.595	Nucleus
HhNAC106	283	32.17	6.4	73.43	38.09	-0.49	Nucleus
HhNAC107	470	53.15	6.37	59.51	51.01	-0.945	Nucleus
HhNAC108	253	28.28	9.32	66.72	43.42	-0.666	Nucleus
HhNAC109	426	48.32	6.56	61.78	42.35	-0.591	Nucleus
HhNAC110	295	33.85	6.16	61.8	36.56	-0.719	Nucleus
HhNAC111	289	33.09	6.51	67.44	50.77	-0.639	Nucleus
HhNAC112	577	64.59	4.84	76.24	36.93	-0.297	Nucleus
HhNAC113	283	31.78	5.03	60.07	48.56	-0.73	Nucleus
HhNAC114	512	58.50	5.96	68.18	54.15	-0.687	Nucleus
HhNAC115	250	28.14	9.75	72.76	50.73	-0.588	Nucleus
HhNAC116	358	40.05	5.5	65.67	49.82	-0.645	Nucleus

Protein name	Number of amino acids	Molecular weight /kDa	Isoelectric point (pI)	Aliphatic index	Instability index (II)	Grand average of hydropathicity	Subcellular localization
HhNAC117	399	44.36	6.75	57.24	43.23	-0.613	Nucleus
HhNAC118	342	39.31	5.82	56.43	47.44	-0.794	Nucleus
HhNAC119	374	43.03	5.2	62.54	49.03	-0.802	Nucleus
HhNAC120	353	40.08	8.95	67.08	41.46	-0.608	Nucleus
HhNAC121	330	37.11	8.88	53.48	35.94	-0.66	Nucleus
HhNAC122	420	47.88	8	75	51.97	-0.575	Nucleus
HhNAC123	240	27.27	9.51	65	56.74	-0.602	Nucleus
HhNAC124	336	38.05	9.11	63.9	50.05	-0.654	Nucleus
HhNAC125	240	27.59	6.8	72.33	40.88	-0.617	Nucleus
HhNAC126	427	47.97	5.17	65.29	48.9	-0.735	Nucleus
HhNAC127	286	32.73	6.32	63.08	35.63	-0.637	Nucleus
HhNAC128	430	48.92	6.2	68	43.41	-0.69	Nucleus
HhNAC129	360	41.99	5.57	63.08	52.85	-0.812	Nucleus
HhNAC130	282	32.31	7.1	70.18	36.81	-0.574	Nucleus
HhNAC131	303	34.08	6.92	67.19	43.45	-0.795	Nucleus
HhNAC132	342	38.66	5.59	54.12	42.1	-0.665	Nucleus
HhNAC133	607	68.44	4.56	70.07	42.56	-0.557	Nucleus
HhNAC134	397	44.34	5.1	74.01	49.34	-0.598	Nucleus
HhNAC135	171	19.91	9.69	38.77	29.72	-1.1	Nucleus
HhNAC136	422	47.56	5.35	69.31	50.33	-0.792	Nucleus
HhNAC137	192	22.32	6.38	75.62	51.24	-0.549	Nucleus
HhNAC138	289	32.82	8.95	59.03	41.3	-0.729	Nucleus
HhNAC139	338	38.61	8.03	69.26	39.53	-0.571	Nucleus
HhNAC140	268	30.35	9.73	67.72	50.94	-0.721	Nucleus
HhNAC141	312	35.80	6.23	79.39	42.73	-0.645	Nucleus
HhNAC142	352	40.12	6.5	66.56	43.37	-0.723	Nucleus
HhNAC143	249	28.60	8.61	75.94	37.76	-0.623	Nucleus
HhNAC144	314	35.55	5.01	83.12	49.21	-0.46	Nucleus
HhNAC145	305	34.52	9.04	77.02	36.71	-0.482	Nucleus
HhNAC146	332	37.06	4.93	74.34	41.61	-0.417	Nucleus
HhNAC147	189	22.12	4.88	58.73	55.59	-0.809	Nucleus
HhNAC148	373	42.99	6.67	64.77	47.03	-0.813	Nucleus
HhNAC149	155	18.10	9.24	59.74	58.78	-0.601	Nucleus
HhNAC150	422	47.48	5.41	66.99	47.89	-0.78	Nucleus
HhNAC151	216	24.76	6	60.51	52.01	-0.726	Nucleus
HhNAC152	354	40.70	7	57.85	38.15	-0.947	Nucleus
HhNAC153	299	34.73	5.67	63.58	42.67	-0.795	Nucleus
HhNAC154	318	36.48	7.75	59.18	40.79	-0.715	Nucleus
HhNAC155	185	21.06	8.88	70	60.16	-0.709	Nucleus

Protein name	Number of amino acids	Molecular weight /kDa	Isoelectric point (pI)	Aliphatic index	Instability index (II)	Grand average of hydropathicity	Subcellular localization
HhNAC156	279	31.90	5.69	60.82	32.39	-0.646	Nucleus
HhNAC157	161	19.09	6.31	64.16	36.69	-0.685	Nucleus
HhNAC158	287	31.87	7.16	75.3	55.03	-0.418	Nucleus
HhNAC159	292	32.60	4.38	73.46	39.03	-0.472	Nucleus
HhNAC160	303	34.13	7.09	58.94	41.14	-0.52	Nucleus
HhNAC161	302	33.82	4.9	72.91	39.11	-0.413	Nucleus
HhNAC162	280	32.22	8.81	66.07	37.22	-0.735	Nucleus
HhNAC163	349	40.85	6.2	64.24	53.25	-0.802	Nucleus
HhNAC164	279	31.60	9.16	66.74	36.26	-0.825	Nucleus
HhNAC165	336	37.76	6.67	66.43	33.26	-0.62	Nucleus
HhNAC166	371	40.65	8.95	59.49	37.07	-0.673	Nucleus
HhNAC167	195	22.80	5.13	63.9	54.23	-0.704	Nucleus
HhNAC168	433	48.60	5.11	73.79	31.77	-0.478	Nucleus
HhNAC169	299	33.94	4.66	86.02	48.35	-0.351	Nucleus
HhNAC170	432	49.13	4.48	77.36	38.29	-0.476	Nucleus
HhNAC171	462	52.02	6.15	61.39	46.05	-0.817	Nucleus
HhNAC172	399	45.00	5.6	68.3	50.29	-0.731	Nucleus
HhNAC173	217	24.91	9.51	66.96	45.16	-0.492	Nucleus
HhNAC174	253	28.81	5.77	58.1	58.24	-0.664	Nucleus
HhNAC175	277	31.68	5.94	73.25	38.06	-0.57	Nucleus
HhNAC176	310	35.63	8.2	68.16	48.31	-0.598	Nucleus
HhNAC177	326	36.78	8.91	65.21	33.14	-0.689	Nucleus
HhNAC178	345	39.09	5.23	54.2	52.03	-0.699	Nucleus
HhNAC179	349	40.70	5.83	64.24	43.15	-0.822	Nucleus
HhNAC180	293	33.41	7.05	73.24	46.47	-0.573	Nucleus
HhNAC181	368	42.11	6.43	67.91	44.77	-0.677	Nucleus
HhNAC182	189	21.92	4.91	65.45	41.84	-0.752	Nucleus

Table S4 The protein sequences of NAC family in *H. helix*, *A. thaliana*, and *O. sativa*.

Name	Sequences
AT2G27300.1	MSKEAEMSIAVSALFPGFRFSPTDVELISYYLRRKIDGDENSVAVIAE VEIYKFEPWDLPEESKLKSENEWFYFCARGRKYPHGSQSRRATQLG YWKATGKERSVKSGNQVVGTKRTLVFHIGRAPRGERTEWIMHEYC IHGAPQDALVVCRLRKNADFRASSTQKMEDGVVQDDGYVGQRGC LEKEDKSYYESEHQIPNGDIAESSNVVEDQADTDDDCYAEILNDDII KLDEEALKASQAFRPTNPTHQETISSESSSKRSKCGIKKESTETMNCY ALFRIKNVAGTDSSWRFPNPFKIKKDDSQRLMKNVLATTVFLAILFS FFWTVLIARN
AT1G02220.1	METPVGLRFCPTDEEIVVDYLWPKNSDRDTSHVDRFINTVPVCRL DPWELPCQSRIKLKDVAWCFFRPKENKYGRGDQQMRKTKSGFW KSTGRPKPIMRNRQQIGEKKILMFYTSKESKSDWVIHEYHGFSHN QMMMTYTLCKVMFNGGMREKSSSSPSSSGVSGIEQSRRDSLIPQL VNNSEGSSLHREDPSQFGDVLQEAPIEDAKLTEELVKWLMNDED DAQIEDAIPIEEWETWLNDIDDAKEKSIMFMHDNRSDYRPPNSLT GVFSDDVSSDDNDSDLLTPKTNSIQTSSTCDSFGSSNHRIDQIKDL QESPTSTINLVSLTQEVSQALITSIDTAEKKKNPYDDAQGTEIGEHK LGQETIKKKRAGFFHRMIQKFVKKIHLCSSISRT
AT3G04420.2	MENPVGLRFRPTDKEIVVDYLRPKNSDRDTSHVDRVISTVTIRSFD PWELPCQSRIKLKDESWCFFSPKENKYGRGDQQIRKTKSGYWKIT GKPKPILRNRQEIGEKKVLMFYMSKELGGSKSDWVMHEYHAFSP TQMMMTYTICKVMFKGDVREISSSSASYGSEIEQSRRDSLIPLLVNDS EEEAQIEDAIPIEEWETWLTDDGVDEQVNHIMNMKDDRNNHRP QKPLTGVLIDDSSDDDDDSDLLSPTTNSIENSSTCDSFGSSDQINLV SLTQEVSFILSLFFSDTNNP
AT3G04420.1	MENPVGLRFRPTDKEIVVDYLRPKNSDRDTSHVDRVISTVTIRSFD PWELPCQSRIKLKDESWCFFSPKENKYGRGDQQIRKTKSGYWKIT GKPKPILRNRQEIGEKKVLMFYMSKELGGSKSDWVMHEYHAFSP TQMMMTYTICKVMFKGDVREISSSSASYGSEIEQSRRDSLIPLLVNDS EEEAQIEDAIPIEEWETWLTDDGVDEQVNHIMNMKDDRNNHRP QKPLTGVLIDDSSDDDDDSDLLSPTTNSIENSSTCDSFGSSDQINLV SLTQEVSQALITSIDTPEKIKSPYDDAQGTGAGGQKLGQETREKKR AGFFHRMIQIFVKKIHQCSSISRT

AT1G28520.5	MTGKRSKTNCRSASHKLFKDKAKNRVDDLQGMLLDLQFARKESRPTDVTLLEEQVNQMLREWKSELNEPSPASSLQQGGTLGSFSSDICRLLQLCDEEDDATSKLAAPKPEPADQNLEAGKAAVFQRGYNLVQGKSEHGLPLVDNCKDLSLAAGNNFDGTAPLEYHQQYDLQQEFEPNFNGGFNNCPSYGVVEGPIHISNFIPTICPPPSAFLGPKCALWDCPRPAQGFDWFQDYCSSFHAALAFNEGPPGMNPVVRPGGIGLKDGLLFAALSAKAGGKDVGIPECEGAATAKSPWNAPELFDLTVLESETLREWLFFDKPRRAFESGNRKQRSLPDYNGRGWHESRKQIMVEFGGLKRSYYMDPQPLHHFEWHLYEYEINKCDACALYRLELKLVDGKKTSKGKVSNDSVADLQKQMGRLTAEFPPENNTTNTTNNNKRCIKGRPKVSTKVATGNVQNTVEQANDYGVGEEFNYLVGNLSDYYIP
AT1G28520.2	MTGKRSKTNCRSASHKLFKDKAKNRVDDLQGMLLDLQFARKESRPTDVTLLEEQVNQMLREWKSELNEPSPASSLQQGGTLGSFSSDICRLLQLCDEEDDATSKLAAPKPEPADQNLEAGKAAVFQRGYNLVQGKSEHGLPLVDNCKDLSLAAGNNFDGTAPLEYHQQYDLQQEFEPNFNGGFNNCPSYGVVEGPIHISNFIPTICPPPSAFLGPKCALWDCPRPAQGFDWFQDYCSSFHAALAFNEGPPGMNPVVRPGGIGLKDGLLFAALSAKAGGKDVGIPECEGAATAKSPWNAPELFDLTVLESETLREWLFFDKPRRAFESGNRKQRSLPDYNGRGWHESRKQIMVEFGGLKRSYYMDPQPLHHFEWHLYEYEINKCDACALYRLELKLVDGKKTSKGKVSNDSVADLQKQMGRLTAEFPPENNTTNTTNNNKRCIKGRPKVSTKVATGNVQNTVEQANDYGVGEEFNYLVGNLSDYYIP
AT1G28520.1	MTGKRSKTNCRSASHKLFKDKAKNRVDDLQGMLLDLQFARKESRPTDVTLLEEQVNQMLREWKSELNEPSPASSLQQGGTLGSFSSDICRLLQLCDEEDDATSKLAAPKPEPADQNLEAGKAAVFQRGYNLVQGKSEHGLPLVDNCKDLSLAAGNNFDGTAPLEYHQQYDLQQEFEPNFNGGFNNCPSYGVVEGPIHISNFIPTICPPPSAFLGPKCALWDCPRPAQGFDWFQDYCSSFHAALAFNEGPPGMNPVVRPGGIGLKDGLLFAALSAKAGGKDVGIPECEGAATAKSPWNAPELFDLTVLESETLREWLFFDKPRRAFESGNRKQRSLPDYNGRGWHESRKQIMVEFGGLKRSYYMDPQPLHHFEWHLYEYEINKCDACALYRLELKLVDGKKTSKGKVSNDSVADLQKQMGRLTAEFPPENNTTNTTNNNKRCIKGRPKVSTKVATGNVQNTVEQANDYGVGEEFNYLVGNLSDYYIP

AT1G28520.4	MTGKRSKTNCRSASHKLFKDKAKNRVDDLQGMLLDLQFARKES RPTDVTLLEEQVNQMLREWKSELNEPSPASSLQQGGTLGSFSSDIC RLLQLCDEEDDATSKLAAPKPEPADQNLEAGKAAVFQRGYNLV QGKSEHGLPLVDNCKDLSLAAGNNFDGTAPLEYHQQYDLQQEF EPNFNGGFNNCPSYGVVEGPIHISNFIPTICPPPSAFLGPKCALWD CPRPAQGFDWFQDYCSSFHAALAFNEGPPGMNPVVRPGGIGLK DGLLFAALSAKAGGKDVGIPECEGAATAKSPWNAPELFDLTVLE SETLREWLFFDKPRRAFESGNRKQRSLPDYNGRGWHESRKQIMV EFGGLKRSYYMDPQPLHHFEWHLYEYEINKCDACALYRLELKLV DGKKTSKGKVSNDSVADLQKQMGRLTAEFPPENNTTNTTNNNK RCIKGRPKVSTKVATGNVQNTVEQANDYGVGEEFNYLVGNLSD YYIP
AT1G28520.3	MTGKRSKTNCRSASHKLFKDKAKNRVDDLQGMLLDLQFARKES RPTDVTLLEEQVNQMLREWKSELNEPSPASSLQQLCDEEDDATSK LAAPKPEPADQNLEAGKAAVFQRGYNLVQGKSEHGLPLVDNCK DLSLAAGNNFDGTAPLEYHQQYDLQQEFEPNFNGGFNNCPSYG VVEGPIHISNFIPTICPPPSAFLGPKCALWDCPRPAQGFDWFQDYC SSFHAALAFNEGPPGMNPVVRPGGIGLKDGLLFAALSAKAGGKD VGIPECEGAATAKSPWNAPELFDLTVLESETLREWLFFDKPRRAF ESGNRKQRSLPDYNGRGWHESRKQIMVEFGGLKRSYYMDPQPL HHFEWHLYEYEINKCDACALYRLELKLVDGKKTSKGKVSNDSVA DLQKQMGRLTAEFPPENNTTNTTNNNKRCIKGRPKVSTKVATG NVQNTVEQANDYGVGEEFNYLVGNLSDYYIP
AT5G07680.2	MDLPPGFRFHPTDEELITHYLHKKVLDLGFSAKAIGEVDLNKAEP WELPYKAKIGEKEWYFFCVRDRKYPTGLRTNRATQAGYWKATG KDKEIFRGKSLVGMKKTLVFYRGRAPKGQKTNWVMHEYRLDGK LSAHNLPKTAKNEWVICRVFHKTAGGKKIPISTLIRIGSYGTGSSLP PLTDSSPYNDKTKTEPVYVPCFSNQAETRGTILNCFSNPSLSSIQPD FLQMIPLYQPQSLNISESSNPVLTQEQSVLQAMMENNRRQNFKTL SISQETGVSNTDNSSVFEFGRKRFDHQEVPSPSSGPVDLEPFWNY
AT5G07680.1	METFGVFHKEDDEQMDLPPGFRFHPTDEELITHYLHKKVLDLGFS AKAIGEVDLNKAEPWELPYKAKIGEKEWYFFCVRDRKYPTGLRT NRATQAGYWKATGKDKEIFRGKSLVGMKKTLVFYRGRAPKGQK TNWVMHEYRLDGKLSAHNLPKTAKNEWVICRVFHKTAGGKKIP ISTLIRIGSYGTGSSLPPLTDSSPYNDKTKTEPVYVPCFSNQAETRGT ILNCFSNPSLSSIQPDFLQMIPLYQPQSLNISESSNPVLTQEQSVLQA MMENNRRQNFKTLSISQETGVSNTDNSSVFEFGRKRFDHQEVPSP SSGPVDLEPFWNY

AT5G66300.1	MMKVDQDYSCSIPPGFRFHPTDEELVGYYLKKKIASQRIDLDVIRE<br>IDLYKIEPWDLQERCRIGYEEQTEWYFFSHRDKKYPTGTRTNRAT<br>VAGFWKATGRDKAVYLNSKLIGMRKTLVFYRGRAPNGQKSDWII<br>HEYYSLESHQNSPPQEEGWVVCRAFKKRTTIPTKRRQLWDPNCL<br>FYDDATLLEPLDKRARHNPDFTATPFKQELLSEASHVQDGDFGS<br>MYLQCIDDDQFSQLPQLESPSLPSEITPHSTTFSENSSRKDDMSSEK<br>RITDWRYLDKFVASQFLMSGED
AT3G12977.1	MEETEKNKGSISMVEANLPPGFRFHPRDDELVCDYLMRRTVRSLY<br>QPVVLIDVDLNKCEPWDIPQTARVGGKEWYFYSQKDRKYATGYR<br>TNRATATGYWKATGKDRAIQRNGGLVGMRKTLVFYRGRSPKGR<br>KTDWVMHEFRLQGKLLHHSPNSLEEEWVLCRVFHKNSNGADID<br>DITRSCSDATASAFMDSYINFDHHHIINQHVPCFSNNLSHNQTNQ<br>SGLISKNSSPLFNASPDQMILRTLLSQLTKKVEESQSRGDGSSESQL<br>TDIGIPSHAWNY
AT1G62700.2	MNSFSQVPPGFRFHPTDEELVDYYLRKKVASKRIEIDIIKDVDLYKI<br>EPCDLQELCKIGNEEQSEWYFFSHKDKKYPTGTRTNRATKAGFW<br>KATGRDKAIYIRHSLIGMRKTLVFYKGRAPNGQKSDWIMHEYRL<br>ETSENGTPQEEGWVVCRVFKKKLAATVRKMGDYHSSPSQHWYD<br>DQLSFMASEIISSSPRQFLPNHHYNRHHHQQTLPCGLNAFNNNN<br>PNLQCKQELELHYNQMVQHQQQNHHLRESMFLQLPQLESPTSN<br>CNSDNNNNTRNISNLQKSSNISHEEQLQQGNQSFSSLYYDQGVE<br>QMTTDWRVLDKFVASQLSNDEEAAAVVSSSSHQNNVKIDTRNT<br>GYHVIDEGINLPENDSERVVEMGEEYSNAHAASTSSSCQIDL
AT1G62700.1	MNSFSQVPPGFRFHPTDEELVDYYLRKKVASKRIEIDIIKDVDLYKI<br>EPCDLQELCKIGNEEQSEWYFFSHKDKKYPTGTRTNRATKAGFW<br>KATGRDKAIYIRHSLIGMRKTLVFYKGRAPNGQKSDWIMHEYRL<br>ETSENGTPQEEGWVVCRVFKKKLAATVRKMGDYHSSPSQHWYD<br>DQLSFMASEIISSSPRQFLPNHHYNRHHHQQTLPCGLNAFNNNN<br>PNLQCKQELELHYNQMVQHQQQNHHLRESMFLQLPQLESPTSN<br>CNSDNNNNTRNISNLQKSSNISHEEQLQQGNQSFSSLYYDQGVE<br>QMTTDWRVLDKFVASQLSNDEEAAAVVSSSSHQNNVKIDTRNT<br>GYHVIDEGINLPENDSERVVEMGEEYSNAHAASTSSSCQIDL
AT5G09330.1	MGKTQLAPGFRFHPTDVELVRYYLKRKILGKKLLVDAIAEVDIYK<br>FEPPDLPDMSFIRSGDLKWHFFCPREKKYASGVRANRATECGYW<br>KTTGKERPVLCNSEVVGKIKTLVYHFGKSPRGERTDWVMHEYRL<br>DDKVLTQMNVPQDTYVVCVLFKKDGPGPRNGAQYGAPFKEED<br>WSDEEVRTDVPSTSNPTNLLEPSKETTLALTAPDDSNKDCFGGMI<br>SESCVSDFLPATTNTTSELPHPSDAATTPMSTAPLAETVQTPNND<br>DLYSMLDLFDDDEEFLGFNNNEVRYDPGVSAPVCLEEEGIFNGLP<br>ELSSMPRTASYDLVENSELYLELQDLTAPLNPQTGLQDLTAPFNP<br>QTGLQDLTAPFNHQTGLQDHTAPFNPQTGLQDHTAPFNHQTGL<br>QDLTAPFNPQTGLQDLTAPFNPQTGLHDLTSPFNPQTGLQDLTA<br>PLNPQTGNRNDPRSSSFLYNQGHFDFSGGNDDDPYGFSASMRHR<br>PKM

AT5G09330.3	MGKTQLAPGFRFHPTDVELVRYYLKRKILGKKLLVDAIAEVDIYK FEPPDLPDMSFIRSGDLKWHFFCPREKKYASGVRANRATECGYW KTTGKERPVLCNSEVVGKIKTLVYHFGKSPRGERTDWVMHEYRL DDKVLTQMNVPQDTYVVCVLFKKDGPGPRNGAQYGAPFKEED WSDEEVRTDVPSTSNPTNLLEPSKETTLALTAPDDSNKDCFGGMI SESCVSDFLPATTNTTSELPHPSDAATTPMSTAPLAETVQTPNND DLYSMLDLFDDDEEFLGFNNNEVRYDPGVSAPVCLEEEGIFNGLP ELSSMPRTASYDLVENSELYLELQDLTAPLNPQTGLQDLTAPFNP QTGLQDLTAPFNHQTGLQDHTAPFNPQTGLQDHTAPFNHQTGL QDLTAPFNPQTGLQDLTAPFNPQTGLHDLTSPFNPQTGLQDLTA PLNPQTGNRNDPRSSSFLYNQGHFDFSGGNDDDPYGFSASMRHR PKM
AT5G09330.4	MGKTQLAPGFRFHPTDVELVRYYLKRKILGKKLLVDAIAEVDIYK FEPPDLPDMSFIRSGDLKWHFFCPREKKYASGVRANRATECGYW KTTGKERPVLCNSEVVGKIKTLVYHFGKSPRGERTDWVMHEYRL DDKVLTQMNVPQDTYVVCVLFKKDGPGPRNGAQYGAPFKEED WSDEEVRTDVPSTSNPTNLLEPSKETTLALTAPDDSNKDCFGGMI SESCVSDFLPATTNTTSELPHPSDAATTPMSTAPLAETVQTPNND DLYSMLDLFDDDEEFLGFNNNEVRYDPGVSAPVCLEEEGIFNGLP ELSSMPRTASYDLVENSELYLELQDLTAPLNPQTGLQDLTAPFNP QTGLQDLTAPFNHQTGLQDHTAPFNPQTGLQDHTAPFNHQTGL QDLTAPFNPQTGLQDLTAPFNPQTGLHDLTSPFNPQTGLQDLTA PLNPQTGNRNDPRSSSFLYNQGHFDFSGGNDDDPYGFSASMRHR PKM
AT5G09330.2	MGKTQLAPGFRFHPTDVELVRYYLKRKILGKKLLVDAIAEVDIYK FEPPDLPDMSFIRSGDLKWHFFCPREKKYASGVRANRATECGYW KTTGKERPVLCNSEVVGKIKTLVYHFGKSPRGERTDWVMHEYRL DDKVLTQMNVPQDTYVVCVLFKKDGPGPRNGAQYGAPFKEED WSDEEVRTDVPSTSNPTNLLEPSKETTLALTAPDDSNKDCFGGMI SESCVSDFLPATTNTTSELPHPSDAATTPMSTAPLAETVQTPNND DLYSMLDLFDDDEEFLGFNNNEVRYDPGVSAPVCLEEEGIFNGLP ELSSMPRTASYDLVENSELYLELQDLTAPLNPQTGLQDLTAPFNP QTGLQDLTAPFNHQTGLQDHTAPFNPQTGLQDHTAPFNHQTGL QDLTAPFNPQTGLQDLTAPFNPQTGLHDLTSPFNPQTGLQDLTA PLNPQTGNRNDPRSSSFLYNQGHFDFSGGNDDDPYGFSASMRHR PKM

AT5G09330.5	MGKTQLAPGFRFHPTDVELVRYYLKRKILGKKLLVDAIAEVDIYKFEPPDLPDMSFIRSGDLKWHFFCPREKKYASGVRANRATECGYWKTTGKERPVLCNSEVVGKIKTLVYHFGKSPRGERTDWVMHEYRLDDKVLTQMNVPQDTYVVCVLFKKDGPGPRNGAQYGAPFKEEDWSDEEVRTDVPSTSNPTNLLEPSKETTLALTAPDDSNKDCFGGMISESCVSDFLPATTNTTSELPHPSDAATTPMSTAPLAETVQTPNNDDLYSMLDLFDDDEEFLGFNNNEVRYDPGVSAPVCLEEEGIFNGLPELSSMPRTASYDLVENSELYLELQDLTAPLNPQTGLQDLTAPFNPQTGLQDLTAPFNHQTGLQDHTAPFNPQTGLQDHTAPFNHQTGLQDLTAPFNPQTGLQDLTAPFNPQTGLHDLTSPFNPQTGLQDLTAPLNPQTGNRNDPRSSSFLYNQGHFDFSGGNDDDPYGFSASMRHRPKM
AT1G32510.1	MVGSFLPPGFRFYPTDEELVGYYLHRRNEGLEIELEIIPLMDLYKFDPWELPEKSFLPNRDMEWFFFCHRDRKYQNGSRINRATKSGYWKATGKDRKIVCHSSSSSSSSSITGCRKTLVFYMGRAPFGGRTEWVMHEYRLFDNDTSQGSLNFKGDFALCRVIKRNEHTLKKCEIISPEVSDESLSNNVNNFCQASDLEKGSCDASNTRLSSPDFILESSFQGNSHSKTEEDSGFQVFTLPEFEYPLEVFADLNFDLEMEDPFMFDYHPEPHMNNEVMSHHIRG
AT2G18060.1	MEPMESCSVPPGFRFHPTDEELVGYYLRKKIASQKIDLDVIRDIDLYRIEPWDLQEQCRIGYEEQNEWYFFSHKDKKYPTGTRTNRATMAGFWKATGRDKAVYDKTKLIGMRKTLVFYKGRAPNGKKSDWIMHEYRLESDENAPPQEEGWVVCRAFKKRATGQAKNTETWSSSYFYDEVAPNGVNSVMDPIDYISKQQHNIFGKGLMCKQELEGMVDGINYIQSNQFIQLPQLQSPSLPLMKRPSSSMSITSMDNNYNYKLPLADEESFESFIRGEDRRKKKKQVMMTGNWRELDKFVASQLMSQEDNGTSSFAGHHIVNEDKNNNDVEMDSSMFLSEREEENRFVSEFLSTNSDYDIGICVFDN
AT2G18060.3	MEPMESCSVPPGFRFHPTDEELVGYYLRKKIASQKIDLDVIRDIDLYRIEPWDLQEQCRIGYEEQNEWYFFSHKDKKYPTGTRTNRATMAGFWKATGRDKAVYDKTKLIGMRKTLVFYKGRAPNGKKSDWIMHEYRLESDENAPPQEEGWVVCRAFKKRATGQAKNTETWSSSYFYDEVAPNGVNSVMDPIDYISKQQHNIFGKGLMCKQELEGMVDGINYIQSNQFIQLPQLQSPSLPLMKRPSSSMSITSMDNNYNYKLPLADEESFESFIRGEDRRKKKKQVMMTGNWRELDKFVASQLMSQEDNGTSSFAGHHIVNEDKNNNDVEMDSSMFLSEREEENRFVSEFLSTNSDYDIGICVFDN
AT1G69490.1	MEVTSQSTLPPGFRFHPTDEELIVYYLRNQTMSKPCPVSIIPEVDIYKFDPWQLPEKTEFGENEWYFFSPRERKYPNGVRPNRAAVSGYWKATGTDKAIHSGSSNVGVKKALVFYKGRPPKGIKTDWIMHEYRLHDSRKASTKRNGSMRLDEWVLCRIYKKRGASKLLNEQEGFMDEVLMEDETKVVVNEAERRTEEEIMMMTSMKLPRTCSLAHLLEMDYMGPVSHIDNFSQFDHLHQPDSESSWFGDLQFNQDEILNHHRQAMFKF

AT1G19040.1	MSEVLTRGFRFRPADEEVTDYLMRKTQSPEFSCFIKTIDLYDKDPW<br>VLGHVRDPCYNHYEWYYFVKKNPLGNEYLDKPRLNGFEHPRLK<br>WDVLKQIHDALSSGQNVPPSVFTYGFRFHPTDQELLSLYLTPKAH<br>SPDFSCFIRERESYMPWSHGFLNMLDLLFTRTVNGTTL
AT1G03490.1	MKILPVGSRFCPTDLGLVRLYLRNKVERNQSSFITTMDIHQDYPW<br>LLPHVNNPLFNNNEWYYFVPLTERGGKILSVHRKVAARGGSEGG<br>TWRSNDGKKEIKDGHMQKGDGLRASDDLQKVVLCRIRYKKEAN<br>VNEFGLVNHQAHQTQDALTGFADQLEMMLEGQEDREQKEEAD<br>LTGFADSLETMLEGQEDHEQPEDADLTGFADSLETMLEGHEDRE<br>QPEEAELTVTQQQQQQQQQQRQEDCDVTQEQEKDDVMVLIN<br>NPNDALALGNYEIIDLTRVDK
AT3G15500.1	MGLQELDPLAQLSLPPGFRFYPTDEELMVEYLCRKAAGHDFSLQL<br>IAEIDLYKFDPWVLPSKALFGEKEWYFFSPRDRKYPNGSRPNRVA<br>GSGYWKATGTDKVISTEGRRVGIKKALVFYIGKAPKGTKTNWIM<br>HEYRLIEPSRRNGSTKLDDWVLCRIYKKQTSAQKQAYNNLMTSG<br>REYSNNGSSTSSSSHQYDDVLESLHEIDNRSLGFAAGSSNALPHSH<br>RPVLTNHKTGFQGLAREPSFDWANLIGQNSVPELGLSHNVPSIRY<br>GDGGTQQQTEGIPRFNNNSDVSANQGFSVDPVNGFGYSGQQSSG<br>FGFI
AT5G61430.2	MAKMGEKEWYFFCVRDRKYPTGLRTNRATEAGYWKATGKDKEI<br>YRGKSLVGMKKTLVFYRGRAPKGQKTNWVMHEYRLEGKFSAH<br>NLPKTAKNEWVICRVFQKSAGGKKIPISSLIRIGSLGTDFNPSLLPS<br>LTDSSPYNDKTKTEPVYVPCFSNQTDQNQGTTLNCFSSPVLNSIQ<br>ADIFHRIPLYQTQSLQVSMNLQSPVLTQEHSVLHAMIENNRRQSL<br>KTMSVSQETGVSTDMNTDISSDFEFGKRRFDSQEDPSSSTGPVDLE<br>PFWNY
AT5G61430.1	METFCGFQKEEEQMDLPPGFRFHPTDEELITHYLHKKVLDTSFSA<br>KAIGEVDLNKSEPWELPWMAKMGEKEWYFFCVRDRKYPTGLRT<br>NRATEAGYWKATGKDKEIYRGKSLVGMKKTLVFYRGRAPKGQK<br>TNWVMHEYRLEGKFSAHNLPKTAKNEWVICRVFQKSAGGKKIPI<br>SSLIRIGSLGTDFNPSLLPSLTDSSPYNDKTKTEPVYVPCFSNQTDQ<br>NQGTTLNCFSSPVLNSIQADIFHRIPLYQTQSLQVSMNLQSPVLTQ<br>EHSVLHAMIENNRRQSLKTMSVSQETGVSTDMNTDISSDFEFGKR<br>RFDSQEDPSSSTGPVDLEPFWNY
AT5G08790.1	MKSELNLPAGFRFHPTDEELVKFYLCRKCASEQISAPVIAEIDLYK<br>FNPWELPEMSLYGEKEWYFFSPRDRKYPNGSRPNRAAGTGYWK<br>ATGADKPIGKPKTLGIKKALVFYAGKAPKGIKTNWIMHEYRLAN<br>VDRSASVNKKNNLRLDDWVLCRIYNKKGTMEKYFPADEKPRTTT<br>MAEQSSSPFDTSDSTYPTLQEDDSSSSGGHGHVVSPDVLEVQSEPK<br>WGELEDALEAFDTSMFGSSMELLQPDAFVPQFLYQSDYFTSFQDP<br>PEQKPFLNWSFAPQG

AT1G52880.1	MESTDSSGGPPPPQPNLPPGFRFHPTDEELVIHYLKRKADSVPLPVAIIADVDLYKFDPWELPAKASFGEQEWYFFSPRDRKYPNGARPNRAATSGYWKATGTDKPVISTGGGGSKKVGVKKALVFYSGKPPKGVKSDWIMHEYRLTDNKPTHICDFGNKKNSLRLDDWVLCRIYKKNNSTASRHHHHLHHIHLDNDHHRHDMMIDDDRFRHVPPGLHFPAIFSDNNDPTAIYDGGGGGYGGGSYSMNHCFASGSKQEQLFPPVMMMTSLNQDSGIGSSSSPSKRFNGGGVGDCSTSMAATPLMQNQGGIYQLPGLNWYS
AT5G14000.1	MEVEKRIVVNGGMKLPIGYRFHPTEQELILHYLLPKAFASPLPSSIIPVFDLFFSHPLSFPGDQKEKQRYFFCKKREVSSNEHRIKISSGDGYWKPIGKERPIIACGKTFGIRRTLAFYETNKSSSYCNKTRWSMTEYCLAGFASAKVSGEWAVYNVYERKGSKGRKQRKSREGDDEELRCIDHFTVGSNHETGPPPPSPPTSADE
AT5G14000.2	MEVEKRIVVNGGMKLPIGYRFHPTEQELILHYLLPKAFASPLPSSIIPVFDLFFSHPLSFPGDQKEKQRYFFCKKREVSSNEHRIKISSGDGYWKPIGKERPIIACGKTFGIRRTLAFYETNKSSSYCNKTRWSMTEYCLAGFASAKVHNNYSRLL
AT5G14000.3	MEVEKRIVVNGGMKLPIGYRFHPTEQELILHYLLPKAFASPLPSSIIPVFDLFFSHPLSFPGLFLSLSLSLCSFCQSLYVCICLLVFLNCFLFKMVYLMFGSRSGVSDAKTEEVLLSGDQKEKQRYFFCKKREVSSNEHRIKISSGDGYWKPIGKERPIIACGKTFGIRRTLAFYETNKSSSYCNKTRWSMTEYCLAGFASAKVSGEWAVYNVYERKGSKGRKQRKSREGDDEELRCIDHFTVGSNHETGPPPPSPPTSADE
AT5G50820.2	MWFSECCTYTFVVGEKNKEVYYFVKKEERERKGRETLSGYWEECEEEEVMEAGGRDCIHLEGRRKTFAFFIGKKPRGTITPWIMYEFRLLSSRATRWSSSPLPRGEVGKWRAVKVVVKEENDEEMVEDEHESDESDGEEVIQSR
AT3G18400.1	MEENLPPGFRFHPTDEELITHYLCRKVSDIGFTGKAVVDVDLNKCEPWDLPAKASMGEKEWYFFSQRDRKYPTGLRTNRATEAGYWKTTGKDKEIYRSGVLVGMKKTLVFYKGRAPKGEKSNWVMHEYRLESKQPFNPTNKEEWVVCRVFEKSTAAKKAQEQQPQSSQPSFGSPCDANSSMANEFEDIDELPNLNSNSSTIDYNNHIHQYSQRNVYSEDNTTSTAGLNMNMNMASTNLQSWTTSLLGPPLSPINSLLLKAFQIRNSYSFPKEMIPSFNHSSLQQGVSNMIQNASSSSQVQPQPQEEAFNMDSIW
AT1G01720.1	MSELLQLPPGFRFHPTDEELVMHYLCRKCASQSIAVPIIAEIDLYKYDPWELPGLALYGEKEWYFFSPRDRKYPNGSRPNRSAGSGYWKATGADKPIGLPKPVGIKKALVFYAGKAPKGEKTNWIMHEYRLADVDRSVRKKKNSLRLDDWVLCRIYNKKGATERRGPPPPVVYGDEIMEEKPKVTEMVMPPPPQQTSEFAYFDTSDSVPKLHTTDSSCSEQVVSPEFTSEVQSEPKWKDWSAVSNDNNNTLDFGFNYIDATVDNAFGGGGSSNQMFPLQDMFMYMQKPY

AT5G14490.2	MERTWIVDGPWISRNVKNASLSSALQIKDCGAYINCFNCSYRIDNSNVLTPWPGLPKGVKFEPTDEEVIEHLEAKCGIDGLKPHLLIQDFICSVTQDVGINYTHPQNLPGVSKDGTSVFFFNKTAHAYQNGQRKRRRITPTSLKDDTVRWHKTGQTKPVMLNGIQKGCKKIMVLYKSARKGFKPEKSNWVLHQYHLGTEEGEIGEYVVSKITYQQPKQQEKTIDESESSGVRGGPSTPKTSTITQVRPVISVDEDEIAFDDDSKMVLDSYAEVSSFLCKYQ
AT5G14490.1	MKTLHRTWIVDGPWISRNVKNASLSSALQIKDCGAYINCFNCSYRIDNSNVLTPWPGLPKGVKFEPTDEEVIEHLEAKCGIDGLKPHLLIQDFICSVTQDVGINYTHPQNLPGVSKDGTSVFFFNKTAHAYQNGQRKRRRITPTSLKDDTVRWHKTGQTKPVMLNGIQKGCKKIMVLYKSARKGFKPEKSNWVLHQYHLGTEEGEIGEYVVSKITYQQPKQQEKTIDESESSGVRGGPSTPKTSTITQVRPVISVDEDEIAFDDDSKMVLDSYAEGLENIQEASSGSTSDKIAKVGGNVSVIEDNLMSKKIEASSIPNHGNVDYGSGNFSVSDLENAELGTLPDLLSVRT
AT5G14490.4	MERTWIVDGPWISRNVKNASLSSALQIKDCGAYINCFNCSYRIDNSNVLTPWPGLPKGVKFEPTDEEVIEHLEAKCGIDGLKPHLLIQDFICSVTQDVGINYTHPQNLPGVSKDGTSVFFFNKTAHAYQNGQRKRRRITPTSLKDDTVRWHKTGQTKPVMLNGIQKGCKKIMVLYKSARKGFKPEKSNWVLHQYHLGTEEGEIGEYVVSKITYQQPKQQEKTIDESESSGVRGGPSTPKTSTITQVRPVISVDEDEIAFDDDSKMVLDSYAEGLENIQEASSGSTSDKIAKVGGNVSVIEDNLMSKKIEASSIPNHGNVDYGSGNFSVSDLENAELGTLPDLLSVRT
AT5G14490.3	MKTLHRTWIVDGPWISRNVKNASLSSALQIKDCGAYINCFNCSYRIDNSNVLTPWPGLPKGVKFEPTDEEVIEHLEAKCGIDGLKPHLLIQDFICSVTQDVGINYTHPQNLPGVSKDGTSVFFFNKTAHAYQNGQRKRRRITPTSLKDDTVRWHKTGQTKPVMLNGIQKGCKKIMVLYKSARKGFKPEKSNWVLHQYHLGTEEGEIGEYVVSKITYQQPKQQEKTIDESESSGVRGGPSTPKTSTITQVRPVISVDEDEIAFDDDSKMVLDSYAEGLENIQEASSGSTSDKIAKVGGNVSVIEDNLMSKKIEASSIPNHGNVDYGSGNFSVSDLENAELGTLPDLLSFASEDSLMNWLGWF
AT1G12260.1	MNSFSHVPPGFRFHPTDEELVDYYLRKKVASKRIEIDFIKDIDLYKIEPWDLQELCKIGHEEQSDWYFFSHKDKKYPTGTRTNRATKAGFWKATGRDKAIYLRHSLIGMRKTLVFYKGRAPNGQKSDWIMHEYRLETDENGTPQEEGWVVCRVFKKRLAAVRRMGDYDSSPSHWYDDQLSFMASELETNGQRRILPNHHQQQHEHQQHMPYGLNASAYALNNPNLQCKQELELHYNHLVQRNHLLDESHLSFLQLPQLESPKIQQDNSNCNSLPYGTSNIDNNSSHNANLQQSNIAHEEQLNQGNQNFSSLYMNSGNEQVMDQVTDWRVLDKFVASQLSNEEAATASASIQNNAKDTSNAEYQVDEEKDPKRASDMGEEYTASTSSSCQIDLWK

AT1G12260.2	MNSFSHVPPGFRFHPTDEELVDYYLRKKVASKRIEIDFIKDIDLYKIEPWDLQELCKIGHEEQSDWYFFSHKDKKYPTGTRTNRATKAGFWKATGRDKAIYLRHSLIGMRKTLVFYKGRAPNGQKSDWIMHEYRLETDENGTPQEEGWVVCRVFKKRLAAVRRMGDYDSSPSHWYDDQLSFMASELETNGQRRILPNHHQQQHEHQQHMPYGLNASAYALNNPNLQCKQELELHYNHLQSNIAHEEQLNQGNQNFSSLYMNSGNEQVMDQVTDWRVLDKFVASQLSNEEAATASASIQNNAKDTSNAEYQVDEEKDPKRASDMGEEYTASTSSSCQIDLWK
AT1G34180.2	MVDSSRDSCFKAGKFSAPGFRFHPTDEELVVYYLKRKICCKKLRVNAIGVVDVYKVDPSELPGNFQHLLIDFDSCLSMLKTGDRQWFFFTPRNRKYPNAARSSRGTATGYWKATGKDRVIEYNSRSVGLKKTLVFYRGRAPNGERTDWVMHEYTMDEEELGRCKNAKEYYALYKLYKKSGAGPKNGEQYGAPFQEEEWVDSDSEDADSVAVPDYPVVRYENGPCVDDTKFCNPVKLQLEDIEKLLNEIPDAPGVNQRQFDEFVGVPQGNSAEVIQSTLLNNSSGEYIDPRTNGMFLPNGQLYNRDSSFQSHLNSFEATSGMAPLLDNEKEEYIEMNDLLIPELGASSTEKSTEFLNHGEFGDVNEYDQLFNDISVFQGTSTDLSCLSNFTNNTSGQRQQLLYEQFQYQTPENQLNNYMHPSTTLNQFTDNMWFKDDQAALYVQPPQSSSGAFTSQSTGVMPESMNPTMSVNPQYKEGQNGGGTRSQFSSALWELLESIPSTPASACEGPLNQTFVRMSSFSRIRFNGTSVTSRKVTVAKKRISNRGFLLLSIMGALCAIFWVFKATVGVMGRPLLS
AT1G34180.1	MVDSSRDSCFKAGKFSAPGFRFHPTDEELVVYYLKRKICCKKLRVNAIGVVDVYKVDPSELPGLSMLKTGDRQWFFFTPRNRKYPNAARSSRGTATGYWKATGKDRVIEYNSRSVGLKKTLVFYRGRAPNGERTDWVMHEYTMDEEELGRCKNAKEYYALYKLYKKSGAGPKNGEQYGAPFQEEEWVDSDSEDADSVAVPDYPVVRYENGPCVDDTKFCNPVKLQLEDIEKLLNEIPDAPGVNQRQFDEFVGVPQGNSAEVIQSTLLNNSSGEYIDPRTNGMFLPNGQLYNRDSSFQSHLNSFEATSGMAPLLDNEKEEYIEMNDLLIPELGASSTEKSTEFLNHGEFGDVNEYDQLFNDISVFQGTSTDLSCLSNFTNNTSGQRQQLLYEQFQYQTPENQLNNYMHPSTTLNQFTDNMWFKDDQAALYVQPPQSSSGAFTSQSTGVMPESMNPTMSVNPQYKEGQNGGGTRSQFSSALWELLESIPSTPASACEGPLNQTFVRMSSFSRIRFNGTSVTSRKVTVAKKRISNRGFLLLSIMGALCAIFWVFKATVGVMGRPLLS

AT1G34180.3	MVDSSRDSCFKAGKFSAPGFRFHPTDEELVVYYLKRKICCKKLRVNAIGVVDVYKVDPSELPGLSMLKTGDRQWFFFTPRNRKYPNAARSSRGTATGYWKATGKDRVIEYNSRSVGLKKTLVFYRGRAPNGERTDWVMHEYTMDEEELGRCKNAKEYYALYKLYKKSGAGPKNGEQYGAPFQEEEWVDSDSEDADSVAVPDYPVVRYENGPCVDDTKFCNPVKLQLEDIEKLLNEIPDAPGVNQRQFDEFVGVPQGNSAEVIQSTLLNNSSGEYIDPRTNGMFLPNGQLYNRDSSFQSHLNSFEATSGMAPLLDNEKEEYIEMNDLLIPELGASSTEKSTEFLNHGEFGDVNEYDQLFNDISVFQGTSTDLSCLSNFTNNTSGQRQQLLYEQFQYQTPENQLNNYMHPSTTLNQFTDNMWFKDDQAALYVQPPQSSSGAFTSQSTGT
AT1G34180.4	MVDSSRDSCFKAGKFSAPGFRFHPTDEELVVYYLKRKICCKKLRVNAIGVVDVYKVDPSELPGNFQHLLIDFDSCLSMLKTGDRQWFFFTPRNRKYPNAARSSRGTATGYWKATGKDRVIEYNSRSVGLKKTLVFYRGRAPNGERTDWVMHEYTMDEEELGRCKNAKEYYALYKLYKKSGAGPKNGEQYGAPFQEEEWVDSDSEDADSVAVPDYPVVRYENGPCVDDTKFCNPVKLQLEDIEKLLNEIPDAPGVNQRQFDEFVGVPQGNSAEVIQSTLLNNSSGEYIDPRTNGMFLPNGQLYNRDSSFQSHLNSFEATSGMAPLLDNEKEEYIEMNDLLIPELGASSTEKSTEFLNHGEFGDVNEYDQLFNDISVFQGTSTDLSCLSNFTNNTSGQRQQLLYEQFQYQTPENQLNNYMHPSTTLNQFTDNMWFKDDQAALYVQPPQSSSGAFTSQSTGT
AT3G44290.2	MAAAPPIEPAVTTTFPGFKFSPTDIELISYYLKRKMDGLERSVEIIPEVEIYNFEPWDLPDKSIVKSDSEWFFFCARGKKYPHGSQNRRATKIGYWKATGKERNVKSGSEVIGTKRTLVFHIGRAPKGGRTEWLMHEYCMIGVSLDALVICRLRRNTEFQGSTIQKPPQPSLPLDKHVNLRNEAISESIYGWETMVDFYLSSESGQELLSEIAESSQSSQNPQEFFHV
AT3G44290.1	MAAAPPIEPAVTTTFPGFKFSPTDIELISYYLKRKMDGLERSVEIIPEVEIYNFEPWDLPDKSIVKSDSEWFFFCARGKKYPHGSQNRRATKIGYWKATGKERNVKSGSEVIGTKRTLVFHIGRAPKGGRTEWLMHEYCMIGVSLDALVICRLRRNTEFQGSTIQKPPQPSLPLDKHVNLRNEAISESIYGWETMVDFYLSSESGQELLSEIAESSQSSQNPQVPSEEDFYADILRDEIVKLDDPAVSGNTLINVPRLQTESNTTRVLPLPDMVDKQMQSLLQKLPLQNDTGEENNISMSNCFIGIYSIKSINRARWDVVVWLLVMIAVLVFYLV
AT3G15170.1	MDVDVFNGWGRPRFEDESLMPPGFRFHPTDEELITYYLLKKVLDSNFSCAAISQVDLNKSEPWELPEKAKMGEKEWYFFTLRDRKYPTGLRTNRATEAGYWKATGKDREIKSSKTKSLLGMKKTLVFYKGRAPKGEKSCWVMHEYRLDGKFSYHYISSSAKDEWVLCKVCLKSGVVSRETNLISSSSSSAVTGEFSSAGSAIAPIINTFATEHVSCFSNNSAAHTDASFHTFLPAPPPSLPPRQPRHVGDGVAFGQFLDLGSSGQIDFDAAAAAFFPNLPSLPPTVLPPPPSFAMYGGGSPAVSVWPFTL

AT3G04060.1	MVEEGGVVVNQGGDQEVVDLPPGFRFHPTDEEIITHYLKEKVFNI<br>RFTAAAIGQADLNKNEPWDLPKIAKMGEKEFYFFCQRDRKYPTG<br>MRTNRATVSGYWKATGKDKEIFRGKGCLVGMKKTLVFYTGRAP<br>KGEKTNWVMHEYRLDGKYSYHNLPKTARDEWVVCRVFHKNAP<br>STTITTTKQLSRIDSLDNIDHLLDFSSLPPLIDPGFLGQPGPSFSGAR<br>QQHDLKPVLHHPTTAPVDNTYLPTQALNFPYHSVHNSGSDFGY<br>GAGSGNNNKGMIKLEHSLVSVSQETGLSSDVNTTATPEISSYPMM<br>MNPAMMDGSKSACDGLDDLIFWEDLYTS
AT3G49530.2	MNQNLHVLSMDSLPVGLRFRPTDEELIRYYLRRKINGHDDDVKA<br>IREIDICKWEPWDLPDFSVIKTKDSEWLYFCPLDRKYPSGSRQNRA<br>TVAGYWKATGKDRKIKSGKTNIIGVKRTLVFHAGRAPRGTRTNW<br>IIHEYRATEDDLSGTNPGQSPFVICKLFKKEELVLGEEDSKSDEVEE<br>PAVSSPTVEVTKSEVSEVIKTEDVKRHDIAESSLVISGDSHSDACDE<br>ATTAELVDFKWYPELESLDFTLFSPLHSQVQSELGSSYNTFQPGSS<br>NFSGNNNNSFQIQTQYGTNEVDTYISDFLDSILKSPDEDPEKHKY<br>VLQSGFDVVAPDQIAQVCQQGSAVDMSNDVSVTGIQIKSRQAQP<br>SGYTNDYIAQGNGPRRLRLQSNFNGINTKNPELQAIKREVRITPTF
AT3G49530.1	MNQNLHVLSMDSLPVGLRFRPTDEELIRYYLRRKINGHDDDVKA<br>IREIDICKWEPWDLPDFSVIKTKDSEWLYFCPLDRKYPSGSRQNRA<br>TVAGYWKATGKDRKIKSGKTNIIGVKRTLVFHAGRAPRGTRTNW<br>IIHEYRATEDDLSGTNPGQSPFVICKLFKKEELVLGEEDSKSDEVEE<br>PAVSSPTVEVTKSEVSEVIKTEDVKRHDIAESSLVISGDSHSDACDE<br>ATTAELVDFKWYPELESLDFTLFSPLHSQVQSELGSSYNTFQPGSS<br>NFSGNNNNSFQIQTQYGTNEVDTYISDFLDSILKSPDEDPEKHKY<br>VLQSGFDVVAPDQIAQVCQQGSAVDMSNDVSVTGIQIKSRQAQP<br>SGYTNDYIAQGNGPRRLRLQSNFNGINTKNPELQAIKREAEDTVG<br>ESIKKRCGKLMRSKNVTGFVFKKITSVKCSYGGLFRAAVVAVVFL<br>MSVCSLTVDFRASAVS
AT4G17980.2	MGSSCLPPGFRFHPTDEELIGYYLSRKIEGLEIELEVIPVIDLYKFDP<br>WELPGKSFLPNRDLEWFFFCPRDKKYANGSRTNRATKAGYWKA<br>TGKDRKITCKSSHVIAGYRKTLVFYEGRAPLGDRTNWFMHEYRL<br>CDIDDHSQKSPNFKGAFALCRVVKKNELKKNSKSLKNKNEQDIG<br>SCYSSLATSPCRDEASQIQSFKPSSTTNDSSSIWISPDFILDSSKDYPQ<br>IKEVASECFPNYHFPVTTANHHVEFPVSSCYFNVDQDIDQSMQTG<br>YWTNYENDQTGSFDYSNLF
AT4G17980.1	MGSSCLPPGFRFHPTDEELIGYYLSRKIEGLEIELEVIPVIDLYKFDP<br>WELPGKSFLPNRDLEWFFFCPRDKKYANGSRTNRATKAGYWKA<br>TGKDRKITCKSSHVIAGYRKTLVFYEGRAPLGDRTNWFMHEYRL<br>CDIDDHSQKSPNFKGAFALCRVVKKNELKKNSKSLKNKNEQDIG<br>SCYSSLATSPCRDEASQIQSFKPSSTTNDSSSIWISPDFILDSSKDYPQ<br>IKEVASECFPNYHFPVTTANHHVEFPLQEMLVRS

AT5G39610.1	MDYEASRIVEMVEDEEHIDLPPGFRFHPTDEELITHYLKPKVFNTFFSATAIGEVDLNKIEPWDLPWKAKMGEKEWYFFCVRDRKYPTGLRTNRATEAGYWKATGKDKEIFKGKSLVGMKKTLVFYKGRAPKGVKTNWVMHEYRLEGKYCIENLPQTAKNEWVICRVFQKRADGTKVPMSMLDPHINRMEPAGLPSLMDCSQRDSFTGSSSHVTCFSDQETEDKRLVHESKDGFGSLFYSDPLFLQDNYSLMKLLLDGQETQFSGKPFDGRDSSGTEELDCVWNF
AT1G60280.1	MKVEDEATYELIKDELMKAEDEATYWLIKEELIKAEDDVIISRYLKRMIVNGDSWPDHFIEDVDVFNKNPNEEFHSQSPRFVIVKPRTENCGRTDGCQSGCWRIIGRDKLIKSKETGKILGFKKILKFCLKKKPREYKRSWVMEEYRLNNNLNCKQDHVICKIRFMFDAEISFLLAKHFSCLSTRSPLPANQLLPAYGVCFFDSEAEGAFYLETIIGYDGNTWPSYVTNDVYRLHPLTLVDPQDDKFKEFGTCIFANRTKTCGKTDECDGGGCWRIVEGHRVIKSKGKVLGYRRIFQFSENEEPRNVCEGEDPKKTAWFIEEYRPDENNKKDKVLCVIKFLIPLNQR
AT1G60350.1	MEEDAAFDLLKAELLNAEDDAIISRYLKRMVVNGDSWPDHFIEDADVFNKNPNVEFDAESPSFVIVKPRTEACGKTDGCETGCWRIMGRDKPIKSTETVKIQGFKKILKFCLKRKPRGYKRSWVMEEYRLTNNLNWKQDHVICKIRFMFEAEISFLLAKHFYTTSESLPRNELLPAYGFLSSDKQLEDVSYPVTIMTSEGNDWPSYVTNNVYCLHPLELVDLQDRMFNDYGTCIFANKTCGKTDRCINGGYWKILHRDRLIKSKSGIVIGFKKVFKFHETEKERYFCGGEDVKVTWTLEEYRLSVKQNKFLCVIKFTYDN
AT3G17730.1	MAPVGLPPGFRFHPTDEELVNYYLKRKINGQEIELDIIPEVDLYKCEPWDLAEKSFLPSRDPEWYFFGPRDRKYPNGFRTNRATRGGYWKSTGKDRRVTSQSRAIGMKKTLVYYKGRAPQGIRTDWVMHEYRLDDKDCDDPSSLQDSYALCRVFKKNGICSELESERQLQTGQCSFTTASMEEINSNNNNNYNNDYETMSPEVGVSSACVEEVVDDKDDSWMQFITDDAWDTSSNGAAMGHGQGVY
AT4G01550.1	MVKDLVGYRFYPTGEELINHYLKNKILGKTWLVDEAISEINICSYDPIYLPSLSKIKSDDPVWYFFCPKEYTSAKKKVTKRTTSSGYWKATGVDRKIKDKRGNRGEIGIKKTLVYYEGRVPKGVWTPWVMHEYHITCLPQDQRNYVICQVMYKGEDGDVPSGGNNSSEPSQSLVSDSNTVRATSPTALEFEKPGQENFFGMSVDDLGTPKNEQEDFSLWDVLDPDMLFSDNNNPTVHPQAPHLTPNDDEFLGGLRHVNREQVEYLFANEDFISRPTLSMTENRNDHRPKKALSGIIVDYSSDSNSDAESISATSYQGTSSPGDDSVGSSNRQFLQTGGDEILSSCNDLQTYGEPSISSSTRQSQLTRSIIRPKQEVKQDTSRAVDSDTSIDKESSMVKTEKKSWFITEEAMERNRNNPRYIYLMRMIIGFILLLALISNIISVLQNLNPAMKFDRER

AT3G04430.1	MYRCTFNPPEEELINYYLNNKITENDDLVGKQIAEVNILHHEPAD<br>LPGLAKIESSHTWYFISPVEKFGKLNRTKRVSRSGHWKITGNSRTI<br>KDVDGNPIGLKKFLVFQENKRSSSSSLSTTTTQQQKTNWIIHEFHS<br>FLPHPNKMLTENKNEDYGGDYCNGLDELLQSLEQGESSGLLFGE<br>NGHACEQDTAENSNVTM
AT1G02250.1	MANPVGFRFRPTDGEIVDIYLRPKNLESNTSHVDEVISTVDICSFDP<br>WDLPSHSRMKTRDQVWYFFGRKENKYGKGDRQIRKTKSGFWKK<br>TGVTMDIMRKTGDREKIGEKRVLVFKNHGGSKSDWAMHEYHAT<br>FSSPNQIMTYTLCKVKFKGERREFSVATGSGIKHTHSLIPPTNNSG<br>VLSVETEGSLFHSQESQNPSQFSGFLDVDALDRDFCNILSDDFKGF<br>FNDDDEQSKIVSMQDDRNNHTPQKPLTGVFSDHSTDGSDSDPIS<br>ATTISIQTLSTCPSFGSSNPLYQITDLQESPNSIKLVSLAQEVSKTPGT<br>GIDNDAQGTEIGEHKLGQETIKNKRAGFFHRMIQKFVKKIHLRT
AT5G62380.1	MESLAHIPPGYRFHPTDEELVDYYLKNKVAFPGMQVDVIKDVDL<br>YKIEPWDIQELCGRGTGEEREWYFFSHKDKKYPTGTRTNRATGSG<br>FWKATGRDKAIYSKQELVGMRKTLVFYKGRAPNGQKSDWIMHE<br>YRLETDENGPPHEEGWVVCRAFKKKLTTMNYNNPRTMMGSSSG<br>QESNWFTQQMDVGNGNYYHLPDLESPRMFQGSSSSSLSSLHQND<br>QDPYGVVLSTINATPTTIMQRDDGHVITNDDDHMIMMNTSTGD<br>HHQSGLLVNDDHNDQVMDWQTLDKFVASQLIMSQEEEEVNKD<br>PSDNSSNETFHHLSEEQAATMVSMNASSSSSPCSFYSWAQNTHT
AT5G62380.2	MESLAHIPPGYRFHPTDEELVDYYLKNKVAFPGMQVDVIKDVDL<br>YKIEPWDIQELCGRGTGEEREWYFFSHKDKKYPTGTRTNRATGSG<br>FWKATGRDKAIYSKQELVGMRKTLVFYKGRAPNGQKSDWIMHE<br>YRLETDENGPPHEEGWVVCRAFKKKLTTMNYNNPRTMMGSSSG<br>QESNWFTQQMDVGNGNYYHLPDLESPRMFQGSSSSSLSSLHQND<br>QDPYGVVLSTINATPTTIMQRDDGHVITNDDDHMIMMNTSTGD<br>HHQSGLLVNDDHNDQVMDWQTLDKFVASQLIMSQEEEEVNKD<br>PSDNSSNETFHHLSEEQAATMVSMNASSSSSPCSFYSWAQNTHT
AT3G01600.1	MARAWIVDGRGIAAKVKNASLSSALQIQDCGAHIKCPNCTYRID<br>NSNVLIPWPGLPKGVKFEPTDEDIIEFLEAKCGIGGSEPHVLIEEFIR<br>PVTEDVGINYTHPQNLPGANKDGVSVFFFHKTVQAYGTGQRKRR<br>KITPTLVNDEPVRWHKTGRTKPVMLSGVQRGCKKIMVLYKSARK<br>GTKPEKSNWVLHQYHLGTEGKEIGDYVVSKITYQQQKLGENPDE<br>GESSSGVRGGPTTPKTNTPTPPSLVDGVAGDEEAFDDLKMFDPFF<br>EELDSIPEAALGKMWSKKARMDEEFVVNLSEDNLICDESMEASSL<br>WENQVLPNPSLGTVGDFDGFSISDLENADLGTPPDFLTLASQESL<br>LNWIGWL

AT5G17260.1	MAPVSLPPGFRFHPTDEELITYYLKRKINGQEIELEIIPEVDLYKCEPWDLPGKSLIPSKDQEWFFFSPRDRKYPNGSRTNRATKGGYWKATGKDRRVSWRDRAIGTKKTLVYYRGRAPHGIRTGWVMHEYRLDESECEPSAFGMQDAYALCRVFKKIVIEAKPRDQHQQQHQPYVHTSSNISGSSSFDVCSDLEISSNTPYNTAAHIQPRFGNANAISDHDDWSQYLSQNMPTSFSDYGSPYGPYLTQSKVNTEVQCEMFQHQMSLPPLRVENSQAQTSDFSKRLHQNSGQSGFDDFTFAASNSNQFYNSNVDDHLIHIGNLDEQSYIEEQELILPSFQSNDQDLELYGGSRTNTIDNIEIDDFFSFENQAQDNDNSNVTPNSAGFEMIGEEIIVNHKMLISTRQTTEILYYQVVPSQILKIHINPVHGNEERTMLMEEDSDDSWFQKAENVAKMKLKQISLVAKRYYKCLTIIF
AT4G27410.3	MGVREKDPLAQLSLPPGFRFYPTDEELLVQYLCRKVAGYHFSLQVIGDIDLYKFDPWDLPSKQTCFTFVGEYNCNYLGKALFGEKEWYFFSPRDRKYPNGSRPNRVAGSGYWKATGTDKIITADGRRVGIKKALVFYAGKAPKGTKTNWIMHEYRLIEHSRSHGSSKLDDWVLCRIYKKTSGSQRQAVTPVQACREEHSTNGSSSSSSSQLDDVLDSFPEIKDQSFNLPRMNSLRTILNGNFDWASLAGLNPIPELAPTNGLPSYGGYDAFRAAEGEAESGHVNRQQNSSGLTQSFGYSSSGFGVSGQTFEFRQ
AT4G27410.2	MGVREKDPLAQLSLPPGFRFYPTDEELLVQYLCRKVAGYHFSLQVIGDIDLYKFDPWDLPSKALFGEKEWYFFSPRDRKYPNGSRPNRVAGSGYWKATGTDKIITADGRRVGIKKALVFYAGKAPKGTKTNWIMHEYRLIEHSRSHGSSKLDDWVLCRIYKKTSGSQRQAVTPVQACREEHSTNGSSSSSSSQLDDVLDSFPEIKDQSFNLPRMNSLRTILNGNFDWASLAGLNPIPELAPTNGLPSYGGYDAFRAAEGEAESGHVNRQQNSSGLTQSFGYSSSGFGVSGQTFEFRQ
AT3G15510.1	MESTDSSGGPPPPQPNLPPGFRFHPTDEELVVHYLKRKAASAPLPVAIIAEVDLYKFDPWELPAKASFGEQEWYFFSPRDRKYPNGARPNRAATSGYWKATGTDKPVLASDGNQKVGVKKALVFYSGKPPKGVKSDWIMHEYRLIENKPNNRPPGCDFGNKKNSLRLDDWVLCRIYKKNNASRHVDNDKDHDMIDYIFRKIPPSLSMAAASTGLHQHHHNVSRSMNFFPGKFSGGGYGIFSDGGNTSIYDGGGMINNIGTDSVDHDNNADVVGLNHASSSGPMMMANLKRTLPVPYWPVADEEQDASPSKRFHGVGGGGGDCSNMSSSMMEETPPLMQQQGGVLGDGLFRTTSYQLPGLNWYSS
AT5G04395.1	MARDSKKAGGALPAPATAMGNAAAETSLPPGFRFHPSDEELISYYLKKKVQGKPMRYDEIGEVDICKLEPWDLAVIIFLCFLALDFRYVLKTRDKEWFFFSALDKKTRTGTSMSRATKQGYWKVTGTDGKIRQGGDGKVTIGTMKTLVFHRGRSPNGLGTDWVMNEYHLAKNDEGVPVRYMFSTRSF

AT5G46590.1	MGSSCLPPGFRFHPTDEELIEYYLKRKVEGLEIELEVIPVIDLYSFDP WELPDKSFLPNRDMEWYFFCSRDKKYPNGFRTNRGTKAGYWKA TGKDRKITSRSSSIIGYRKTLVFYKGRAPLGDRSNWIMHEYRLCDD DTSQGSQNLKGAFVLCRVAMKNEIKTNTKIRKIPSEQTIGSGESSG LSSRVTSPSRDETMPFHSFANPVSTETDSSNIWISPEFILDSSKDYPQI QDVASQCFQQDFDFPIIGNQNMEFPASTSLDQNMDEFMQNGYW TNYGYDQTGLFGYSDFS
AT5G46590.2	MGSSCLPPGFRFHPTDEELIEYYLKRKVEGLEIELEVIPVIDLYSFDP WELPDKSFLPNRDMEWYFFCSRDKKYPNGFRTNRGTKAGYWKA TGKDRKITSRSSSIIGYRKTLVFYKGRAPLGDRSNWIMHEYRLCDD DTSQGSQNLKNEIKTNTKIRKIPSEQTIGSGESSGLSSRVTSPSRDET MPFHSFANPVSTETDSSNIWISPEFILDSSKDYPQIQDVASQCFQQD FDFPIIGNQNMEFPASTSLDQNMDEFMQNGYWTNYGYDQTGLF GYSDFS
AT1G01010.1	MEDQVGFGFRPNDEELVGHYLRNKIEGNTSRDVEVAISEVNICSY DPWNLRFQSKYKSRDAMWYFFSRRENNKGNRQSRTTVSGKWKL TGESVEVKDQWGFCSEGFRGKIGHKRVLVFLDGRYPDKTKSDWV IHEFHYDLLPEHQRTYVICRLEYKGDDADILSAYAIDPTPAFVPNM TSSAGSVVNQSRQRNSGSYNTYSEYDSANHGQQFNENSNIMQQQ PLQGSFNPLLEYDFANHGGQWLSDYIDLQQQVPYLAPYENESEM IWKHVIEENFEFLVDERTSMQQHYSDHRPKKPVSGVLPDDSSDTE TGSMIFEDTSSSTDSVGSSDEPGHTRIDDIPSLNIIEPLHNYKAQEQP KQQSKEKVISSQKSECEWKMAEDSIKIPPSTNTVKQSWIVLENAQ WNYLKNMIIGVLLFISVISWIILVG
AT5G53950.1	MDIPYYHYDHGGDSQYLPPGFRFHPTDEELITHYLLRKVLDGCFS SRAIAEVDLNKCEPWQLPGRAKMGEKEWYFFSLRDRKYPTGLRT NRATEAGYWKATGKDREIFSSKTCALVGMKKTLVFYKGRAPKGE KSNWVMHEYRLEGKFSYHFISRSSKDEWVISRVFQKTTLASTGAV SEGGGGGGATVSVSSGTGPSKKTKVPSTISRNYQEQPSSPSSVSLPP LLDPTTTLGYTDSSCSYDSRSTNTTVTASAITEHVSCFSTVPTTTTA LGLDVNSFSRLPPPLGFDFDPFPRFVSRNVSTQSNFRSFQENFNQF PYFGSSSASTMTSAVNLPSFQGGGGVSGMNYWLPATAEENESKV GVLHAGLDCIWNY
AT1G52890.1	MGIQETDPLTQLSLPPGFRFYPTDEELMVQYLCRKAAGYDFSLQLI AEIDLYKFDPWVLPNKALFGEKEWYFFSPRDRKYPNGSRPNRVA GSGYWKATGTDKIISTEGQRVGIKKALVFYIGKAPKGTKTNWIMH EYRLIEPSRRNGSTKLDDWVLCRIYKKQSSAQKQVYDNGIANARE FSNNGTSSTTSSSSHFEDVLDSFHQEIDNRNFQFSNPNRISSLRPDL TEQKTGFHGLADTSNFDWASFAGNVEHNNSVPELGMSHVVPNL EYNCGYLKTEEEVESSHGFNNSGELAQKGYGVDSFGYSGQVGGF GFM

AT1G33060.2	MNQIKNKTLPEMTTEQALLSMEALPLGFRFRPTDEELINHYLRLKI NGRDLEVRVIPEIDVCKWEPWDLPGLSVIKTDDQEWFFFCPRDRK YPSGHRSNRATDIGYWKATGKDRTIKSKKMIIGMKKTLVFYRGRA PRGERTNWIMHEYRATDKELDGTGPGQNPYVLCRLFHKPSDSCD PAHCEEIEKVNFTPTTTTRCSPDDTSSEMVQETATSGVHALDRSD DTERCLSDKGNNDVKPDVSVINNTSVNHAETSRAKDRNLGKTLV EENPLLRDVPTLHGPILSEKSYYPGQSSIGFATSHMDSMYSSDFGN CDYGLHFQDGASEQDASLTDVLDEVFHNHNESSNDRKDFVLPN MMHWPGNTRLLSTEYPFLKDSVAFVDGSAEVSGSQQFVPDILASR WVSEQNVDSKEAVEILSSTGSSRTLTPLHNNVFGQYASSSYAAIDP FNYNVNQPEQSSFEQSHVDRNISPSNIFEFKARSRENQRDLDSVV DQGTAPRRIRLQIEQPLTPVTNKKERDADNYEEEDEVQSAMSKV VEEEPANLSAQGTAQRRIRLQTRLRKPLITLNNTKRNSNGREGEA SHRKCEMQEKEDISSSSSWQKQKKSLVQFSSVVIIVAVIVVLVEIW KESRDAKCSFLFHQLDSFKGMFT
AT1G33060.1	MNQIKNKTLPEMTTEQALLSMEALPLGFRFRPTDEELINHYLRLKI NGRDLEVRVIPEIDVCKWEPWDLPGLSVIKTDDQEWFFFCPRDRK YPSGHRSNRATDIGYWKATGKDRTIKSKKMIIGMKKTLVFYRGRA PRGERTNWIMHEYRATDKELDGTGPGQNPYVLCRLFHKPSDSCD PAHCEEIEKVNFTPTTTTRCSPDDTSSEMVQETATSGVHALDRSD DTERCLSDKGNNDVKPDVSVINNTSVNHAETSRAKDRNLGKTLV EENPLLRDVPTLHGPILSEKSYYPGQSSIGFATSHMDSMYSSDFGN CDYGLHFQDGASEQDASLTDVLDEVFHNHNESSNDRKDFVLPN MMHWPGNTRLLSTEYPFLKDSVAFVDGSAEVSGSQQFVPDILASR WVSEQNVDSKEAVEILSSTGSSRTLTPLHNNVFGQYASSSYAAIDP FNYNVNQPEQSSFEQSHVDRNISPSNIFEFKARSRENQRDLDSVV DQGTAPRRIRLQIEQPLTPVTNKKERDADNYEEEDEVQSAMSKV VEEEPANLSAQGTAQRRIRLQTRLRKPLITLNNTKRNSNGREGEA SHRKEKEDISSSSSWQKQKKSLVQFSSVVIIVAVIVVLVEIWKESRD AKCSFLFHQLDSFKGMFT
AT3G56530.1	MAIPQRNKRKARSSPERLTQPPELPHNSDVPSSSSSSAADNFFSWS TKQFAFPPGYRFVPKDQELIFHYLKPFSQGNKCSLLNVPIHRVNIY ESNPQHLSEKYEKGNDKDWFYISERTKTGKAGRSNKRVDNGGY WSATVAAQKINAGNGIVGYKTSLEYYVGKQSNSVKGDWLMQEY WFESSDDNNNEKVDHALCKIYLTPAAAKKKKAEEAENEKLKKE EDVEQLDLNQPDQLQLQQPHDIVYQPQYCLLPEHHQPQPFPDNF SELISFQQQPVMIPDDFEDFLAEFTKPHSLDGDEEFNNYGLFEGFF DTEGMIKH

AT1G61110.1	MENMGDSSIGPGHPHLPPGFRFHPTDEELVVHYLKKKADSVPLP VSIIAEIDLYKFDPWELPSKASFGEHEWYFFSPRDRKYPNGVRPNR AATSGYWKATGTDKPIFTCNSHKVGVKKALVFYGGKPPKGIKTD WIMHEYRLTDGNLSTAAKPPDLTTTRKNSLRLDDWVLCRIYKKN SSQRPTMERVLLREDLMEGMLSKSSANSSSTSVLDNNDNNNNNN EEHFFDGMVVSSDKRSLCGQYRMGHEASGSSSFGSFLSSKRFHHT GDLNNDNYNVSFVSMLSEIPQSSGFHANGVMDTTSSLADHGVLR QAFQLPNMNWHS
AT5G22290.2	MGYWKATGKERDVKSGSEVIGTKRTLVFHIGRAPKGERTDWIMH EYCVKGVSLDDAMVVCRVRRNKEYNSGTSQKAPKPNSSAEKHA KVQNGATSSGSPSDWDNLVDFYLAGESGEKLLAEMAESSENLQV DNDEDFFADILRDEIINLDEAVMTGNTPNEVPTLESASMEIRVLPL PNMIDKQMSSLLEERPSQKKKGKDATESLSSCFVGLYSIKSVNKAR WDVIIGVVALIAMLFYLE
AT5G22290.1	MDTKAVGVSKDTAASMEASTVFPGFKFSPTDVELISYYLKRKMDG LERSVEVIPDLEIYNFEPWDLPDKSIVKSDSEWFFFCARGKKYPHG SQNRRATKMGYWKATGKERDVKSGSEVIGTKRTLVFHIGRAPKG ERTDWIMHEYCVKGVSLDDAMVVCRVRRNKEYNSGTSQKAPKP NSSAEKHAKVQNGATSSGSPSDWDNLVDFYLAGESGEKLLAEM AESSENLQVDNDEDFFADILRDEIINLDEAVMTGNTPNEVPTLES ASMEIRVLPLPNMIDKQMSSLLEERPSQKKKGKDATESLSSCFVGL YSIKSVNKARWDVIIGVVALIAMLFYLE
AT1G32870.2	MDLSVENGGLAPGFRFHPTDEELVVYYLKRKIRRKKLRVEAIGET DVYKFDPEELPEKALYKTRDRQWFFFSLRDRKHGSRSSRATERGY WKATGKDRVIHCDSRPVGEKKTLVFHRGRAPNGERTNWVMHE YTLHKEELKRCGGEDVKDAYVLYKIYKKSGSGPKNGEQYGAPFIE EEWAEDDDDDVDEPANQLVVSASVDNSLWGKGLNQSELDDND IEELMSQVRDQSGPTLQQNGVSGLNSHVDTYNLENLEEDMYLEI NDLMEPEPEPTSVEVMENNWNEDGSGLLNDDDFVGADSYFLDL GVTNPQLDFVSGDLKNGFAQSLQVNTSLMTYQANNNQFQQQS GKNQASNWPLRNSYTRQINNGSSWVQELNNDGLTVTRFGDSSEF LNPVPSGISTTNEDDPSKDESSKFASSVWTFLESIPAKPAYASENPF VKLNLVRMSTSGGRFRFTSKSTGNNVVVMDSDSAVKRNKSGGN NDKKKKKNKGFFCLSIIGALCALFWVIIGTMGGSGRPLLW

AT1G32870.1	MDLSVENGGLAPGFRFHPTDEELVVYYLKRKIRRKKLRVEAIGET DVYKFDPEELPEKALYKTRDRQWFFFSLRDRKHGSRSSRATERGY WKATGKDRVIHCDSRPVGEKKTLVFHRGRAPNGERTNWVMHE YTLHKEELKRCGGEDVKDAYVLYKIYKKSGSGPKNGEQYGAPFIE EEWAEDDDDDVDEPANQLVVSASVDNSLWGKGLNQSELDDND IEELMSQVRDQSGPTLQQNGVSGLNSHVDTYNLENLEEDMYLEI NDLMEPEPEPTSVEVMENNWNEDGSGLLNDDDFVGADSYFLDL GVTNPQLDFVSGDLKNGFAQSLQVNTSLMTYQANNNQFQQQS GKNQASNWPLRNSYTRQINNGSSWVQELNNDGLTVTRFGEAPG TGDSSEFLNPVPSGISTTNEDDPSKDESSKFASSVWTFLESIPAKPA YASENPFVKLNLVRMSTSGGRFRFTSKSTGNNVVVMDSDSAVKR NKSGGNNDKKKKKNKGFFCLSIIGALCALFWVIIGTMGGSGRPLL W
AT1G32870.3	MKNKIFPQNKTLLPFGTEKSKLFRVFFFVITREKKVMDLSVENGGL APGFRFHPTDEELVVYYLKRKIRRKKLRVEAIGETDVYKFDPEELP EKALYKTRDRQWFFFSLRDRKHGSRSSRATERGYWKATGKDRVI HCDSRPVGEKKTLVFHRGRAPNGERTNWVMHEYTLHKEELKRC GGEDVKDAYVLYKIYKKSGSGPKNGEQYGAPFIEEEWAEDDDDD VDEPANQLVVSASVDNSLWGKGLNQSELDDNDIEELMSQVRDQ SGPTLQQNGVSGLNSHVDTYNLENLEEDMYLEINDLMEPEPEPTS VEVMENNWNEDGSGLLNDDDFVGADSYFLDLGVTNPQLDFVSG DLKNGFAQSLQVNTSLMTYQANNNQFQQQSGKNQASNWPLRN SYTRQINNGSSWVQELNNDGLTVTRFGEAPGTGDSSEFLNPVPSG ISTTNEDDPSKDESSKFASSVWTFLESIPAKPAYASENPFVKLNLVR MSTSGGRFRFTSKSTGNNVVVMDSDSAVKRNKSGGNNDKKKKK NKGFFCLSIIGALCALFWVIIGTMGGSGRPLLW
AT2G24430.1	MEQGDHQQHKKEEEALPPGFRFHPTDEELISYYLVNKIADQNFT GKAIADVDLNKSEPWELPEKAKMGGKEWYFFSLRDRKYPTGVRT NRATNTGYWKTTGKDKEIFNSTTSELVGMKKTLVFYRGRAPRGE KTCWVMHEYRLHSKSSYRTSKQDEWVVCRVFKKTEATKKYISTSS SSTSHHHNNHTRASILSTNNNNPNYSSDLLQLPPHLQPHPSLNIN QSLMANAVHLAELSRVFRASTSTTMDSSHQQLMNYTHMPVSGL NLNLGGALVQPPPVVSLEDVAAVSASYNGENGFGNVEMSQCMD LDGYWPSY
AT2G24430.2	MEQGDHQQHKKEEEALPPGFRFHPTDEELISYYLVNKIADQNFT GKAIADVDLNKSEPWELPEKAKMGGKEWYFFSLRDRKYPTGVRT NRATNTGYWKTTGKDKEIFNSTTSELVGMKKTLVFYRGRAPRGE KTCWVMHEYRLHSKSSYRTSKQDEWVVCRVFKKTEATKKYISTSS SSTSHHHNNHTRASILSTNNNNPNYSSDLLQLPPHLQPHPSLNIN QSLMANAVHLAELSRVFRASTSTTMDSSHQQLMNYTHMPVSGL NLNLGGALVQPPPVVSLEDVAAVSASYNGENGFGNVEMSQCMD LDGYWPSY

AT5G18270.2	MAVVVEEGVVLNHGGEELVDLPPGFRFHPTDEEIITCYLKEKVLN SRFTAVAMGEADLNKCEPWDLPSKAKMGEKEFYFFCQRDRKYPT GMRTNRATESGYWKATGKDKEIFKGKGCLVGMKKTLVFYRGRA PKGEKTNWVMHEYRLEGKYSYYNLPKSARDEWVVCRVFHKNN PSTTTQPMTRIPVEDFTRMDSLENIDHLLDFSSLPPLIDPSFMSQTE QPNFKPINPPTYDISSPIQPHHFNSYQSIFNHQVFGSASGSTYNNN NEMIKMEQSLVSVSQETCLSSDVNANMTTTTEVSSGPVMKQEMG MMGMVNGSKSYEDLCDLRGDLWDF
AT5G18270.1	MAVVVEEGVVLNHGGEELVDLPPGFRFHPTDEEIITCYLKEKVLN SRFTAVAMGEADLNKCEPWDLPKRAKMGEKEFYFFCQRDRKYP TGMRTNRATESGYWKATGKDKEIFKGKGCLVGMKKTLVFYRGR APKGEKTNWVMHEYRLEGKYSYYNLPKSARDEWVVCRVFHKN NPSTTTQPMTRIPVEDFTRMDSLENIDHLLDFSSLPPLIDPSFMSQT EQPNFKPINPPTYDISSPIQPHHFNSYQSIFNHQVFGSASGSTYNN NNEMIKMEQSLVSVSQETCLSSDVNANMTTTTEVSSGPVMKQEM GMMGMVNGSKSYEDLCDLRGDLWDF
AT2G02450.1	MAIVSSTTSIIPMSNQVNNNEKGIEDNDHRGGQESHVQNEDEAD DHDHDMVMPGFRFHPTEEELIEFYLRRKVEGKRFNVELITFLDLY RYDPWELPAMAAIGEKEWYFYVPRDRKYRNGDRPNRVTTSGYW KATGADRMIRSETSRPIGLKKTLVFYSGKAPKGTRTSWIMNEYRLP HHETEKYQKAEISLCRVYKRPGVEDHPSVPRSLSTRHHNHNSSTS SRLALRQQQHHSSSSNHSDNNLNNNNNINNLEKLSTEYSGDGST TTTTTNSNSDVTIALANQNIYRPMPYDTSNNTLIVSTRNHQDDDE TAIVDDLQRLVNYQISDGATTLMPQTQAALAMNMIPAGTIPNNA LWDMWNPIVPDGNRDHYTNIPFK
AT2G02450.2	MAIVSSTTSIIPMSNQVNNNEKGIEDNDHRGGQESHVQNEDEAD DHDHDMVMPGFRFHPTEEELIEFYLRRKVEGKRFNVELITFLDLY RYDPWELPAMAAIGEKEWYFYVPRDRKYRNGDRPNRVTTSGYW KATGADRMIRSETSRPIGLKKTLVFYSGKAPKGTRTSWIMNEYRLP HHETEKYQKAEISLCRVYKRPGVEDHPSVPRSLSTRHHNHNSSTS SRLALRQQQHHSSSSNHSDNNLNNNNNINNLEKLSTEYSGDGST TTTTTNSNSDVTIALANQNIYRPMPYDTSNNTLIVSTRNHQDDDE TAIVDDLQRLVNYQISDGGNINHQYFQIAQQFHHTQQQNANAN ALQLVAAATTATTLMPQTQAALAMNMIPAGTIPNNALWDMW NPIVPDGNRDHYTNIPFK
AT5G13180.1	MDNVKLVKNGVLRLPPGFRFHPTDEELVVQYLKRKVCSSPLPASII PEFDVCRADPWDLPGNLEKERYFFSTREAKYPNGNRSNRATGSG YWKATGIDKRVVTSRGNQIVGLKKTLVFYKGKPPHGSRTDWIMH EYRLSSSPPSSMGPTQNWVLCRIFLKKRAGNKNDDDDGDSRNLR HNNNNNNSSDQIEIITTDQTDDKTKPIFFDFMRKERTTDLNLLPSSP SSDHASSGVTTEIFSSSDEETSSCNSFR

AT5G56620.3	MKNSKCNLIDSKLEEHHHLCGSKHCPGCGRMIQAATKPNWVGL PAGVKFDPTDQELIEHLEAKVKGKEENKKWSSSHPLIDEFIPTIDG EDGICYTHPQKLPGVTRDGLSKHFFHKPSRAYTTGTRKRRKIIQTD HDSELTGSSETRWHKTGKTRPVMINGQQRGCKKILVLYTNFGKN RRPEKTNWVMHQYHLGINEEEREGELVVSKIFYQTQPRQCVSNT NWSDHHGSKDVIGIGVGDEISSVAATLQSLGSGDVVSRVNMHPH TRSFDEGTAEASKGRENQHVSGTCEEVHDGIITSSMSSHHMIHDH HNQHHQIGDRREFHMSSSYPMTPTITSQHESIFHVTSTMPFQRQQ LRGRSSGSGLEDLIMGCTTATCTEDEHSEANPQRNAEWLTFPQF WNQAESDDQNRRF
AT5G56620.2	MKNSKCNLIDSKLEEHHHLCGSKHCPGCGRMIQAATKPNWVGL PAGVKFDPTDQELIEHLEAKVKGKEENKKWSSSHPLIDEFIPTIDG EDGICYTHPQKLPGVTRDGLSKHFFHKPSRAYTTGTRKRRKIIQTD HDSELTGSSETRWHKTGKTRPVMINGQQRGCKKILVLYTNFGKN RRPEKTNWVMHQYHLGINEEEREGELVVSKIFYQTQPRQCVSNT NWSDHHGSKDVIGIGVGDEISSVAATLQSLGSGDVVSRVNMHPH TRSFDEGTAEASKGRENQHVSGTCEEVHDGIITSSMSSHHMIHDH HNQHHQIGDRREFHMSSSYPMTPTITSQHESIFHVTSTMPFQRQQ LRGRSSGSGLEDLIMGCTTATCTEDEHSEANPQRNAEWLTFPQF W
AT5G56620.1	MKNSKCNLIDSKLEEHHHLCGSKHCPGCGRMIQAATKPNWVGL PAGVKFDPTDQELIEHLEAKVKGKEENKKWSSSHPLIDEFIPTIDG EDGICYTHPQKLPGVTRDGLSKHFFHKPSRAYTTGTRKRRKIIQTD HDSELTGSSETRWHKTGKTRPVMINGQQRGCKKILVLYTNFGKN RRPEKTNWVMHQYHLGINEEEREGELVVSKIFYQTQPRQCVSNT NWSDHHGSKDVIGIGVGDEISSVAATLQSLGSGDVVSRVNMHPH TRSFDEGTAEASKGRENQHVSGTCEEVHDGIITSSMSSHHMIHDH HNQHHQIGDRREFHMSSSYPMTPTITSQHESIFHVTSTMPFQGSV VWFGIRRPNYGLYHSYVYRRRKFLTNLCI
AT5G39690.1	MAKKEKIEQVISMGGIMWEGLNSSLIKVDEALLKQQIREFEKGND KEWFIITERNKVDQGLSQTKRVGNGAKRQKRVDTNGGYWHATV AAQKINAGDGVVGNKRPLAYYDRKPSEDVKTDWLMQEYSLDH NNDKVRLHFVQDLSYSTSNKEVGEEKKKQKKGEPVEASEGQQPC NAEYHQPLAPLDSCQPQPHDLAEQLDLHQPEQLQLQQPHDIVY QPQYCLLPEQHQLQPFPDNFSELNSFQQQPVMIPDDLEDFLAEL MEPHSLDGDEESNNYGFFEGLFDTEGINDKTLH
AT1G33280.1	MSSSNGGVPPGFRFHPTDEELLHYYLKKKISYEKFEMEVIKEVDLN KIEPWDLQDRCKIGSTPQNEWYFFSHKDRKYPTGSRTNRATHSGF WKATGRDKCIRNSYKKIGMRKTLVFYKGRAPHGQKTDWIMHEY RIEDTEDDPCEDGWVVCRVFKKKNLFKVGNDVGSNISNNRLEAR SFIRRESPYQGISMFELNKPEEISVHQYPQPPMFQPHHKPLSIGYDY SLALLPRESEYQQACQPSGVEVGTCKAVSEWGIVNCNMVSHEDS SRAMRFEDDGNNTSSTVQPPSNLLSLRGENGFLGLF

AT3G04070.1	MISKDPRSSLPPGFRFHPTDEELILHYLRKKVSSSPVPLSIIADVDIY KSDPWDLPAKAPFGEKEWYFFSPRDRKYPNGARPNRAAASGYW KATGTDKLIAVPNGEGFHENIGIKKALVFYRGKPPKGVKTNWIM HEYRLADSLSPKRINSSRSGGSEVNNNFGDRNSKEYSMRLDDWV LCRIYKKSHASLSSPDVALVTSNQEHEENDNEPFVDRGTFLPNLQ NDQPLKRQKSSCSFSNLLDATDLTFLANFLNETPENRSESDFSFMI GNFSNPDIYGNHYLDQKLPQLSSPTSETSGIGSKRERVDFAEETIN ASKKMMNTYSYNNSIDQMDHSMMQQPSFLNQELMMSSHLQYQ G
AT3G04070.2	MISKDPRSSLPPGFRFHPTDEELILHYLRKKVSSSPVPLSIIADVDIY KSDPWDLPAKAPFGEKEWYFFSPRDRKYPNGARPNRAAASGYW KATGTDKLIAVPNGEGFHENIGIKKALVFYRGKPPKGVKTNWIM HEYRLADSLSPKRINSSRSGGSELDDWVLCRIYKKSHASLSSPDVA LVTSNQEHEENDNEPFVDRGTFLPNLQNDQPLKRQKSSCSFSNLL DATDLTFLANFLNETPENRSESDFSFMIGNFSNPDIYGNHYLDQK LPQLSSPTSETSGIGSKRERVDFAEETINASKKMMNTYSYNNSIDQ MDHSMMQQPSFLNQELMMSSHLQYQG
AT3G10500.1	MGRGSVTSLAPGFRFHPTDEELVRYYLKRKICNKPFKFDAISVTDV YKSEPWDLPDKSRLKSRDLEWYFFSMLDKKYRNGSKTNRATEMG YWKTTGKDREILNGSKVVGMKKTLVYHKGRAPRGERTNWVMH EYRLVDQDLDKTGVHQDAFVLCRIFQKSGSGPKNGEQYGAPFVE EEWEEEDDMTFVPDQEDLGSEDHVYVHMDDIDQKSENFVVYDA IPIPLNFIHGESSNNVETNYSDSINYIQQTGNYMDSGGYFEQPAES YEKDQKPIIRDRDGSLQNEGIGCGVQDKHSETLQSSDNIFGTDTSC YNDFPVESNYLIGEAFLDPNSNLLENDGLYLETNDLSSTQQDGFD FEDYLTFFDETFDPSQLMGNEDVFFDQEELFQEVETKELEKEETSR SKHVVEEKEKDEASCSKQVDADATEFEPDYKYPLLKKASHMLGA IPAPLANASEFPTKDAAIRLHAAQSSGSVHVTAGMITISDSNMGW SYGKNENLDLILSLGLVQGNTAPEKSGNSSAWAMLIFMCFWVLL LSVSFKVSILVSSR
AT4G01520.1	MMKDPTGYRFSPTGEEVINHYLKNKILGKTWLVDEAISEINILNH KPSKDLPKLARIQSEDLEWYFFSPIEYTNPNKMKMKRTTGSGFWK PSGVDRKIRDKRGNGVVIGIKKTLVYHEGKSPHGVRTPWVMHEY HITCLPHHKRKYVVCQVKYKGEAAEISYEPSPSLVSDSHTVIAING EPEPELQVEQPGKENLLGMSVDDLIEPMNQQEEPQGPHLAPNDD EFIRGLRHVDREPVEYLFANEENMDGLSIMNDLTIPMIAQQEDLIL SEWEGFIAATFFSDNNNNNNLNVHQLTSFLPG

AT4G35580.3	MGAVSMESLPLGFRFRPTDEELVNHYLRLKINGRHSDVRVIPDID VCKWEPWDLPALSVIKTDDPEWFFFCPRDRKYPNGHRSNRATDS GYWKATGKDRSIKSKKTLIGMKKTLVFYRGRAPKGERTNWIMHE YRPTLKDLDGTSPGQSPYVLCRLFHKPDDRVNGVKSDEAAFTAS NKYSPDDTSSDLVQETPSSDAAVEKPSDYSGGCGYAHSNSTADGT MIEAPEENLWLSCDLEDQKAPLPCMDSIYAGDFSYDEIGFQFQDG TSEPDVSLTELLEEVFNNPDDFSCEESISRENPAVSPNGIFSSAKML QSAAPEDAFFNDFMAFTDTDAEMAQLQYGSEGGASGWPSDTNS YYSDLVQQEQMINHNTENNLTEGRGIKIRARQPQNRQSTGLINQ GIAPRRIRLQLQSNSEVKEREEVNEGHTVIPEAKEAAAKYSEKSGS LVKPQIKLRARGTIGQVKGERFADDEFVVVTGTGAEQKETRREA MEGGCNGNGGCDGWGRDGYMEDTGEFMTHETQRERERESNVV
AT4G35580.1	MGAVSMESLPLGFRFRPTDEELVNHYLRLKINGRHSDVRVIPDID VCKWEPWDLPALSVIKTDDPEWFFFCPRDRKYPNGHRSNRATDS GYWKATGKDRSIKSKKTLIGMKKTLVFYRGRAPKGERTNWIMHE YRPTLKDLDGTSPGQSPYVLCRLFHKPDDRVNGVKSDEAAFTAS NKYSPDDTSSDLVQETPSSDAAVEKPSDYSGGCGYAHSNSTADGT MIEAPEENLWLSCDLEDQKAPLPCMDSIYAGDFSYDEIGFQFQDG TSEPDVSLTELLEEVFNNPDDFSCEESISRENPAVSPNGIFSSAKML QSAAPEDAFFNDFMAFTDTDAEMAQLQYGSEGGASGWPSDTNS YYSDLVQQEQMINHNTENNLTEGRGIKIRARQPQNRQSTGLINQ GIAPRRIRLQLQSNSEVKEREEVNEGHTVIPEAKEAAAKYSEKSGS LVKPQIKLRARGTIGQVKGERFADDEVQVQSRKRRGGKRWKVV ATVMVAVMVGVGMGIWRTLVSS
AT4G35580.2	MGAVSMESLPLGFRFRPTDEELVNHYLRLKINGRHSDVRVIPDID VCKWEPWDLPALSVIKTDDPEWFFFCPRDRKYPNGHRSNRATDS GYWKATGKDRSIKSKKTLIGMKKTLVFYRGRAPKGERTNWIMHE YRPTLKDLDGTSPGQSPYVLCRLFHKPDDRVNGVKSDEAAFTAS NKYSPDDTSSDLVQETPSSDAAVEKPSDYSGGCGYAHSNSTADGT MIEAPEENLWLSCDLEDQKAPLPCMDSIYAGDFSYDEIGFQFQDG TSEPDVSLTELLEEVFNNPDDFSCEESISRENPAVSPNGIFSSAKML QSAAPEDAFFNDFMAFTDTDAEMAQLQYGSEGGASGWPSDTNS YYSDLVQQEQMINHNTENNLTEGRGIKIRARQPQNRQSTGLINQ GIAPRRIRLQLQSNSEVKEREEVNEGHTVIPEAKEAAAKYSEKSGS LVKPQIKLRARGTIGQVKGERFADDEKFVVVTGTGAEQKETRREA MEGGCNGNGGCDGWGRDGYMEDTGEFMTHETQRERERESNVV

AT1G26870.1	MGDRNNDGDQKMEDVLLPGFRFHPTDEELVSFYLKRKVQHNPL SIELIRQLDIYKYDPWDLPKFAMTGEKEWYFYCPRDRKYRNSSRP NRVTGAGFWKATGTDRPIYSSEGNKCIGLKKSLVFYKGRAAKGV KTDWMMHEFRLPSLSEPSPPSKRFFDSPVSPNDSWAICRIFKKTNT TTLRALSHSFVSSLPPETSTDTMSNQKQSNTYHFSSDKILKPSSHFQ FHHENMNTPKTSNSTTPSVPTISPFSYLDFTSYDKPTNVFNPVSCL DQQYLTNLFLATQETQPQFPRLPSSNEIPSFLLNTSSDSTFLGEFTS HIDLSAVLAQEQCPPLVSLPQEYQETGFEGNGIMKNMRGSNEDH LGDHCDTLRFDDFTSTINENHRHHQDLKQNMTLLESYYSSLSSIN SDLPACFSSTT
AT5G24590.2	MKEDMEVLSLASLPVGFRFSPTDEELVRYYLRLKINGHDNDVRVI REIDICKWEPWDLPDFSVVKTTDSEWLFFCPLDRKYPSGSRMNRA TVAGYWKATGKDRKIKSGKTKIIGVKRTLVFYTGRAPKGTRTCWI MHEYRATEKDLDGTKSGQNPFVVCKLFKKQDIVNGAAEPEESKS CEVEPAVSSPTVVDEVEMSEVSPVFPKTEETNPCDVAESSLVIPSEC RSGYSVPEVTTTGLDDIDWLSFMEFDSPKLFSPLHSQVQSELGSSF NGLQSESSELFKNHNEDYIQTQYGTNDADEYMSKFLDSFLDIPYE PEQIPYEPQNLSSCNKINDESKRGIKIRARRAQAPGCAEQFVMQG DASRRLRLQVNLNSHKSETDSTQLQFIKKEVKDTTTETMTKGCG NFTRSKSRTSFIFKKIAAMGCSYRGLFRVGVVAVVCVMSVCSLVA
AT5G64530.1	MNLPPGFRFFPTDEELVVHFLHRKASLLPCHPDVIPDLDLYHYDP WDLPGKALGEGRQWYFYSRKTQERVTSNGYWGSMGMDEPIYTS STHKKVGIKKYLTFYLGDSQTNWIMQEYSLPDSSSSSSRSSKRSSRA SSSSHKPDYSKWVICRVYEQNCSEEEDDDGTELSCLDEVFLSLDDL DEVSLP
AT5G64530.2	MNLPPGFRFFPTDEELVVHFLHRKASLLPCHPDVIPDLDLYHYDP WDLPGKALGEGRQWYFYSRKTQERVTSNGYWGSMGMDEPIYTS STHKKVGIKKYLTFYLGDSQTNWIMQEYSLPDSSSSSSRSSKRSSRA SSSSHKPVSTLFCFISLKLSALT
AT3G10490.3	MNRATNRGYWKATGKDREIRRDILLLGMKKTLVFHSGRAPDGL RTNWVMHEYRLVEYETEKNGNLVQDAYVLCRVFHKNNIGPPSG NRYAPFMEEEWADDEGALIPGIDVKLRLEPPPVANGNDQMDQEI QSASKSLININEPPRETAPLDIESDQQNHHENDLKPEEHNNNNN YDENEETLKREQMEEEERPPRPVCVLNKEAPLPLLQYKRRRQSES NNNSSRNTQDHCSSTTTTVDNTTTLISSSAAATNTAISALLEFSLM GISDKKEKPQQPLRPHKEPLPPQTPLASPEEKVNDLQKEIHQMSV ERETFKLEMMSAEAMISILQSRIDALRQENEELKKNNANGQ
AT3G10490.1	MGRESVAVVTAPPSATAPGTASVATSLAPGFRFHPTDEELVSYYL KRKVLGQPVRFDAIGEVDIYKHEPWDLAVFSRLKTRDQEWYFYS ALDKKYGNGARMNRATNRGYWKATGKDREIRRDILLLGMKKTL VFHSGRAPDGLRTNWVMHEYRLVEYETEKNGNLVQDAYVLCRV FHKNNIGPPSGNRYAPFMEEEWADDEGALIPGIDVKLRLEPPPVA NGNDQMDQIIYASSGVHL

AT3G10490.4	MNRATNRGYWKATGKDREIRRDILLLGMKKTLVFHSGRAPDGLRTNWVMHEYRLVEYETEKNGNLVQDAYVLCRVFHKNNIGPPSGNRYAPFMEEEWADDEGALIPGIDVKLRLEPPPVANGNDQMDQEIQSASKSLININEPPRETAPLDIESDQQNHHENDLKPEEHNNNNNYDENEETLKREQMEEEERPPRPVCVLNKEAPLPLLQYKRRRQSESNNNSSRNTQDHCSSTTTTVDNTTTLISSSAAATNTAISALLEFSLMGISDKKEKPQQPLRPHKEPLPPQTPLASPEEKVNDLQKEIHQMSVERETFKLEMMSAEAMISILQSRIDALRQENEELKKNNANGQ
AT3G10490.2	MGRESVAVVTAPPSATAPGTASVATSLAPGFRFHPTDEELVSYYLKRKVLGQPVRFDAIGEVDIYKHEPWDLAVFSRLKTRDQEWYFYSALDKKYGNGARMNRATNRGYWKATGKDREIRRDILLLGMKKTLVFHSGRAPDGLRTNWVMHEYRLVEYETEKNGNLVQDAYVLCRVFHKNNIGPPSGNRYAPFMEEEWADDEGALIPGIDVKLRLEPPPVANGNDQMDQEIQSASKSLININEPPRETAPLDIESDQQNHHENDLKPEEHNNNNNYDENEETLKREQMEEEERPPRPVCVLNKEAPLPLLQYKRRRQSESNNNSSRNTQDHCSSTTTTVDNTTTLISSSAAATNTAISALLEFSLMGISDKKEKPQQPLRPHKEPLPPQTPLASPEEKVNDLQKEIHQMSVERETFKLEMMSAEAMISILQSRIDALRQENEELKKNNANGQ
AT2G42400.1	MSNHPKITSAHQNVEEKLRELQERFCHLQAARKEGRHGDLALLEAQISQNIREWQAELTAPSPESSLLGEGISQFLEEFAPLLKLDEEDDATSTLKEHAGAKPDPEGFSQSLCPPEWTSENFSQSPFNGNFSCGFEDALNSTETHGQQLHYGYEGFDPSINSAPDFHDQKLSSNLDITSQYDYIFSEVRQELDNSPSTKLDSSEEIDNFAEFSTPSSVRVPPSAFLGPKCALWDCTRPAQGSEWYLDYCSNYHGTLALNEDSPGTAPVLRPGGISLKDNLLIDALRAKTQGKNVGIPVCEGAVNTKCPWNAAELFHLELVEGETIREWLFFDKPRRAYDSGNRKQRSLPDYSGRGWHESRKQLMKEQEGQKRSYYMDPQPPGPFEWHLFEYQINESDACALYRLELKVGNGKKSPKGKISKDPLADLQKKMGQFKVASDKPSPPTKGRKE
AT1G34190.1	MADSSPDSCFKGGKFSAPGFRFHPTDEELVMYYLKRKICRKRLRVNVIGVVDVYKMDPEELPGQSMLKTGDRQWFYFTPRSRKYPNAARSNRGTENGYWKATGKDRVIEYNSRSVGLKKTLVFYRGRAPSGERTDWVMHEYTMDEDELGRCKNPQEYYALYKLFKKSGAGPKNGEQYGAPFQEEEWVDDDNEDVNAIAVAVPEQPVVRYEDARRVDERRLFNPVILQLEDIDELLNGIPNAPGVPQRCIPQVNSEEELQSTLVNNSAREFLPNGQQYNRPSSFDSLETAEVTSAPLVFEKEDFIEMDDLLLIPEFGASSTEKAAQFSNHGEFDDFNEFDQLFHDVSMSLDMEPIDQGTSANLSSLSDSANYTSDQKQQLLYQQFQDQTPENQLNNIMDPSTTLNQITSDIWFEDDQAILFDQQQSFSGAFASPSSGVMPDSTNPTMSVNAQGHEIQNGGGTTSQFSSALWALMDSIPSTPASACEGPLNRTFVRMSSFSRMRFNGKANGTPVSTTIAKKGIRNRGFLLLSIVGALCAIFWVLVATVRVSGRSLLLKD

AT1G54330.1	MAPMSLPPGFRFHPTDEELVAYYLDRKVNGQAIELEIIPEVDLYKC EPWDLPEKSFLPGNDMEWYFYSTRDKKYPNGSRTNRATRAGYW KATGKDRTVESKKMKMGMKKTLVYYRGRAPHGLRTNWVMHE YRLTHAPSSSLKESYALCRVFKKNIQIPKRKGEEEEAEEESTSVGKE EEEEKEKKWRKCDGNYIEDESLKRASAETSSSELTQGVLLDEANSS SIFALHFSSSLLDDHDHLFSNYSHQLPYHPPLQLQDFPQLSMNEA EIMSIQQDFQCRDSMNGTLDEIFSSSATFPASL
AT1G60300.1	MEDDDAAYDLIKHELLYSEDEVIISRYLKGMVVNGDSWPDHFIED ANVFTKNPDKVFNSERPRFVIVKPRTEACGKTDGCDSGCWRIIGR DKLIKSEETGKILGFKKILKFCLKRKPIDYKRSWVMEEYRLTNNLN WKQDHVICKIRFMFEAEISFLLSKHFYTTSESVLENELLPSYGYYLS NTQEEDEFYLDAIMTSEGNEWPSYVTNNVYCLHPLELVDLQDRM FNDYGTCIFANKTCGETDKCDGGYWKILHGDKLIKSNFGKVIGFK KVFEFYETVRQIYLCDGEEVTVTWTIQEYRLSKNVKQNKVLCVIK LTYDR
AT1G60380.1	MADTLLNAEDEVIISRYLKPMIVNRVSWPDLFIEDADVFNKDPYV KFHAEIPSFVIVKPRTKACGKTDGCDSGCWRIIGRDKLIKSEETGKI LGFKKILKFCLKWKPREYKRSLVMEEYRLTNNFNWKQDHVICKIR LLFEAEISFLLAKHFYTTSDSLPRNVLLPAYGFCSPDKQEEDEFYPV TIMISEGKDWPSYVTNNVYCLHPSELVNVHDGKFHDNGICIFAN RTCGVTDKCNEGYWKIKHREKLIMSRYGQTIGWKKVFQFYETEK ERHFGNGEEVKVTWTLKEYRLTRKMNKNKVVCVIKYKVKCLPRI TS
AT5G41090.1	MGPTYRALPVGMRFRPSDLELAVYFLIKKALGLPMKALTVPDEC NDIFSTHPRDLPGYGSEEHWYFYCKKPKNQVTRTKSYNLWIPTGE KTDVLDPKKNGGELVGIKHSFTFIENEEEEESDNKNGDEEEPPQC NWFLDEISLPLTVVDTDWTLCHIFYEKVKPEFGNLHIVESESESEEE EEDESVDKPAESLDSVKEKDGTVLPPPPATP
AT1G60340.1	MEDNDAAYDQVKSELLNSEDEVIISRYLKPMVVNGDSWPDHFIE DANVFTKNPNEVFNSERPRFVIVKPRTEACGKTDGCESGCWRTM GRDKLIKSEETGKILGFKKILKFCIKWKPIEYKRSWVMEEYRLTNN LNWKQDHVICKIRLLFEAEISFLLAKHFYTTSESLLRHELLPSYGYL SNTQEEDEFYLKTIMTSEGNDWPSYVTNNVYCLHPLELVDLQDV MFQSYGTCIFANGTCGESDKCDGGYWKILHGDKLIKSNFGMVTG FKKVFEFYETVIHRYFCDGEEVKEETERLGHQRCQGLLLPHRRGH VQTKSCGVVTVKEKRSLVEQPEEIVAAPETPRQGKSEHNDPLKRK KAIGCVGEKAEM

AT5G64060.1	MGKTNLAPGFRFHPTDVELVRYYLKRKVMGKKFQVDAIAEVDIYKFEPPDLPDKSCLGTGDLKWYFFCPREKKYPKGGKANRSTECGYWKTTGRDRDVSYNDEVTGKIRTLIYHYGKIPRGDRTDWVIHEYRLEDKVLAQKNVPQDTYVLCVLFKKNGLGPRHGSQYGAPFKEEDWSDKEEEYTQNHLVAGPSKETSLAAKASHSYAPKDGLTGVISESCVSDVPPLTATVLPPLTSDVIAYNPFSSSPLLEVPQVSLDGGELNSMLDLFSVDNDDCLLFDDFDYHNEVRHPDGFVNKEAPVFLGDGNFSGMFDLSNDQVVELQDLIQSPTPHPPSPPAQASIPDDSRSNGQTKDD
AT1G77450.1	MMKSGADLQFPPGFRFHPTDEELVLMYLCRKCASQPIPAPIITELDLYRYDPWDLPDMALYGEKEWYFFSPRDRKYPNGSRPNRAAGTGYWKATGADKPIGRPKPVGIKKALVFYSGKPPNGEKTNWIMHEYRLADVDRSVRKKNSLRLDDWVLCRIYNKKGVIEKRRSDIEDGLKPVTDTCPPESVARLISGSEQAVSPEFTCSNGRLSNALDFPFNYVDAIADNEIVSRLLGGNQMWSTTLDPLVVRQGTF
AT4G28500.1	MTWCNDRSDVQTVERIIPSPGAAESPVASLPVSCHKTCPSCGHNFKFHEQAGIHDLPGLPAGVKFDPTDQEVLEHLEGKVRDDAKKLHPLIDEFIRTIDGENGICYTHPEKLPGVNKDGTVRHFFHRPSKAYTTGTRKRRKVHTDSDVGGETRWHKTGKTRPVLAGGRVRGYKKILVLYTNYGKQKKPEKTNWVMHQYHLGTSEEEKEGELVVSKVFYQTQPRQCGGSVAAAATAKDRPYLHGLGGGGGRHLHYHLHHNNGNGKSNGSGGTAGAGEYYHNIPAIISFNQTGIQNHLVHDSQPFIP
AT3G03200.1	MAPVSLPPGFRFHPTDEELITYYLKRKINGLEIELEVIAEVDLYKCEPWDLPGKSLLPSKDQEWYFFSPRDRKYPNGSRTNRATKGGYWKATGKDRRVSWRDRAIGTKKTLVYYRGRAPHGIRTGWVMHEYRLDETECEPSAYGMQDAYALCRVFKKIVIEAKPRDQHRSYVHAMSNVSGNCSSSFDTCSDLEISSTTHQVQNTFQPRFGNERFNSNAISNEDWSQYYGSSYRPFPTPYKVNTEIECSMLQHNIYLPPLRVENSAFSDSDFFTSMTHNNDHGVFDDFTFAASNSNHNNSVGDQVIHVGNYDEQLITSNRHMNQTGYIKEQKIRSSLDNTDEDPGFHGNNTNDNIDIDDFLSFDIYNEDNVNQIEDNEDVNTNETLDSSGFEVVEEETRFNNQMLISTYQTTKILYHQVVPCHTLKVHVNPISHNVEERTLFIEEDKDSWLQRAEKITKTKLTLFSLMAQQYYKCLAIFF
AT1G32770.1	MADNKVNLSINGQSKVPPGFRFHPTEEELLHYYLRKKVNSQKIDLDVIREVDLNKLEPWDIQEECRIGSTPQNDWYFFSHKDKKYPTGTRTNRATVAGFWKATGRDKIICSCVRRIGLRKTLVFYKGRAPHGQKSDWIMHEYRLDDTPMSNGYADVVTEDPMSYNEEGWVVCRVFRKKNYQKIDDCPKITLSSLPDDTEEEKGPTFHNTQNVTGLDHVLLYMDRTGSNICMPESQTTTQHQDDVLFMQLPSLETPKSESPVDQSFLTPSKLDFSPVQEKITERPVCSNWASLDRLVAWQLNNGHHNPCHRKSFDEEEENGDTMMQRWDLHWNNDDNVDLWSSFTESSSSLDPLLHLSV

AT1G60240.1	MMKSRREQSIEEAIVANYLKMMIDNVNVWPRHFLRSEDVYCKNPWTLFVTRDPIILHFGRYFFVNRSVNSGLTDGCEYGCWRIIGRDRVIKSVTTGKILGLKKVYKFCETDRKPKSVFKFLEKEKRRVRDRRIWAMEEYRFASTWKQDYVICKIRRLYPQPFDYMLAQHIRGYYK
AT1G02210.1	MVWFFFSCDEYNEEILKTNSGYWKETVSNTPIIGKWITSNGVKIGEKQVLVFQSYENINGSKSDWVMHVYQPTFLPPNQVIFRIYDV
AT1G25580.1	MAGRSWLIDSNRIATKIMSASASSDPRQVVWKSNPSRHCPKCQHVIDNSDVVDDWPGLPRGVKFDPSDPEIIWHLLAKSGLSGLSSHPFIDEFIPTVNQDDGICYTHPKNLPGVKSDGTVSHFFHKAIKAYSTGTRKRRKIHDDDFGDVRWHKTGRTKPVVLDGVQRGCKKIMVLYGGKAVKTNWVMHQYHLGIEEDEKEGDYVVSKIFYQQPQQLVVKRGDKAEQEVSEDIFAAVTPTADPVTPKLATPEPRNAVRICSDSHIASDYVTPSDYVSAHEVSLAETSEVMCMEDEVQSIQPNHERPSSGPELEHGLENGAKEMLDDKEEQEKDRDNENQGEEDPTWFDSGSQFILNSQQLVEALSLCDDLLGSQDREENTNSGSLKDKQPCIADYAHLGPEDFKRDLEECQKIVLDPSNIELDTPPEFRLSQLEFGSQDSFLAWGTGKTD
AT4G10350.1	MGSSSNGGVPPGFRFHPTDEELLHYYLKKKISYQKFEMEVIREVDLNKLEPWDLQERCKIGSTPQNEWYFFSHKDRKYPTGSRTNRATHAGFWKATGRDKCIRNSYKKIGMRKTLVFYKGRAPHGQKTDWIMHEYRLEDADDPQANPSEDGWVVCRVFMKKNLFKVVNEGSSSINSLDQHNHDASNNNHALQARSFMHRDSPYQLVRNHGAMTFELNKPDLALHQYPPIFHKPPSLGFDYSSGLARDSESAASEGLQYQQACEPGLDVGTCETVASHNHQQGLGEWAMMDRLVTCHMGNEDSSRGITYEDGNNNSSSVVQPVPATNQLTLRSEMDFWGYSK
AT4G10350.2	MLSFYFLEERCKIGSTPQNEWYFFSHKDRKYPTGSRTNRATHAGFWKATGRDKCIRNSYKKIGMRKTLVFYKGRAPHGQKTDWIMHEYRLEDADDPQANPSEDGWVVCRVFMKKNLFKVVNEGSSSINSLDQHNHDASNNNHALQARSFMHRDSPYQLVRNHGAMTFELNKPDLALHQYPPIFHKPPSLGFDYSSGLARDSESAASEGLQYQQACEPGLDVGTCETVASHNHQQGLGEWAMMDRLVTCHMGNEDSSRGITYEDGNNNSSSVVQPVPATNQLTLRSEMDFWGYSK
AT3G61910.1	MNISVNGQSQVPPGFRFHPTEEELLKYYLRKKISNIKIDLDVIPDIDLNKLEPWDIQEMCKIGTTPQNDWYFYSHKDKKYPTGTRTNRATTVGFWKATGRDKTIYTNGDRIGMRKTLVFYKGRAPHGQKSDWIMHEYRLDESVLISSCGDHDVNVETCDVIGSDEGWVVCRVFKKNNLCKNMISSSPASSVKTPSFNEETIEQLLEVMGQSCKGEIVLDPFLKLPNLECHNNTTITSYQWLIDDQVNNCHVSKVMDPSFITSWAALDRLVASQLNGPNSYSIPAVNETSQSPYHGLNRSGCNTGLTPDYYIPEIDLWNEADFARTTCHLLNGSG

AT4G01540.4	MMKGLIGYRFSPTGEEVINHYLKNKLLGKYWLVDEAISEINILSHK<br>PSKDLPKLARIQSEDLEWYFFSPIEYTNPNKMKMKRTTGSGFWKP<br>TGVDREIRDKRGNGVVIGIKKTLVYHEGKSPHGVRTPWVMHEYH<br>ITCLPHHKRKYVVCQVKYKGEAAEISYEPSPSLVSDSHTVIAITGEP<br>EPELQVEQPGKENLLGMSVDDLIEPMNQQEEPQGPHLAPNDDEF<br>IRGLRHVDRGTVEYLFANEENMDGLSMNDLRIPMIVQQEDLSEW<br>EGFNADTFFSDNNNNYNLNVHHQLTPYGDGYLNAFSGYNEGN<br>PPDHELVMQENRNDHMPRKPVTGTIDYSSDSGSDAGSISTTVKHI<br>SHFLYHYFPGVYSVFSAFHSNLTELPRNIKSKY
AT4G01540.1	MMKGLIGYRFSPTGEEVINHYLKNKLLGKYWLVDEAISEINILSHK<br>PSKDLPKLARIQSEDLEWYFFSPIEYTNPNKMKMKRTTGSGFWKP<br>TGVDREIRDKRGNGVVIGIKKTLVYHEGKSPHGVRTPWVMHEYH<br>ITCLPHHKRKYVVCQVKYKGEAAEISYEPSPSLVSDSHTVIAITGEP<br>EPELQVEQPGKENLLGMSVDDLIEPMNQQEEPQGPHLAPNDDEF<br>IRGLRHVDRGTVEYLFANEENMDGLSMNDLRIPMIVQQEDLSEW<br>EGFNADTFFSDNNNNYNLNVHHQLTPYGDGYLNAFSGYNEGN<br>PPDHELVMQENRNDHMPRKPVTGTIDYSSDSGSDAGSISTTSYQG<br>TSSPNISVGSSSRHLSSCSSTDSCKDLQTCTDPSIISREIRELTQEVKQ<br>EIPRAVDAPMNNESSLVKTEKKGLFIVEDAMERNRKKPRFIYLMK<br>MIIGNIISVLLPVKRLIPVKKL
AT4G01540.3	MMKGLIGYRFSPTGEEVINHYLKNKLLGKYWLVDEAISEINILSHK<br>PSKDLPKLARIQSEDLEWYFFSPIEYTNPNKMKMKRTTGSGFWKP<br>TGVDREIRDKRGNGVVIGIKKTLVYHEGKSPHGVRTPWVMHEYH<br>ITCLPHHKRKYVVCQVKYKGEAAEISYEPSPSLVSDSHTVIAITGEP<br>EPELQVEQPGKENLLGMSVDDLIEPMNQQEEPQGPHLAPNDDEF<br>IRGLRHVDRGTVEYLFANEENMDGLSMNDLRIPMIVQQEDLSEW<br>EGFNADTFFSDNNNNYNLNVHHQLTPYGDGYLNAFSGYNEGN<br>PPDHELVMQENRNDHMPRKPVTGTIDYSSDSGSDAGSISTTSYQG<br>TSSPNISVGSSSRHLSSCSSTDSCKDLQTCTDPSIISREIRELTQEVLSC<br>SYVDFVAICLYLVI
AT4G01540.2	MMKGLIGYRFSPTGEEVINHYLKNKLLGKYWLVDEAISEINILSHK<br>PSKDLPKLARIQSEDLEWYFFSPIEYTNPNKMKMKRTTGSGFWKP<br>TGVDREIRDKRGNGVVIGIKKTLVYHEGKSPHGVRTPWVMHEYH<br>ITCLPHHKRKYVVCQVKYKGEAAEISYEPSPSLVSDSHTVIAITGEP<br>EPELQVEQPGKENLLGMSVDDLIEPMNQQEEPQGPHLAPNDDEF<br>IRGLRHVDRGTVEYLFANEENMDGLSMNDLRIPMIVQQEDLSEW<br>EGFNADTFFSDNNNNYNLNVHHQLTPYGDGYLNAFSGYNEGN<br>PPDHELVMQENRNDHMPRKPVTGTIDYSSDSGSDAGSISTTVKQE<br>IPRAVDAPMNNESSLVKTEKKGLFIVEDAMERNRKKPRFIYLMK<br>MIIGNIISVLLPVKRLIPVKKL

AT4G36160.3	MESVDQSCSVPPGFRFHPTDEELVGYYLRKKVASQKIDLDVIRDIDLYRIEPWDLQESCRIGYEERNEWYFFSHKDKKYPTGTRTNRATMAGFWKATGRDKAVYDKSKLIGMRKTLVFYKGRAPNGQKTDWIMHEYRLESDENAPPQEEGWVVCRAFKKKPMTGQAKNTETWSSSYFYDELPSGVRSVTEPLNYVSKQKQNVFAQDLMFKQELEGSDIGLNFIHCDQFIQLPQLESPSLPLTKRPVSLTSITSLEKNKNIYKRHLIEEDVSFNALISSGNKDKKKKKTSVMTTDWRALDKFVASQLMSQEDGVSGFGGHHEEDNNKIGHYNNEESNNKGSVETASSTLLSDREEENRFISGLLCSNLDYDLYRDLHV
AT4G36160.2	MKKIMESVDQSCSVPPGFRFHPTDEELVGYYLRKKVASQKIDLDVIRDIDLYRIEPWDLQESCRIGYEERNEWYFFSHKDKKYPTGTRTNRATMAGFWKATGRDKAVYDKSKLIGMRKTLVFYKGRAPNGQKTDWIMHEYRLESDENAPPQEEGWVVCRAFKKKPMTGQAKNTETWSSSYFYDELPSGVRSVTEPLNYVSKQKQNVFAQDLMFKQELEGSDIGLNFIHCDQFIQLPQLESPSLPLTKRPVSLTSITSLEKNKNIYKRHLIEEDVSFNALISSGNKDKKKKKTSVMTTDWRALDKFVASQLMSQEDGVSGFGGHHEEDNNKIGHYNNEESNNKGSVETASSTLLSDREEENRFISGLLCSNLDYDLYRDLHV
AT4G36160.1	MESVDQSCSVPPGFRFHPTDEELVGYYLRKKVASQKIDLDVIRDIDLYRIEPWDLQESCRIGYEERNEWYFFSHKDKKYPTGTRTNRATMAGFWKATGRDKAVYDKSKLIGMRKTLVFYKGRAPNGQKTDWIMHEYRLESDENAPPQEEGWVVCRAFKKKPMTGQAKNTETWSSSYFYDELPSGVRSVTEPLNYVSKQKQNVFAQDLMFKQELEGSDIGLNFIHCDQFIQLPQLESPSLPLTKRPVSLTSITSLEKNKNIYKRHLIEEDVSFNALISSGNKDKKKKKTSVMTTDWRALDKFVASQLMSQEDGVSGFGGHHEEDNNKIGHYNNEESNNKGSVETASSTLLSDREEENRFISGLLCSNLDYDLYRDLHV
AT1G71930.1	MDNIMQSSMPPGFRFHPTEEELVGYYLDRKINSMKSALDVIVEIDLYKMEPWDIQARCKLGYEEQNEWYFFSHKDRKYPTGTRTNRATAAGFWKATGRDKAVLSKNSVIGMRKTLVYYKGRAPNGRKSDWIMHEYRLQNSELAPVQEEGWVVCRAFRKPIPNQRPLGYEPWQNQLYHVESSNNYSSSVTMNTSHHIGASSSSHNLNQMLMSNNHYNPNNTSSSMHQYGNIELPQLDSPSLSPSLGTNKDQNESFEQEEEKSFNCVDWRTLDTLLETQVIHPHNPNILMFETQSYNPAPSFPSMHQSYNEVEANIHHSLGCFPDS
AT1G71930.2	MDNIMQSSMPPGFRFHPTEEELVGYYLDRKINSMKSALDVIVEIDLYKMEPWDIQARCKLGYEEQNEWYFFSHKDRKYPTGTRTNRATAAGFWKATGRDKAVLSKNSVIGMRKTLVYYKGRAPNGRKSDWIMHEYRLQNSELAPVQEEGWVVCRAFRKPIPNQRPLGYEPWQNQLYHVESSNNYSSSVTMNTSHHIGASSSSHNLNQMLMSNNHYNPNNTSSSMHQYGNIELPQLDSPSLSPSLGTNKDQNESFEQEEEKSFNCVDWRTLDTLLETQVIHPHNPNILMFETQSYNPAPSFPSMHQSYNEVEANIHHSLGCFPDS

AT3G44350.1	MGEELSVGFRFYPTEVELLTYYLRIQLGGGNATIHSLIPILDVFSVEP TQLPNLAGERCRGDAEQWIFFVPRQEREARGGRPSRTTGSGYWK ATGSPGPVFSPDNRVIGVKKTMVFYTGKAPTGRKTKWKMNEYK AVETASVSTIPKVHNHFFKMKTFIK
AT3G44350.2	MGEELSVGFRFYPTEVELLTYYLRIQLGGGNATIHSLIPILDVFSVEP TQLPNLAGERCRGDAEQWIFFVPRQEREARGGRPSRTTGSGYWK ATGSPGPVFSPDNRVIGVKKTMVFYTGKAPTGRKTKWKMNEYK AVETASVSTIPKLRPEFSICRIYIKSGSSRAFDRRPTEAYAIERNLPSN GVETSSRATISTSPETSHSGGNQVDLPVNATTITQSISDMVDELSQP FWEWEQMNWS
AT1G79580.1	MEIGSSSTVAGGGQLSVPPGFRFHPTEEELLYYYLKKKVSYEPIDLD VIREVDLNKLEPWELKEKCRIGSGPQNEWYFFSHKDKKYPTGTRT NRATAAGFWKATGRDKSIHLNSSKKIGLRKTLVFYTGRAPHGQK TEWIMHEYRLDDSENEIQEDGWVVCRVFKKKNHFRGFHQEQEQ DHHHHHQYISTNNDHDHHHHIDSNSNNHSPLILHPLDHHHHH HHIGRQIHMPLHEFANTLSHGSMHLPQLFSPDSAAAAAAAAS AQPFVSPINTTDIECSQNLLRLTSNNNYGGDWSFLDKLLTTGNM NQQQQQQVQNHQAKCFGDLSNNDNNDQADHLGNNNGGSSSS PVNQRFPFHYLGNDANLLKFPK
AT1G79580.2	MEIGSSSTVAGGGQLSVPPGFRFHPTEEELLYYYLKKKVSYEPIDLD VIREVDLNKLEPWELKEKCRIGSGPQNEWYFFSHKDKKYPTGTRT NRATAAGFWKATGRDKSIHLNSSKKIGLRKTLVFYTGRAPHGQK TEWIMHEYRLDDSENEIQEDGWVVCRVFKKKNHFRGFHQEQEQ DHHHHHQYISTNNDHDHHHHIDSNSNNHSPLILHPLDHHHHH HHIGRQIHMPLHEFANTLSHGSMHLPQLFSPDSAAAAAAAAS AQPFVSPINTTDIECSQNLLRLTSNNNYGGDWSFLDKLLTTGNM NQQQQQQVQNHQAKCFGDLSNNDNNDQADHLGNNNGGSSSS PVNQRFPFHYLGNDANLLKFPK
AT1G79580.4	MEIGSSSTVAGGGQLSVPPGFRFHPTEEELLYYYLKKKVSYEPIDLD VIREVDLNKLEPWELKEKCRIGSGPQNEWYFFSHKDKKYPTGTRT NRATAAGFWKATGRDKSIHLNSSKKIGLRKTLVFYTGRAPHGQK TEWIMHEYRLDDSENEIQEDGWVVCRVFKKKNHFRGFHQEQEQ DHHHHHQYISTNNDHDHHHHIDSNSNNHSPLILHPLDHHHHH HHIGRQIHMPLHEFANTLSHGSMHLPQLFSPDSAAAAAAAAS AQPFVSPINTTDIECSQNLLRLTSNNNYGGDWSFLDKLLTTGNM NQQQQQQVQNHQAKCFGDLSNNDNNDQADHLGNNNGGSSSS PVNQRFPFHYLGNDANLLKFPK

AT1G79580.5	MEIGSSSTVAGGGQLSVPPGFRFHPTEEELLYYYLKKKVSYEPIDLDVIREVDLNKLEPWELKEKCRIGSGPQNEWYFFSHKDKKYPTGTRTNRATAAGFWKATGRDKSIHLNSSKKIGLRKTLVFYTGRAPHGQKTEWIMHEYRLDDSENEIQEDGWVVCRVFKKKNHFRGFHQEQEQDHHHHHQYISTNNDHDHHHHIDSNSNNHSPLILHPLDHHHHHHHIGRQIHMPLHEFANTLSHGSMHLPQLFSPDSAAAAAAAAASAQPFVSPINTTDIECSQNLLRLTSNNNYGGDWSFLDKLLTTGNMNQQQQQQVQNHQAKCFGDLSNNDNNDQADHLGNNNGGSSSSPVNQRFPFHYLGNDANLLKFPK
AT1G79580.3	MEIGSSSTVAGGGQLSVPPGFRFHPTEEELLYYYLKKKVSYEPIDLDVIREVDLNKLEPWELKEKCRIGSGPQNEWYFFSHKDKKYPTGTRTNRATAAGFWKATGRDKSIHLNSSKKIGLRKTLVFYTGRAPHGQKTEWIMHEYRLDDSENEIQEDGWVVCRVFKKKNHFRGFHQEQEQDHHHHHQYISTNNDHDHHHHIDSNSNNHSPLILHPLDHHHHHHHIGRQIHMPLHEFANTLSHGSMHLPQLFSPDSAAAAAAAAASAQPFVSPINTTDIECSQNLLRLTSNNNYGGDWSFLDKLLTTGNMNQQQQQQVQNHQAKCFGDLSNNDNNDQADHLGNNNGGSSSSPVNQRFPFHYLGNDANLLKFPK
AT4G28530.1	MGLKDIGSKLPPGFRFHPSDEELVCHYLCNKIRAKSDHGDVDDDDDDVDEALKGSTDLVEIDLHICEPWELPDVAKLNAKEWYFFSFRDRKYATGYRTNRATVSGYWKATGKDRTVMDPRTRQLVGMRKTLVFYRNRAPNGIKTTWIMHEFRLECPNIPPKEDWVLCRVFNKGRDSSLQDNNYYNNDNQTQRLEVNDAPDLNYNNQLPPLLSSPPHNHQHEKMKIQVCDQWEQLMKQPSRTTGHPYHHHCHHQTIACGWEQMMIGSLSSPSSHGPDHESLLNLLYVDNNNSVNISGDHHQNYEKILLSSLDMTSLDHDKTCMGSSSDGGMVSDLHMECGGLSFETENILAFQ
AT4G28530.2	MGLKDIGSKLPPGFRFHPSDEELVCHYLCNKIRAKSDHGDVDDDDDDVDEALKGSTDLVEIDLHICEPWELPDVAKLNAKEWYFFSFRDRKYATGYRTNRATVSGYWKATGKDRTVMDPRTRQLEDWVLCRVFNKGRDSSLQDNNYYNNDNQTQRLEVNDAPDLNYNNQLPPLLSSPPHNHQHEKMKIQVCDQWEQLMKQPSRTTGHPYHHHCHHQTIACGWEQMMIGSLSSPSSHGPDHESLLNLLYVDNNNSVNISGDHHQNYEKILLSSLDMTSLDHDKTCMGSSSDGGMVSDLHMECGGLSFETENILAFQ

AT1G65910.1	MAPVSMPPGFRFHPTDEELVIYYLKRKINGRTIELEIIPEIDLYKCEP<br>WDLPGKSLLPSKDLEWFFFSPRDRKYPNGSRTNRATKAGYWKAT<br>GKDRKVTSHSRMVGTKKTLVYYRGRAPHGSRTDWVMHEYRLEE<br>QECDSKSGIQDAYALCRVFKKSALANKIEEQHHGTKKNKGTTNS<br>EQSTSSTCLYSDGMYENLENSGYPVSPETGGLTQLGNNSSSDMETI<br>ENKWSQFMSHDTSFNFPPQSQYGTISYPPSKVDIALECARLQNRM<br>LPPVPPLYVEGLTHNEYFGNNVANDTDEMLSKIIALAQASHEPR<br>NSLDSWDGGSASGNFHGDFNYSGEKVSCLEANVEAVDMQEHH<br>VNFKEERLVENLRWVGVSSKELEKSFVEEHSTVIPIEDIWRYHND<br>NQEQEHHDQDGMDVNNNNGDVDDAFTLEFSENEHNENLLDK<br>NDHETTSSSCFEVVKKVEVSHGLFVTTRQVTNTFFQQIVPSQTVIV<br>YINPTDGNECCHSMTSKEEVHVRKKINPRINGVSSTVLGQWRKF<br>AHVIGFIPMLLLMRCVHRGNSNKNRGSEGYSRQPTRGDCNNRGT<br>ILMMENAVVRRKIWKKKKEKNMVDEQGFRFQDSFVLKKLGLSL<br>AIILAVSTISLI
AT3G55210.1	MSPPSTIAYVLPPGFKFVPNDEEVIHCYLKPYSDGNTNVLLHVPIH<br>LVNIYESNPQTLSEEFQKGNDKEWFIITERNKVDQGLSQTKRVGY<br>GAKRQKRVDTNGGYWHATVAAQKINAGDGVVRNKRPLAYYV<br>GKPSEGVKTDWLMQEYSLDHSSHNNDKDYTLCKIYLTPQATKM<br>NKEVGEEKKKQKKGEAVVSVAPVEALEEQLPCNVEYHQPLAPL<br>DSCQPQPHDLAYQQQQFCPGPLDSYQPQPHDMENQQPHNEKL<br>KKEEDVEQLDLHQPDQGKGC
AT3G29035.1	MDYKVSRSGEIVEGEVEDSEKIDLPPGFRFHPTDEELITHYLRPKVV<br>NSFFSAIAIGEVDLNKVEPWDLPWKAKLGEKEWYFFCVRDRKYP<br>TGLRTNRATKAGYWKATGKDKEIFKGKSLVGMKKTLVFYKGRA<br>PKGVKTNWVMHEYRLEGKFAIDNLSKTAKNECVISRVFHTRTDG<br>TKEHMSVGLPPLMDSSPYLKSRGQDSLAGTTLGGLLSHVTYFSDQ<br>TTDDKSLVADFKTTMFGSGSTNFLPNIGSLLDFDPLFLQNNSSVLK<br>MLLDNEETQFKKNLHNSGSSESELTASSWQGHNSYGSTGPVNLD<br>CVWKF
AT1G28470.1	MSWCDGSDDNYDLNLERVSNTDHPSVQLKDQSQSCVTSRPDSKI<br>SAETPITTCPSCGHKLHHHQDDQVGSIKDLPSLPAGVKFDPSDKEI<br>LMHLEAKVSSDKRKLHPLIDEFIPTLEGENGICYTHPEKLPGVSKD<br>GQVRHFFHRPSKAYTTGTRKRRKVSTDEEGHETRWHKTGKTRPV<br>LSQSGETGFKKILVLYTNYGRQKKPEKTNWVMHQYHLGSSEDEK<br>DGEPVLSKVFYQTQPRQCGSMEPKPKNLVNLNRFSYENIQAGFG<br>YEHGGKSEETTQVIRELVVREGDGSCSFLSFTCDASKGKESFMKN<br>Q
AT2G33480.3	MMITGDCESEMYFFSTREAKYPNGNRSNRSTGSGYWKATGLDKQ<br>IGKKKLVVGMKKTLVFYKGKPPNGTRTNWVLHEYRLVDSQQDS<br>LYGQNMNWVLCRVFLKKRSNSNSKRKEDEKEEVENEKETETERE<br>REEENKKSTCPIFYDFMRKDTKKKRRRRRCCDLNLTPATCCCCSS<br>STSSSSVCSSALTHTSSNDNRQEISYRENKFCLFL

AT2G33480.1	MEKRSSIKNRGVLRLPPGFRFHPTDEELVVQYLRRKVTGLPLPASV<br>IPETDVCKSDPWDLPGDCESEMYFFSTREAKYPNGNRSNRSTGSG<br>YWKATGLDKQIGKKKLVVGMKKTLVFYKGKPPNGTRTNWVLH<br>EYRLVDSQQDSLYGQNMNWVLCRVFLKKRSNSNSKRKEDEKEE<br>VENEKETETEREREEENKKSTCPIFYDFMRKDTKKKRRRRRCCDL<br>NLTPATCCCCSSSTSSSSVCSSALTHTSSNDNRQEISYRENKFCLFL
AT2G33480.2	MEKRSSIKNRGVLRLPPGFRFHPTDEELVVQYLRRKVTGLPLPASV<br>IPETDVCKSDPWDLPGDCESEMYFFSTREAKYPNGNRSNRSTGSG<br>YWKATGLDKQIGKKKLVVGMKKTLVFYKGKPPNGTRTNWVLH<br>EYRLVDSQQDSLYNMNWVLCRVFLKKRSNSNSKRKEDEKEEVEN<br>EKETETEREREEENKKSTCPIFYDFMRKDTKKKRRRRRCCDLNLTP<br>ATCCCCSSSTSSSSVCSSALTHTSSNDNRQEISYRENKFCLFL
AT5G39820.1	MVLVMDDEESNNVERYDDVVLPGFRFHPTDEELVSFYLKRKVLH<br>KSLPFDLIKKVDIYKYDPWDLPKLAAMGEKEWYFYCPRDRKYRN<br>STRPNRVTGGGFWKATGTDRPIYSLDSTRCIGLKKSLVFYRGRAA<br>KGVKTDWMMHEFRLPSLSDSHHSSYPNYNNKKQHLNNNNNSK<br>ELPSNDAWAICRIFKKTNAVSSQRSIPQSWVYPTIPDNNQQSHNN<br>TATLLASSDVLSHISTRQNFIPSPVNEPASFTESAASYFASQMLGVT<br>YNTARNNGTGDALFLRNNGTGDALVLSNNENNYFNNLTGGLT<br>HEVPNVRSMVMEETTGSEMSATSYSTNN
AT1G64100.2	MTLQSQSSHCLNVHSSMVRDIEKCEQKTKRDIEKKNTKSGGVRL<br>NSRRLIHGRVAEKGTKSLPSLTQVTFEGEELKLKSGSHYFKSLDDA<br>IDFFDYMVRSRPFYTAVDCNKVIGVFVRMNRPDVAISLYRKMEIR<br>RIPLNIYSFNILIKCFCDCHKLSFSLSTFGKLTKLGFQPDVVTFNTLL<br>HGLCLEDRISEALALFGYMVETGFLEAVALFDQMVEIGLTPVVITF<br>NTLINGLCLEGRVLEAAALVNKMVGKGLHIDVVTYGTIVNGMC<br>KMGDTKSALNLLSKMEETHIKPDVVIYSAIIDRLCKDGHHSDAQY<br>LFSEMLEKGIAPNVFTYNCMIDGFCSFGRWSDAQRLLRDMIEREI<br>NPDVLTFNALISASVKEGKLFEAEKLCDEMLHRCIFPDTVTYNSMI<br>YGFCKHNRFDDAKHMFDLMASPDVVTFNTIIDVYCRAKRVDEG<br>MQLLREISRRGLVANTTTYNTLIHGFCEVDNLNAAQDLFQEMIS<br>HGVCPDTITCNILLYGFCENEKLEEALELFEVIQMSKIDLDTVAYN<br>IIIHGMCKGSKVDEAWDLFCSLPIHGVEPDVQTYNVMISGFCGKS<br>AISDANVLFHKMKDNGHEPDNSTYNTLIRGCLKAGEIDKSIELISE<br>MRSNGFSGDAFTIKMAEEIICRVSDEEIIENYLRPKINGETSSIPRYV<br>VELAEELYTVEPWLLPRQTAPILNPGEWFYFGKRNRKYSNLEGVH<br>CEGSWILEDGCIAVLSKETGEEIGGTTRFRYYYRNKGDKESRLKMS<br>NWFMREYRLYYKSRRVFNGRQVFCIITCNDEHFIE
AT1G56010.1	MACVGGKDWYFYSQRDRKYATGLRTNRATATGYWKATGKDRT<br>ILRKGKLVGMRKTLVFYQGRAPRGRKTDWVMHEFRLQGSHHPP<br>NHSLSSPKEDWVLCRVFHKNTEGVICRDNMGSCFDETASASLPPL<br>MDPYINFDQEPSSYLSDDHHYIINEHVPCFSNLSQNQTLNSNLTN<br>SVSELKIPCKNPNPLFTGGSASATLTGLDSFCSSDQMVLRALLSQL<br>TKIDGSLGPKESQSYGEGSSESLLTDIGIPSTVWNC

AT1G56010.2	METEEEMKESSISMVEAKLPPGFRFHPKDDELVCDYLMRRSLHN NHRPPLVLIQVDLNKCEPWDIPKMACVGGKDWYFYSQRDRKYA TGLRTNRATATGYWKATGKDRTILRKGKLVGMRKTLVFYQGRA PRGRKTDWVMHEFRLQGSHHPPNHSLSSPKEDWVLCRVFHKNT EGVICRDNMGSCFDETASASLPPLMDPYINFDQEPSSYLSDDHHYI INEHVPCFSNLSQNQTLNSNLTNSVSELKIPCKNPNPLFTGGSASA TLTGLDSFCSSDQMVLRALLSQLTKIDGSLGPKESQSYGEGSSESLL TDIGIPSTVWNC
AT3G10480.3	MGRESLAVVSSPPSATAPSTAVSATSLAPGFRFHPTDEELVSYYLK RKVLGKPVRFDAIGEVDIYKHEPWDLAEKILCSYLALNCFKNFVF ACATVMFACATVMFALFSKLKTRDQEWYFFSALDKKYGNGARM NRATNKGYWKATGKDREIRRDIQLLGMKKTLVFHSGRAPDGLRT NWVMHEYRLVEYETETNGSLLQDAYVLCRVFHKNNIGPPSGNR YAPFMEEEWADGGGALIPGIDVRVRVEALPQANGNNQMDQWA DLLKLHNSIKFAITFCRTQLNLTALSNERCSTREIFIVFWLICKEMH SASKDLININELPRDATPMDIEPNQQNHHESAFKPQESNNHSGYE EDEDTLKREHAEEDERPPSLCILNKEAPLPLLQYKRRRQNESNNN SSRNTQDHCSSTITTVDNTTTLISSSAAAATNTAISALLEFSLMGIS DKKENQQKEETSPPSPIASPEEKVNDLQKEVHQMSVERETFKLEM MSAEAMISILQSRIDALRQENEELKKKNASGQAS
AT3G10480.2	MGRESLAVVSSPPSATAPSTAVSATSLAPGFRFHPTDEELVSYYLK RKVLGKPVRFDAIGEVDIYKHEPWDLAVFSKLKTRDQEWYFFSAL DKKYGNGARMNRATNKGYWKATGKDREIRRDIQLLGMKKTLV FHSGRAPDGLRTNWVMHEYRLVEYETETNGSLLDAYVLCRVFH KNNIGPPSGNRYAPFMEEEWADGGGALIPGIDVRVRVEALPQAN GNNQMDQEMHSASKDLININELPRDATPMDIEPNQQNHHESAF KPQESNNHSGYEEDEDTLKREHAEEDERPPSLCILNKEAPLPLLQ YKRRRQNESNNNSSRNTQDHCSSTITTVDNTTTLISSSAAAATNT AISALLEFSLMGISDKKENQQKEETSPPSPIASPEEKVNDLQKEVH QMSVERETFKLEMMSAEAMISILQSRIDALRQENEELKKKNASGQ AS
AT3G10480.1	MGRESLAVVSSPPSATAPSTAVSATSLAPGFRFHPTDEELVSYYLK RKVLGKPVRFDAIGEVDIYKHEPWDLAVFSKLKTRDQEWYFFSAL DKKYGNGARMNRATNKGYWKATGKDREIRRDIQLLGMKKTLV FHSGRAPDGLRTNWVMHEYRLVEYETETNGSLLQDAYVLCRVF HKNNIGPPSGNRYAPFMEEEWADGGGALIPGIDVRVRVEALPQA NGNNQMDQEMHSASKDLININELPRDATPMDIEPNQQNHHESA FKPQESNNHSGYEEDEDTLKREHAEEDERPPSLCILNKEAPLPLLQ YKRRRQNESNNNSSRNTQDHCSSTITTVDNTTTLISSSAAAATNT AISALLEFSLMGISDKKENQQKEETSPPSPIASPEEKVNDLQKEVH QMSVERETFKLEMMSAEAMISILQSRIDALRQENEELKKKNASGQ AS

AT4G29230.1	MNKSNPAGSVTGSDIIDAKIEEHQLCGSKKCPSCGHKLEGKPQD<br>WVGLPAGVKFDPTDQELIEHLEAKVLAKDFKSHPLIDEFIPTIEGE<br>DGICYTHPEKLPGVTRDGLSRHFFHRPSKAYTTGTRKRRKIQTECD<br>NNLQGSSSSGETRWHKTGKTRPVMVNGKQKGCKKILVLYTNFG<br>KNRKPEKTNWVMHQYHLGTHEEEKEGELVVSKIFYQTQPRQCN<br>WSSSTSSLNAIGGGGGEASSGGGGGEYHMRRDSGTTSGGSCSSSR<br>EIINVNPPNRSDEIGGVGGGVMAVAAAAAAVAAGLPSYAMDQL<br>SFVPFMKSFDEVARRETPQTGHATCEDVMAEQHRHRHQPSSSTS<br>HHMAHDHHHHHHQQQQQRHHAFNISQPTHPISTIISPSTSLHH<br>ASINILDDNPYHVHRILLPNENYQTQQQLRQEGEEEHNDGKMG<br>GRSASGLEELIMGCTSSTTHHDVKDGSSSMGNQQEAEWLKYSTF<br>WPAPDSSDNQDHHG
AT4G29230.2	MNKSNPAGSVTGSDIIDAKIEEHQLCGSKKCPSCGHKLEGKPDW<br>VGLPAGVKFDPTDQELIEHLEAKVLAKDFKSHPLIDEFIPTIEGED<br>GICYTHPEKLPGVTRDGLSRHFFHRPSKAYTTGTRKRRKIQTECD<br>NNLQGSSSSGETRWHKTGKTRPVMVNGKQKGCKKILVLYTNFG<br>KNRKPEKTNWVMHQYHLGTHEEEKEGELVVSKIFYQTQPRQCN<br>WSSSTSSLNAIGGGGGEASSGGGGGEYHMRRDSGTTSGGSCSSSR<br>EIINVNPPNRSDEIGGVGGGVMAVAAAAAAVAAGLPSYAMDQL<br>SFVPFMKSFDEVARRETPQTGHATCEDVMAEQHRHRHQPSSSTS<br>HHMAHDHHHHHHQQQQQRHHAFNISQPTHPISTIISPSTSLHH<br>ASINILDDNPYHVHRILLPNENYQTQQQLRQEGEEEHNDGKMG<br>GRSASGLEELIMGCTSSTTHHDVKDGSSSMGNQQEAEWLKYSTF<br>WPAPDSSDNQDHHG
AT4G29230.3	MNKSNPAGSVTGSDIIDAKIEEHQLCGSKKCPSCGHKLEGKPQD<br>WVGLPAGVKFDPTDQELIEHLEAKVLAKDFKSHPLIDEFIPTIEGE<br>DGICYTHPEKLPGVTRDGLSRHFFHRPSKAYTTGTRKRRKIQTECD<br>NNLQGSSSSGETRWHKTGKTRPVMVNGKQKGCKKILVLYTNFG<br>KNRKPEKTNWVMHQYHLGTHEEEKEGELVVSKIFYQTQPRQCN<br>WSSSTSSLNAIGGGGGEASSGGGGGEYHMRRDSGTTSGGSCSSSR<br>EIINVNPPNRSDEIGGVGGGVMAVAAAAAAVAAGLPSYAMDQL<br>SFVPFMKSFDEVARRETPQTGHATCEDVMAEQHRHRHQPSSSTS<br>HHMAHDHHHHHHQQQQQRHHAFNISQPTHPISTIISPSTSLHH<br>ASINILDDNPYHVHRILLPNENYQVKYLIFSFSYILELQKK
AT2G17040.1	MGKDIELPGFRFHPTEEELLDFYLKNMVYGKRSSVEVIGFLNIYRH<br>DPWDLPGLSRIGEREWYFFVPRERKHGNGGRPSRTTEKGYWKAT<br>GSDRKIISLSEPKRVIGLKKTLVFYRGRAPGGSKTDWVMNEFRMP<br>DNCSLPKDVVLCKIYRKATSLKVLEQRAEMEAKMNQTCPNSPLS<br>SSETISFVGKEENMMTSFRAPQVIAMEEANKIQMHQENAKTEEK<br>QREAETKEPSSSLKLPFGSLPELQLPKPGVEWDQLLSISPWLQNLT<br>PIVNIYW

AT2G46770.1	MMSKSMSISVNGQSQVPPGFRFHPTEEELLQYYLRKKVNSIEIDLD VIRDVDLNKLEPWDIQEMCKIGTTPQNDWYFFSHKDKKYPTGTR TNRATAAGFWKATGRDKIIYSNGRRIGMRKTLVFYKGRAPHGQK SDWIMHEYRLDDNIISPEDVTVHEVVSIIGEASQDEGWVVCRIFKK KNLHKTLNSPVGGASLSGGGDTPKTTSSQIFNEDTLDQFLELMGR SCKEELNLDPFMKLPNLESPNSQAINNCHVSSPDTNHNIHVSNV VDTSFVTSWAALDRLVASQLNGPTSYSITAVNESHVGHDHLALPS VRSPYPSLNRSASYHAGLTQEYTPEMELWNTTTSSLSSSPGPFCHV SNGSG
AT5G63790.1	MDFALFSSISIFEINHKDPPIRRFIKTQNRILSTRKQQGTFPKMKAEL NLPAGFRFHPTDEELVKFYLCRRCASEPINVPVIAEIDLYKFNPWE LPEMALYGEKEWYFFSHRDRKYPNGSRPNRAAGTGYWKATGAD KPIGKPKTLGIKKALVFYAGKAPKGIKTNWIMHEYRLANVDRSAS TNKKNNLRLDDWVLCRIYNKKGTMEKYLPAAAEKPTEKMSTSD SRCSSHVISPDVTCSDNWEVESEPKWINLEDALEAFNDDTSMFSSI GLLQNDAFVPQFQYQSSDFVDSFQDPFEQKPFLNWNFAPQG
AT5G63790.2	MNLPFVYKSSMDFALFSSISIFEINHKDPPIRRFIKTQNRILSTRKQQ GTFPKMKAELNLPAGFRFHPTDEELVKFYLCRRCASEPINVPVIAE IDLYKFNPWELPEMALYGEKEWYFFSHRDRKYPNGSRPNRAAGT GYWKATGADKPIGKPKTLGIKKALVFYAGKAPKGIKTNWIMHEY RLANVDRSASTNKKNNLRLDDWVLCRIYNKKGTMEKYLPAAAE KPTEKMSTSDSRCSSHVISPDVTCSDNWEVESEPKWINLEDALEAF NDDTSMFSSIGLLQNDAFVPQFQYQSSDFVDSFQDPFEQKPFLNW NFAPQG
AT1G76420.1	MMLAVEDVLSELAGEERNERGLPPGFRFHPTDEELITFYLASKIFH GGLSGIHISEVDLNRCEPWELPEMAKMGEREWYFYSLRDRKYPT GLRTNRATTAGYWKATGKDKEVFSGGGGQLVGMKKTLVFYKGR APRGLKTKWVMHEYRLENDHSHRHTCKEEWVICRVFNKTGDRK NVGLIHNQISYLHNHSLSTTHHHHHEALPLLIEPSNKTLTNFPSLL YDDPHQNYNNNNFLHGSSGHNIDELKALINPVVSQLNGIIFPSG NNNNDEDDFDFNLGVKTEQSSNGNEIDVRDYLENPLFQEASYGL LGFSSSPGPLHMLLDSPCPLGFQL
AT1G02230.1	MMNPVGFRFRPNDEEIVDHYLRPKNLDSDTSHVDEVISTVDICSF EPWDLPSKSMIKSRDGVWYFFSVKEMKYNRGDQQRRRTNSGFW KKTGKTMTVMRKRGNREKIGEKRVLVFKNRDGSKTDWVMHEY HATSLFPNQMMTYTVCKVEFKGEETEISSSSTGSEIEQIHSLIPLVNS SGGSEGSSFHSQELQNSSQSGVFANVQGESQIDDATTPIEEEWKT WLNNDGDEQRNIMFMQDHRSDYTPLKSLTGVFSDDSSDDNDSD LISPKTNSIGTSSTCASFASSNHQIDQTQHSPDSTVQLVSLTQEVSQ GPGQVTVIREHKLGEESVKKKRASFVYRMIHRLVKKIHQCYSISRT

AT2G43000.1	MSGEGNLGKDHEEENEAPLPGFRFHPTDEELLGYYLRRKVENKTI KLELIKQIDIYKYDPWDLPRVSSVGEKEWYFFCMRGRKYRNSVRP NRVTGSGFWKATGIDKPVYSNLDCVGLKKSLVYYLGSAGKGTKT DWMMHEFRLPSTTKTDSPAQQAEVWTLCRIFKRVTSQRNPTILPP NRKPVITLTDTCSKTSSLDSDHTSHRTVDSMSHEPPLPQPQNPYW NQHIVGFNQPTYTGNDNNLLMSFWNGNGGDFIGDSASWDELRS VIDGNTKP
AT2G43000.2	MRGRKYRNSVRPNRVTGSGFWKATGIDKPVYSNLDCVGLKKSLV YYLGSAGKGTKTDWMMHEFRLPSTTKTDSPAQQAEVWTLCRIFK RVTSQRNPTILPPNRKPVITLTDTCSKTSSLDSDHTSHRTVDSMSH EPPLPQPQNPYWNQHIVGFNQPTYTGNDNNLLMSFWNGNGGD FIGDSASWDELRSVIDGNTKP
LOC_Os01g01430.1	MMTIDLQLPAAACGDHHTAAGAGLPPGFRFHPTDEELLLHYLG KRAAAAPCPAPVIAEVDIYKYNPWELPAMAVFGESDGEWYFFSP RDRKYPNGVRPNRAAGSGYWKATGTDKPISISETQQTVLLGVKK ALVFYRGRPPKGTKTSWIMHEYRLANAAASSSSSYTSNMKQLASS SSSSSSSASMRLDEWVLCRIYKKKEANQQLQHYIDMMMDDDND DEHNLQVQQQQQQQAQSHRMPRPPSISDYLLDYSDDLPPSTDQT PSLHLGFTAVNEGNNKRHKTMEEYYSISISTADMLHASSSTSNNK STQINFSSIFEPQTPAAAGHQLMSSHNDDTSI
LOC_Os01g01470.1	MGEQQQQVERQPDLPPGFRFHPTDEEIITFYLAPKVVDSRGFCVA AIGEVDLNKCEPWDLPGKAKMNGEKEWYFYCQKDRKYPTGMR TNRATEAGYWKATGKDKEIFRDHHMLIGMKKTLVFYKGRAPKG DKTNWVMHEYRLADASPPPPPSSAEPPRQDDWAVCRIFHKSSGI KKPVPVAPHQVPAAANYQQQQMAMASAGIIQVPMQMQMPS MSDQLQMLDDFSTTASLSLMAPPSYSTLPAGFPLQINSGAHPQQF VGNPSMYYHQQQQMDMAGGGFVVSEPSSLVVSPQDAADQNNN AADISSMACNMDAAIWKY
LOC_Os01g09550.1	MYTSPTLLALSFALLLLILLLYYSSSLLYSQLATRRRLTHACARSLP DAYESGMNRGHISSSELIDAKLEERRISTAKHCPSCGNKLDCKPD WVGLPAGVKFDPTDQELIEHLEAKVREEGSRSHPLIDEFIPTIEGE DGICYTHPEKLPGVTRDGLSKHFFHRPSKAYTTGTRKRRKIQTEC DVQKGETRWHKTGKTRPVMVSGRQKGCKKILVLYTNFGKHRKP EKTNWVMHQYHLGDLEEEKEGELVVCKIFYQTQPRQCSWSSDR GGGAAATASAVTTAAVQQDQQRRRDSGSGSCSSTRDHEVSATSY STAGYAVAAAVEMQHLKHAADHFSFAPFRKSFEEVGISGDQVHS NQLGRSEQQHAGQEQQPHRPLLATTTAVPATAFLISRPTNPVSNI VPPAMQHASVVLDHDQFHVPAILLHHDKFQQQQQKLDRRSAG LEELIMGCTSSSTKGEASIPHSQETEWPYQPYWTPDNQDHHG

LOC_Os01g09550.2	MNRGHISSSELIDAKLEERRISTAKHCPSCGNKLDCKPDWVGLPAGVKFDPTDQELIEHLEAKVREEGSRSHPLIDEFIPTIEGEDGICYTHPEKLPGVTRDGLSKHFFHRPSKAYTTGTRKRRKIQTECDVQKGETRWHKTGKTRPVMVSGRQKGCKKILVLYTNFGKHRKPEKTNWVMHQYHLGDLEEEKEGELVVCKIFYQTQPRQCSWSSDRGGGAAATASAVTTAAVQQDQQRRRDSGSGSCSSTRDHEVSATSYSTAGYAVAAAVEMQHLKHAADHFSFAPFRKSFEEVGISGDQVHSNQLGRSEQQHAGQEQQPHRPLLATTTAVPATAFLISRPTNPVSNIVPPAMQHASVVLDHDQFHVPAILLHHDKFQQQQQKLDRRSAGLEELIMGCTSSSTKGEASIPHSQETEWPYQPYWTPDNQDHHG
LOC_Os01g09550.3	MVSGRQKGCKKILVLYTNFGKHRKPEKTNWVMHQYHLGDLEEEKEGELVVCKIFYQTQPRQCSWSSDRGGGAAATASAVTTAAVQQDQQRRRDSGSGSCSSTRDHEVSATSYSTAGYAVAAAVEMQHLKHAADHFSFAPFRKSFEEVGISGDQVHSNQLGRSEQQHAGQEQQPHRPLLATTTAVPATAFLISRPTNPVSNIVPPAMQHASVVLDHDQFHVPAILLHHDKFQQQQQKLDRRSAGLEELIMGCTSSSTKGEASIPHSQETEWPYQPYWTPDNQDHHG
LOC_Os01g15640.1	MESLRDMVLPPGFGFHPKDTELISHYLKKKIHGQKIEYEIIPEVDIYKHEPWDLPAKCDVPTQDNKWHFFAARDRKYPNGSRSNRATVAGYWKSTGKDRAIKMGKQTIGTKKTLVFHEGRPPTGRRTEWIMHEYYIDERECQACPDMKDAYVLCRITKRNDWIPGNGNELDNSDPHPEPYDAPPSVISTEQLNPAAEPVVGVEAAPVTVAEPDGVTTSAITANIPSPSDDINLDDWLNELFDPFFDPEQSLASADLSPDEQNVESSNVGALAPKVEQDYSSPNENVVDDTEYLLPEDVYNILHPGTDDFNMLQNPLDQYPIQYATDVWSGIQKEELWSPQANAEPSQSNEAADNGIIRRYRSMKTPETSVPQFKGKTQAKMRVGINKMATSSSESINQTIKFENSGRLVEHQKNQAHDVASTKRSDAGKPSTELSSNRGFLRGIRNAFAGCSDARWNMILVAGFAIGVAVVALHIGQRLGLSQRDQQHT
LOC_Os01g29840.1	MGEQQQQVERQPDLPPGFRFHPTDEEIITFYLAPKVVDSRGFCVAAIGEVDLNKCEPWDLPGKAKMNGEKEWYFYCQKDRKYPTGMRTNRATEAGYWKATGKDKEIFRNHHMLIGMKKTLVFYKGRAPKGDKTNWVMHEYRLADASPPQPPPPPSSAEPPRQDDWAVCRIFHKSSGIKKPVQVPMQMPMQMQMPVAHQVPAANYQQQMAMASASIIQVPMQMQMPSMSDQLQMLDDFSTGSLMAPPPPPPSYSTLPGFPLQINGGAQQFVGNPSMYYQQQQQQQQQMDMAAGGFVVSEPSSLVVSPQDAADQNNAADISSVACNMDATIWKY

LOC_Os01g47670.1	MGAASRPRRQCRAPRRLDGGGGGSMDVHPSELLPRSRCTAPRRLDDDEMDVHPSEQELIETGFAPAVARSSGDEAWYFFSAVRGLKGGRKARTVDDGAGCWHSEAGAKPVLAASSGRRLGHRQSFSFITKDDDGQRVRSGWLMVELSLDVDEEEQLVLSKVYFSPRAPGARKPTTAAAMSRHKRKLSTTDIASPPRRQRRHRVVPSSPPEEPNTSPSPAAAPPDQQEGGDDDPDRGSISWWLRRVFGLTATFTEEESIELNPWLKDILRPFPPPLPPTPPPPCPSPRRKLIDMPEIREFIMRGSYLGGGPAPPRYECDHPAMVMTGGDDQQQLDEQRRDDVGDDRAHYDRVDGQLQFERHYLQL
LOC_Os01g48130.1	MTWCNSFNDVRAVENNLATAAAVAAAKKQQQQQQVSQHVNLIKTCPSCGHRAQYEQAAAAATIQDLPGLPAGVKFDPTDQELLEHLEGKARPDARKLHPLIDEFIPTIEGENGICYTHPERLPGVGKDGLIRHFFHRPSKAYTTGTRKRRKVHTDEQGGETRWHKTGKTRPVFTGGKLKGYKKILVLYTNYGKQRKPEKTNWVMHQYHLGSDEEEKDGELVVSKVFYQTQPRQCGGGSAATAKDLSVDLVAGNNIKASNAAAEHHHNDGVGGGGHGGNNSSMLKEAAGIVDFYNPAAALIGYSQAAPNNRAAASAHLTMPNFEVHTGGAGFGP
LOC_Os01g48130.2	MTWCNSFNDVRAVENNLATAAAVAAAKKQQQQQQVSQHVNLIKTCPSCGHRAQYEQLQAAAAATIQDLPGLPAGVKFDPTDQELLEHLEGKARPDARKLHPLIDEFIPTIEGENGICYTHPERLPGVGKDGLIRHFFHRPSKAYTTGTRKRRKVHTDEQGGETRWHKTGKTRPVFTGGKLKGYKKILVLYTNYGKQRKPEKTNWVMHQYHLGSDEEEKDGELVVSKVFYQTQPRQCGGGSAATAKDLSVDLVAGNNIKASNAAAEHHHNDGVGGGGHGGNNSSMLKEAAGIVDFYNPAAALIGYSQAAPNNRAAASAHLTMPNFEVHTGGAGFGP
LOC_Os01g48446.1	MSPSRPDEADPAADFGSHPTDQELVTKYLRRHVDSGGNPWRYVHEADVYAADPDDLTGKYSPAVASDGSRAWYFFTTVRSKSTGGQRRARAVGDGGCWHSEAGAKDVVGGIRSPRPIGRRQFFSFVNKEGPRRVRSGWIMVEIGLKYAQQNASSDELVLCKVYRSPRAPPAAAAANKSMAAPPPTATKSKTEEATPPPDDVKPVVAAAQTPDTKILRAAKEAAATGCKRKADVKSSGARRGKRLCSRCRAETSESDSETAVLDRSPSIEDETADSSEIHGSSDGKFIRFL
LOC_Os01g59640.1	MAESSESAWPQQSQQLQISSTMPAGSAWPEEENLENLEQPLPLLMPSSEDHREQQLVPVPWLQQDQDQEWHEQEQFLPLKNQNQEQLQDQQPLQDQEETRRYLGVPGIRFVPSDIELILDFLRPKLRGEQLPSYSYMHVCDVYSDHPKELTSKLGPSREGNWYMFSPRNRKYNKGKRPSRSTGQLGFWKSTTKNEAVLDALSDNMLIGYKACLTYHEYDESMPTPKLKKENAIKTPWKMWEFVCSNSNRPFDAEEEPMRLNDWVLCKVTNKDNKVTTKKFKPQRSKKPKKPKKLQQEEQPQNQGIVIRQPSESGSASSSHQEIPGSSLPGAGGDAAAAAATAAAVVDPMPLHMIPPSSWNYFSTGVTADGIVMDDSTGVDSYGCVDGAGALNFQRNIFYHR

LOC_Os01g60020.1	MEMAAAVGGSGRRDAEAELNLPPGFRFHPTDEELVVHYLCRKV<br>ARQPLPVPIIAEVDLYKLDPWDLPEKALFGRKEWYFFTPRDRKYP<br>NGSRPNRAAGRGYWKATGADKPVAPKGSARTVGIKKALVFYSG<br>KAPRGVKTDWIMHEYRLADADRAPGGKKGSQKLDEWVLCRLY<br>NKKNNWEKVKLEQQDVASVAAAAPRNHHHQNGEVMDAAAA<br>DTMSDSFQTHDSDIDNASAGLRHGGCGGGGFGDVAPPRNGFVT<br>VKEDNDWFTGLNFDELQPPYMMNLQHMQMQMVNPAAPGHD<br>GGYLQSISSPQMKMWQTILPPF
LOC_Os01g64310.1	MADGGGRRAPGFRFYPTEEELICFYLRNKLDGLRDDIERVIPVFDV<br>YSVDPLQLSEIHHEMLGGGGEEGEPWFYFCPRQEREARGGRPSRT<br>TPSGYWKAAGTPGVVYSADRRPIGMKKTMVFYRGRAPSGTKTA<br>WKMNEYRAFHYPDASSASASSAGAAAPPNHLPPQLRSEFSLCRL<br>YTRSGGIRQFDRRPLAGGGDENPGPSMAAAAASPEENDGSGSSM<br>QQLELMDQGGAVDPDWDQWDDLATLTALLYWPRD
LOC_Os01g64310.2	MADGGGRRAPGFRFYPTEEELICFYLRNKLDGLRDDIERVIPVFDV<br>YSVDPLQLSEIHHEMLGGGGEEGEPWFYFCPRQEREARGGRPSRT<br>TPSGYWKAAGTPGVVYSADRRPIGMKKTMVFYRGRAPSGTKTA<br>WKMNEYRAFHYPDASSASASSAGAAAPPNHLPPQVVPFYWRRV<br>NIQTCIF
LOC_Os01g66120.1	MSGGQDLQLPPGFRFHPTDEELVMHYLCRRCAGLPIAVPIIAEIDL<br>YKFDPWQLPRMALYGEKEWYFFSPRDRKYPNGSRPNRAAGSGY<br>WKATGADKPVGSPKPVAIKKALVFYAGKAPKGEKTNWIMHEYR<br>LADVDRSARKKNSLRLDDWVLCRIYNKKGGLEKPPAAAVAAAG<br>MVSSGGGVQRKPMVGVNAAVSSPPEQKPVVAGPAFPDLAAYYD<br>RPSDSMPRLHADSSCSEQVLSPEFACEVQSQPKISEWERTFATVGP<br>INPAASILDPAGSGGLGGLGGGGSDPLLQDILMYWGKPF
LOC_Os01g66120.3	MALYGEKEWYFFSPRDRKYPNGSRPNRAAGSGYWKATGADKPV<br>GSPKPVAIKKALVFYAGKAPKGEKTNWIMHEYRLADVDRSARKK<br>NSLRLDDWVLCRIYNKKGGLEKPPAAAVAAAGMVSSGGGVQRK<br>PMVGVNAAVSSPPEQKPVVAGPAFPDLAAYYDRPSDSMPRLHA<br>DSSCSEQVLSPEFACEVQSQPKISEWERTFATVGPINPAASILDPAG<br>SGGLGGLGGGGSDPLLQDILMYWGKPF
LOC_Os01g66490.1	MHMRRGRAAGGEGEAAAVVVMNRYDNNGHAAAAAAAAVA<br>GGGGGGGGNKAAGEVDGHEDDLVMPGFRFHPTEEELIEFYLRR<br>KVEGKRFNVELITFLDLYRYDPWELPAMAAIGEKEWFFYVPRDRK<br>YRNGDRPNRVTASGYWKATGADRMIRAENNRPIGLKKTLVFYSG<br>KAPKGVRSSWIMNEYRLPPADTDRYHKTEISLCRVYKRTGIDDGH<br>GQVSTARSSAHSRGGGAAPVQDNKQGSSSTSTPTPPPTPSKLHLLS<br>SECTSPPAIVTDHAAMVAHKAPSPRHHQQQQQLHAAKPCGGYL<br>QNSSMASAAGGDQQQQFQQDFAAALYQQYSKNTSGAFASTYSL<br>LNLVNAASMGSSAAAIDELSSLVGHGTPSYINPAAGSHNYSQFLH<br>LPTTPSSHQPTPAPLGTTTAAAAATLPMSLAAFSDRIWDWNNPIP<br>EAGGRDYSTSTGFK

LOC_Os01g70110.1	MAAEAASGGGGGYRMLPQAGLPIGFRFRPTDEELLLHYLRRKVM<br>SRPLPADVIPVADLARLHPWDLPGEGDGERYFFHLPATSCWRRG<br>GGGSRAGGGGGAWRASGKEKLVVAPRCGKRPVGAKRTLVFFRR<br>GGARTDWAMHEYRLLPADDHPPEANDVWVVCRVFKKTTTLAH<br>RRSPPSIRGAPRRRAAAADDDDMPSSPSSCVTDGGDAGEEGEESS<br>SCSVVASNCP
LOC_Os01g71790.1	MGKEMNLIREDEYGGGGVGFEPTEDELMLHFLRPQLRGFAPRVA<br>GAVVEADPCGAAPWELLARHGRREEGFFFSARARRKPSVRRTVA<br>GCGGGGGGGGAWMHSSTKNGQSVTDLGVVVRWCRINYCFYVR<br>GEMGQQRSTGWMMAEYEITDPRCYRRADDGEEDDFWVLCHVR<br>KSSRPQAAKISPAKPARRRKPAAAAAADVRAA
LOC_Os02g06950.1	MVEARLPPGFRFHPRDDELVVDYLSGKLRSGDGGAASGGGAAG<br>AGCPTPTLIDVDLNKCEPWDLPEIACIGGKEWYFYNLKDRKYAR<br>GQRTNRATESGYWKATGKDREITRKGSLVGMRKTLVFYRGRAPK<br>GERTDWVMHEFRQELDHANHHHHLKVLAHRFRFQFALDCIISH<br>SHASWQLDYMQEGWVLCRVFYKSRTEAVAAPTMESTLPPRYING<br>GTSRSPLPPLVDSSISFNHGGYEEVLPCFSSSHHQQPSPASMNASA<br>AADDDQDYHHLSEGQRHYSDKKMMRDVQNDQVTTRFDGHLA<br>VKREMSLKKDLSEDEQAAPNADAGGFSILLKYSVSKMTSLMKPIQ<br>RNISTLQEFLNQKKEAILEKVEIFTKLLLPSRLGSAVFQLCLEHLIK<br>NHKVGISWDGIWELSDWEVADNEVVLKMVGQCSAPADSKSKDL<br>KRLFDLLRPYYDQEGKDPHLFFEHLKFDFTDVLKTIVTDAKWEW<br>FWKYLLNHVFVMPPTGNTY
LOC_Os02g12310.1	MAMTPQLAFSRMPPGFRFQPTDEQLVVDYLQRRTAAQPCVTPDI<br>TDIDVYNVDPWQLPAMAMYGSDHDRYFFTMAAREAQARRTTPS<br>GFWKPTGTKKTIFVVAGGHEVPTAVKRRFVFYLGHHQPSGSNNN<br>NKTSWIMHEYRLMNSPRAAVPSSSSVNRLPTDDLTEEMVLCRISN<br>KDLPKPPFIHNSLLQFSSVGLNGDGYNYLILDHLEPPAMEYPNVGI<br>GNVDDAAAGTDDPGDLDEEIDDSMQRNHGG
LOC_Os02g15340.1	MHPNGAPLAVPPGFRFHPTDEELLYYYLRKKVAYEAIDLDVIREI<br>DLNKLEPWDLKDRCRIGTGAQEEWYFFSHKDKKYPTGTRTNRAT<br>VAGFWKATGRDKAIFLGSGGGTRIGLRKTLVFYTGRAPHGKKTD<br>WIMHEYRLDDDNVDVPEEGWVVCRVFKKKSIHQRGFDQPDMA<br>AAADEDELRYQLLHGAGMSSSPVDQKHVLLQEQLVAHGAHGG<br>GFVVPAFEASMHLPQLASADAAPCGGGGGGHVAFASMNPLDA<br>AGCGSQNMMTMKMAATSGGEMLLMSGGGVDGGRFGAAADW<br>SILDKLLASHQNLDQLFHGKVAGAHQQQQQMAMDAASSLQRL<br>PFHHYLGLEAADLLKFSM

LOC_Os02g18460.1	MAAAARSQGLSPGFKFNPSDQMLVELFLLPYLIDGELPVRGLVFVEDDHLGGLPLPPWILLDRHGRGDEDEAYFVAPMGAGDGARQVRSVAGGGKWVKQRSEGKGEVVVAPGGEAFLWENFSLNFHRDDRRSGSTGWVMHEYIVSPPAGSAVAASHRATHIAFTGHGQNRKRVPDGYVLVLDDAVPPAAAAAAPPPESEQSNQEEQEYAAYTDQIQQQCFVPEQQMSNQEYFPEAAAEQSNQQFFVPAEEQSSHQLFLPAEEQSNQQFFMPAEEQSSHQFLPAEEQSSHQFLPAEEQSNYQQFLPALEQMTQSNQEFAYGEQSQCYIVPEQQQLSNQEYAYSEQSQCYILPEQQQLSDQEYAYSEQSQCYILPEQQELSNQEAEYAFVCYDEQQQQQQQSNQEAEYAFACYDEQQQQQQQYLHGDLTSWQEPFVTSSSSSSQQFLGQEQLLPDGLLLDGFGEISQQQGDQEYAYCEESQCYIMPEQQQQSNQEAEFAFACYD
LOC_Os02g18470.1	MATAAESQGLSPGFKFNPSVEQLLCFFLLPYLQHRRLLVDGVVFLDDPASAPPWALLHRHGRGGEDEAYFIGPVPAGDGHGGRRQQQVSRTVTGGGGGKWIKQRTERPRGEEEPVVVFGGETFRWEEFSLNFHADERCRSGSTGWVMHEFAVVPPAGSRVAATHTACRIAFTGHGQKRKRVPDGYVFVDVHVQTAAAAAAVAPPLPMLSSYGEPPHEHFSDDHPPPHSYTYYTQEYQQFLPAAEQSDQEQEYCAPEQQNFQDYHVAAAEQTDQDYFYTEMINQEQDYAYQQQQQHLFHGDFLATSQQFLGQDHEVMFTGLGGGLVVSDNGEHASAAAPATEPPVHDVFLETLVPEPPENAYVDGAGESAMASASSAGGAPLLEQPFATPPQQFLDQEPAPAGLNDGGAMIYNNNGDGEHDAAPAAQPPARYYSGPVPAVDSVFLDKMREYLMADAKGLCRIDAPINNGEHAAAPAPAADDPLAAQHGHGDAPPLPVPPDAAELERVVGHLLREVEDIIKVAAAGGYGGSSDKPLSEFDKAQNQILAKLMAVFNQVAES
LOC_Os02g34970.1	MGGATNLPPGFHFFPSDEELVVHFLRRKVSLLPCHPDIIPTLLPHRYNPWELNGKALQAGNQWYFFCHLTQSRTSSNGHWSPIGVDETVRSGGRNVGLKKTLLFSIGEPSEGIRTNWIMHEYHLLDGDCVAGGSSNLTSSSSNRRSHRKRGHSSMESNNWVLCRVFESSCGSQVSFHGEGTELSCLDEVFLSLDDYDEVSLPNK
LOC_Os02g36880.3	MRLARQQQQVVVAATMEHDVHHHRQMMQQQQQQEMDLPPGFRFHPTDEELITHYLLRKAADPAGFAARAVGEADLNKCEPWDLPSRATMGEKEWYFFCVKDRKYPTGLRTNRATESGYWKATGKDREIFRGKALVGMKKTLVFYTGRAPRGGKTGWVMHEYRIHGKHAAANSKQDQEWVLCRVFKKSLELAPAAAAAVGRRGAGAGTDVGPSSMPMADDVVGLAPCALPPLMDVSGGGGGAGTTSLSATAGAAAAPPPAHVTCFSNALEGQFLDTPYLLPAADPADHLAMSSASPFLEALQMQYVQDAAAAGGAGMVHELLMGGGWYCNKGERERLSGASQDTGLTSSEVNPGEISSSSRQQRMDHHDASLWAY

LOC_Os02g36880.1	MRLARQQQQVVVAATMEHDVHHHRQMMQQQQQQEMDLPPG FRFHPTDEELITHYLLRKAADPAGFAARAVGEADLNKCEPWDLP SRATMGEKEWYFFCVKDRKYPTGLRTNRATESGYWKATGKDREI FRGKALVGMKKTLVFYTGRAPRGGKTGWVMHEYRIHGKHAAA NSKQDQEWVLCRVFKKSLELAPAAAAAVGRRGAGAGTDVGPSS MPMADDVVGLAPCALPPLMDVSGGGGGAGTTSLSATAGAAAA PPPAHVTCFSNALEGQFLDTPYLLPAADPADHLAMSSASPFLEAL QMQYVQDAAAAGGAGMVHELLMGGGWYCNKGERERLSGASQ DTGLTSSEVNPGEISSSSRQQRMDHHDASLWAY
LOC_Os02g36880.4	MRLARQQQQVVVAATMEHDVHHHRQMMQQQQQQEMDLPPG FRFHPTDEELITHYLLRKAADPAGFAARAVGEADLNKCEPWDLP SRATMGEKEWYFFCVKDRKYPTGLRTNRATESGYWKATGKDREI FRGKALVGMKKTLVFYTGRAPRGGKTGWVMHEYRIHGKHAAA NSKDQEWVLCRVFKKSLELAPAAAAAVGRRGAGAGTDVGPSSM PMADDVVGLAPCALPPLMDVSGGGGGAGTTSLSATAGAAAAPP PAHVTCFSNALEGQFLDTPYLLPAADPADHLAMSSASPFLEALQ MQYVQDAAAAGGAGMVHELLMGGGWYCNKGERERLSGASQD TGLTSSEVNPGEISSSSRQQRMDHHDASLWAY
LOC_Os02g36880.2	MRLARQQQQVVVAATMEHDVHHHRQMMQQQQQQEMDLPPG FRFHPTDEELITHYLLRKAADPAGFAARAVGEADLNKCEPWDLP SRATMGEKEWYFFCVKDRKYPTGLRTNRATESGYWKATGKDREI FRGKALVGMKKTLVFYTGRAPRGGKTGWVMHEYRIHGKHAAA NSKQDQEWVLCRVFKKSLELAPAAAAAVGRRGAGAGTDVGPSS MPMADDVVGLAPCALPPLMDVSGGGGGAGTTSLSATAGAAAA PPPAHVTCFSNALEGQFLDTPYLLPAADPADHLAMSSASPFLEAL QMQYVQDAAAAGGAGMVHELLMGGGWYCNKGERERLSGASQ DTGLTSSEVNPGEISSSSRQQRMDHHDASLWAY
LOC_Os02g38130.1	MARSWLITGRGVAKKIRNAPHCSSRPISELGAEAQMECPNCKHVI DNSDVAIQWPGLPAGVKFDPSDLELLEHLEQKIGLGGSKPHTFID EFIPTIDNDEGICYSHPENLPGMKKDGTSGHFFHRVSNAYGCGQR KRRKISNCDHVVSVEHVRWHKTGKSKAIVEKGVTKGWKKIMVL YKSSQRGAKPDKANWVMHQYHLGAEEDEKDGELVVSKISYQLH GKQIDKSETGNADEESDAFAARVGPKTPKSNTPQPCRLKNSPCET ENYDPILEDQDEEESNIPIVSLKDDAGNPAWCAGETQAAREAVQ ACPNLDESLRCHEVLDSFYHETLLPSDRPILSQGGNEILDRNLNAV YGLPDLYNVDLGTPPDFQLADLQFGSQESIGNWLDSI
LOC_Os02g41450.1	MRVFVLSKFIVVAICAYTNAEVAKLTASEWYFFSFRDRKYATGSR TNRATKTGYWKATGKDREVRGSSSSSSRAVVGMRKTLVFYQGR APNGVKTGWVMHEFRLDSPHSQPREDWVLCRVFQKRKGDGDG PQDSGGAASPTFTGSMSTTTLSQLQPPDHRRHAAAAAGGYYVGS QQLAAGYDSAAGFANPTQPAVPHYQYGGAVIGFPEEFGGGGGV ADEYGFGTYLDLGFELDDTASVLGGIRSFPQGWN

LOC_Os02g42970.1	MDTFSHVPPGFRFHPTDEELVDYYLRKKVASKKIDLDVIKDVDLY<br>KIEPWDLQEKCKIGMEEQNDWYFFSHKDKKYPTGTRTNRATGA<br>GFWKATGRDKPIYARSCLVGMRKTLVFYKGRAPNGQKSDWIMH<br>EYRLETNENGTTPEEGWVVCRVFKKRVATVRRMADGSPCWFDD<br>HGAVGAFMPDLSSPRQLLPHHHHHHPGSSAALYHGHHHQQLQ<br>QMYGHCKPELEYHHLLPQEAFLQHLPQLESPKPPPPPPAAAAYIG<br>GHLGSSSSTALTTHDDEASGSAAQQQPPSLEAVYMAGAGVGIGV<br>DASVTDWRLLDKFVASQLLSKESMSSYGSHPAQVFQAADGGKH<br>EEALDYASTSAGSGGGEADLWK
LOC_Os02g56600.1	MGLRDIELTLPPGFRFYPSDEELVCHYLHNKVVNQHRFAGVGGA<br>AAAGGGTMVEVDLHTHEPWELPDVAKLSTNEWYFFSFRDRKYA<br>TGLRTNRATKSGYWKATGKDRVIHNPKLHAAAHRRASIVGMRK<br>TLVFYRGRAPNGVKTNWVMHEFRMENPHTPPKEDWVLCRVFY<br>KKKAETETESSYSMENEQEAVIAMARSAAAIKAGGCYSNSSSSHD<br>PAAAGHHSPPPFPASLAACSSSHHYSSHPPPPPDHHHHHHMPVT<br>GGGGGSLNEFIPTTSMALYSSIFDFSQHLDGGAVAASASAAGSRV<br>DGGEQCGLMELGLEEHYNYNGLMPM
LOC_Os02g57650.1	MSQSPPDSSSAAAVPLAPGFRFHPTDEELVSYYLRRRILGRRLRID<br>AIAEVDLYRLEPWDLPSLSRIRSRDAQWYFFARLDRKVTGAGAGG<br>RGGPGNRTNRATPRGYWKTTGKDRDVHHRGKLVGMKKTLVFH<br>SGRAPKGQRTNWVMHEYRLLDADGTQDLHVVCRIFQKNGSGP<br>QNGAQYGAPYLEEDWEEEDDAIENMPASGAFAEMAAVTDTAD<br>ESTEEDGNFSLKTNDEPLQTQEYQPEITPVKAQGSNEETNGGGYS<br>CDVFSLDEILQEPENVCKNEEQNAIDDKFTIAELSGYPRQDDGYV<br>GENGPVNWIDPSNGDNTNWPLRAYSTQNHVNGTLSADGFFDTV<br>NGTNSYSGPSDNQNLYLQDDGLTSSHQVGDNMPFYDASSNHK<br>WVDGKDDYLNLNDLLYPPAENQPLFDAGDDLMAYFDATEDDF<br>KFDIMGTEDSNSQLPDMSNFVQKDDNNNKFTLDGISNTALYGAS<br>SSGSHGNMYPDTAVPDMPMDDTVDKSFGKRLASMLGSIPAPPA<br>MASEFPPSTGKSVVPLSAVNPSSSIRVTAGIIQLGGITFTGSTEHLQK<br>NGDFNLLLSFTVEGDVSTKSIGFEPDTQMSTTPMVLRSGMYLFFVS<br>AMILMLSYKVGLCIYSR
LOC_Os03g01870.1	MDGLISDDSMMMGGEVRAIESRLPPGFRFHPSDEELVGYYLRNK<br>QQQQQQQTAATSMLVEVDLHACEPWDLPEVAKVGSDEWYFFS<br>WRERKYATGWRRNRASKQGYWKATGKDKPILHPTVAGARKTL<br>VFYSGRAPNGRKTAWVMHEFRLLHHHHHPNPNIQNMQQQEG<br>DDWVLCRVFRKGNNSNGQPLATSSPPAHHLVESLISSPAPTIMSD<br>HDRLFTIQLPHHQHCDEQYFFLDDDEQHQQQLLDLSVLQAPTSF<br>ESEQAPGHGGMEINIAEMESFDTTCAALQDASDYCMQLY

LOC_Os03g02800.1	MAPVSLPPGFRFHPTDEELIIYYLKRKINGRQIELEIIPEVDLYKCEP<br>WDLPEKSFLPSKDLEWYFFSPRDRKYPNGSRTNRATKAGYWKAT<br>GKDRKVNSQRRAVGMKKTLVYYRGRAPHGSRTDWVMHEYRLD<br>ERECETDTGLQDAYALCRVFKKTAPGPKIIEHYGVVHHHVEQPQ<br>WMTSSIDRSPTLDVSCDGRGDDFESSSFSFPTETPMDSMHGGFGM<br>QMSAPHEDGKWMQFLSEDAFNATNPFLTNPVSANFSCLPSKVD<br>VALECARLQHRLTLPPLEVEDFPQDVSLDTKIGILRSNPNEVDILQ<br>EFLSVATASQELINGSTSSYPEMWLGASTSSASYVNELSSLVEMGG<br>VGTSNHHESARLQVEIADMEVFKDEKKRVENLRGVKLVNNDLG<br>EIVVEGDESNPTEDIIAQYPIKVTADNSGEAGHRMTDPTDVGGID<br>TAPIFSQSQPDDFAAGFDDVNPNASFDLYEKVDVNHRLFVSRVA<br>AAKTFFHRIEPSKKVSFHSNPAATAVSKATEKFHFPVTTKVSGRVS<br>IFSKFKALIRDKFLMMRPSHSYQRLGSKETTVNELLQIVSLLLAPK<br>QINGCPTEQELVKKKAKEVMKPGWGREGSNKLWLPLSKGKGISS<br>MFLSGKWTFLTSALAISTPAECDH
LOC_Os03g03540.1	MESCVPPGFRFHPTDEELVGYYLRKKVASQKIDLDVIRDIDLYRIE<br>PWDLQEHCGIGYDEQSEWYFFSYKDRKYPTGTRTNRATMAGFW<br>KATGRDKAVHDKSRLIGMRKTLVFYKGRAPNGQKTDWIMHEYR<br>LETDENAPPQEEGWVVCRAFKKRTAYPARSMVETWDYSLHERNI<br>MSAAAAAAFADPSAAYAQMRRQHRSGRFKQEAELDGAATALL<br>HYSSHLAELPQLESPSAAAAPLQPNPSQLATAGEDDDCKGDNGG<br>RRAKKARAAGDKVATTTDWRALDKFVASQLSPGECGSMEATAE<br>AAAAAVAGVSSPLDHGDDDMAALLFLNSDERDEVDRWTGLLGS<br>GAGASGVDGDLGICVFDK
LOC_Os03g04070.1	MAAAVAVAGPSMEVEQDLPGFRFHPTEEELLDFYLSRVVLGKKL<br>HFNIIGTLNIYRHDPWDLPGMAKIGEREWYFFVPRDRKAGNGGR<br>PNRTTERGFWKATGSDRAIRSSGDPKRVIGLKKTLVFYQGRAPRG<br>TKTDWVMNEYRLPDYGAARAAAPPPKEDMVLCKIYRKATPLKE<br>LEQRASAMEEMQRGSSHGDYTATRASLVHDASASTGDDYFSSDD<br>VHDSGFLIQSSSSSAAPSGSSSKNGGAGAPREAKKEEADVTVTVAS<br>ATSLQLPAVSQLPSLQLPAMDWLQDPFLTQLRSPWQDQHCLSPY<br>AHLLYY

LOC_Os03g12120.1	MAKDKEVGPPPPPCVDSDHEDLPLAERRRRLLRPPAESKPPAPER<br>REASAAAAAAEDSGGAAQQGWPGLPRGVEFNPTDSDLLWHLA<br>AEVGNGQARRHPFINEFIKSVDETIGFGYTHPQDIPGIRQDGCASY<br>FFHKNFKECANENSKCIRWQKSGNPISITLDGNLQGCKEVFVLYA<br>YETDGNNPQITDWRLHQYHIESTEKDEGELVVSKIFYELEKNQFK<br>WAEKSHAQSAQGASAIDDDSKEELQLDNHSFNMITENSSVQGNE<br>NKQKQTQTGTCPNLDKLSYFNVVSNMHIGNQINDHDEIEELDH<br>MSLQERYRILMAENHSSSAVVSSEQCAIDGLENSCKPGTNGMIPK<br>RIHEGTAFRDGMYSMLQEISSAPAIIGSIDNDNNRRLLTEGLSNNQ<br>QSHEAGCESGFLSTSSSAAPPQCQVVCSHDLLVNGKTLIYSRDPSS<br>SSTPTFGDKNIQLEGTDDRTLLVDIKLEPALEGDFTEKITSSVQRTD<br>PNHGTEGSNLVGSINSVSSAISKRISEAARSNPENSHVEGLLPSSRI<br>KSEVTGSELPLVVCGLTSISIAELTAKKTNTLNHDGVLAYCSRKRK<br>RRKTLRDPSEKTLEEDSLRNDEGTAYFSRQRRRRKTATDSIETALE<br>EDAPGLLQILLDKGILVKEIKLYGVEEEDDMVPDCTESDFQDLEN<br>VITKLFPQRTSLLKSALRHEKGEKAIYCLTCLISLIEQSRYLQFRDCP<br>VEWGWCRDLQSFIFIFKSHNRIVLERPEYGYATYFFEIVKSLPIQWQ<br>IQRMVTAMKLSGCGRTALIENRPLLIGEDLTEGEARVLEEYGWVP<br>NSGLGTMLNYRDRVVHDRWNERSGTDWKTKIGKLLMNGYSEG<br>HLVLSHFPTKVGKIEDDTEIKQEDPL
LOC_Os03g21030.1	MSEVSVMAEVEETAAAAPLDLPPGFRFHPTDEEIVSHYLTPKALN<br>HRFSSGVIGDVDLNKCEPWHLPAMAKMGEKEWYFFCHKDRKYP<br>TGTRTNRATESGYWKATGKDKEIFRGRGILVGMKKTLVFYLGRA<br>PRGEKTGWVMHEFRLEGKLPSQLPRSAKDQWAVCKVFNKELAL<br>AAKNGPMAVTEATADDAGIERVGSFSFLSDFIDPAELPPLMDPSF<br>VADIDGVDDAKVSASTSGQAAIAAGFHVASQVMSYQQVKMEEP<br>LPLPYLHQQPPRMLHSGQYFSLPAVHPGDLTPSAIRRYCKAEQVS<br>GQTSALSASRDTGLSTDPNAAGCAEISSAPTSQPFPEFDDGILGLD<br>DFWN
LOC_Os03g21060.1	MVLSNPAMLPPGFRFHPTDEELIVHYLRNRAASSPCPVSIIADVDI<br>YKFDPWDLPSKENYGDREWYFFSPRDRKYPNGIRPNRAAGSGYW<br>KATGTDKPIHSSGGAATNESVGVKKALVFYKGRPPKGTKTNWIM<br>HEYRLAAADAHAANTYRPMKFRNTSMRLDDWVLCRIYKKSSHA<br>SPLAVPPLSDHEQDEPCALEENAPLYAPSSSSAASMILQGAAAGA<br>FPSLHAAAAATQRTAMQKIPSISDLLNEYSLSQLFDDGGAAAAAP<br>LQEMARQPDHHHHQQQQHALFGHPVMNHFIANNSMVQLAHL<br>DPSSSAAASTSAGAVVEPPAVTGKRKRSSDGGEPTIQALPPAAAA<br>AKKPNGSCVGATFQIGSALQGSSLGLSHQMLLHSNMGMN

LOC_Os03g39050.1	MAGADGLPPGLRFDPSDDELVSRYLLRRIQQKPLPLDGVIVDADP LSVPPWTPLADHTRGDEAFFFAGARAKNGKGKRQKRTVEGGGF WQGQRMAVDGERLVVPGDGGGGVDGSGGGGLEITWRKYVLSFF AEGEQGSSGWVQKRKREPQCLDSHDDEDGGDQERAAPRRGVAL PAADGTDQGSYGVIDGEPSLVSHCLPDQIVPPAEEADATAGAVD EERWSPPQPASPTAALVKQNSYDLMVISSLLFSDLPDRIDDDDLSV SQTEGTELSEQGSSGVIDDDYWREADATGGAEREEIALLDEERCP QPQPAPPTDALVPPLQGQNSYDVMADSSLLFADLPGRIDDDELQ RSLRVSDMPDLFLSQTEEAGAGGGGGAAPVLNKQSNSSPLGVMD SEVPIVLSDLEFPESIDEVLSYIDFATDDCLDFDMDELFSDMPAD
LOC_Os03g39100.1	MAGADGLLPGLRFDPSDDELVSRYLLRRIQQKPLPLDGVIVDADP LSVPPWTLLADHTRGDEAFFFAEARAKNGKGKRQKRTVEGGGF WQGQRMAVDGERLVVPGDDGGGGGDEGLEITWRKYVLSFFAEG ERGSSGWVMHEYAVTSPADLASSQLRLYRVRFSGHGKKRKREPQ CLDSHDDDDGGDQESATHRRAVAETTLFDGYVPRPAADGTDQG TYGVIDGESSLASHCLPDQIVPPAEEADATAGVENPLLDEERWSP PQPALVKQNSYDLMAISSLLFSDLPDRIDDDDLSVSQTEGTELSEQ GSSGVIDDDYWRVFHGLSDLIALPAEEADATGGAEREEIALLDEE RCPQPQPAPPTAALVPPLQGQSSYDLMADSSLLFADLPGSIDDDE LQRSLRASDMPDQFLAQTEEAGAGGGGGAAAALNKQSNSSPLG VEVPMALSDLESPESMPLSDLEFPESIDEVLSYIDFTTDDTSCLDFD MDELFSDMPAD
LOC_Os03g42630.1	MEEGLPPGFRFHPTDEELVTYYLARKVSDFGFATRAIADVDLNKC EPWDLPSKASMGEKEWYFFSMRDRKYPTGIRTNRATDSGYWKTT GKDKEIFHGGALAGMKKTLVFYRGRAPKGAKTSWVMHEYRLQS KFPYKPAKDEWVVCRVFKKLQCHLAKPRPPHDDVDGDGASPPE MVDASSLGELGELDVSSILLGGFAPPSGELCHGGGGGDGFGAHR LHVGAYMSWLQAAAAANQGMFQWPAATQAGLVGGTVFAAA HKAAGTMPFGGGCSQQQARDVGVSLANVGGGDALFGGAPLAK VDMECGEQAPQLDMDDSTWRAF
LOC_Os03g56580.1	MVTSKEFARDQAAMDQKIKSDVGEVVLAGDEEEDGDVVLPGFR FHPTDEELVTFYLRRKVARKSLSIEIIKEMDIYKHDPWDLPNASTV GGEKEWYFFCLRGRKYRNSIRPNRVTGSGFWKATGIDRPIYSAAV NSNSGESIGLKKSLVYYRGSAGKGTKTDWMMHEFRLPPAIAAAD ASPCMQEAEVWTICRIFKRSITYRKQQQQQAWRPPATVTVKAPPP GDSSSNTGSFESDGGGDEFMNCGLTPAISQQQQHGGRHQMMST MSCNGGYFFNDGIHHSHSHHKLHSQWGSLQMAPPEPKPEPEQK PLSSPAMTIAFHQNDHGFPAAAADFYKDGYLEEIARMMEVADPS PTGFYDCRY

LOC_Os03g59730.1	MAAINPVAGEVAAATAKAPPPAMATVRAPLPANHYSPYHSASA<br>AGSYAANTQSTSSPVSPASPAMISSSSSSLPPQQQRTWQPQPTTFS<br>QANPGHAYQQDHLPAVAGRRFFPPPAMQMQYYHQQPDGVAM<br>VGSGHPMAAPVHSSPLATTSGSNHAVVPDAPPQEPAKRRRRNT<br>AAAATARRGRGRPRGATASSAHSAPPPPQQQQQPTTSAPAITAQ<br>RNDDVNQEDDNQSSKNSAEEAVVVDGGEPPAATSALAIVPRHG<br>DVGDADRPVSPYSDIPGVRFTPTDQELIIHFLKPKYNLRDAMPTNI<br>IVIKQLDVCKLNLDELHGDLGLGKSLDGAWYVFSPRSRYKERGVR<br>PARGIKTTAVGYWKSNSAEADVVDDDGEVIGRVNSLTLALGHQP<br>RGKATHWRMKEYRIPQFQIPLGQEDSNRLLTPMLSQDIVLFYFLQ<br>LDEWVLCKLYHSFAYKQKGKCKVHEEGSKSDRGVQDLSIDDDRK<br>TCDIEANKPNGV
LOC_Os03g59850.1	MASPEDGEEKMAGKNKHGFPLGFRFVPEDQELLDIPDDKLRGAP<br>LDRAHDAVFHEARILDFHTAKLYGATMGLAAMPFAAGMRHAW<br>GRLFRWRGREALRAWQLPADRRLWGDPGSSASGWASSPRLTLCL<br>LSSKPHAVVGVGHVAAAAAAEEGAGGWQEKGHLDALALALAP<br>STSPSASPGSPPAATPSSAPRHDAHPAHGGFAHRQYPAFLTGEQE<br>RWTRGGHDTKAVSDVVDIVVAARRVGVGEDSCGAGGAAPFDD<br>DRYVEGHVGVFKLGLFSAQSETIRVLEFYVKGVRTNWGMHEFIRII<br>GPDNEVTAKTLRCVSVIQVTAKAPI
LOC_Os03g60080.1	MGMGMRRERDAEAELNLPPGFRFHPTDDELVEHYLCRKAAGQR<br>LPVPIIAEVDLYKFDPWDLPERALFGAREWYFFTPRDRKYPNGSRP<br>NRAAGNGYWKATGADKPVAPRGRTLGIKKALVFYAGKAPRGV<br>KTDWIMHEYRLADAGRAAAGAKKGSLRLDDWVLCRLYNKKNE<br>WEKMQQGKEVKEEASDMVTSQSHSHTHSWGETRTPESEIVDND<br>PFPELDSFPAFQPAPPPATAMMVPKKESMDDATAAAAAAATIPR<br>NNSSLFVDLSYDDIQGMYSGLDMLPPGDDFYSSLFASPRVKGTTP<br>RAGAGMGMVPF
LOC_Os03g61249.1	MADDDEIALEPGYVFHPSDDGLITLFLRPSIAKIPFEDRLINHADV<br>YSANPAELVGEHRPAPGTHGSSSVWYFFCSPRFTSKRKTSGRRQR<br>AVGGGGGGESVWKSEGGKKAVIGADGRRVGYLQKFSYGVYESSS<br>SGSARTFTRLGWCMTEYGLDDDAIDGADKQVLCKVYRSPRAVC<br>AEARTAAAAKSADSPCSGSKRKADDGADHPEAPPSARPRQEEA<br>GSEQPAILPELDLDALLSAPMDDSLGVEFDTATTEQYMRYLMND<br>EPLPWAPTMEVAGGGDEFIETTNGPCMGEEEIIQRLASGETLDDIL<br>GSNPN
LOC_Os03g61319.1	MADDDEIALEPGYVFHPSDDGLITLFLRPSIAKIPFEDRLINHADV<br>YSANPAELVGEHRPAPGTHGSSSVWYFFCSPRFTSKRKTSGRRQR<br>AVGGGGGGESVWKSEGGKKAVIGADGRRVGYLQKFSYGVYESSS<br>SGSARTFTRLGWCMTEYGLDDDAIDGADKQVLCKVYRSPRAVC<br>AEARTAAAAKSADSPCSGSKRKADDGADHPEAPPSARPRQEEA<br>GSEQPAILPELDLDALLSAPMDDSLGVEFDTATTEQYMRYLMND<br>EPLPWAPTMEVAGGGDEFIETTNGPCMGEEEIIQRLASGETLDDIL<br>GSNPN

LOC_Os03g61650.1	MAEIGKGLVFSPSDDQLTDGYLRSYLVRTSLDDLPSAATSYFHVA DVYSAPPDQLVAGLAPAPGTGDGDGRVWYVFTPVRVLGSRGAR KARTVGGGCGECWHAEGGPKDVKGSAAGGKLQKFSYKIKTALG AVVKPGWLMVEFSFPGSDHLALCKVYRSPRTSRYGAPSPPSSAAS SPSRAAPPPVSSTSGRKRKAEEESDHPEAPASSAPRRTLPASEQHV DVDAAAASEPDQGGYLSTDQLDSVAAFVQEHEAFVQEHEGDEE FCKSLGFDERSDPQCWTNFFLSALEEFGPAPETDAAAVAVAAVEP GPCPEYEEHDDTATTAASSHAYDSATAELVNLSDKEFYDIIFSGD QQGGAAVAG
LOC_Os03g62470.1	MDDDEIALEPGYAFRPSDDGLITLFLRPKIAKIPFEHRLINHADVYS ADPTELVGEHRPAPGTHGSGSVWYFFCSPRYTSKRKASGRRQRA VGGESVWKSEGGKKAVIGADGRRVGYLQKFSYHYTGQGHL
LOC_Os04g35660.1	MAGASNLPPGFHFFPSDEELIIHFLRRKASLLPCQPDIVPTLILNLY DPWELNGKALQSGNQWYFFSHATQTRTSPNGHWKPIADETVISG GCNVGLKKTLIFFIGEPFEAIKTNWVMHEYHLMDGSTNCSSSSTSS SSSKRSHKKKGHSDTESKNWVICRVFESSYDSQVSFHEEGTELSCL DEVFLSLDDYDEVSFAK
LOC_Os04g38720.1	MEQHQGQAGMDLPPGFRFHPTDEELITHYLAKKVADARFAALA VAEADLNKCEPWDLPSLAKMGEKEWYFFCLKDRKYPTGLRTNR ATESGYWKATGKDKDIFRRKALVGMKKTLVFYTGRAPKGEKSG WVMHEYRLHGKLHAAALGFLHGKPASSKNEWVLCRVFKKSLVE VGAAGGKKAAVVTMEMARGGSTSSSVADEIAMSSVVLPPLMDM SGAGAGAVDPATTAHVTCFSNALEGQFFNPTAVHGHGGGDSSP FMASFTQYGQLHHGVSLVQLLESCNGYGGLVDMAASGSQLQPA ACGGERERLSASQDTGLTSDVNPEISSSSGQKFDHEAALWGY
LOC_Os04g39960.1	MASSAHMAAVLNLPWGYRFRPSDRQIIANYLGPMAIHGADSLP QRGDVVEGVDVFATRPAAIPFEPRRHVFGRDEVRAYFFGDQPTDS RGREVPGGAWLPCGGGDKAYSGGADGGEAVAYRRKYEFRAAN EEADRAGEEAATPARPRWRMKEYRLNKSAAEFRRAYAQPNPKA NMDCVVREIYTKAVPPPTPPSGRSGDEEMQEGSDYSVMDEDELV DYLLQGFEDGNFDEDQDQPAAAEDGDYSDEDEDQPAAAEDGD YSDEDQDQPAAAEDGDYSDEDEP
LOC_Os04g40140.3	MAQNWKIQQIYHNGVLKGWKKILVLYKGSKKNKIVQANWVM HQYNLGVEEGGEDGELVVSKVFYQLSSKQTGTPEMDSVTEEASD ALTIRSDPITPITNPPLPRCLMNSPCDTEQNGTISHDQEGECSTSTL RPMVEAGNRAGCSAGASTAGDFNEDLLQRCEFPEDPVPTLDDTL PFLYTDETDLFSWEDFQFGSQESFGWVDGDHT
LOC_Os04g40140.1	MAQNWKIQQIYHNGVLKGWKKILVLYKGSKKNKIVQANWVM HQYNLGVEEGGEDGELVVSKVFYQLSSKQTGTPEMDSVTEEASD ALTIRSDPITPITNPPLPRCLMNSPCDTEQNGTISHDQEGECSTSTL RPMVEAGNRAGCSAGASTAGDFNEDLLQRCEFPEDPVPTLDDTL PFLYTDETDLFSWEDFQFGSQESFGWVDGDHT

LOC_Os04g40140.2	MAQNWKIQQIYHNGVLKGWKKILVLYKGSKKNKIVQANWVMHQYNLGVEEGGEDGELVVSKVFYQLSSKQTGTPEMDSVTEEASDALTIRSDPITPITNPPLPRCLMNSPCDTEQNGTISHDQEGECSTSTLRPMVEAGNRAGCSAGASTAGDFNEDLLQRCEFPEDPVPTLDDTLPFLYTDETDLFSWEDFQFGSQESFGWVDGDHT
LOC_Os04g42940.1	MLPMRPRASQAAAPETAAAAESLREEETEDGWVFLAGRSRATRPPPPPPSPMARAVASGSSGGGGGQPFDPTAEDIVNRYLPLRRALRCDALPRQVHDADVYGAHPALLASVYPAANERFEWFFFVCRRQCPGGRRRAGPGDYRLSQEAKHRGNAFCHSFRYYEYEDAGGGFRETEWRMVEYGDRGRDAGAGGSEGFELVVCKVYPARGGALHERLGADRAVLATRHRADEDAKPQVLVQLYLASLRLGNPLACRVHRADDVFDAHPAVITAALPAANDRCEWFFAAVRPRGHAQGHGDGAPPRPRKAGPGAYVPVRECRVVDGRRGDMGCRLVFWYREDDEEARRASRRTEWWMDEYRFGPDFPYGELPAPMARGEDEELVVYKVYPRLVGNRR
LOC_Os04g43560.1	MGLREIESTLPPGFRFYPSDEELVCHYLYKKVSNERASQGTLVEVDLHAREPWELPDVAKLTASEWYFFSFRDRKYATGSRTNRATKTGYWKATGKDREVRSPATRAVVGMRKTLVFYQGRAPNGVKSGWVMHEFRLDSPHSPPKEDWVLCRVFQKSKGDGEQDNPTSAASPAATFAGSSQAAVPGQAAYSSDDHTGSSMGFAPRQNEILDSSSHQLLNLAMLQCNSVLDHFPQEVNSSPMMGLAGSIGIGDEYGFFYDTGFEETASLGGMRFPQGWS
LOC_Os04g52810.1	MVFFTCHESLAYFLHNLIIFPARARARAAGRRRREDAARVGATEWYFFSLHDRKYATGQRTNRATRSGYWKATGKDRAIVTRRRAAAGEAVAGGEVVGMRKTLVFYQGRAPRGSKTEWVMHEFRVDGHAVADHPSSSTSSSSNLLKEDWVLCRVFYKSRTATPRAVVSGEAAVSLSGELSLPPPLPPPVAPAVVDGYTGGGYYEQDSSAGYHHHHHHRPPPSAALPFKDLTDFRDLLSNMAQGGGGGAAAAKTEGFHLGWSEEESSGYVQQSAMASQAWNPF
LOC_Os04g59470.1	MEEEWCCVAPGFRFHPTEEELVGYYLARKVVGQQDDGIIQEVDLNSIEPWDLLQAQQHDQEYYCYFFSYKDRKYPSARGTGTRTNRATAAGFWKATGRDKPVLSSSRSSSSPAVIGMRKTLVFYRGRAPNGCKTDWIIHEYRLVAHHQQPDGSCWVVCRAFHKPTTTTLQHQLHLHRPAPLLHHPGYYDDQYLYPPPAAAAGGGGLLCSPALDMELEDEEDESKMMILSNDNIPLVVSPTAVHTQGTGGDIINDATTAPAAAAAADHRRHLAPPPPLHLLANSVD

LOC_Os05g10620.1	MGAGSIQARMSSDGASGSIGMKHDDGDHRPSTGASSRRCPSCGHDPDCNKPFDMVGMPAGVRFDPTDQELIEHLEAKVKDGGSTSHPLIDEFIHTIQGEDGICYTHPENLPGVTRDGLSKHFFHRSAKAYPTGTRKRRKVLADQQPDDHPQASKGRNVAAAETRWHKTGKTREITVRGQPKGCKKILVLYTSFGKKRKAEKTSWVMHQYHLGELDDEKEGELILSKVFYQTQTRSAAAAEAPVSSGAAMEVQGQQQQVLKLQADDGHFSSAPTKKRLHQDVVAQVKVDRGHHCMPAQRQVNFNLKVTPVPTTSSFPVVVDKQLYSPVALFRSEHLHVGKNFNSSAPKSRLASPALAS
LOC_Os05g10620.2	MSSDGASGSIGMKHDDGDHRPSTGASSRRCPSCGHDPDCNKPFDMVGMPAGVRFDPTDQELIEHLEAKVKDGGSTSHPLIDEFIHTIQGEDGICYTHPENLPGVTRDGLSKHFFHRSAKAYPTGTRKRRKVLADQQPDDHPQASKGRNVAAAETRWHKTGKTREITVRGQPKGCKKILVLYTSFGKKRKAEKTSWVMHQYHLGELDDEKEGELILSKVFYQTQTRSAAAAEAPVSSGAAMEVQGQQQQVLKLQADDGHFSSAPTKKRLHQDVVAQVKVDRGHHCMPAQRQVNFNLKVTPVPTTSSFPVVVDKQLYSPVALFRSEHLHGRTSIHQPQKADWPHQRWPPDNQDQHG
LOC_Os05g10620.3	MSSDGASGSIGMKHDDGDHRPSTGASSRRCPSCGHDPDCNKPFDMVGMPAGVRFDPTDQELIEHLEAKVKDGGSTSHPLIDEFIHTIQGEDGICYTHPENLPGVTRDGLSKHFFHRSAKAYPTGTRKRRKVLADQQPDDHPQASKGRNVAAAETRWHKTGKTREITVRGQPKGCKKILVLYTSFGKKRKAEKTSWVMHQYHLGELDDEKEGELILSKVFYQTQTRSAAAAEAPVSSGAAMEVQGQQQQVLKLQADDGHFSSAPTKKRLHQDVVAQVKVDRGHHCMPAQRQVNFNLKVTPVPTTSSFPVVVDKQLYSPVALFRSEHLHVGKNFNSSAPKSRLASPALAS
LOC_Os05g25960.1	MAAADGLLPGLKFDPSDHDLVGRYLLRRLQGQPLPLDGVILEADPLSAPPWKLLADHGRGDEAFFFAEAHAKNGKGKRQKRTVEGGGFWQGQNTCVDGERLCVPDDGDGSGGGGGGLEIAWRKYVLSFFANGERGSSGWVMHEYAVTAPDGLASSQLRLYRVRFSGYGKKRKREPQCPGAHGDDDGELQCAPPPRSMAETALLEERGPLPHPVLGPASVVDQCTDQGSSGVIDDSSLVFRDLPDLIDLPVAEEADASHGAETALLNEHLPLPPPQLFVPPTAVPLDLADDSNGADQNSYGMMGDDQLLLPDLPGTINDDMPDLFVSQAEEASAVPAISYHSSGFMGNEVAALSDFELPESYSSSDAMDGEALALSNYEFPESFEEDLSCIDFATANASSLGFPMDGYPMDELFDDMPDQGSSGAMDDSSVVFRDLPGLINLPAAEEADAIGDAETALLRDLADDSNGTDRNSYGVMGDDQDRLLLPEIPRRIDMPDLFVSQAEEAGLGGGAALDSSSGAMDGEALALSDFEFPESVEEVLSCMDFSTVDMSFLDVPIDELLDDLPAD

LOC_Os05g26049.1	MAAAAAGADGLLPGLKLDPSDDELVGRCLLRRLQGQPLPLGGDI LEADPLSAPPPWNLLADHGRGDEAFFLAKKGNGKRQRSSVEGQ RMCVDGGRLRVPDDGRGGGGGLAFLPSIFPSPPPVPRCSTPLSPSS PPFKPSRVIVRWWAPPGGWCKLNFDGSVYDDGSRRASIGGVIRG CDGGVVLAFAEITEHWTVGVVEARAMIRGLRLALACFVERLVVE GDDLVLVQLIRGEETQTRIPAAMHEEILDLLRCFADVEVRHIYREG NSMAHTLCRQAYVHPGLWTDCATLPAAVWEKIDDDLRGVVHE RLCNKKKKSSA
LOC_Os05g26026.1	MAAAAAGADGLLPGLKLDPSDDELVGRCLLRRLQGQPLPLGGDI LEADPLSAPPPWNLLADHGRGDEAFFLAKKGNGKRQRSSVEGQ RMCVDGGRLRVPDDGRGGGGGLAFLPSIFPSPPPVPRCSTPLSPSS PPFKPSRVIVRWWAPPGGWCKLNFDGSVYDDGSRRASIGGVIRG CDGGVVLAFAEITEHWTVGVVEARAMIRGLRLALACFVERLVVE GDDLVLVQLIRGEETQTRIPAAMHEEILDLLRCFADVEVRHIYREG NSMAHTLCRQAYVHPGLWTDCATLPAAVWEKIDDDLRGVVHE RLCNKKKKSSA
LOC_Os05g27749.1	MAAAAAGADGLLPGPKLDPSDDELVGGYLLRRLQGQPLPLEAD PLSARPRNLAADHGRGDEAFFLAEAQAKNAKGKRQRSTVEGQS MCVDGGRLRVPDDGRGGGGLAFSHFLPLSPSIVPSPAPSPRCSTST PLSPPKLLADHGRGDEAAFFADAWAKNGKRQKQRSTVEGGGL WQGQGMLVDGERLRVADDGGGGSAFLPPSILPSLLPAPRCSTPLS PSSPPFRPSRVIVRWARPPPGWCKLNFDGSVFNDGSPRASIGGVIR DSDAGVVLAFAETTEHWTVGVVEARAMIRGLRFALACFIERLVV EGDDLVLVQLIRGEETQTRIPAAMQEEILNLLRCFAEVDVRHIYRE GNSVAHTLCRQAYVCPGIWSQRGGGMPAAVWDKVDDDRRGV VHERIRKNK
LOC_Os05g34310.1	MAMQLSLPVLPTGFRFHPTDEELVINYLQRRATGLSCPIPIIADVEI YNFNPWELPSMALFGEHEWYFFTLRDHRYPNSVRPSRSAASGFW KATGTDKPVQVANMQSTPVAMKKALVFYVGRPPMETKTTWIM HEYRLTNTGGSTASHPSLSSSTAHPSVKLDEWVLCKIFNKSPEPDN TAPPSNVVSRLQCSPPLPPPAAPPGNYPPLPVGATNDGGVFAGA GDMLFTIQEHQEGTPSMLPPIPNLEPPAATIGNSSLNGTAAAAAA ADGHGRLEEEDTSAYTFTDQEMEQMLMDLMDQDFFGNDQPQE MSRDDVDDVTAGAAGSGEEAAADQEEAAAAVAGDSHENDLV
LOC_Os05g34600.1	MPGFRFHPTEEELIEFYLRRKVEGRRFNVELITFLDLYRFDPWELPA MAVIGEKEWFFYVPRDRKYRNGDRPNRVTASGYWKATGADRMI RGENSRPIGLKKTLVFYSGKAPKGVRSSWIMNEYRLPPPAADADL FYKSEISLCRVYKRSGIDDGHGHHQRPAGNVQASSSSAAARPPEQ HSGNNTAAGLPACRHRPSPSSSSTTTAQQHTSFHQLLQGECSAA AAAPPPPPSLPASATTRNSNASQLLMPPPPPRPPCAAAYTSAAAA PTESAAVLAAASTYSLLAAAGSSSTHIDELSTLLAGHSHGGAYGN NHIVAGSHHHFPLPPSQLMPQLGTLPISPPLAAVSDKLWDWSSVP DTSTARDYDSSGFSDPK

LOC_Os05g34830.1	MSGGGEGAAAAERQELQLPPGFRFHPTDEELVMHYLCRRCAGLPIAVPIIAEVDLYKFDPWHLPRMALYGEKEWYFFSPRDRKYPNGSRPNRAAGSGYWKATGADKPVGTPRPVAIKKALVFYAGKAPKGDKTNWIMHEYRLADVDRSARKKNTLRLDDWVLCRIYNKKGGVEKPSGGGGGERSNMMSHGETASAGSPPEQKPAVLPPPPPPYAAAAPFSELAAFYDVRPSDSVPRAHGADSSCSEHVLTTSASSGGVVERPEVQSQPKIAEWERTFAGAAAPAGAVSTAGPILGQLDPAAAVAGGGDPLLQDILMYWGKPF
LOC_Os05g34830.3	MALYGEKEWYFFSPRDRKYPNGSRPNRAAGSGYWKATGADKPVGTPRPVAIKKALVFYAGKAPKGDKTNWIMHEYRLADVDRSARKKNTLRLDDWVLCRIYNKKGGVEKPSGGGGGERSNMMSHGETASAGSPPEQKPAVLPPPPPPYAAAAPFSELAAFYDVRPSDSVPRAHGADSSCSEHVLTTSASSGGVVERPEVQSQPKIAEWERTFAGAAAPAGAVSTAGPILGQLDPAAAVAGGGDPLLQDILMYWGKPF
LOC_Os05g34830.2	MALYGEKEWYFFSPRDRKYPNGSRPNRAAGSGYWKATGADKPVGTPRPVAIKKALVFYAGKAPKGDKTNWIMHEYRLADVDRSARKKNTLRLDDWVLCRIYNKKGGVEKPSGGGGGERSNMMSHGETASAGSPPEQKPAVLPPPPPPYAAAAPFSELAAFYDVRPSDSVPRAHGADSSCSEHVLTTSASSGGVVERPEVQSQPKIAEWERTFAGAAAPAGAVSTAGPILGQLDPAAAVAGGGDPLLQDILMYWGKPF
LOC_Os05g35170.4	MAQTCLPPGFRFHPTDVELVSYYLKRKIMGKKPLIQAISDVELYKFAPWDLPAQSCLQSRDLEWFFFCPRDKKYPNGSRTNRSTPNGYWKTSGKDRTIELNSRIVGSKKTLIFHEGKAPKGNRTDWVMYEYKMEDNQLVSAGFSKDDFVLCKIFKKSGLGPRIGEQYGAPFNEEEWEHADAEMFPLLPNVETSVFPLLPSSEVVNSTDDTRVQPSVAARAIEELPVQHLPHVCAGNGSTYQNITVTGESALMELPSQHSVESIGDEVVSVDNCSNVVNNADSPVIEGLVLEELSRFLTDSPHHGNPVGEVDRACSLSDLPVFLLCLGI
LOC_Os05g35170.2	MAQTCLPPGFRFHPTDVELVSYYLKRKIMGKKPLIQAISDVELYKFAPWDLPAQSCLQSRDLEWFFFCPRDKKYPNGSRTNRSTPNGYWKTSGKDRTIELNSRIVGSKKTLIFHEGKAPKGNRTDWVMYEYKMEDNQLVSAGFSKDDFVLCKIFKKSGLGPRIGEQYGAPFNEEEWEHADAEMFPLLPNVETSVFPLLPSSEVVNSTDDTRVQPSVAARAIEELPVQHLPHVCAGNGSTYQNITVTGESALMELPSQHSVESIGDEVVSVDNCSNVVNNADSPVIEGLVLEELSRFLTDSPHHGNPVGEHSGLPPMSEAEAHAFEVSTNDLYNEIAGLAELGVPNGDGFSPSNAGVTEQQPTYFGVPNSENYVNMDDIFAPDTRLSYAYPLPNNQFWHYPMDQFTYSTTLSAAFPSGDSRPTMRIVDDLPAAANNGGFASKPSMQFPLS

LOC_Os05g35170.1	MAQTCLPPGFRFHPTDVELVSYYLKRKIMGKKPLIQAISDVELYKFAPWDLPAQSCLQSRDLEWFFFCPRDKKYPNGSRTNRSTPNGYWKTSGKDRTIELNSRIVGSKKTLIFHEGKAPKGNRTDWVMYEYKMEDNQLVSAGFSKDDFVLCKIFKKSGLGPRIGEQYGAPFNEEEWEHADAEMFPLLPNVETSVFPLLPSSEVVNSTDDTRVQPSVAARAIEELPVQHLPHVCAGNGSTYQNITVTGESALMELPSQHSVESIGDEVVSVDNCSNVVNNADSPVIEGLVLEELSRFLTDSPHHGNPVGEHSGLPPMSEAEAHAFEVSTNDLYNEIAGLAELGVPNGDGFSPSNAGVTEQQPTYFGVPNSENYVNMDDIFAPDTRLSYAYPLPNNQFWHYPMDQFTYSTTLSAAFPSGDSRPTMRIVDDLPAAANNGGFASKPSMQFPLS
LOC_Os05g35170.5	MAQTCLPPGFRFHPTDVELVSYYLKRKIMGKKPLIQAISDVELYKFAPWDLPAQSCLQSRDLEWFFFCPRDKKYPNGSRTNRSTPNGYWKTSGKDRTIELNSRIVGSKKTLIFHEGKAPKGNRTDWVMYEYKMEDNQLVSAGFSKDDFVLCKIFKKSGLGPRIGEQYGAPFNEEEWEHADAEMFPLLPNVETSVFPLLPSSEVVNSTDDTRVQPSVAARAIEELPVQHLPHVCAGNGSTYQNITVTGESALMELPSQHSVESIGDEGLLNWVCQMVMDFLRPMLVLLNNSPHTLEFLTVKIM
LOC_Os05g35170.3	MAQTCLPPGFRFHPTDVELVSYYLKRKIMGKKPLIQAISDVELYKFAPWDLPAQSCLQSRDLEWFFFCPRDKKYPNGSRTNRSTPNGYWKTSGKDRTIELNSRIVGSKKTLIFHEGKAPKGNRTDWVMYEYKMEDNQLVSAGFSKDDFVLCKIFKKSGLGPRIGEQYGAPFNEEEWEHADAEMFPLLPNVETSVFPLLPSSEVVNSTDDTRVQPSVAARAIEELPVQHLPHVCAGNGSTYQNITVTGESALMELPSQHSVESIGDEHSGLPPMSEAEAHAFEVSTNDLYNEIAGLAELGVPNGDGFSPSNAGVTEQQPTYFGVPNSENYVNMDDIFAPDTRLSYAYPLPNNQFWHYPMDQFTYSTTLSAAFPSGDSRPTMRIVDDLPAAANNGGFASKPSMQFPLS
LOC_Os05g37080.1	MGELPPGYRFYPTEEELVCFYLRHKLDGGRRVPDIERVIPVADVCSLDPWQLPEAHQGAWTGDGEPWFYFCPRQEREARGGRPSRTTPSGYWKAAGTPGWVYSSDGRPIGTKKTMVFYRGRAPAGAKTKWKMNEYRAFEEDDDNAAAAAPAQNHYLQTRSDFSLCRLYTRSGCPRQFDRRPPSSSVAGGGGENRAAPSSTAAAFANEDAAESSGKSQKRKRSAPDDSLDSTSSSDDNGGCDGSMLQQQQQRQRGTDEELVECSMTDWADLLDWF

LOC_Os05g43950.1	MAGDPAAGGDGGRGSSGGKGSSPSSSRHQQFRNLAKTRVDDLQ EMFSGLQSARKESRSADAALLEEQVHHMLREWRAELNVPSPASS LQNSQSQGNNREASDPPSETLRLLQLAGAEEEDDATSKLVMPRSP MPMQSSHEGHNLSPVLQGGTMAGGAAELMVPRSPLQQMPSSH QSHGHGQDGGQNLQGEAVMGSTAATAAPHLGQGMQGDCGG MAGVTNAMFHDQLYYIDHELNIDDFLQDDDYKINLPGSNPDGP NTMQGIGQLEHQQYNLPLDLPPNSFVDANNSAQSSGDVFFHMS DLLTTMCPSPSQYLGPKCALWDCGRPVRGSDECQHYCNPYHAG LALNDDGLLGTRPVMRPRGIDLKDGPLFAALSAKVQGKNVGIPV CEGAATTKSPWNAPELFDLSLLEGESLREWLFFDTPRRAFDSGNR KQRSLPDYNGRGWHESRKQVMKDFGGLKRSYYMDPQPSSNYEW HLFEYETNDSDALALYRLEYKSSDTKRSVKSKLASSPLSEIQQQMV RLSADSPVESKRTARSRAKANQKDNNSNAYPALNTPVQVSASNA HQTMSVNTPDQVNVSNAYQTMPLNTPNQPGPSNAYHAASQMD QMTFLDGSVVYGPHLPYGYSTERSDFYWNPSDGT
LOC_Os05g43960.1	MAGLGKGESSGGGGGGGGEMGFRFKPREAEAVEYYLLPRLQGRP PVPNPAIVVENVYEFEPERLINEKCNGGVAGEGEEGWYFLSPRDR KYRNGKRPSRSTEDKAGRWKASTGKTEGKDPITECYGWVKFCVT SLVYFKGPVKTEKKTKWLMREFTIPHFENKLDKTAAAGGSSNQR QLDQYVLCRIYTSPKKGADDGEQAEVVRGGGGGGEDIDEWAEA CAVFDLGPETAEGSDNADAAAAEGDMRSAKQAGKRPVAAAAV AEQPSKRPWLPPSPSTPCDGGPSQAMGNRQVPMQGLSLMHNFP PPPTTFCGHAPFQQGFPVHNNRAQMRWPTMQHNCMPSPAHSF QPRPVQRRPVLVGQAPPQRRPVHHVGGHAPMHMHQAQWTPV HIAQAPMQQLPFDDWVFDPFDDPPPPMQQLPVMMNNYQPQAP MQLPPMMNNDQPAMVHGGELQAPMQLLPATTHGGEVQAPMP LNVYEEEQRPSQEDGGQCTNAEG
LOC_Os05g48850.1	MTWCNSFSDVRTAVDSSLSPAAAVAAAAGKKAAASLAVLVKM CPSCGHRARYEQETTTIQDLPGLPAGVKFDPTDQELLEHLEGKAR PDSRKLHPLVDEFIPTIEGENGICYTHPERLPGVSKDGLVRHFFHR PSKAYTTGTRKRRKVHSDEVDGGETRWHKTGKTRPVMANGRPR GYKKILVLYTNYGKQRKPEKTNWVMHQYHLGSDEEERDGELVV SKVFFQTQPRQCGSTAAAAAAKEASAAVAAAVVNSNYSIVHGH QGGGGGSFLKEANVVHEFYDPAATMGYRPPAPAAHFAPNFAVH AARNSFGGP
LOC_Os06g01230.1	MKQGKVEMTPAVAAAAVLPVGFRFRPTDEELVRHYLKGKIAGRS HPDLLLIPDVDLSTCEPWDLPAMSVIKSDDPEWFFFAPRDRKYPG GHRSNRSTAAGYWKATGKDRLIRSRPAGPLIGIKKTLVFHRGRAP RGLRTAWIMHEYRTTEPHFQSGKNGSFVLYRLFNKHEQDDTHTP ASNLDQQLSTSSQGNPQNGTPAVQPALASIMKDHQTLPSSGFSQL TEIQDASTSVHDKEQTVAHDDAFLDVLSQLPDLEPEQRYNGFPNI TSPIRPYSDHPFVGNLGEQDLSAHFGSTLSEQDLQSLLFSPNYTKM DKHPTGNVESNPTASSNNPNNNTLLMDSWRKNDSYQMLLIQTT QMLLAVLLQSMHHKQKQVMLILKQEPRVAAWSTVE

LOC_Os06g01230.2	MKQGKVEMTPAVAAAAVLPVGFRFRPTDEELVRHYLKGKIAGRSHPDLLLIPDVDLSTCEPWDLPAMSVIKSDDPEWFFFAPRDRKYPGGHRSNRSTAAGYWKATGKDRLIRSRPAGPLIGIKKTLVFHRGRAPRGLRTAWIMHEYRTTEPHFQSGKNGSFVLYRLFNKHEQDDTHTPASNLDQQLSTSSQGNPQNGTPAVQPALASIMKDHQTLPSSGFSQLTEIQDASTSVHDKEQTVAHDDAFLDVLSQLPDLEPEQRYNGFPNITSPIRPYSDHPFVGNLGEQDLSAHFGSTLSEQDLQSLLFSPNYTKMDKHPTGNVESNPTASSNNPNNNTLLMDSWRKNDSYQMLLIQVRIF
LOC_Os06g01480.1	MDGSSMSSSSTQQQAQVPPGFRFHPTDEELVDYYLRKKVAARRIDLNVIKDVDLYKIEPWDLQERCRINGGSAAEEQNEWYFFSHKDKKYPTGTRTNRATAAGFWKATGRDKPIYATKQHSLLVGMRKTLVYYRGRAPNGHKSDWIMHEYRLETTETAPPQEEGWVVCRVFKKRLPTTRRDSDHDAPCGSWYVDEDAPGAFMSPMMITRSSILRPHQHHAGITLQEQHLHTTYKHRDLTTKIQQLQVPAAGHHLLNTMPHDLESSTSSFHSLLVSPDHHQINMHHAQADPFFDDMHAVDQATTTDWRVLDKFVASQLSNDATNKPADHYTDEGDILQVSDKQQEVAAADYASTSTSSSQIDPWK
LOC_Os06g04090.1	MSISVNGQSVVPPGFRFHPTEEELLTYYLKKKVASERIDLDVIRDVDLNKLEPWDIQERCRIGSGPQNDWYFFSHKDKKYPTGTRTNRATAAGFWKATGRDKAIYSSSNRIGMRKTLVFYKGRAPHGQKSDWIMHEYRLDDPSSASASVSVNLPSYYSSSSSSSPMHGVAGDQGAQEEGWVICRVFKKKNLVHHGGGAAAASHHAAAKLAAAAMEGSPSNCSTVTVSDHVKAQMLHSSASDDALDHILQYMGRSGCKQETKPAAMSASSAAAAAALEQHLSTPQYGKFMKLPPLEHVAGGVGLLAAAGGGGEYCSAADASGIADWDTLDRLAASYELNGALSDVASGKNMAGFFDVVDQPAGAAAFSSGDGDLWSLARSVSSSLHADLTTMNNV
LOC_Os06g23650.1	MERCSVLGLGGGGGGGGRLDGELPPGFRFHPTDEELITYYLLRKVVDGSFNGRAIAEIDLNKCEPWELPEKAKMGEKEWYFYSLRDRKYPTGLRTNRATGAGYWKATGKDREIRSARTGALVGMKKTLVFYRGRAPKGQKTQWVMHEYRLDGTYAYHFLSSSTRDEWVIARIFTKPGVFPVVRKGRLGISGGGGDTSCFSDSTSASVGGGGGTSASSALRAPLAEASLFAAAAAPAVDGADSSNYGGGGGAGSATATANLVTGLELVPCFSTTAHMDASFGTGQYNPAPLAVEPPPPPPAFFPSLRSLQENLQLPLFLSGGMQAGVSSQPLSGGGAFHWQSGMDVKVEGAVGRAPPQMAVGPGQLDGAFAWGF

LOC_Os06g33940.1	MHPSGGALSVPPGFRFHPTDEELLYYYLRKKVAYEAIDLDVIREIDLNKLEPWDLKDRCRIGTGPQNEWYFFSHKDKKYPTGTRTNRATTAGFWKATGRDKAIFLANACRIGMRKTLVFYVGRAPHGKKTDWIMHEYRLDQDNVDVQEDGWVVCRVFMKKSYQRGLNPADMAAVDDDDLLHHHHHPFPPAQLHGGAADHKHDGAGGHHHHHLMQPHHHYDDFPSFDPSMQLPQLMSADQPPPPPPSLLPGVPPSAAAALSSLDVECPQNLMKLTSAAAGGGATGLLHAGGDHRFATAATDWSILDKLLASHQNLDQLFQGRVIAGASSPAAMAAPSHHQHLMDQLAGGGGGTASSLQRLPLQYLGCEAADLLRFSK
LOC_Os06g36480.1	MDGVVAEDQAGGSGSGHRRLIGSRIEEHRKYMSEESCCPRCGHKIDRKLDWVGLPAGVKFDPTDQELIEHLEAKVRPGGEAAAHPLIDEFIPTIEGEDGICYTHPEKLPGVSKDGLSRHFFHRPSKAYTTGTRKRRKIQPPAAAASSGGGGGNASSSSASAAAAVARHGHQQQQQQRSETRWHKTGKTRAVVGGGRQRGCKKILVLYTNFGKHRKPEKTNWVMHQYHLGEAEEERDGELVVSKIFYQTQPRQCAAADAAATASASAVDRRTTSLRDRAAAAAAAAAAAAPMASANVSVAAFHGGAAGIDEFSFAQFRSSFEEAGMGASSSDHQSAMVDQRRRQQQHDDDEHDHRRGGGGHHYVGQQQSVAATFHVVSSPADPIARLMSPPPAHQGTVMLRQPEPPYIYHHQEDERPHQPRKFDGRSTSGSGLEEVIMGCTSRRSKGGETSGGKDGTEWQYPSFWPSDSQDHHGDKDDIEAKQKGETEKEKGEEKQSFSRYTFAKKYSMNKETLKISSILHSGILSESKLPENFPVCVVEGEYDEHGNAWMNLILPFSDSSKLRLM
LOC_Os06g46270.1	MSGMNSLSMVEARLPPGFRFHPRDDELVLDYLERKLLDGGVGGAAAAAAAVTIYGCPVMVDVDLNKCEPWDLPEIACVGGKEWYFYSLRDRKYATGQRTNRATESGYWKATGKDRPISRKGLLVGMRKTLVFYKGRAPKGKKTEWVMHEFRKEGQGDPMKLPLKEDWVLCRVFYKSRTTIAKLPTEGSYNNIDSVATTSLPPLTDNYIAFDQPGSMQNLEGYEQVPCFSNNPSQQPSSSMNVPLTSAMVDQEQNNMGRAIKDVLSQFTKFEGNVKREALQSNFSQDGFDYLAESGFTQMWNSLS
LOC_Os06g51070.1	MEQQRSRSTAAGGEVEVEQLPGFRFHPTEEELLEFYLKQVVQGKKLKFDIIPTVHLYRHDPRELPGLARIGEREWYFFVPRDRKQATGGGGGGRPSRTTERGFWKATGSDRAIRCAADPKRLIGLKKTLVYYEGRAPRGTKTDWVMNEYRLPDAAAIPDTMQLQMQHDDMVLCKVYRKAVSLKELEQRVAMEELARSTTSSGTHNTGSPLQQDSSSISISSSSDAMKKEVVGVDEASAAAHELVRPATLSLPQLEVARPQSGLEWMQEPFLTQLRSPWMETWSPYYASVLNF
LOC_Os07g04560.1	MKRGCEDELGAGDVILRGVEEVEEEDDDDLVLPGFRFHPTDEELVTFYLRRKIAEKRLSIEIIKEMDIYKHDPSDFLKTSTVGSEKEWYFFCLRGRKYRNSIRPNRVTGSGFWKATGIDRPICSAAGGGGGDCIGLKKSLVYYRGSAGKGTKTDWMMHEFRLPPPPADDLAAGRSSPPPSLQEAEVWTICRIFQRNITHKKQPQPQLAVAAAAVPAPVPDATSSITGSLESDSAGDDVVEYMNTLQPPPASNVNGGYSNQRYFQEQWNSSSNDNTTVFHQHAAAAPPPEPSPATAMAGFGHDQSVLSSPAPSDFYYKDGCNDDIYRMVMELADPSLFYDHIYA

LOC_Os07g09740.1	MASAAGDGLPPGLKFEPKDDELVARFLLARIQGKPLPLHGVILDA<br>DPLCAPPWRLLADHGRGDEAFFFADARAKNGKGSRQKRTVEGG<br>GYWQGQRMCVDGERLVVPDGGLEIAWRKYVLSYFADGEKGSSG<br>WVMHEYAITTPADLASSTMRLYRIRFSGHGKKRKREPESQSAHH<br>DDGRARCAPQIAMPETALLEDSAPPPQPVLPPAAVVNSVSDGAV<br>PPPAPVVNCDSDVTDEDELQSFVPEFSARNLFVSLPQGSHEAEAD<br>VVGGALPAQSMSSFADVGGPENMDDQSCSGVVFANLSDLIVLPP<br>VEASGAAPAPSWASSLDNQNDEAPVFFEFPESMDDIIGCFDFATM<br>DDPSCTSAISEEPFLPPAAMVNHDDGYASDNADQGCSGAVPLPS<br>AVVDLPNETDGADQSCSGVVDDSSMVFANIHPLDSPAEGGHEAE<br>AGAGGGRAAPAPSWVSSLDNQNDEAPMFFELPESLDDMVSCFD<br>FAAMDGQSCTSAVSETALIEELVLPPAAMVNHHDDSVSDIADHG<br>CSGAAPPPSAVVDLPDDSDGADQSCSGMVDDSLPGYYEAELKVP<br>LEYAARNPVDSPSKGGHDHEAEVDASGGAGSMMSSPDKEKEHS<br>SSGVMDVEATGFGVPDSMDGLSCIDFAETMDDLSCIDFTIDDELF<br>DLWS
LOC_Os07g09830.1	MAAAAGDGLPPGLRFEPKDDELVARFLLARIQGKPLPLHGVILD<br>ADPLCAPPWRLLADHGRGDDAFFFAEARAKNGKGSRQKRTVEG<br>GGYWQGQRMCVDGERLVVPDGGGGGGVEIAWRKYVLSYFADG<br>EKGSSGWVMHEYAITAPADLASSTTRLYRIRFSGHGKKRKREPES<br>QSDNHQIAVAETAMLEDSAPPPQPVHPPAAMVNCVSDDTDQGF<br>SGAVPPPAPVVHHTNDSDVTDRYSSLVFSDQPGSIYEDELQSFVPE<br>FPARNLFVSLPEGSHEAEPAQSVSSLADVGGPENMDDQSCSGVVF<br>ANLPELIVLPSAPSLDNQNDEAPVFFEFPESMDDIVGCFDFAAMD<br>NQSCTSAIPEEPFLPPAAMVNHDDGYASNNADQGCSGAFPLPAA<br>VVDLPNETDGADQSCSGVGDNSTLLFSDVTGSIDEDELQSFVPEF<br>AASNLFVSLPQGSCEADAEADSGGGVAPAQFAEFGGSESMDDSS<br>MMYPLNFPAEAGGGGGRAAPASSWVSSQHNQNDEAPMFFELPE<br>SLDDMVGCFDFAAMDGQSCTSAVSETALIEELVLPPAAMVNHH<br>DDSVSDIADHGCSGSVPPPSAVVDLPNDSDQSCSGYYEAELKVPL<br>EYAARNPVDSPAKGGNEAEVDASNGAGSMMSSPDKQKEHSSSG<br>VMDVEAIGFGVPDSMDSLSCIDFAETMDDLSCIDFTIDDELFDLWS

LOC_Os07g09860.1	MAAAAGDGLPPGLRFEPKDDELVARFLLARIQGKPLPLHGVILD ADPLCAPPWRLLADHGRGDDAFFFAEARAKNGKGSRQKRTVEG GGYWQGQRMCVDGERLVVPDGGGGGGVEIAWRKYVLSYFADG EKGSSGWVMHEYAITSPADLASSAMRLYRIRFSGHGKKRKREPDS QSAHDEHGRARCAPQIAMPETALLEDSAPPPQPVHPPAAVVDC VCDVTDQGSSLVFPDQPGSIYEDELQSFVPEFAARNLFVSLPEGSR DVVAEAALIEDLALSPQPVPPPAEVVNQADDSDGADQGCSSVFA ALPDLIVLPPEEACGSGGAAPAPSWASSLDNQNDDAPAFFEFPES MDDMVGCFDFASMDNQSCTSAVSEIAVLEEPFLPPPTMVNHDN NSVSDGADQSCFGVGDNSTLVFSDLTGSIDEDELQSFVPEFVSLPQ GSCEADAEADSGGGVAPAQFAEFGGPESMDDPLNFPAEASGGG DRAAPASSWVSSQDNQNDEAPMFFELPESLDDMVGCFDFAAMD GQSCTSAVSETALIEELVLPPAAMVNHHDDSVSDIADHGCSGAV PPPNSEVVDLPNDSVGADQSCSGMVDDSLSGYYEAELKDASGGA GSMMSSPDKQKEHSSSGVMDVEATGFGVPDSMDGLSCIDFAETM DDLSCIDFTIDDELFDLWS
LOC_Os07g12340.1	MAAAKRRVRDAEADLNLPPGFRFHPTDEELVAHYLCPRAAGRA APVPIIAELDLYRHDPWDLPHRALFGRREWYFFTPRDRKYPNGSR PNRAAASGYWKATGADKPVLHNGRTAGIKKALVFYHGKPPRGV KTEWIMHEYRLAKKGGAAAAAGAGALRLDDWVLCRLYNKKNE WEKMQSRKEEEEAMAAAQSWGETRTPESEVVDSDAFPEMDYSL PAASFDDALLPKEEARDDDWLMGMSLDDLQGLGSLLQADDLSM LAPPPAAKTEPLGAPFF
LOC_Os07g13920.1	MAAAGADGLPPGLRFDPSDDELVGRYLLRRLQGQPLPLDGVVLD ADPLSAQPWRLLADHGRGGDEAFFLAEAHAKNAKGKRQKRTV EGGGFWQGQRMCVDGKKLLVPGGDDGGGGGEVLEIAWRKYVL SFFAEGERGSSGWVMHEYSVTAPADLASSPLRLYRIRFSGYGKKR KREPEDDGRAHGAPRRAEAETALFDLEVGPPPPPLLVPPPAAAA ADHGTDQSSSGVTDMVFRDLPDLIADAGAALPDQNQQDWSEV ADQSSFCVMGDDSSLLLPDLPGMIDDNEHQQFVRECDMPHLFVP QAEEAIAGGGAASAPSADNQNCEFNDGEDMALSDFEFPESIDEV LSYIDFSTSDTSCRDFTMDELFDLPVD

LOC_Os07g17180.1	MAAGGGGADGLPPGLRFDPTDGELVSRFLLRRLQGKPLPLNGVI<br>LEADPLSVPPWKLLAEHGRGDEGFFFAEARAKNGKGSRQKRTVE<br>GSGLWQGQRVCADGEKLLVPDGGGVEVEIVWRKYLLSFFAEGER<br>GSSGWVMHEYAVTSPAELAASPIRLYRVRFSGHGKKRKREPQSGE<br>DGVGRARAAPQSAGTETALLEERVMPPQPAPQSVGTEDALVEER<br>IPPPQPVPIPPIAGTEDALDVGTEDVRGRAAPQSAGTESALLEECV<br>LPPQTAPQITGTGVALLDEVVPPPQTVSISPPAALVDAVDDADCA<br>NQGCSGVMDDSTMVFSHLPDMITLPAEEGDAAGGAALASMDYS<br>WADFEFPEINMDELPNCIDFTTTDPSCLDIELSMGDLHEPQSTGIE<br>SDLLEEFVPQPQPVLVPPLAALVEVADSSEGPDQGCSVVMHDSSA<br>VFTHLSDPIVLPEEEEADRPDAPAGTMSLDYQNYSLSDFEFPEYPL<br>LDVAGDADGADQCSSNVMDDSSMVFSHLEDLITLPAEEAEADA<br>CSAAPAPSLDNQKYSSQGIIDSEAPALSDFEFPETIDEVLNSINFTM<br>ADPSCLDMEFSMDDLLDFDPPAD
LOC_Os07g27330.1	MAAAADGLPPGVRFDPADDELVSRYLLRRLRKQPIPLHGVIHEA<br>DPLGAPPWMLLAAHGRGGDEAFFFAEARAKNVRGKRQKRTVE<br>GGGFWQGQRVCIDGERLRVPGDGGGGEVGGELEIEWRKYMLSFF<br>AEGERGSSGWVMHEYAITAPADLASSPIRLYRVRFSGHGKKRKRE<br>PERLGARVHDDDVDGGERAAPRRAVAETALFVQQSSAVDCAES<br>ADQSFSGVIEPVFHDLPDMMPEQADAGDTAETTAAVVNLTDAM<br>TEQPVLPLAADGDDQSSYGVIDPAFRDLADLMVLPPVLAQQEPP<br>LAPVAMVDLPPGNADCADHQSCSGVIDPAFRDLPDMTVLPPEQ<br>ADTGGGAETTTAMVSLTDKLKYSSSMDGEAAPAWCDFDFPESTD<br>EVLSYMNFTAGAHDNNDGSVGRAAPWRPVSEIAMFEQPSAVDL<br>PPGDADCTESADQSFSGVIEPVFHDLPDMIREQADAGDTAETTA<br>AVVNQNYSMALCDFDSGIDFTAGAHDSGMERATPWTPMSEAAL<br>FEQQGPPLAPAAVVDLPPGNADCADHQSSYGDMIVLPSEQAGA<br>GGGAETTEALFDQPVPPLAADCANQGSYGVIDPVFRDLADLIVLP<br>PEQADAMDGEAAPAWCDFDFPENIDEALSYVDFTAGAHADND<br>GGVSETAMFEQPGSPPQHDPLLMDADGADQSSSSGALIDTVFGD<br>HAEPIVLPLEQADTGGGAAAAVKLMDKQKYSSSSMDGEEAPAW<br>CDSDFPESIDEVLSYVDFSTDGASCDFSMDELFDLAD

LOC_Os07g27340.1	MAAAADGLPPGVRFDPADDELVSRYLLRRLRKQPIPLHGVIHEA DPLGAPPWMLLADHGRGGDEAFFFAEARAKNVKGKRQKRTVE GGGFWQGQRVCVDGERLSVPGGDGGGEVGGGLEIEWRKYMLSF FAEGERGSSGWVMHEYAITAPDDLASWPIRLYRVRFSGHGKKRK REPERLGARVHDDDVDGGQRAAPRRAVTETALFVQPSAVDCAE SAGQSFSGAIEPVFHDLPDMMPEQADAGDTTETTAAVVNLTDA MSEQPVLPLAADGDDQSSYGVIDPAFRDLADLIVLPPEPDDGGM ERATPCTPMSETALFEQQGPPGNADCADHQSSYGVIDPAFCEQA DAGEAETTVSAAVVNQNYSMALCDFNFPEVLSYVDFTAGMEPS WQQRWPPMSESAPFEQQEPPLAPVAMVDLPPGNADCADHQSCS GVIDPAFRDLPDMTVLPPEQADTGGGAETTTAMVSLTDKLKYSSS MDGEAAQAWCDFDFPESTDEALSYIDFTAGAHTDNDGGVSETA MFEQLGSPPQHDPLPMDADGADQSSSGPLIDTVFRDHAEPIVLPL EQADTGGGAAAAVNLMDKQKYSSSMDGEAVPAWCDSDFPESID EVLSYIDVSTDDTSCIDFSMDDLFDLAD
LOC_Os07g31410.1	MAPLADPAAEGFRIPFLPSDSDLLDCLLRPKIASGRVDPRFAPLVH DVADAFALPPAQLAAAHAPAPGAGGAEAWYFFSVRPRARARA RAGSKRAASRAVGGGGGKRWCSMGAKKAVEGGGYCQRFRYKE RTAAGVVAPRWMMVEYGVAQEHDGEGVAQEHGGEGVAELVL CKIFRSPEPSRRSESGSPSSSSSASASPSCSGGRKRKAAE
LOC_Os07g37920.1	MESPDSSSGSAPPRVLRRQQQQPGSAPELPPGFRFHPTDEELVVH YLKKKAASVPLPVTIIAEVDLYKFDPWDLPEKANFGEQEWYFFSP RDRKYPNGARPNRAATSGYWKATGTDKPIMSSGSTREKVGVKK ALVFYRGKPPKGVKTNWIMHEYRLTDTSSSAAAVATTRRPPPPIT GGSKGAVSLRLDDWVLCRIYKKTNKAGAGQRSMECEDSVEDAV AAYAPSSQQHATAAAGMAGSDGAGGVAAAHGGDYSSLLHHDS HEDTFLVNGLLTAEDAAGLSTGASSLSQLAAAARAAATPCDATK QLLAPSPTPFNWFEAFLPRAKEFPSGLSRSSRDIGDMSLSSTVDRSL SEAGAVAIDTGDAANGANTMPAFINPLGVQGATYQQHQAIMG ASLPSESAAAAAACNFQHPFQLSRVNWDS
LOC_Os07g48450.1	MEMTMSSAATSLPPGFRFHPTDEELILHYLRSRATAGQCPVPIIAD VDIYKFDPWDLPSKAVYGESEWYFFSPRDRKYPNGIRPNRAAGSG YWKATGTDKPIHDSATGESVGVKKALVFYRGRPPKGTKTSWIMH EYRLAADPLAAAANTYKPSSSSRFRNVSMRLDDWVLCRIYKKSG QASPMMPPLAADYDHDEPSGVLDDAYSFYAPPMISTTLIPKLPKIP SISELFDEHALAQIFDAAADPPADHHQHALAVHPSLNQLLGVGD NFLAECYPSTASTATVAGGKRKASPAGDYAGGGHTPAKRLNGSC FDVAPQSVVGGLQATPSSVLAGLNHQMLPPQLF

LOC_Os07g48550.1	MSESEVSVINQLEEEETRLELPPGFRFHPTDEEVVTHYLTRKAQDRSFSCVVIADVNLNNCEPWDLPSKAKMGEKEWFFFCHKDRKYPTGMRTNRATASGYWKATGKDKEIFRGRGLLVGMKKTLVFYMGRAPRGEKTPWVMHEYRLDGKLPPNLPRSAKEEWAVCRVFNKDLAAKIAQMPPPPFPRNDSFDLDLDDFLHLDADLPPLIDDPFASTSTLKTEPPPPANLMHNHYGYFSLPASATNYNHSSGAMADQAIRRFCKAEASTACFSGADADVDPVVDELLSFPDSITDYSYIWKA
LOC_Os08g01330.1	MDRHEEEAGESPCVPPGFRFHPTEEELVGYYLARKVASQKIDLDIIQELDLYRIEPWDLQERCKYGGHGGDEQTEWYFFSYKDRKYPSGTRTNRATAAGFWKATGRDKPVLSSPSTRVIGMRKTLVFYKGRAPNGRKTDWIIHEYRLQSNEHAPTQEEGWVVCRAFQKPMPNQQQHRLSYGCIPGSYGAGAYAAVPDNYSLLLHHDNPSFAGRPLMSAAASALFANNNNNSVVDHSNILSSESKLHFSDMMPPLESPTIVDGEGYVSQASSCVDVDQQAGIVDWNLLTSLLPPPAHQLFHHLPSASSSKNSNNISSSGFIDDRD
LOC_Os08g02160.1	MRGSDHHQDVVAAPRGGGGGGDDGQAHDMVMPGFRFHPTEEELIEFYLRRKVEGKRFNIELIAFVDLYRYDPWDLPALASIGDKEWFFYVPRDRKYRNGDRPNRVTPSGYWKATGADRMVKVEGDRPIGLKKTLVFYVGKAPKGLRSSWIMNEYRLPHGDADRYQKEISLCRVYKRPGIEDNFHLTGTTTKSSGSKAAAAMGKKHAAANRTSSTAAAAAPRLAPMFDGGGGGQARATASAAKKQAATAAAAKRRHPRRHRRPWSLQRRRHSTSSRVCSRVCHRWYGAPLTHGSPAGAGTGDISHPTTCAGSGSGGEVGEQGGGVPVQPTPLPTL
LOC_Os08g02300.1	MSISVNGQSCVPPGFRFHPTEEELLNYYLRKKVASEQIDLDVIRDVDLNKLEPWDIQERCKIGSGPQNDWYFFSHKDKKYPTGTRTNRATAAGFWKATGRDKAIYNAVHRIGMRKTLVFYKGRAPHGQKSDWIMHEYRLDDPATDTAAATPTVTSAAAAAAAMAAAADGGQEDGWVVCRVFKKKHHHKEAGGGGGKHGGDGSAGAKAAHAYSSSDDALDQILQYMGRSCKQEHELPSPQASGGGGAGAGSRPASRYLRPIDTVLGGHGFMKLPPLESPSAATALSSTPSTGGDAASSAAAAAADHLLLHHHHRTDWAMMDRLVASHLNGANSDAPDDQLCFDAADDDGLAYYSAAATRLLGGANAGTDDDLWSFARSAAPPPPPPPPSSATPERLSHVAL
LOC_Os08g05300.1	MADDVAPALLVDALLRRQHGHPLPPQLPFAIFDANVFASDPSTLYNEYWSYAADDGSIYLFSPGPSTEGQWRTATAARSITTADGTYIGRRTTWVIFDRVNGGWAMEEFCTYHNDGGGGGVAEDVRLYRIYRRIPSLQPLPPLVQRRRQHQVGLEGQFSQMCSLR

LOC_Os08g06140.2	MTVMELKKLPLGFRFHPTDEELVRHYLKGKITGQIRSEADVIPEID VCKCEPWDLPDKSLIRSDDPEWFFFAPKDRKYPNGSRSNRATEAG YWKATGKDRVIRSKGDKKKQQVIGMKKTLVFHRGRAPKGERTG WIMHEYRTTEPEFESGEQGGYVLYRLFRKQEEKIERPSPDEVDRSG YSPTPSRSTPDNMEPIEDGNTPLNRESPESALHESPIDLPALTEAQA APITRWLADRTDNATTNEVNISHMPHHGLDGGAKASPSAGAFP QLIGSQQNIHDNNELATVSAPMLPHEDFNNFPLGAIGNFDGNM NPRDPVEEFLNQTIADPDEHSSTTSKAQYDSDTGIIPTEFENHGVM QGEFMDDLSGLENLDFWPDDRNPQLSALYEDTPLLPYDSTDQDV LSMDSGAESLQDLFNSMDDSNARNNVWGNEPFLQGTGFPMSWP LQPNSAFPNQGTANRRLMLQLSESLSPDFDVSMTRDECEDEEPGI VVTSKYVNEAPEESTAEKDMPSDGDDAEPTGITILRRRHAPTASSF SDGDDAESTGITILRQHQAPNASLLSDGDDAESTGITILRRRQAPT ASSASSFTQQGAAVQRVRLQSNLDAAPCSSVDGSSSCIINEGESER TMEKPEIEENAGSTLAEGGTCHEDDQKEHDASAANAKSVLRLRK TAEGSDKENKQEEEEGVLASHVRAPGNKRGFPSYIIWLVLSVALV LLISLGIYGWV
LOC_Os08g06140.3	MTVMELKKLPLGFRFHPTDEELVRHYLKGKITGQIRSEADVIPEID VCKCEPWDLPDKSLIRSDDPEWFFFAPKDRKYPNGSRSNRATEAG YWKATGKDRVIRSKGDKKKQQVIGMKKTLVFHRGRAPKGERTG WIMHEYRTTEPEFESGEQGGYVLYRLFRKQEEKIERPSPDEVDRSG YSPTPSRSTPDNMEPIEDGNTPLNRESPESALHESPIDLPALTEAQA APITRWLADRTDNATTNEVNISHMPHHGLDGGAKASPSAGAFP QLIGSQQNIHDNNELATVSAPMLPHEDFNNFPLGAIGNFDGNM NPRDPVEEFLNQTIADPDEHSSTTSKAQYDSDTGIIPTEFENHGVM QGEFMDDLSGLENLDFWPDDRNPQLSALYEDTPLLPYDSTDQDV LSMDSGAESLQDLFNSMDDSNARNNVWGNEPFLQGTGFPMSWP LQPNSAFPNQGTANRRLMLQLSESLSPDFDVSMTRDECEDEEPGI VVTSKYVNEAPEESTAEKDMPSDGDDAEPTGITILRRRHAPTASSF SDGDDAESTGITILRQHQAPNASLLSDGDDAESTGITILRRRQAPT ASSASSFTQQGAAVQRVRLQSNLDAAPCSSVDGSSSCIINEGESER TMEKPEIEENAGSTLAEGGTCHEDDQKEHANAKSVLRLRKTAEG SDKENKQEEEEGVLASHVRAPGNKRGFPSYIIWLVLSVALVLLISL GIYGWV

LOC_Os08g06140.1	MTVMELKKLPLGFRFHPTDEELVRHYLKGKITGQIRSEADVIPEIDVCKCEPWDLPDKSLIRSDDPEWFFFAPKDRKYPNGSRSNRATEAGYWKATGKDRVIRSKGDKKKQQVIGMKKTLVFHRGRAPKGERTGWIMHEYRTTEPEFESGEQGGYVLYRLFRKQEEKIERPSPDEVDRSGYSPTPSRSTPDNMEPIEDGNTPLNRESPESALHESPIDLPALTEAQAAPITRWLADRTDNATTNEVNISHMPHHGLDGGAKQASPSAGAFPQLIGSQQNIHDNNELATVSAPMLPHEDFNNFPLGAIGNFDGNMNPRDPVEEFLNQTIADPDEHSSTTSKAQYDSDTGIIPTEFENHGVMQGEFMDDLSGLENLDFWPDDRNPQLSALYEDTPLLPYDSTDQDVLSMDSGAESLQDLFNSMDDSNARNNVWGNEPFLQGTGFPMSWPLQPNSAFPNQGTANRRLMLQLSESLSPDFDVSMTRDECEDEEPGIVVTSKYVNEAPEESTAEKDMPSDGDDAEPTGITILRRRHAPTASSFSDGDDAESTGITILRQHQAPNASLLSDGDDAESTGITILRRRQAPTASSASSFTQQGAAVQRVRLQSNLDAAPCSSVDGSSSCIINEGESERTMEKPEIEENAGSTLAEGGTCHEDDQKEHDASAANAKSVLRLRKTAEGSDKENKQEEEEGVLASHVRAPGNKRGFPSYIIWLVLSVALVLLISLGIYGWV
LOC_Os08g10080.1	MSFIGMVEARMPPGFRFHPRDDELVLDYLLHKLAAGGRGGGVYGGGGGVAIVDVDLNKCEPWDLPDAACVGGKEWYFFSLRDRKYATGHRTNRATRSGYWKATGKDRSITRRSSISSGEPSSSAAAAAVGMRKTLVFYRGRAPKGRKTEWVMHEFRLEPQPLHLKEDWVLCRVFYKTRQTIPSPSSEEAVTLPNELDLPATPSLPPLIDAYIAFDSAPTTTPSMVGSYEQVSCFSGLPALPMKGSISFGDLLAMDTSAEKKAIRVLHNSNTAKLELSPDWGQESGLSQMWNPQ
LOC_Os08g23880.1	MAAAKMAGLTPGFKFEPSDEQLVQFFLLPYLRELPVPLGGLVIRDDPRSVPPWKLFARNGRGDEEDAYFLAPADGEGRQARTCDGGRGRWITQRLERTGNLRLAGGGGSGEAVVFEKHRLNYHAGEGRCGSTGWVMHEYAVVKPAALGARHRACHIAFTGHGQKRKRVPDGYVDVEDDGSKASTNAAAAVPPSSTAAMSACPSNVTYNQGCHISPEQSIEQHFPAEHNNIQIQQQAYYQSQDHEQCQYSDEEKYLLQQIKQEQYYYNQQNCFLPGQGNQELYYNDEQQQIFSLPEHQCSQEQYCHHDDQQDCVLPEQHSQELHGYNNEEQGYLLPPEPIDQEEQALFVGGEPQHEQQPLTSTPRQALLDYDDGKLLPPVGVNGAIAIPPQDAAVASNDDDGGQATEAPAAKMTAEEKKWFMEELLTEGCWSGPLLFDQPYYGSALKN
LOC_Os08g33670.1	MEEEQRLPAGFRFFPTDEELVTYYLARKAMDATFTSAAIRDVDLYTSDPWHLPCDSSAASTGGGGGGECYFFCRRSSKYPSGARVRRATAGGYWKSTGKDKGVYAAGGGGGLVGTKKTLVFYEGRAPRGEKTSWVMHEYSRAPSTNFIRGAQARTHNLLDIIYSEWVICRVFKKQPPIEHWLEMEEVETTTTTTTTVQEHTPNRRRLPPAEAAAAPPPPSGQPWQHTSRRSGDGRAAIDGGNREEEEDEHGLAREESSSPVVISSPSRCTSSPSSRLLNHEHLGASSSDDLPELMEFGDIYGGIAAGGPTDQQASSSNSNSICNFLDEPYYCWNF

LOC_Os08g33910.1	MEERNDVNMDKSDEILLPGFRFHPTDEELVSFYLKRKIQQKPISIEL IRQLDIYKFDPWDLPKLASTGEKEWYFYCPRDRKYRNSVRPNRVT TAGFWKATGTDRPIYSTEGTKCIGLKKSLVFYKGRAARGIKTDW MMHEFRLPTLTDPSLPKKPIDKNIPLNDSWTICRIFKKTSSMAQRA LCQTWGAQLPGTIDPDIFSTLQSVQASQFALESSSCSLQAAATAA HQITSKYALQGNNNNQQQQQHKPSNPLDGSSCKVINFNCSQS AEVQNSQIILPFEAHTSQKTATPLLFDTQFGQPDQISRFVVDSSVN ANGGGISNKSQDPSARKPGSGFSMNSDWDGVARINFPFDLGADS SEDWRSSIPWESFLSPTTVHAEMPN
LOC_Os08g40030.1	MGDALWEMLGEEMAAAAAAAGEHGLPPGFRFHPTDEELVTFYL AAKVFNGACCGGVDIAEVDLNRCEPWELPEAARMGEKEWYFFS LRDRKYPTGLRTNRATGAGYWKATGKDREVVAAAAAGGALIG MKKTLVFYKGRAPRGEKTKWVLHEYRLDGDFAAARRSTKEEWV ICRIFHKVGDQYSKLMMMKSPASYYLPVSHHHPSSIFHDLPPVPFP NPSLVPFHHDLPTSFHPPLLQHSHANSKNSSSNNGGFVFPNEPNT TNSSDNHISCNGAMAAAAAAAFPSFSCASTVTGKGGPPAQLGV NAGQQEPPPPTWMDAYLQHSGFIYEMGPPAVPRGA
LOC_Os08g42400.2	MERAAAAAPVVVRHGGVVLPPGFRFHPTDEELVVQYLRRKAFG LPLPAAVIPDLHNLFKLDPWDIPGASSDGDKYFFAVRPPAARGRR QHVTASGGCWKPAGGRDKPVVVARCGGSHLVGVKKGMVFVPR QGRKAPAAAAAAAGGGCWVMHEYSLALPMHKKVRNHY
LOC_Os08g42400.3	MERAAAAAPVVVRHGGVVLPPGFRFHPTDEELVVQYLRRKAFG LPLPAAVIPDLHNLFKLDPWDIPGASSDGDKYFFAVRPPAARGRR QHVTASGGCWKPAGGRDKPVVVARCGGSHLVGVKKGMVFVPR QGRKAPAAAAAAAGGGCWVMHEYSLALPMHKKNG
LOC_Os08g42400.1	MERAAAAAPVVVRHGGVVLPPGFRFHPTDEELVVQYLRRKAFG LPLPAAVIPDLHNLFKLDPWDIPGASSDGDKYFFAVRPPAARGRR QHVTASGGCWKPAGGRDKPVVVARCGGSHLVGVKKGMVFVPR QGRKAPAAAAAAAGGGCWVMHEYSLALPMHKKGCLAEAEEW VVCRIFQRSSSGSRSPRRPDNDVRRTMPAVAELGRSPSPSSSSSQSS CVTSSSDQEEVSSG

LOC_Os08g44820.2	MSHPSSSSSSAPPAAAEATSLAPGFRFHPTDEELVSYYLKRKVHGR PLKVDAIAEVDLYKVEPWDLPARSRLRSRDSQWYFFSRLDRKHA NRARTNRATAGGYWKTTGKDREVRNGPTTVGMKKTLVFHAGR APKGERTNWVMHEYRLDGQTTIPPQDSFVVCRIFQKAGPGPQNG AQYGAPFVEEEWEEDDEDVGLLPVEEKDNSDDQEKEISGAMEKG YLQMSDLVQNLVDQNENGTIALPVSDNSNNSNHSEDVDGNSGD ILSDQNLGSNFLHHVEPVEQNGLVLNENMFSSANAGDLFNISSPN DGFLELKDFADIADLENPLANESTIWPSDGWPWKSTDSMEAVNG ASNEFSPLAGEQIFQPEELEQLLQSLQEDSHMGSTISDPPHSSITNL AKPEEDCLMFYDAPFDSSMCDDGFRQLNGFLGSPSTNLSGIDMV DDGMPYYDAMDDNLFNDLLSSVQPSAGSSSHAFSGPVLTQEVN NSTYTYSPTQKVLEPNFVVGAPSSARLPEAGSQLNYVVLPGNITM EKYHRSIIISCSLVLIHHGKRAFSALFGPLIINLFFFAMRNCSLLYLL VLIEQDLSITRSFLWRHSYLAQSVQCYTQLACDFSLPGH
LOC_Os08g44820.1	MSHPSSSSSSAPPAAAEATSLAPGFRFHPTDEELVSYYLKRKVHGR PLKVDAIAEVDLYKVEPWDLPARSRLRSRDSQWYFFSRLDRKHA NRARTNRATAGGYWKTTGKDREVRNGPTTVGMKKTLVFHAGR APKGERTNWVMHEYRLDGQTTIPPQDSFVVCRIFQKAGPGPQNG AQYGAPFVEEEWEEDDEDVGLLPVEEKDNSDDQEKEISGAMEKG YLQMSDLVQNLVDQNENGTIALPVSDNSNNSNHSEDVDGNSGD ILSDQNLGSNFLHHVEPVEQNGLVLNENMFSSANAGDLFNISSPN DGFLELKDFADIADLENPLANESTIWPSDGWPWKSTDSMEAVNG ASNEFSPLAGEQIFQPEELEQLLQSLQEDSHMGSTISDPPHSSITNL AKPEEDCLMFYDAPFDSSMCDDGFRQLNGFLGSPSTNLSGIDMV DDGMPYYDAMDDNLFNDLLSSVQPSAGSSSHAFSGPVLTQEVN NSTYTYSPTQKVLEPNFVVGAPSSARLPEAGSQLNYVVLPDSQTKS SLIGKRFVKILDSISAPPAFAAAEFPASLRKSLAPISGAHHNTIRVSA EVISIGSLTPDSQDKWSLEKDEGMELLFSAGFEPDTRVHFGCNTIT AVLRGGFCLFFFSAIMLLVSYEVGMCIYGK
LOC_Os08g44820.3	MSHPSSSSSSAPPAAAEATSLAPGFRFHPTDEELVSYYLKRKVHGR PLKVDAIAEVDLYKVEPWDLPARSRLRSRDSQWYFFSRLDRKHA NRARTNRATAGGYWKTTGKDREVRNGPTTVGMKKTLVFHAGR APKGERTNWVMHEYRLDGQTTIPPQDSFVVCRIFQKAGPGPQNG AQYGAPFVEEEWEEDDEDVGLLPVEEKDNSDDQEKEISGAMEKG YLQMSDLVQNLVDQNENGTIALPVSDNSNNSNHSEDVDGNSGD ILSDQNLGSNFLHHVEPVEQNGLVLNENMFSSANAGDLFNISSPN DGFLELKDFADIADLENPLANESTIWPSDGWPWKSTDSMEAVNG ASNEFSPLAGEQIFQPEELEQLLQSLQEDSHMGSTISDPPHSSITNL AKPEEDCLMFYDAPFDSSMCDDGFRQLNGFLGSPSTNLSGIDMV DDGMPYYDAMDDNLFNDLLSSVQPSAGSSSHAFSGPVLTQEIPRP RVV

LOC_Os09g12380.1	MADADADACPAVFASRHPTEQELISSYLHPRLLLTTTKPAAAVA<br>AGGVPSFIHHADAYAADPADLTARHLPARAADGSRAWYFFSPV<br>RTTTERGTRRARAVESGDGCWHSESGVRAVVDAAGRRVGHRQF<br>FSFVKKREEDGKRVRTGWLMVELGVDNDAASASSSNELVLCKIY<br>MTPRMPPPSPPSAVTSSAAATMELMPRAPPPSAPSAVTSPAATTM<br>ELMAGGVHKRRKISDEIAAAATPPHPQQQRRQRCVPDNDGSKES<br>SGESSSVVILDDDDDDADAPEDGGAVRSKLRSDDGVMLADARD<br>DEQHAATSDSMAGTSGGAVTGGGHGKLLPDLNVVATVAHDDE<br>GRHARGAPRPQDGGTSTTTTMVASAGAERGSTTGHLPAATAGY<br>RRTLMLFLEEEDDDAVEDEQQQQAPPLPPATSTATTTTRTAAE<br>ANVQRQRQPPCCTFVVHPCAVHAKMRHGAAYGCGCRVTGAVR<br>RGGYHLPRRAVHTTTTGQ
LOC_Os09g24560.1	MSARGGVTMAGGGGGDRAPSSSSTAMISRLLPPGFRFRPTDGELV<br>AHYLARKAADAGFTSAAIRDADLYRAEPWDLLPPPRCDAAAEEE<br>EEEEERCGYFFCTRSFRWPSGTRTNRATATGYWKSTGKDKAVLH<br>GGGGGGGRPVGVKKTLVFYRGRAPRGEKTSWVMHEYRLLHGG<br>AAATASSSPTPTTVVARSEWVICRVFVRKTPDGNNDRGTTEHHLP<br>SDDAHLRSSPAPANSVDGAGHASCSFFSGANESMAPSDHFNIGD<br>DMILHGHDEEELLMMNCSSAFDLPELLDYESFSLDL
LOC_Os09g32040.1	MGMENPPLRWPPGFRFSPTDEELVLYFLKRRIATGRPTPYIADVD<br>VYKSHPSHLPERSALRTGDKQWFFFSRMDRKYPNGSRASRTTGEG<br>YWKATGKDRSICNGGGGGTASGRAVGSKKTLVYHHGRAPRGER<br>SDWVMHEYTLLADALPPAARDREAYALYKLFHKSGAGPKNGEQ<br>YGAPFREEDWLDDDDHHHDQLPAEAALPAPATTSGRAATTEEH<br>ADFELPGGDLDVLLAQIENDQDIIEAQLDFSTHVTSQVQIQHRVH<br>QGWLSDDGGKSDVADATTSGSALLMAENTCAELPIDGLEQLLM<br>QISDDQQTVEMLSGFSASVPQSQLQHDYHQGCLGVHREEVGVA<br>DSTTVSSAVVTEECTVRELQDIEGLLMQIENDQENAESLPDFSTPV<br>HLHDCHQAAFGDFQGSQRATFNIANLSTMVQESPNFDLQTGPS<br>NQITESILTTEPMNGETNAVEETSPLRSMSVLGSYDRQDGDDEFLE<br>INDFFDPEDLEQILGSTRSQNLIPADDGVFDSLQYSDAPMFLPGSF<br>DRTGVVAENHYVEFGASGIQNQGFQHTTELLAHNQVALNVRN<br>HMKDNHVVFSHSSDATIIHTVNEQPPNRSSNASQSWFNGALSAL<br>LDSVPSSPAMAAENIGLNRTLQRISSFRSQQPAREEVSSTLINTRRR<br>GGGLIFISLMVLLVAIMWTFSNGSAVKLSKGLWKFPST
LOC_Os09g32260.1	MAMGMEGSGGGGSAKKKEESLPPGFRFHPTDEELITYYLRQKIAD<br>GGFTARAIAEVDLNKCEPWDLPEKAKMGEKEWYFFSLRDRKYPT<br>GVRTNRATNAGYWKTTGKDKEIFTGQPPATPELVGMKKTLVFYK<br>GRAPRGEKTNWVMHEYRLHSKSIPKSNKDEWVVCRIFAKTAGV<br>KKYPSNNAHSRSHHPYTLDMVPPLLPALLQQDPFGRGHHPYMN<br>PVDMAELSRFARGTPGLHPHIQPHPGYINPAAPFTLSGLNLNLGS<br>SPAMPPPPPPPPQSILQAMSMPMNQPRSTTNQVMVTEQMIPGLA<br>NGVIPQGTDGGFTTDVVVGGTGIRYQNLDVEQLVERYWPGSYQ<br>M

LOC_Os09g32260.2	MAMGMEGSGGGGSAKKKEESLPPGFRFHPTDEELITYYLRQKIADGGFTARAIAEVDLNKCEPWDLPEKAKMGEKEWYFFSLRDRKYPTGVRTNRATNAGYWKTTGKDKEIFTGQPPATPELVGMKKTLVFYKGRAPRGEKTNWVMHEYRLHSKSIPKSNKDEWVVCRIFAKTAGVKKYPSNNAHSRSHHPYTLDMVPPLLPALLQQDPFGRGHHPYMNPVDMAELSRFARGTPGLHPHIQPHPGYINPAAPFTLSGLNLNLGSSPAMPPPPPPPPQSILQAMSMPMNQPRSTTNQVMVTEQMIPGLANGVIPQGTDGGFTTDVVVGGTGIRYQNLDVEQLVERYWPGSYQM
LOC_Os09g33490.1	MATPGQQLPPGFRFHPTDEELVVQYLRRRALCRPLPAAVIPDVHDATVLDPWDLPGAGDGEAYFFSFRQLAAASGGGGWRRRRAGSGYWKATGAEKPVFLRGFGCGGGGGGGGQHLVGVKTTLLFLRAKPPSRTHWVMHEYRLAAAGAVAVAAAGQTKRGNHSCMAQPGEWVVCRIFLKNNRSSRRRAGDADGETPVTGVHGHRRRQPSPSPSSSSCVTAEVSDGEGEEEVSSGSINGAPSASQREA
LOC_Os09g38000.1	MEKVQASCEGDQGICKRGAKGGHPIDQNLVGGMTMSTGVPTTVPPTPALAALKNLLIRVVAAAAGKGLSSSAEEARCLWAKLPAPEEEEEAGQQPERPPSSYRRPPTSTLMAPVGLPPGFRFHPTDEELVNYYLKRKIHGLKIELDIIPEVDLYKCEPWELAEKSFLPSRDPEWYFFGPRDRKYPNGFRTNRATRAGYWKSTGKDRRVVHQHGGRAIGMKKTLVYYRGRAPQGVRTDWVMHEYRLDDKDCEDTMPIKDTYALCRVFKKNAICTEVEELQGQCSMALLEGACQQLLASGGGGSQEQYETPSPPDVPVGSTFGGADADAEDDPDKDDSWMQFISDDAWCSSTADGGAEESTSCVALAG
LOC_Os09g38010.2	MAPVGLPPGFRFHPTDEELVNYYLKRKVHGLSIDLDIIPEVDLYKCEPWELEEKSFLPSKDSEWYFFGPRDRKYPNGCRTNRATRAGYWKSTGKDRRINYQNRSIGMKKTLVYYKGRAPQGIRTSWVMHEYRIEESECENAMGIQLQDSYALCRIFKKNVVLGEFDKKGECSSSQAKGNEEVTDFGDAGQSSGANENDKDNSWMQFIAEDLWCTNKLK
LOC_Os09g38010.1	MAPVGLPPGFRFHPTDEELVNYYLKRKVHGLSIDLDIIPEVDLYKCEPWELEEKSFLPSKDSEWYFFGPRDRKYPNGCRTNRATRAGYWKSTGKDRRINYQNRSIGMKKTLVYYKGRAPQGIRTSWVMHEYRIEESECENAMGIQDSYALCRIFKKNVVLGEFDKKGECSSSQAKGNEEVTDFGDAGQSSGANENDKDNSWMQFIAEDLWCTNKLK

LOC_Os10g09820.1	MTAESNGGNSSAAATTASNGGRRRHELVPKLRLPPGYHFVPSDE ELVDFYLRGKIEQRRPPMDFINEVDIMSFDPVKLIEKYKGYGENR WYFFTVRKPSKTKKKDEPNRKVVVDGVEEGSWSATGSVAYICGK DHETVIGTKRVLTYKSARSAEEDKWSMHEYVMLDKSQILDTTRD TMSMDQYVLCAIQLKQTYEAEKKAQEEEERGVKRKRTATRKRRK GDIDQTTSQEQEDQQQETPPPGDPHDQSVVDAPYYSTQMALGGE EEVAPVPWCADCMAQPDRIEYPAVWYNQQEQQPSQLVDRSMM TQGYIGDLSYIQNQFDQQQAHDHGSINAFDEALDQCHDTNFAW DNAGIYPGNNLLDGNLDDDTQDQFGNQSTLGALTGELEYGTGY QFHDALQATPGSDDASAQSMGIQPAAGHSMGDDDETCCNDDL SSLLADISRILLDGNGVINNEGNPEGSNQGLHICKDGHQWPLEES TMPTVECVLQHPGESA
LOC_Os10g21560.1	MSSKVPGLALLNTSISKFWSDEELVRFLAERKEAHSLPENVFVGM NISLIDPRNSEDIWYMNFSDDPQSPKNGENAIIKSKTGYWKVVGT VRIPTSTVIVGMKVSLDHYEGEAPSGKRTGWVMDEYLIEQNDEA NLPQDYKNLCTIFFQGDDILNAGDKQICLNANVPNERKEFYLQY LAELEEQNAAWSNQAVSVNEQDVSSSKGLDGQKTSAADDQSVN HAPSREGYIELNDFLNSDSSASTSEYSSQRTMISEEYFDSDAFLREIR NDHNAADEEHTDSKFSVAAASKSDCVVISPPEQGFVNNLDNHA TIAGDSPQKSVKNDKVDEHSSEEHPQHSPTTSCFPSHVKRSLSSSSS SSQGTSNSKSPQRQRERSTKKIGKLGKYWCCGSL
LOC_Os10g21560.2	MNFSDDPQSPKNGENAIIKSKTGYWKVVGTVRIPTSTVIVGMKVS LDHYEGEAPSGKRTGWVMDEYLIEQNDEANLPQDYKNLCTIFFQ GDDILNAGDKQICLNANVPNERKEFYLQYLAELEEQNAAWSNQ AVSVNEQDVSSSKGLDGQKTSAADDQSVNHAPSREGYIELNDFL NSDSSASTSEYSSQRTMISEEYFDSDAFLREIRNDHNAADEEHTDS KFSVAAASKSDCVVISPPEQGFVNNLDNHATIAGDSPQKSVKND KVDEHSSEEHPQHSPTTSCFPSHVKRSLSSSSSSSQGTSNSKSPQRQ RERSTKKIGKLGKYWCCGSL
LOC_Os10g25620.1	MAELPPEGRGGGGNNNGKWKGKEKVVPEYGKNRHGMSVGWY FVPKDLELFAILKCKLVRGQLPGALNNVFEHIRILEFHPALLHEMY IKNEEDGYIYFFSKRQFTTKAGNKRRPTRVTKGGTWKASGGSKTV RSKKVGGIDVGQKLTMVFYERRFEGDRNPIKTNWGMHEFTKIID GTKNQLEDLAVYRLYKIKRKEDEEPSNTAAAASSTDEPSTSSALPP PTPPRPLPDMAGPSSATPLLPLQLPGLAGSSSAMSLPALQLPGMA GSSSAMPLPPLSLPGLAGGMMSMADQANMASTSQASTPSSELLQ DWYDEFEITYGAVAPPSPSTISWVAPQSSPTGWWPSPNGGPVQH DGYLGMAADPTSYMLENLLPTAAIPPEPMMPPTSSPAPPPAVDH HHRLSPPHDAAGSNYNHPELADYNGGVQAQHEHQYHPQEPQA SLVDAEDGYSAMAGGDDAQLGGAELDTERIAEMVNHIMDGEFE FKFEDNTVLKYNEVFPDNDEVVAAPMMIDGGRDGDGADGGDG DDPFDN

LOC_Os10g25640.1	MEGVDRIGWNLGLGFLRKERPPVHGDSGGSGGSNSNDGKWKG<br>KEKVVPEYGKNRHGMPVGFYFVPKDLELLAILMCKLVRGKVPGA<br>LNNVFKHIRILNSTPPSSMTYIETMEDGYIYFFSKRQFATKARNKR<br>RPMRVADGGTWKASGGSKKVGGIDVSQKFTMVFYERRFEGDRN<br>PVKTNWGMHEFTKIIPGTKN
LOC_Os10g26240.1	MGSGRGRRRWCPSTARTGTACLSAATSSPRTLELFAILRCKLVCG<br>QLPGALNNVFEHIRILEFHPALLHETYIGKEEDGYIYFFNRWQFAT<br>KAGNKRRPTQVAKGGTWKASSGSKTVRSKKVGGIDIGQKLTMM<br>FYERRFEGDRNPIKTNWGMHEFTKIIDDSKNQKPGSPC
LOC_Os10g27360.1	MAAPGDGEEKKAGSGRNKHGFPRGFRFVPRDQELLDILDDKLRG<br>APLDRALDAVFHDTRILDFHPAKLYGMYAEDEENGYIYFFSTIEFK<br>AAKPKQKKWPRRAAQGGRWKAVLGSSQMVEVGGVPVGRKLSM<br>EFYVKGVRTNWGMHEFVRIIGPNIEVADLAVYRLHKLWTNGEEK<br>PGDLAADVAKSTNQSGQASAADYYQTYQNAVSQAYAYAPPYVL<br>QPGWSQGYPYDVAAAPPTAPWPVCWAPPSAPGSYDCCYASTFS<br>RPPPPPPIAASTLDKAPITSTDHGASTNTSAATPVANNKPPPPPVA<br>ATATTLGKKGEGKGKAPTTTSTDHAGSTNTSAPPAANYQPPPTT<br>TTPPLQGTQHVFAPGVVVGHEDEEGYLIVDEVNTWRNTQQLVLE<br>DDDDDDDGRAAGAGAGEGGASASGR
LOC_Os10g27380.1	MAAPEGQNKFAGVRFLPKDLELLAILDAKLRGSPLGPVEAIFHDT<br>QILDFHPYKLYGIHRLDRSISLSLSLCRSPSSSPATEMYAEDEEEEGY<br>IYFFSTMQFRCRKIVERAAQGGRWKVNNCETLEVGGVAVGRKFT<br>MNFYEHIGGDNDLIWTNWGMQEFARIIGPNKELADLALYRLYK<br>KKITRGTGEEKPEDIAAASDGDTESSMNKRRRVEASAAAMALPPP<br>PPSPGLPGTMMFMAADQANVASTSQEWHGQFANGAAAAPSPS<br>GCWPWAPPPTPSAVEPFSFWASASAATPPAAANYHPSPQPQPLP<br>PQGGEYYSRHGAFSVAPVPASACSTPSPEAATSCLLATTSPLPAAG<br>TEGSDSQQQEPPCELMEF
LOC_Os10g27390.1	MAAPEDGEDKNFGKNKHGLPIGFYFAPTDQELLAILEAKRLGRP<br>LSRAHDAFFHDIRILDFHPAELYEKYAKDEEKGYFYFFSKREFPTSS<br>KKRPLRVAEGGAWNSSGAVYKVVKSSKSGGGYDVGHKKTLVFH<br>QRFPGDKEAVKTNWAIQEFTRIIGPQNEVPDLAVYRLYKMRKEG<br>RETPADLAADEAAAAAAMNNRGQQASAAAMALPPPATGLPGG<br>RMMSMADKANMASTSKAYGPSKSSSSQLQQDAAAAAAPPNAA<br>GASNWAPRPCNCRECAPAAGHYGYFAAAAAMNNRGGQASAA<br>AKALPLPAPGLPGVRRMSMADKANMASTSKAYATSQSSSSQLQQ<br>GAAAVAAPPNAAGPSNWAPRPCNCRECAPAAGQYGYFASMVP<br>RPSLDRKGKGKAPMDCAEQAGGGGGCHAESTSTPAPPKGAEYY<br>GCSVAVEDDDEELLKFLQAMVRGEEVEGDGDHAMADERGPQQ<br>GSSPVAAAAASGSAPAGHDGRRGSLQGGHHGSSSPTSLAAAAAT<br>GDDVTSAASAGDDVSGSQQEDHPAR

LOC_Os10g29680.1	MNKHGFPRVFRFVPDDLELLNILDDKLRGVPIDRADDAVDFHET<br>RILDFHRPTSYRCPFFFSGDGLRAPGDCMRICGHLLLQHEEEEGGR<br>GSLLRRISRLAASRPQSAPTPTTSSVSGAAARVSTADRVLDRMKLV<br>RAARSGRWKSLGSCKKVGVRRRRRPEEHVFDGDKNPVVLERGH<br>GGTGRRVRQDAVGARGSERPVESLRQLQLPLAGISPCRSTNTCGG<br>HGAAIAHRAPARGRGPVTMMFMANQANVAPHRTQSSMSELHQ<br>EWHQTTSTPTMPRGGAEF
LOC_Os10g33760.1	MAGLREMESTLPPGFRFCPSDEELICFYLRNKVANHRVASGTLVD<br>VDLHAREPWELPEVAKLTAEEWYFFSFRDRKYATGSRTNRATKT<br>GYWKATGKDRIVHEGTTRAVVGMRKTLVFYLGRAPNGQKTTW<br>VMHEFRLETPNSQPKEDWVLCRVFDKKKPSTIEAEGGGSSGSDLFI<br>PGATDGSTDPSSPTTMAPLLGSSPDPTVVDRFDHRSAAVPPLMVL<br>MQGGGDQMISGSGVHCSNNDNSGSSSALLNLTMLQYSFLEHRPT<br>GDDMAVGAHFGTCQGGNNDATMALGMGFEEHGMGEIIEMEP<br>AWRQGGSNCVYRDELYF
LOC_Os10g38834.1	MVIMESCVPPGFRFHPTDEELVGYYLRKKVASQKIDLDVIRDVDL<br>YRIEPWDLQEHCRIGYEEQSEWYFFSYKDRKYPTGTRTNRATMTG<br>FWKATGRDKAVRERSRLIGMRKTLVFYKGRAPNGHKTDWIVHE<br>YRLESDENAPPQEEGWVVCRAFKKRTMQPPRSSIGAWEASYSYH<br>DPAVFVGGGEHFKQEAAAELDGVAAAAGANAFLRYSTRLAELP<br>QLESPPLPSQGSQAASAVVDGEEDNADSSRRPGGGGGAAAAVTT<br>DWRAFDKFVASQLSPEEQHTCRATDDDDMAALLLLDGGGQED<br>DAGRWLGSAGLLSAVAADATTDCGLGTSCVPGDIN
LOC_Os10g42130.1	MGTMTLPPGFRFHPTDDELVGYYLKRKVDSLKIELEVIPVIDLYKF<br>EPWELPEKSFLPKRDLEWFFFCPRDRKYPNGSRTNRATSTGYWKA<br>TGKDRKIACAGEVFGLRKTLVFYKGRAPGGERTDWVMHEYRLC<br>QDLAHGVSNFIGAYALCRVIKRHEAGLHGEPPAAKAKGMISKVS<br>SSSSLVTVEHQLSSRGNASPSFTPTNNGSPLVDEMFVGGGGGDPF<br>QLLPSCVPYHGGDACGFDLPPLCMPQTQDPFFSDAGFTQAAPPL<br>YGDVMGSVSEHELKWDTLGGYSGGGGGELWNAAAAPLLCRQA<br>SDGDDLTAWFTAADDNMSVF
LOC_Os11g03300.1	MPSSGGAMPALPPGFRFHPTDEELIVHYLMNQAASVKCPVPIIAE<br>VNIYKCNPWDLPGKALFGENEWYFFSPRDRKYPNGARPNRAAG<br>SGYWKATGTDKSILSTPTSDNIGVKKALVFYKGKPPKGVKTDWIM<br>HEYRLTGTSANSTTTTKQRRASSMTMRLDDWVLCRIHKKSNDFN<br>SSDQHDQEPEESTVEQLEDIHDNNSSEQPPAPADMNNQQSDFQP<br>MTAMSMSKSCSLTDLLNTIDCAALSQFLLDGSSDAIAEPPAPPSPL<br>IYTTPHPNYQTLNYNINSNSSMPHAFESRLDHHDGYVNNYNVN<br>GLRRKRMMACSATSFDDGSSSNDFVHAVVKKPQLLPSDSRGSGF<br>GGGYCNQQLSETATGFQFQNGNLLSHPFPLNNHLQMQ

LOC_Os11g03310.1	MGNNEWYFSRKDMKYPTGMRTNRATKEGYWKATGKDREIFKP<br>AIYEGSSKNNKQLVGMKKTLVFYMGRAPKGTRTNWVMHEFRPH<br>ANLHNHYPNLRLNPNEWVVCKVFHKKQGDEAINNQQQQPAV<br>DQADDDDIFQLDDIFADPSIYDFSNSSANILSAPPNNNAVHSSVSA<br>GTTMTSTTTASSFQHQPNCYSAPLQQHVSSWNNTPGAGGAHGI<br>GSSYYNLQQQQQQAAMVKDLEDIIAVPDYGTLLPSSNKGSSIRSA<br>TAGVSQQNPLGVPQYKIENYGDHYISRE
LOC_Os11g03370.1	MVETSTSLVKLEQDGSLFLPPGFRFHPTDAEVILSYLLQKFLNPSFT<br>SLPIGEVDLNKCEPWDLPSKAKMGEKEWYFFSHKDMKYPTGMR<br>TNRATKEGYWKATGKDREIFNLQPTSYGGSSNNKNNKQLVGMK<br>KTLVFYMGRAPKGTKTNWVMHEFRLHANLHNDNPNLRLNLKD<br>EWVVCKVFHKKGDDREAINKQQAQAAAVDQYSAGTPNNGSSV<br>EAGDDDDDLFQLDSIIDPSIYFSNSSAANILSAPPNMSNSVVAANY<br>GASTTTTGTASAGSFQQQPNYCSLINKSISSSNVSSWNNMPPPPPV<br>AEGGVHGIGSSYSLQHQAAMVKALRDVIRLPNPLGMPQYKLDD<br>AYLWDSS
LOC_Os11g04360.1	MATLPQMAATKERQEAANPTTTTRTLVESVTNWIRVYSDGSVDR<br>LGPPEAAAFMVLVPPYDDPRDGVTVHDVATDHGVDVRLYLTTT<br>APARRRPVLVHFHGGGFCLSHAAWSLCHRFYARLTVDLDVAGIV<br>SVVLPLAPEHRLPAAIDAGHAALLWLRDVASGGSDTIAHPAVER<br>LCGAADFSRVFLIGDSAGGVLVHNVAARAGEAGAEALDPIRLAG<br>GVQLHPGFILPEKSPSELENPPTPFMTQETVDKFVVLALPPTKDTE<br>KFICRADVYGSEPSDLAGKFAPVPRCEKGGRLFFTSCKRHKGSSTR<br>KERTAGDGTWVRQNSKGVKNKAGVKVGETQNFRFKKDGSYTD<br>WLMEEHHCCRQQAVAGDEEPVICRMYVSPRAPPDSAARQESAA<br>FVQQQPAPQVSEPPCDKKKRDDVAEEAPAAA
LOC_Os11g04470.1	MAEEDDKKQKGPDVTVPSGYFFVPKPEQLIRDYLNHWITGRPSEE<br>LRDIVREADVYGSDPATLTEAHSAYGHDGKSWREKTTGASQQNI<br>FTISRRGGFEGGGTWHNSQRRRVIEGYGDRQAFEYRAPGNKKTD<br>WLMEEIASNLPAAITDEGIMVICKVYLSPRAKEATANEEERQETN<br>VVPGPKRLREAEATGYDAPAPPQPDVGYSYSGGGETSQATASMD<br>YCCSTTTHTADDTANAAYYHGDADAIKPDAYDGGDYGIGFNAD<br>GELVLCGNGHGGIGTQGQTPLAMQNTNGEMTLFSPMNGYGVG<br>FNEEVRQEPQVEGEVEMDNFFNDLFVDFDGAGDLNPNPNGGGD<br>SHGHILCE

LOC_Os11g04960.1	MNPTIENGGSGGDGSAAAAAAEGSAIWKSELVPQLQLPPGYHFV<br>PTDEELVDFYLRGKIEGRDPPRHFISEENIMRYDPQKLIEKYKGYG<br>EDRWYFFMVREPSKTKKKDEPNRKVVVDGVEEGSWSATGSVVQI<br>HSTKETNRKAIIGSKRVLTYKSARSAENDMWSMHEYVLAGKSQM<br>GQYVLCAIQLKQTYEREEKAREEQKNDNKRNKKAARRKNMQQ<br>QPTACQAQDEQQETAPTPGEETIVDPDQFMDIAHSMHMMFGGV<br>DQDAPPFMPSLIAPCNNDDGMLQLQPLQLQNPNPAMLYSNQLE<br>PSYIGDQSMFTPCCCDRNCISCRQLQFYQQQQAEDGSVAFGEAD<br>LYQQHDRALGNTGVYPDNVWVDGNMADYAQRQIYNDQDNG<br>GVLMQGPEDSATFPDNFLMLDEMAAGSDDASGFDYEVDQSMA<br>VVPHVADQTVDDIMSSLLN
LOC_Os11g05614.1	MATTRSGVGGAISDPFATPGFRFYPTEEELLGFYLRHRLAGTRPDV<br>ERVIPVVDVYGYHPSQLAALAGEASARDTEQWFFFCPRAERELH<br>GGRPARTTPSGYWKATGSPSCVISSATNRVIGVKRTMVFYQGRAP<br>TGTKTRWKMNEYKAVADDADAAAAAMLHPMAPPRLRNELGV<br>CRVYISTGTLRSFDRRPLDNQAAAPTQQQVMPSLTAAAAVNTNL<br>CGGGGGVVFAGAQGDSSRDCSSSSGSRELAGGADGSEDDAIDW<br>NSLISSATADDLGFNTVVGFDPSIVGSWPQV
LOC_Os11g07700.1	MASTSRKRSARSLQEDEQTSSAAEAPAAVREDEERVAVAGMEA<br>WRFGFSRFSWFPAFKFDPTDADIVASYLLPRALYGRGHAAVIQDD<br>VSRCEPWTLMREHGHATSAHAFFVHDHESVGGGGGGGRRKVQ<br>RAVKNGGGVWRIQKGEVAILTIVRGGGGGGGELDVVYKRRNLSF<br>HRRGESSSSGWVMHEYEITSPPLPATVLSRIRATPRAKDKKLCIKE<br>EPSCSTSAAGERSGPNPDHTAAGAGDSATANHNNTTSAATTTM<br>AAAV
LOC_Os11g08210.1	MECGGALQLPPGFRFHPTDDELVMYYLCRKCGGLPLAAPVIAEV<br>DLYKFNPWDLPERAMGGEKEWYFFSPRDRKYPNGQRPNRAAGT<br>GYWKATGADKPVGSPRAVAIKKALVFYAGKPPKGVKTNWIMHE<br>YRLADVDRSAAARKLSKSSHNALRLDDWVLCRIYNKKGVIERYD<br>TVDAGEDVKPAAAAAAAKGGRIGGGGGAAAMKVELSDYGFYD<br>QEPESEMLCFDRSGSADRDSMPRLHTDSSGSEHVLSPSPSPDDFPG<br>GGDHDYAESQPSGGCGGWPGVDWAAVGDDGFVIDSSLFELPSP<br>AAFSRAAGDGAAFGDMFTYLQKPF
LOC_Os11g31330.1	MANTGLSIPMVNGATIHLLPGFRFRPTDDELVIKYLYPRAFHVPLP<br>CAIITDVDIHHHNPWDIVPVAEREKGKHFFTRKEVKYPGSRRSNR<br>VAGNGFWRAAGSEVPIYYKPEGAANDMLVGMRRTLVFHYGKSR<br>SAERTEWAMHEFQLAGAGLLPHPMMRHATSNGSEPPCGCLEAT<br>IAKKSDGLSATLRAKRDSAPLMRIMVEPDSSWVICCIYKKRQRAP<br>PVVIPPVIGDVGEAIIPHAIGDAREGQLHFIDFLGQPARNDPSSPHS<br>CTIDPSSLEEGSDESAGDGEDKDGDGMNEAN

LOC_Os11g31340.1	MASPGVCINLVNGTSTRLPTNADLVVHYLHRRAIQEPVPCDFITNVDILQHNPWDIVPAEEKTNGKYFFIHEENERLGNHHSNRAAGDGFWRPVGSEVPIYHKRSGGADEALVGMKRTLVFHYGNSSSAKRTEWVMQEFRLAGATLIPCPVTRPATGDGSMLPCHRTGTTIATENNGSPSAGQTHGPLEKTMVEPDSSLRICRIYKKRQRTPQFIIPPSIGDARELILALPTIGNTREVALALPAIDFLGQPSFEEGSDVSADVITDDKDGYGHGMN
LOC_Os11g31360.1	MAGPGVCINLLNGTTMHLSVGCVFRPTEGELVVNYLYRRAMQEPLPCDFITDVDIQCHNPWEIVPAGEKKNGKHFFTRKENSHPRDYESNHAAGDGFWRLAGTEVPIYNKPSGGADEKLVGMKRTLVFHFRKSSSTERTGWVMPTCRCQPCALPCDEAGHRCHLYHGCTLIDYMRTVLQKNNGSPSAAHTHAPLVETMVEPDNSWMICRIYKKRQRAPQVIIPPSIGNAREAVLAVPAIGNAGDRQVTSLTSQGIDVSRRGVMSLPMSSQRTRAVMVMGRTNRRKKLGLV
LOC_Os11g31370.1	MAGPQMSLPYDFITDIDILHHNPLDIVPTRQEKKNGKHFFTRKEKKHHGDNCRNHAAGVGFWRKSSFAESMEWAMQEFQLAGSYLLPCFVMRFATSDGTEQPFGCTRVTIANMRHCILTPYCCFIG
LOC_Os11g31380.1	MHLPAVGMSHPTEGELVFHYLYRRAVNMPLPSEFICDVNVLPHNPWDIVPGSEKPVYYNQGGGSDCMLVGMRRTLTFYFGNSRTAERTKWGMQEFRLAGNGLSPYPAMKHATGDGSKPPCNCAETTIAKRNDGLSAVLRNVLAVTPLVETVVEPDGSWLICRIYRTRQRALPVITPPAIENAREIIIPPANGNAREAQVRFIDFLQQGSHIESSSPCSCIVGPSLAEGSDESAGSVDQKD
LOC_Os11g40350.1	MARTARRVASSSPTAATPTAEDGQDGGGGGERGEGDGEDGKSGRHGTRGGSGKGWRGEKPRQSRQLRPLPRRNVSMVLDTSFTSAAIRDVDLYRAEPWDLLPPR
LOC_Os11g45950.1	MGAGAGEPWFYFRHHERWMHRMDRSTPSGYWKTAGKASFVYSADRHPVGLKKSMLFYRGPEPSGRKTKWKIDEFWALDNAANGSGELLAQLCRSRQNVGLMPRFPLSLCRLYSTKSSSERHVLATSSDEISEDESD
LOC_Os12g03040.1	MPSSGGAMPALPPGFRFHPTDEELIVHYLMNQAASIKCPVPIIAEVNIYKCNPWDLPGKALFGENEWYFFSPRDRKYPNGARPNRAAGSGYWKATGTDKSILSTPTSDNIGVKKALVFYKGKPPKGVKTDWIMHEYRLTGTSANNTTTTKQRRASSMTMRLDDWVLCRIHKKSNDFNSSDQHDQEPEGSTVDEQLEDIHDNNSSSQQPPAPPDMNNQQSDFQPMTAMSMSKSCSLTDLLNNLDCAALSQFLLDGSSDAIAELPAPPSPLIYPNQTLNYNINNNMPHAFESRLDHHDGYVNNYNVNGLRRKRMMACSATSFDDGSSSSSSDFLHVAKKPLLLPSDSRGSGFGGGYCNQQLSETATGFQFQNGNMLSHPFPLNQQLLLNNHLQMQ

LOC_Os12g03050.1	MVESTTSLVKLEQDGGLFLPPGFRFHPTDAEVILSYLLQKLLNPSFTSLPIGEVDLNKCEPWDLPSKAKMGEKEWYFFSHKDMKYPTGMRTNRATKEGYWKATGKDREIFRQPAAVNTSSYGGSSNKKKQLVGMKKTLVFYMGRAPKGTKTNWVMHEFRLHANLHNHHPNLRLNPKDEWVVCKVFHKKQGDEAINNQQQPQYAAVDQYSAETPNSGSSVVQAGDIDGGDDFFQLDDIIDPSIYFVSNSSNILSAPPNNNNAVYSVSASTTTTNTTAVSFQQQPNYYSLINKSSSSSSSNYSAPLQQHVSSWNITPGAGGAHGIGSSYYNLQQQQAAMVKALENVIAVPNFGTLLPSSNKLKGLSKSAMAGLTQQNPLGVPQYKIENYGDHYISRQ
LOC_Os12g04230.1	MAEEDDKKQKGPDVTVPSGYFFVPKPEQLIRDYLNHWITGRPIEELRDIVREADVYGSDPATLTEAHRAYGHDGKSWYFLTVAKWKGGRGGAGTAGRLNRCVEGGGTWHNSQRRRVIEGYGDRQAFEYRAPGNKKTNWLMEEIASNLPAAITDEGIMVICKVYLSPRAKEATADEEERQETNVVPGPKRLREAEATGYDAPAPETPQPDVGCSYSGGGETSQATASMDYCCSTTTHTADDTANAAAYYYGDVDAIKPDAYDGGDYGIGINADGELVLCGNGHGGIGTQGQMPLAMQNTNGEMTLFSPMNGYGVGFNEEVRQEPQVGGEVEMNDFFNDLFVDFDGAGDPNPNPNEGGDSHGHILCE
LOC_Os12g05990.1	MVMSGGGGGARIVSDPAATPGFRFYPTEEELIGFYLRHRLAGTRADDVARVIPVVDVYGYHPSQLAAMAGVATAGDREQWFFFCPRAERELHGGRPARTTPSGYWKATGSPSFVFSSSAAAAARVIGVKRTMVFYQGRAPSGTKTRWKMNEYKAVAAAAADDDHNAAGVAVQLPPMAPPPSSSACVRLRNELSVCRVYVSTGTLRSFDRRPLDAPPVISHHQPQLQQQQRQLPSSAAAAATNGNLIALAGGYECSHDSSGGSSEDAAIDWSSLITAATDSATAAVDFSFNDDIDFSPAAVGPWAPQL
LOC_Os12g07790.1	MDVDPPPPLPPPPPPPPPPATPQQNKAVELPPGVYFNPTREEAMHHYLNRWIAGKTIPEMEAGFVAGADVYGDGPDALRRRHRPGYWCNCVYKWFFLCHRKRQSSRRTTGNKRAERVVAAGGRWKVEQGKKVLGGGGGGGERDSLGFYSSNSTKKTSWIMEEYTSSAADGAAAARGEEDRMEPVLCKIYLSPRAPAGEKRALFGEDGVAVGPDGRKRNARVTVLATLFDDVAALLGQPVAAPPLPAPGEQLGHGHGHFDDVAARFGQVAVAALPASGDLGHGDFDDDAAALLGQVAMAPAPAPGHHQQGYVVAEAPLPESGYLGHYHHDGHLAQAAAAPEQDHYLGYHSHDAHVVDAEATPEQGYHDDAHVAVAPAPEQGDLGHDQGHLAAALTPEECGEIVGAYEFHPEMVQMLSIGFAAPDEQLLPQLLDPTGGGYDMASSAIAAVGDVNAYAAAAPTPRIRPNAAEAMAATATAETMPPPLDAVAAELSAPPRGLPPELAFSALPSVQQEPSCDDGDNFGELVAEAMPPLIGENAGVDAGSDEPLPDLAGIMTELDFGHDFFSNQHRE

LOC_Os12g22630.1	MATNLHLLLELGFRFNPSPEEVVTYYLPRLIAGHPPKDTESCIHRANVYGAEPRELAAQFAPVARSSNGDRFFLTECKRIKGKVSRVAGGGSWVSQTSKDIKNREGIKVGEAKNFRFKKDGTNTDWLMEEYHLCLRQASDLEPVLCRVYVSPRAAKDSAAHQESAALTPQEPAPPLAHAPAPAPIQEPAALPRQELAPAPPRLEAVITQQQATMKMGGSVPASKATRQSCVTASAPPPRRVAPQPAPPSLRTAPAAVAPPRQVPVITQQQAPPLKRPAPPVPSPPCAKKIRGPVSASPAARQSCVAASAPPPWCVPPPPRPAPPSRRVMAPLPPYPMDPFETPPSPHAPRHDPFEPPPSPDPPIQSYAIDPPIQSYAMDPFEQPPSPYAPHGVDDMDEFTRSLEAQLEEADGDEIAAATVAPPMAQNVAPDDDMDEFTRSLEAQLEEADGDDKIDDDEIDEEIFQIPLKD
LOC_Os12g22940.1	MEKGKLDFLLELGFRFNPSPEQVVTYYLPCLVAGQQPKDTEGCIHSADVYGADEPRDLAGKYAPVARSSNGDRFFFTGCKRMKGKFSRSAGGGTWVSQSSKDLKNREGIKIGEVKNFRFKKDGKNTDWLMEEYHLCGQESGDVVEPVVCRIYVSPRAAPDSVAHQESAVLQPQEPAPLPVPAAPAPPRQVPVVTQQAPPPPPPLVPVITQDAPPLKRPAPVAAPPCAKKMRGDVSAFPVVRQSCVAAPRCAPRVVAPPPRHPPIQTYPTDPFESAPLDPFEPPPAAASVTGGHHTPQPSVPVPATPEQGFSLAASNSPELDPANIGIDMDELMRYLGNTPLDGVLPSQLFVLPTNDDEDVELAKVLEDGLQGGGGRQWQSTAVCDSSSSATGILARHGATAASSTHPDLSQGSVRAQ
LOC_Os12g23090.1	MASLDLLLKLGFRFNPSQEEVITYYLPRLIAGHPPKDTEGYIHRADVYGADEPRDLAGKYAPVARSPNGDRFFFTGCKRVKGKFSRSAGGGTWVSQSSKDLKNREGIKIGEVKNFRFKKGGNNTDWLMEEYHLCGKEAGGVVEPVVCRIYVSPRAAPDSVAHQESAALPPPQELVPPPQELAPPPYPAAQAAPQAPAPPRQVPVITQQQAPPQKRPAAPVAEPPCATKKMKGAVSAKPMAPQSSVTASAAPPRCAVAPSQHHPPFQTYPTDPFEPPAPAASVTQPSVPATPEQGPAYVPDPADIGMEMDELMSFLDSIPVDGILPSQLYEYDELAKELEDALQGGGEEDGNDNPPRRRGRGGCDKQSQGGYRVLLKDMGDDQIDQQWLKVSLKDYHHLMKSCKL
LOC_Os12g29330.1	METTAAKKLPPGFRFRPTDEELVVHYLRRRALGSPLPPAVDIPDVRLLAHDPSDLLPPGWSEQERYFFTCKEAKYVKGRRANRATGAGYWKATGKEKPVAVSVAAAPRSQAAAVVVGMKRSLVFYRGKPPTGKKTDWVMHEYRLAGAGLAPCRRAATADHPARPAEGWVLCRVFRKKGSAAASTASPTADADDDDATTERADDAAAGVRFIDFFARADARRRRAASPVSSSCVTDASAEHCREQETTSRNGGAAAGDASD

LOC_Os12g41680.1	MSMMSFLSMVEAELPPGFRFHPRDDELICDYLAPKVAGKVGFSG RRPPMVDVDLNKVEPWDLPEVASVGGKEWYFFSLRDRKYATGQ RTNRATVSGYWKATGKDRVVARRGALVGMRKTLVFYQGRAPK GRKTEWVMHEYRMEGVHDQQASSFSSKEDWVLCRVICKRKSGG GATSKSRSLTTTTTTIVHDTSTPTSSPPLPPLMDTTLAQLQASMNTS SSSAIAAVAALEQVPCFSSFSNSIASNNNNSNSATVNAQQCYLPIV TGSNNNGMSYLDHGLPEFGSFLDTQSCDKKMLKAVLSQLNSIGG EVLPGLPPPSEMAAAVSSSWMNHF
LOC_Os12g43530.1	MEEEKRLERIIKEIDSPISPGGGAALLAEDDDLVFPGFRFHPTDQEL VGFYLTRKVEKKPFSIDIIKEIDIYKHDPWDLPKVSHGAVALQGSSS SSSLSTAAAAEKECGYFFCLRGRKYRNSIRPNRVTGSGFWKATGID KPIYSSSLAAAAAAAGAGDCIGLKKSLVYYRGSAGKGTKTDWM MHEFRLPSSISDSDHLQDASETWTICRIFKRSMTYTKGRAAAAAA SMNKRISHELQHIHHHQQQQFYYHEVVHDGHGHHHRRHLQHY AGSASMAAAAANIVDVIDHSSDAETTTRSHSHSQSHLVADIRHR QSPFMLDFHAGTASSSSSTAAGWSEVMSFSRDGGSSSGSSWDELG RIMDISTNSANNNYYL

Table S5 The protein sequences of triterpenoid saponin biosynthetic enzymes in *H. helix*.

Name	Sequences
gene-ENSIDEG00000000378	MWKLKIAVGGNPWLRTLNDHVGRQIWEFDPKIGSPEELAEIDNVRDTFR KHRFEKKHSSDLLMRIQFANENPGSVVLPQVKVKDTDDISKDKVTVTLKR AMSFYSTLQAHDGHWPGDYGGPMFLMPGLVITLSVTGALNTVLSKEHKL EICRYLYNHQNRDGGWGLHIEGPSTMFGTVLNYVTLRLLGEGANDGQG AMEKGRQWILDHGGATAITSWGKMWLSVLGVFEWSGNNPLPPETWLLP YILPIHPGRMWCHCRMVYLPMSYLYGKRFVGPITPTVLSLRKELFTVPYHE IDWNQARNLCAKEDLYYPHPLIQDILWASLDKVWEPIFMHWPAKKLREK SLRTVMEHIHYEDENTRYICIGPVNKVLNMLCCWVEDPNSEAFKLHLPRL YDFLWLAEDGMKMQGYNGSQLWDTAFAVQAIISTNLAEEYGPTLRKAH TFIKNSQVLDDCPGDLDTWYRHISKGAWPFSTADHGWPISDCTAEGFKA VLLLSKLPSELVGEPLDAKWLYDAVNVILSLQNSDGGYATYELTRSYRWL ELVNPAETFGDIVIDYPYVECTSAAIQALTAFKKLYPGHRQEEIQHSIEKAA LFIEKIQSSDGSWYGSWGVCFTYGTWFGIKGLVTAGRTFSSCASIRKACDFL LSKQVASGGWGESYLSCQNKEYTNLEGNRSHVVNTGWAMLALIDAGQA ERDTTPLHRAAKLLINSQMENGDFPQEEIMGVFNKNCMITYAAYRNIFPI WALGEYRCRVLGAP
gene-ENSIDEG00000000716	MASQKNVGILAMDIYFPPTCIQQEVLEAHDGASKGKYTIGLGQDCMGFC TEVEDVISMSLTIVTSLLEKYKIDPKQIGRLEVGSETVIDKSKSIKTFLMQIFE ESGNTDIEGVDSTNACYGGTAALFNCVNWVESSSWDGRYGLVVCTDSAV YAEGPARPTGGAAAIAMLIGPDAPIAFESKFRASHMSHAYDFYKPNLASE YPVVDGKLSQTCYLMALDSCYKRYCHKYEKLEGKQFSMADADYFVFHSP YNKLVQKSFARLTFNDFLRNASSIDESAKEKLAPFSTLTGDESYASRDLEK ATQQVAKPQFDAKVLPTTLIPKQVGNMYTASLYAAFASLIHNKHSSLEG NRVIMFSYGSGLTATMFSFHLREGQHPFSLSNIANVMNVEEKLESRHEFPP EKFVEIMKLMEHRYGAKDIVTSKDCSLLSPGTYYLTEVDSMYRRFYAKKA VEKTTSTKNGYLANGH
gene-ENSIDEG00000002084	MATQKNVGIIAMEIYFPPTCIQQEVLEAHDGASKGKYTIGLGQDCMGFCT EVEDVISMSLTAVTSLLEKYKIDPKQIGRLEVGSETVIDKSKSIKTFLMQIFE KCGNTDIEGVDSTNACYGGTAALFNCVNWVESSSWDGRYGLVVCTDSA VYAEGPARPTGGAAAIAMLIGIDAPVAFESKFRGSHMSHAYDFYKPNLAS EYPVVDGKLSQTCYLMALDSCYKRYCDKYEKLEGKQFSMDDADYFVFHS PYNKLVQKSFARLMFNDFLRNSSSIDESAKEKLAPFSTLTGDESYASRDLE KATQQVAKPQYDAKVQPTTLIPKQVGNMYTASLYAAFASLIHNKHSTLD NKRVIMFSYGSGLSATMFSFRLREGQHPFSLSNIATVMNVAEKLKSRNEFP PEKFVEIMKLMEHRYGAKDFVTSKDCSLLSPGTYYLTEVDSMYRRFYAKK AVNKTASTENGTLANGH
gene-ENSIDEG00000002405	MAIIASAPGKVLMTGGYLILERPNTGLVLSTNARFYAIVKPLYEEIKPNSW AWAWTDVKLTSPQMARETTYKMSLKHLLLQCASSSDSRNPFVEYAVQYS VANALHKLLLQGLDITILGCNQFYSYRNQIEALGLPLSPESLATLKPFASIT FNAGESNGENSKPEVAKTGLGSSAAMTTAVVAALLSYLGVVNLSSLSEDQ NLEMDSEDLDVVHVIAQTAHCNAQGKVGSGFDVSSAVYGSQHYVRFSPE VLSPAQGAVGGRPLDEVITDVLKGKWDHERTKFTLPPLMMLSLGEPGTG GSSTPSMVGAVRKWQKSDPQKSRDTWTKLSNANSTLETQLSLLRKFAEE HWDSYKCVINSCSMCKSEEWMGQASEPSQVQIVKALLGSRDAMLEIRCQ LRQMGEAAGIPIEPGSQTRLLDATMNMEGVLLAGVPGAGGFDAIFAVTL GAASSTNLTKAWSSHNVLAMLVREDPRGVSLHSSDPRATEITSGISAVHIE

gene-ENSIDEG00000003422	MQDMLWYFLHNVAEPVLTRWPFSTMREKALKVAMEHIHYEDKSSRYLCIGCVEKVLCLIACWVEDPNSEAYKRHLARIPDYFWLAEDGMKMQSFGSQTWDAAFAIQAIIASNLLEEYGPTLRKAHQFMKASQVKDNPPGNFHKMYRHQSKGAWTFSMQDHGWQVSDCTAEGLKAALLFSQIQPNLVGEKIETQHAYDAVDVILSLQSKNGGFPAWEPQRAYSWIEKFNPTEFFEDVLIEKEYVECTSSAIQALALFQKLHPKYRRKEIQRCIAKAIQFIEDNQNADGSWYGCWGICFTNGTWFAVEGLVACGMKIQNSPTLGKACKFLLSKQQPDGGWGESYLSCSNKVYTNLEGNRSNLVQTAWALLSLIKAGQADVDPTPIHRGMRTLINSQMEGGDFPQQEITGVFMKNCTLNYSSYRNIFPIWCLGEYRRHVIFS
gene-ENSIDEG00000005299	MWKLKIAKGDGPNERWLTSTNNHVGRQYWEFDPSAGTPEERAEVESLRQEFKRNRFQIKQSADLLMRMQLSKENTLGPIPPAVKLKETDDITEKVITTTLRRAINFYSALQAHDGHWPAESAGPLFFLPPLVIALYVTGSLDSILSPQHQKEIIRYIYNHQNEDGGWGLHIEGRSTVFGSALGYVALRLLGEGQEDGENKAMAKGRKWIADHGGAVGIPSWGKFWLTVLGVYEWEGCNPMPPEFWLLPKFFPIHPGKMLCYCRLVYMPMSYLYGKRFVGPLTELVRSLRQELYSEAYHGINWNKARNTCAKEDLYYPHPLMQDMLWAFLHNVAEPFLTRWPFSTIREKALKVAMEHIHYEDESSRYLCIGCVEKVLCLIACWVEDPNSEAYKRHLARIPDYFWLAEDGMKMQSFGSQTWDAAFAIQAIIASNLQEEYGPTLGKAHQFIKASQVQDNPPGNFQKMYRHQSKGAWTFSMQDHGWQVADCTAEGLKAALLFSAMQPNLVGEKIETQCAYDAVDIILSLQSKNGGFPAWEPQRAYSWIEKFNPTEFFEDVLIEKEYVECTSSAVQALVLFQKLHPGYRRKEIERCISKAIQFIEDNQNADGSWYGCWGICFTNGTWFAVEGLVACGMKIQNSPTLSKACKFLLSKQLPDGGWGESYLSSSTKVYTNLEGNRSNLVQTAWALLSLIKAGQADIDPTPLHRGMRTLINSQMEGGDFPQQEITGVFMKNCTLNYSSYRNIFPIWCLGEYRRQVIY
gene-ENSIDEG00000005830	MASQKNVGILSMEIYFPPTCIQQEVLEAYDGASKGKYTIGLGQDCMGFCTEVEDVISMSLTAVTSLLEKYKIDPKQIGRLEVGSETVIDKSKSIKTFLMQIFEKCGNTDIEGVDSTNACYGGTAALFNCVNWVESSSWDGRYGLVVCTDSAVYAEGPARPTGGAAAIAMLIGTDAPIAFESKFRGSHMSHAYDFYKPNLASEYPVVDGKLSQTCYLMALDSCYKRYCNKYEKLEGKQFSMDDADYFVFHSPYNKLVQKSFARLMFNDFLRNASSVNESAKEKLAPFSTLTGDESYASRDLEKATQQVAKPQYDAKVQPTTLIPKQVGNMYTASLYAAFASLIHNKHSKLDNKRVIMFSYGSGLSATMFSFRLREGQHPFSLSNIASVMNVAEKLKSRNEFPPEKFVEIMKLMEHRYGGKDFVTSKDCSLLSPGTYYLTEVDSMYRRFYAKKAVDKTIGTENGTLANGH

gene-ENSIDEG00000005981	MWKLKIGEGDKNDPYLYSTNNFVGRQTWEFDPDYVGSPGELEEVEEARRQFWENRYKVKPCGDLLWRLQLLREKNFKQTIPQVKVGDDESVTYEVATTTLRRAVHFFSALQASDGHWPAEIAGPLYFLPPLVMCVYITGHLDTVFPAEHRKEILRYLYCHQNEDGGWGFHIEGHSTMFCTTLSYICMRILGEGPDGGINNACARGRKWILDHGSVTAIPSWGKTWLSILGVYEWMGSNPMPPEFWILPSFLPMHPAKMWCYCRMVYMPMSYLYGKRFVGPITPLILQLREELYAQPYNEIKWRKVRHVCAKEDIYYPHPLIQDLLWDSLYVLTEPLLTRWPFNKLREKALQTTMKHIHYEDENSRYITIGCVEKVLCMLACWVEDPNGDYFKKHLARIPDYIWVAEDGMTMQSFGSQEWDAGFGIQALLASDLTHELGPTLMKGHDFIKKSQVKDNPSGDFKSMHRHISKGSWTFSDQDHGWQVSDCTAEGLKCCLIFSTMPEEIVGKKMEPELLYNSVNVLLSLQSKNGGLAAWEPVTAQDWLELLNPTEFFEGIVIEHEYVECTSSAIQALVLFKKLYPGHRKKEIDNFITNAIRYLEDIQMPDGSWYGNWGVCFTYGSWFALGGLAAAGKTYDNCAAVRKGVNFLLKSQLDDGGWGESYLSCPKKVYVPLEGNRSNLVHTGWALMGLIHSGQAERDPTPIHRAAKLLINSQMEDGDFPQQEITGVFMKNCMLHYTNYRNIYPLWALAEYRRRVPLPSLGT
gene-ENSIDEG00000006287	MAPEVGFSDSIRPRDVCIVGVARTPIGDFLGSLSSLSATRLGSIAIESALKRAKVDPSFVQEVFFGNVLSANLGQAPARQAALGAGIPNTVVCTTINKVCSSGMKATMLAAQSIQLGANDVVVAGGMESMSNAPKYLVDSRKGSRLGHDNIIDGMLKDGLWDVYNDFGMGICAELCAEQHTITREEQDSYATQSFDRGIAAQRSGAFAWEIVPVEVSVGRGKPSSIVDKDEGLGKFDATKLRKLRPSFKANGGSVTAGNASSISDGAAALVLVSGEKALKLGLQVIAKIKGFADAAQAPELFTTAPSLAIPKAISNAGLQASEIDFYEINEAFSVVALANQRLLGLSREKLNVHGGAVSLGHPLGCSGARLLVTLLGVLRQNNAKFGIAGICNGGGGASALVLELMPVARVGHSRL
gene-ENSIDEG00000006820	MWKLKIAEGHGPYLYSTNNFVGRQIWEFVDDAGTPEERQEVEKARQAFTKNKLSQGVHPCGDMLMRMQLIKESGIDLLSIPPVRLGEKEEVNYEAATTAVKKALRLNRAIQAHDGHWPAENAGPMFFTPPLVIALYISGAINTILTEEHKKEMIRYIYNHQNKDGGWGFYIEGHSTMIGSALSYVTLRLLGEGPDGGKGAVQRARKWILDQGGVSSIPSWGKIYLAVLGVYEWEGCNPLPPEFWLFPSFLPFHPAKMWCYCRTTYMPMSYLYGRKYHGPITDLVLSLRQEIHRIPYEEINWNKQRHNCCKEDLYYPHSFVQDLLWDGLHYLSEPIIKYWPFNKLRQRGLRKAIELMRYGATESRYITIGCVEKSLQMMCWWAENPNGYEFKHHLARVPDYLWLAEDGMKMQSFGSQVWDCTLATQAIIATNMVEEYGDSLKKAHFYLKESQIKENPSGDFESMCRLLTKGSWTFSDQDQGWAVSDCAAEALKCLLLLSRMPTEIAGEKAKVERLYEAVNFLLYLQSPESGGFAAWEPAIPKPYLQMLNPSEIFADIVVEKEHMDCTASIIEALVAFKRIHESYREKEINSSVEKAVHFLEGKQLPNGSWYGYWGICFLYGTFFVLRGLVSVGKTYDNCEAIRKAVQFFLSTQNEEGGWGESLESCPSEIYTPLDGNRTNLVQTSWVMLGLMYGGQAMRDPTPLHRAAKLLINAQMDNGDFPQQEITGVVMKNCMLHYAGYRNIFPLWALGEYRKRVWLSN

gene-ENSIDEG00000007403	MWKLKIGEGDKNDPYLYSTNDFVGRQTWEFDPDYVGSPGELEEVEEARRQFWENRYKVKPCGDLLWRLQLLREKNFKQTIPQVKVGDDEAVTYEAATTTLRRAVHFFSALQASDGHWPAEIAGPLYFLPPLVMCVYITGHLDTVFPAEHRKEILRYLYCHQNEDGGWGFHIEGHSTMFCTTLSYICMRILGEGPDGGVNNACARGRKWILDHGSATAIPSWGKTWLSILGVYEWKGSNPMPPEFWILPSFLPMHPAKMWCYCRMVYMPMSYLYGKRFVGPITPLILQLREELYAQPYNEIKWGKVRHVCAKEDIYYPHPLIQDLLWDSLYILTEPLLTRWPFNKVREKALQTTMKHIHYEDENSRYITIGCVEKVLCMLACWVEDPNGDYFKKHLARIPDYIWVAEDGMTMQSFGSQEWDTGFGIQALLASDLTHELGPTLMKGHDFIKKSQVKDNPSGDFKSMHRHISKGSWTFSDQDHGWQVSDCTAEGLKCCLIFSTMPEEIVGKKMEPELLYNSVNVLLSLQSKNGGLAAWEPVTAQDWLELLNPTEFFEDIVIEHEYVECTSSAIQALVLFKKLYPGHRKKEIDNFITNAIRYLEDIQMPDGSWYGNWGVCFTYGSWFALGGLAAAGKTYDNCAAVRKGVNFLLESQLDDGGWGESYLSCPKKVYVPLEGNRSNLVHTGWALMGLIHSEQAERDPTPIHRAAKLLINSQMEDGDFPQQEITGVFMKNCMLHYTNYRNIYPLWALAEYRRRVPLPFLGA
gene-ENSIDEG00000007770	IKMDVRRRQLTKTVTAGETLKSQNQPSSDALPLPLYLTNALFFTMFFSVMYFLLHRWREKIRNSVPLHVLTLSEMAALVSLVASVIYLLGFFGIGFVQSVIRPSPDSWDILEDDNVILEADSCHEPCTAAIDCSLPPNPKIVHMLPQKPKSAFPDVVVVEQKQSASATITEEDEEIIKSVVAGTTPSYSLESKLGDCLKAAAIRREALQRITGKSLAGLPLDGFDYESILGQCCEMPVGYVQIPVGIAGPLLLNETEYSVPMATTEGCLVASTNRGCKAIYASGGATSVLLRDGMTRAPVVRFGTAKRAAELKFFLEDPMNFETLFLVFNKSSRFGRLQGIKCAIAGKNLYMRFTCSTGDAMGMNMVSKGVQNVLDFLQNDFPDMDVMGISGNYCSDKKPAAVNWIEGRGKSVVCEAIIKEEVVKKVLKTNVAALVELNMLKNLTGSAVAGALGGFNAHASNIVSAVYIATGQDPAQNIESSHCITMMEAVNDGKDLHISVTMPSIEVGTVGGGTQLASQSACLNLLGVRGASKGSPGSNSRLLASIVAGSVLAGELSLMSALAAGQLVKSHMKYNRSSKDVSKEREKKKGTDA
gene-ENSIDEG00000008386	MGSVGEILKHPDELYPLVKLIWSVWDAEKQILQEPHWSFCYSMLAKVSRSFCLVIHQLSPQLRDALCIFYLVLRALDTVEDDMSIPTEVKEPILMAFHHHIYDTDWHFSCGKKEYKILMDEFHHVSIAFLELRSSYREIIEDVTMRMGAGMTKYICKEVETIDDYDEYCHYVAGLVGLGLSRLLHASGVEILATDSLSESVGLFLQKTNIIRDYLQDINEIPKPRMFWPRQIWSKYANELEDFKYKENSIKAVQCLNEMVTNALLHVEDCLKLMSDLQDPAVLRSCAIPQIMAMGTLALCYNNIQVFRGAVKMRRGLGAKIFDQTRTMSDVYGAFYDFSSILMSKVSNSDPNAKATLSTLEAIQKTCDESGNLTKRKSYIIEGKPSYNSALIVVFFTILAILFVYLTAN
gene-ENSIDEG00000008611	MAIVASAPGKVLMTGGYLILERPNAGLVLSTNARFYAIVKPLYDELKPDSWAWAWTDVKLTSPQMARETTYKMSLKHLLLQCASSSDSRNPFVEYAVQYSVAAAYASLDNDKKNALHKLLLQGLDITILGCNQFYSYRNQIEALGLPLSPESLATLKPFTSITFNAGESNGENSKPEVAKTGLGSSAAMTTAVVAALLSYLGVVNLSSLSEDQNQEMDTADLDVVHVIAQTAHCIAQGKVGSGFDVSSAVYGSQRYVRFSPEFLSSAQGAVGGMPLDEVISDVLKGKWDHERTKFSLPPLMMLLLGEPGTGGSSTPSMVGAVKKWQKSDPQKSRDTWTKLSNANSALETQLSLLRKFAEEHWDAYKCVISSCSMCKSEELEQWMRQASEPSQVQIIKALLGSRDAMLEIRCQMRWMGEAAGIPIEPESQTQLLDATMNMEGVLLAGVPGAGGFDAIFAVTLGAASSTNLTKAWSSHNVLAMLVREDPRGVSLQSSDPRATEITSGISAVHIE

gene-ENSIDEG00000008720	MAEELHKWVVMVTAQTPTNIAVIKYWGKRDETLILPINDSISVTLDPDHLCTTTTVSVSPSFEQDRMWLNGTEISLLGGRFQSCLREIRSRARDLEDEKKGIKIKKMDWEKLHLHIASYNNFPTAAGLASSAAGLACFVFALAKLMNLKEDNGQLSAIARRGSGSACRSLYGGFVKWIMGKEENGSDSIAVQLADEKHWDDLVIVIAVVSARQKETSSTTGMQDSCKTSMLIQHRAKEVVPKRIIQMEDAIEKRDFPSFARLGCADSNQFHAVCLDTSPPIFYMNDTSHKIISCVEKWNRSEGTPQVAYTFDAGPNAVLIARDRKTAALLLQRLLFHFPPHSNTDLNSYVIGDKSILQDVGIQDMKDVEALPPPPDIKDNIPAQKSKGDVSYFICTRPGRGPVVLPDSQALLNPETGFPK
gene-ENSIDEG00000012525	MELGRSYVVIIDQYLIFTATFLFGFALLFALRWKREKRSGAASMEVNGAYKMTSSSEVNGHCSLEDIDGSSADVIIVGAGVAGSALAYTLAKDGRRVHVVERDLTEQDRIVGELLQPGGYLKLVELGLEDCVNEIDAQRVFGYALYMDGKNTRLSYPLEKFHADVAGRSFHNGRFIQRMREKAASLPNVRMEQGTVTSLVEKNGTVKGVQYKAKNGQEMSAYAPLTIVCDGCFSNLRRSLCNPKVDVPSCFVGLILENIDLPHINHGHVILADPSPILFYKISSTEIRCLVDVPGQKVPSIANGELAHYLKTSVAPQIPPELYKSFIAAIDKGQIKTMPNRSMPADPHPTPGALLLGDAFNMRHPLTGGGMTVALSDIVLIRDLLRPLRDLHDSSTLCKYLESFYTLRKPVASTINTLAGALYKVFCASPDQARQEMRDACFDYLSLGGICSEGPIALLSGLNPRPVSLFFHFFAVAIYGVGRLLIPFPSPKKVWLGARLISGASGIIFPIIKSEGVRQMFFPATVPAYYRAPPITK
gene-ENSIDEG00000013564	MGSLGAILKHPDDFYPLLKLKIAARHAAKQIPPEPHWAFCYSMLHKVSRSFGLVIQQLGPQLRDAVCIFYLVLRALDTVEDDTSISTEVKVPILMAFHRHIYDNNWHFSCGTKEYKVLMDEFHHVSNAFLELGSGYKEAIEDITMKMGAGMAKFICKEVETIDDYDEYCHYVAGLVGLGLSKLFHASGAEDLATDSLSNSMGLFLQKTNIIRDYLEDINEIPKSRMFWPRQIWSKYVDKLEDLKYEENSGKAVQCLNDMVTNALLHVEDCLKYMSDLRDPAIFRFCAIPQVMAIGTLALCYNNIQVFRGVVKMRRGLTAKVIDRTNTMSDVYGAFFDFSCMLNSKVDDKDPNATKTLSRLEAIQKTCKDSGALTTKRKSYIIENESGYNSTLLVILFIMLAILYAYLSSNLPNTV
gene-ENSIDEG00000014201	MWKLNIAQGKNDPYLYSTNNFIGRQTWEFDPNYGTPEDQAQVEQVRLHFWNNRHNVKPSSDLLWRMQFLREKKFKKTIPQVKVGDEEEITYETATTTLRRAVHFFSALQASDGHWPAEIAGPLFFLPPLVMCLYITGHLNTVFGAEHHKEILRYIYCHQNEDGGWGFHIEGHSVMFCTALNYICMRILGEGPDGGQNYACSRARKWILEHGSVTAIPSWGKTYLSILGVHEWSGSNPMPPEFWMLPSFFPMHPGTKMWCYCRLVYMPMSYLYGKRFVGPITPLILQLREELYTQPYHEINWAKVRHLCAKEDLYYPHPLIQDLLWDSLYILGEPILTRWPFNKLREKALQTALEHIHYEDENSRYITVGCVEKVLCMLACWVDDPNGDYFKKHLARITDYIWVAEDGMKMQSFGSQEWDTGFAIQALLASDLTDEIGPTLMRGHEFIKNSQVKDNPSGDFKGMHRHISKGSWTFSDQDHGWQVSDCTAEGLKCCLLFSTMPPDIVGEKLEPERLYDAVNIILSLQSKNGGLSAWEPAGAQKWLEVSLSTKLHTKEIKCRYVECTASAIQALVLFKKLYPGHRKKEIDHFITSAVRYLEDEQIPDGSWYGCWGVCFIYGCWFALGGLAAAGKTYNNCAAIRNGVGFLLKSQQGDGGWGESYLSCPNKVYTPLEGNRSNLVHTAWAMIGLIHSGQAERDPKPLHRAAKLLINSQTEDGDFPQQEITGVFNRSCMLHYSAHRNIYPLWALAEYRRCLYNLKGRNY

gene-ENSIDEG00000014823	MEVRARAPGKIILAGEHAVVHGSTAVAASIDLYTYASMHFPSPSDNDDTLKLQLKDLDLEFSWTIEKIKDAFHDSGDCNASSPTSFSPETIKIIAALVEEQYIPEAKIGLAAGVTAFLWLYTSIQGYKPAKVIVTSELPLGSGLGSSAAFCVSLSAAFIALSDSVNLDFNHEGWLMFGESELELVNKWAFEGEKLIHGKPSGIDNTVSTFGNMIKFRSGALTRIKSNMQLKMLITNTKVGRNTKALVASVSERTLRHPDAMTAVFTAVDSISNKLATIIESPGSDDCAITEKEVLVEELMEMNQGLLQCMGVSHASIETVIRTTLKYKLATKLTGAGGGGCVLTLLPTILSGKIVDKVIAELESCGFQCLIAGIGGKGLEICFGGSS
gene-ENSIDEG00000017517	MQPSLATLVENRELLMLLTTSVAVLIGCVVVLVWRRSSSQKPAKSFEAPKLLIPKFEAEEEVDDGKKKVTIFFGTQTGTAEGFAKALAEEAKARYEKAIFKVIDLDDYAPEDDDYETKLKKESLAFFFLATYGDGEPTDNAARFYKWFTEGKEKGEWLNNLQYGVFGLGNRQYEHFNKIAKVVDDGLAEQGAKRLVPVGMGDDDQCIEDDFTTWRELVWPELDQLLRDEEDTAAATPYTASVLEYRVVFHDRTDSSSLLNGTTSVSDDHAFYDVQHPCRANVAVKRELHTPESDRSCTHLEFDISSTGLAYETGDHVGVYTKNLIEIVEEAERLLAISPDTYFSIHTEKEDGSPLSGGSLQPPFPPCTLREALRRYADLLSSPKKSALLALAAHASDPSEADRLRFLASPAGKDEYAQWIVANQRSLLEVLAEFPSAKPPLGVFFASVAPRLQPRYYSISSSPRMVPSRIHVTCALVYERTPAGRIHKGVCSTWMKNAVSLEEGKDCSWAPIFVRQSNFKLPSDSKVPIIMIGPGTGLAPFRGFLQERLALKEAGAELGPAVLYFGCRNRKLDFIYEDELNNFVESGAISEMVVAFSREGPSKEYVQHKMSQKASEIWDMISQGAYIYVCGDAKGMARDVHRMLHTIAQEQGALDSSKAESLVKNLQMTGRYLRDVW
gene-ENSIDEG00000017676	MEEHYALGSICASLLGFLLVYTLFFKKNDRRDSVEAAESTTATTTIAIERECSSRSGAGDDADVIIVGAGVAGAALAHTLGKDGRRVHVIERDLTEPDRIVGELLQPGGYLKLIELGLEDCVEEIDAQKVFGYALFKDGKNTRLSYPLEKFHSDVSGRSFHNGRFIQRMREKAATLPNVQMEQGTVTSLLEENGTIKGVQYKTKNGEEMSAYAPLTIVCDGCFSNLRRNLCSPKVDVPSCFVGLVLENCKLPHANHGHVILADPSPILFYPISSTEIRCLVDVPGQKVPSISNGEMATYLKTVVAPQIPPELHDAFIATVEEGNIRTMPNRSMPAAPHPTPGALLMGDAFNMRHPLTGGGMTVALSDIVVLRNLLRPLRDMNDASTLCKYLESFYTLRKPVASTINTLAGALYKVFCASPDQARKEMREACFDYLSLGGVCSEGPISLLSGLNPRPLSLVVHFFAVAIFGVGRLLLPFPSPKRMWIGARLISSASGIIIPIIKAEGFRQMFFPATVPAYYRAPPVW
gene-ENSIDEG00000017952	MWKLVLSQGDDDEGPRLKSVNNHIGRQFWEFDPNLGTPEERAHVDEVRQEFQQNRFEVKHSSDLLTRFQFEREKSSSCETNLVDEEEVKVGSEDVTSNSVEVVKKALRRALKFYSTIQADDGHWPGDYGGPLFLLPGLIIGLYVMGAMDTILPNEHQREICRYIYNHQNVDGGWGLHIEGCSTMLCTALNYITLRLLQGGHDDKIRDEGLEKARRWIVDHGGATYIPSWGKLWLSILGVYEWSGNNPLPPEMWLLPYFLPLHPGRMWSHCRMVYLPMSYLYGRRFVGPINSTIVSLRRELYTDPYHHINWNLARNQCAKEDLYYPHPLIQDMLWSCLHKGAEPLLMQWPLSKIRQRALTTVMQHIHYEDENTSYICLGPVNKVLNMVCCWVEDSNSMANILHLSRIKDYLWVAEDGMKMKGYNGSQLWDVTFAVQAILSTGLVDEYGSVLKRAYDFIKISQVREDSPGNLSSWYRHISKGGWPFSTPDNGWPVSDCTAEGLKAALLLSNMPFDIVGEAISPVQLYDAVNFILSLQNITGGFASYELTRSYAWLEILNPAETFGDIVIDYQYVECTSAAIQGLKSFMRVHPGYRRKEIEACIAKAVNFIESLQLPDGSWYGSWGICYTYGTWFGIKGLVAAGRTYQNCYSIRTACDFLLSKQLDSGGWGESYLSCQNKVYTSIEGNISHVANTGWAMLALIEAGQAKRDSTPLHCAARVLINSQMKNGDFPQQEIVGVFNKNCMISYSAYRNIFPIWALGEYLNRVLQHNLTFP

gene-ENSIDEG00000018481	MDIRRRRPLKPPRPIPITESSHHHRKTPFPAAVDRSPSPTPKASDALPLPLYLTNGIFFTLFFSVAYYLLHRWRDKIRSSTPLHIVTLSELAAIVSLIASFIYLLGFFGIDFVQSFVSRADVDVDIDAEPDILEADRQPCSKLMDQPQPPPVLMSSEEDEEIVKSVVSGKTPSYSLESKLGDCYRAASIRREAVQRTTKRSLLELPLDGFDYESILGQCCEMPIGCVQIPVGIAGPLLLNGCEYFVPMATTEGCLVASTNRGCKAIYACGGATGILLKDGMTRAPVVRFSTAKRASDLKFFLEDPLNFDTLAVVFNKSSRFARLQSIQCSMAGKNLYIRFCCSTGDAMGMNMVSKGVQNVLEFLQSDFPDMDVIGISGNFCSDKKPAAVNWIEGRGKSVVCEAIITEDVVKRVLKTTVPALVELNMLKNLAGSALAGALGGFNAHAANIVSAVFIATGQDPAQNIESSHCITMMEAINDGKDLHISVTMPSIEVGTVGGGTQLASQSACLNLLGVKGANKESPGSNSRLLATIVAGSVLAGELSLMSAIAAGQLVKSHMKYNRSSRDMSKIGS
gene-ENSIDEG00000019086	MSDLKTRFLEVYSVLKSELLNDPAFEFTDDSRQWVERMLDYNVPGGKLNRGLSVIDSYKLLKEGKELSDDEIFLSSALGWCIEWLQAYFLVLDDIMDSSHTRRGQPCWFRLPKVGMIAVNDGILLRNHIPRILKNHFRQKPYYVDLLDLFNEVEFQTACGQMIDLITTLVGEKDLSKYSLPIHRQIVQYKTAYYSFYLPVACALLMSGEDLEKHTNVKDILIEMGTYFQVQDDYLDCFGAPEVIGKIGTDIEDFKCSWLVVKALELSNEEQKKFLHENYGKEDPASVAKVKELYYTLKLQDVFAEYESKSYEKLIKFIETHPSQAVQAVLKSFLGKIYKRQK
gene-ENSIDEG00000020689	MEISRRLPPKPPPPTPLNESSHRHPYHKRPYSPADRSPSPAPKASDALPLPLYLTNGIFFTLFFSVAYYLLHRWRDKIRSSTPLHVVTLTELAAIVSLIASFIYLLGFFGIDFVQSFISRADVEIDAETDILKADRRPCPKLMDQSLPPPVMISSVEDEEIVNSVVSGKTQSYSLESKLGDCYRAASIRREAVQRTTERSLVGLPLEGFDYESILGQCCEMPIGYVQIPVGIAGPLLLNGCEYLVPMATTEGCLVASANRGCKAIYASGGATGILLKDGMTRAPVVRFATAKRASDLKFFLEDPLNFDTLAIVFNKSSRFGRLQSIQCSMAGKNLYIRFSCSTGDAMGMNMVSKGVQNVLEFLQSDFPDMDVIGISGNFCSDKKPAAVNWIEGRGKSVVCEAIITEDVVKKVLKTTVPALVELNMLKNLAGSAVAGALGGFNAHAANIVSAVFIATGQDPAQNIESSHCITMMEAINAGKDLHISVTMPSIEVGTVGGGTQLASQSACLNLLGVKGANKESPGSNSRLLATIVAGSVLAGELSLMSAIAAGQLVKSHMRYNRSSRDISKIVSKD
gene-ENSIDEG00000020820	MPPSLAMLVENRELLMLLTTSVAVLIGCVVVLVWRRSSRKPAKSLDPPKLVIPKTEPEEEVDDGKKKVTIFFGTQTGTAEGFAKALAEEAKARYEKVTFKVIDLDDYAADDDEYETKLKKESIVFFFLATYGDGEPTDNAARFYKWFSEGKEKGEWLNNLQYGVFGLGNRQYEHFNKIAKVVDDGLAEQGGRRLVPVGMGDDDQCIEDDFTAWRELVWPELDQLLRDEEDTTIATPYTAAILEYRVDFHDQTDTSSLLDRSLSKLNGHSVYDAQHPCRTNVAVKRELHTPASDRSCTHLEFDISSTGLTYETGDHVGVYTENLIEIVEEAERLLDISPDTYFSIHTDKEDGTPLSRGSLPPPFPPCTLRTALTKYADLLSSPKKSALLALAAHASDPSEADRLKFLASPAGKDEYAQWLVANQRSLLEVLAEFPSAKPPLGVFFASVAPRLQPRYYSISSSPRMALSRIHVTCALVYEKTPAGRIHKGVCSTWMKNAVSLEDSLDCSWAPIFIRQSNFKLPADSKVPIIMIGPGTGLAPFRGFLQERLALKEAGAELGPAVLYFGCRNRKLDFIYEDELNNFVETGAMSEMVVAFSREGPTKEYVQHKMSQKASEIWDMISQGAYIYVCGDAKGMAKDVHRMLHTIVQEQGALDSSKAESFVKNLQMSGRYLRDVW

gene-ENSIDEG00000020977	MAFFLDLKMTSYQFILGGAIVSFLGFAMFYLLAKNKKSNNSTNLVQIKRNECAKTVQNGECQQEIVGNPDIIIVGAGVAGSALAYTLGKDGRRVLAVERDLTEPDRIVGELLQPGGYLKLIELGLEDCVNEIDAQQVFGYALFKDGKSTRLSYPLKEFHTDVTGKSFHNGRFIQQMREKAATLSNVKLEQGTVTSLVERKGTVKGVHYKTKSGQDITAYAPLTIVCDGCFSNLRRSLCNPKVEIPSFFVALILKNCQLPYPNHGHVILANPSPILLYRISSTEIRCLVDVPGKKVPSIANGDMAHYLRTLVAPQIPFELHDAFITAIDEGNIKTMANRSMPADPYPTPGALLLGDAFNMRHPLTGGGMTVALSDIVVLRDLLRPLSDLNDAPALCRYLESFYTLRKPVASTINTLAGALYKVFCASPDQARNEMRQACFDYLSLGGIFSNGPVALLSGLNPQPLSLVLHFFSVAIYGVGRLLLPIPSLQRACLGARLISDASSIIFPIIKAEGIKQMFFPRVIPAYYRRPPTY
gene-ENSIDEG00000022401	MCLTLLTRTLSLWLSYPYSPAVTGYDIQIKSESRSHSLGLLSLSISSSSIHHNPHNFYLHLSMREILHVQGGQCGNQIGSKFWEVICDEHGVDPTGRHVTDGASDLQLERINVYYNEASGGRYVPRAVLMDLEPGTMDSIRSGPYGQIFRPDNFVFGQSGAGNNWAKGHYTEGAELIDAVLDVVRKEAENCDCLQGFQVCHSLGGGTGSGMGTLLISKMREEYPDRMMLTFSVFPSPKVSDTVVEPYNATLSVHQLVENADECMVLDNEALYDICFRTLKLSTPSFGDLNHLISATMSGVTCCLRFPGQLNSDLRKLAVNLIPFPRLHFFMVGFAPLTSRGSQQYISLTVPELTQQMWDSKNMMCAADPRHGRYLTASAMFRGKMSTKEVDEQMINVQNKNSSYFVEWIPNNVKSSVCDIPPTGLKMASTFVGNSTSIQEMFRRVSEQFTAMFRRKAFLHWYTGEGMDEMEFTEAESNMNDLVAEYQQYQDATAEDEEEDYGDGEGEEGEQYGD
gene-ENSIDEG00000022420	MAESLKGGSIDLSMPASMAMLFENRELLMLLTTSIAILIGCVVVLVWRRSSSQGSAKSFEPPKLVISKIEPEEEVDDGKKKVTIFFGTQTGTAEGFAKAFAEEAKARYEKAKFKVTDLDDYAEDDDEYEAKLKKESLAFFFLATYGDGEPTDNAARFYKWFSEGEEKDEWLKNLQYGVFGLGNRQYEHFNKIAKVVDDGLAEQGAKRLVPVGMGDDDQCIEDDFTAWRELVWPELDQLLLDKEDAAVAMPYTAAVLEYRVVVHDQTDTSLLDRNLSTLNGHTVYDAQHPCRSNVAVRRELHTPASDRSCIHLEFDISHTGLSYETGDHVGVYCENLIEIVEEAERLLGIAPATYFSVHTDKEDGTPLSGGSLPPSFPPCTLRTALTRYADLLSSPKKSALLALAAHASDPSEADRLRFLASPAGKDEYAHWLLANQRSLLEVMAEFPSANAPLGVFFASVAPRLQPRYYSISSSPRMAPSRIHVTCALVYEKTPTGRIHKGVCSTWMKNAVSLEENNDCSWAPIFVRQSNFKLPSDTKVPIIMIGPGTGLAPFRGFLQERLALKEAGAELGPAVLYFGCRNRKLDFIYEDELNNFVETGAISELVLAFSREGPAKEYVQHKMSQKASEVWNMISQGAYIYVCGDAKGMARDVHRMLHTIAQEQGALDSSKAESLVKNLQMTGRYLRDVW
gene-ENSIDEG00000023236	MEVRARAPGKIILAGEHAVVHGATAVAASIDLYTYASLHFPSPSDNHNTLKLQLKDLDLEFSWTVEKIKDAFRDHGGCNASSPTSCSPETIKIIAALVEEQYIPEAKIGLAAGVTAFLWLYTSIHGYKPAKVIVTSELPLGSGLGSSAAFCVSLSAAFIALSDSVNLDFNHQGWLMFGESELELVNRWAFEGEKLIHGKPSGIDNTVSTFGNMIKFRAGALTRIKSNMQLKMLITNTKVGRNTKALVASVSERTLRHPDAMTAVFTAVDSISNKLTTIIESPASDDCAITEKEVLVEELMEMNQGLLQCMGVSHASIETVIRTTLKYKLATKLTGAGGGGCVLTLLPTILSGKIVDKVIFELESCGFQCLIAGIGGNGLEICFGGSS

gene-ENSIDEG00000024014	MELGRSYMVINDQYFLGGITVATFLFGFVVLFTLKRKREKKGGATSMAINGTYKNNIKMTTSSEVNGHCSLEDIAGSSADVIIVGAGVAGSALAYTLAKDGRRVHVIERDLTEQDRIVGELLQPGGYLKLVELGLEDCVNEIDAQRVFGYALYMDGKNTRLSYPLEKFHADVAGRSFHNGRFIQRMREKAASLPNLRMEQGTVTSLVEQRGTVKGVQYKTKNGQVMSAYAPLTIVCDGCFSNLRRSLCNPKVDVPSCFVGLILENIDLPHINHGHVILADPSPILFYKISSTEIRCLVDVPGQNVPSIANGELAHYLKTSVAPQIPPELYKSFIAAIDKGQIKTMPNRSMPADSHPTPGALLLGDAFNMRHPLTGGGMTVALSDVVLIRDLLRPLHNLHDSSALCKYFESFYTLRKPVASTINTLAGALYKVFCASPDQARQEMRDACFDYLSLGGICSEGPIALLSGLNPRPMSLFFHFFAVAIYGVGRLLIPFPSPKKMWLGARLISGASGIIFPIIKSEGVRQMFFPATVPAYYRAPPLQKKCE
gene-ENSIDEG00000024263	MFIPHYYLRNSHHQLQIKQAYSNTYRYIERERMGSLGAILKHPDDFYPLLKLKIAARHAEKQIPPEPHWAFCYSMLHKVSRSFGLVIQQLGPQLRDAVCIFYLVLRALDTVEDDTSISTEVKVPILIAFHRHIYDNDWHFACGTKEYKVLMDEFHHVSNAFLELGSGYKEAIEDITMRMGAGMAKFICKEVETVDDYDEYCHYVAGLVGLGLSKLFHASGLEDLATDSLSNSMGLFLQKTNIIRDYLEDINEIPKSRMFWPRQIWSKYVDKLEDFKYEVNSGKAVQCLNDMVTNALLHVEDCLKYMSDLRDPGIFRFCAIPQIMAIGTLALCYNNIQVFRGVVKMRRGLTAKVIDRTNTMSDVYGAFFDFSCMLKSKVDNNDPNATKTLSRLEAIQKTCKNSGALTTKRKSYIIENESRYNSTLIVILFIILAILYAYLSSSLPNRL
gene-ENSIDEG00000026974	MNSSSSTLSSSTATLHSFMEAMLIDPYILRWIFAFLSGFILLFNFKRKREKINGANSTEFGTDSINNTSSENGYYRPENIAGSTDVIIVGAGVAGSALAYTLAKDGRRVHVIERDLTEQDRIVGELLQPGGYLKLIELDLEDCVNEIDAQRVFGYALYMDGKNTRLSYPLEKFHADVAGRSFHNGRFVQRMREKAASLPNVRMEQGTVTSLVEKKGTVKGVQYKTKDGQQLSAFAPLTIVCDGCFSNLRRSLCNPKVEVPSSFVGLILENIDLPHINHGHVILADPSPILFYKISSTEIRCLVDVPGQKVPSVSNGELANYLKTVVAPQVPKELYNSFIAAVDKGNIRTMPNRSMPADPHPTPGALLLGDAFNMRHPLTGGGMTIALSDIVLIRDILRPLPDLHDSSTLCKYLESFYTLRKPVASTINTLAGALYKVFCASPDKARQEMRNACFDYLSLGGICSQGPIALLSGLNPRPLSLFLHFFAVAIYGVGRLLIPFPSPTRMWLGARLILGASGIIFPIIKSEGVRQMFFPATVPAYYRAPPVH
gene-ENSIDEG00000027362	MAKLFLICTCWVIDYDHSFIFFSFLVIASLLGFAVFYLLGKMKNNNNYSTLAQAQRKEKCAKTALDGECQEEMVRNLDVIIVGAGVAGSALACTLGKDGRRVLVIERDLTEPDRIVGELLQPGGYLKLIELGLEDCVNEIDAQQVFGYALFKDGKSTRLSYPLKEFHSDVTGKSFHNGRFIQRMREKAATVKLEQGTVTSLVERKGTVKGVHYKTKTGQDMTAYAPLTIVCDGCFSNLRRSLCNPKVEIPSYFVALILKNCQLPYPNHGHVIIANPSPILLYRISSTEIRCLVDVPGKKVPSISNGDMAHHLKTLVASQVYLSFFIDEGNMRTMANRSMPADPYPIPGAFLLGDAFNMRHPLTGGGMTVALSDIVVLRDLLRPLGDLNDASALCRYLESFYTLRKPVASTINTLAGALYKVFCASPDQARNEMRQACFDYLSLGGIFSNGPVALLSGLNPQPLSLVLHFFSVAIYGVGRLLLPFPSAKRAWLGARLISVSNSYFIKKLYSLTN

gene-ENSIDEG00000028100	MDVRRRLTPKPPRPIPITESSHHHCKTPFLAAVDRSPSPTPKASDALPLPLYLTNGIFFTLFFSVAHYLLHRWRDKIRSSTPLHIVTLAELAAIVSLIASFIYLLGFFGIDFVQSFVSRADVDVDIDAEPDILEADRRPCSKLMDQPSPPPLLMSSEEDEEIVKSVVSGKTPSYSLESKLRDCYRAASIRREAVQRTTERSLLGLPLDGFDYDSILGQCCEMPIGYVQIPVGIAGPLLLNGCEYLVPMATTEGCLVASTNRGCKAIYACGGATGILLKDGMTRAPVVRFSSAKRASDLKLFLEDPLNFDTLAIVFDKSSRFARLQSIQCSMAGKNLYIRFCCSTGDAMGMNMVSKGVQNVLEFLRSDFPDMDVIGISGNFCSDKKPAAVNWIEGRGKSVVCEAILTEDVVKKVLKTTVPALVELNMLKNLAGSAVAGALGGFNAHAANIVSAVFIATGQDPAQNIESSHCITMMEAINEGKDLHISVTMPSIEVGTVGGGTQLASQSACLNLLGVKGANKESPGSNSRLLATIVAGSVLAGELSLMSAIAAGQLVRSHMKYNRSSRDMSKIGS
gene-ENSIDEG00000028760	MQPSLAILVQNRELLMLLTTSVAVLIGCVVVLVWRRSSSQKPAKSFEAPTLLIPKIEAEEEVDNGKKKVTIFFGTQTGTAEGFAKALAVEAKARYEKAIFKVVDLDDYAPEDDDYEIKLKKESLAFFFLATYGDGEPTDNAARFYKWFTEGKEKGEWLNNLQYGVFGLGNRQYEHFNKIAKVVDDGLAEQGAKRLLPVGMGDDDQCIEDDFTAWRELVWPELDQLLGDKEDTAAATPYTAAVLEYRVVFHDRTDSSSLLNGTTSVSNGHAFYDAQHPCRAKVVVKRELHTPESDRSCAHLEFDISSTGLAYETGDHVGVYTENLVEIVEEAERLLAISPDTYFSIHTEKEDGSPLGGGSFQPPFPPCTLREALRRYADLLSSPKKSALLALAAHASDPSEADRLRFLASPAGKDEYAQWIVANQRSLLEVLAEFPSAKLPLGVFFASVAPRLQPRYYSISSSPRMAPSRIHVTCALVYERTPAGRIHKGVCSTWMKNAVSSEEGNDCSWAPIFIRRSNFKLPSDSKVPIIMIGPGTGLAPFRGFLQERLALKEAGAELGPAVLYFGCRNRKLDFIYEDELNNFVESGAISEMVVAFSREGPTKEYVQHKMSQKASEIWDMISQGAYIYVCGDAKGMARDVHRKLHTIAQEQGALDSSKAESLVKNLQMTGRYLRDVW
gene-ENSIDEG00000028976	MEEHYVLGSILASLLGFILVYALFAKKNAPRDSVEAAKSTTATTTTVIKGECRSSNGAGDDADVIIVGAGVAGAALAHTLGKDGRRVHVIERDLTEPDRIVGELLQPGGYLKLIELGLEDCVEEIDAQKVFGYALFKDGKSTRLSYPLEKFRSDVSGRSFHNGRFIQRMREKAATLPNVQMEQGTVTSLLEENGTIKGVQYKTKTGEEMSAYAPLTIVCDGCFSNLRRNLCSPKVDVPSCFVGLVLENCELPHANHGHVILADPSPILFYPISSTEIRCLVDVPGQKVPSISNGEMATYLKTMVAPQIPPELHDAFIATVEKGNIRTMPNRSMPAAPHPTPGALLMGDAFNMRHPLTGGGMTVALSDIVVLRNLLRPLRDMNDASTLCKYLESFYTLRKPVASTINTLAGALYKVFCASPDEARKEMREACFDYLSLGGVCSEGPVSLLSGLNPRPLSLVVHFFAVAIFGVGRLLLPFPSPKRMWIGARLISSASGIIFPIIKAEGLRQMFFPATVPAYYRAPPVW

gene-ENSIDEG00000030213	MWRLKIAEGGNDLYLYSTNNYVGRQIWEFDPEYGTPEERAKVEEARCHFWNNRYQVKPSGDLLWRMQFLQEKNFKQTIPQVKVEDGEEITYETATTTLRRAVHFFVALQADDGHWPAENAGPLFFLPPLVMCLYITGHLNTVFPAEHRKEILRYIYCHQNEDGGWGLHIEGHSTMFCTALSYICMRILGEGPDGGENNACARARKWILDHGSVTAIPSWGKTWLSILGIFEWSGTNPMPPEFWILPSYLPMHPAKMWCYCRMVYMPMSYLYGKRFVGPITLLILQLREELYARPYDKINWRKARHNCAKEDLYYPHPLIQDLMWDSLYIFTEPFLTRWPFNKLREKALQTTMKHIHYEDENSRYITIGCVEKVLCMLACWVEDPNGDYFKKHLARIPDYIWVAEDGIKMQSFGSQEWDTGFAIQALLASDLTDEIQPILMKGHDFIKKSQVTENPSGDFNSMHRHISKGSWTFSDQDHGWQVSDCTAEGLKCCLLFSRMPTEIVGKKMEDSRLFDAVNVLLSLQSKNGGLAAWEPAGSSEWLELFNPTEFFADIVIEHEYVECTSSAIQAMVLFKKLYPGHRKKEIEVAISNAVRYLEDIQMPDGSWYGNWGVCFTYGTWFAVGGLTAAGKTYRNCQAICNAVDFLLKSQRSDGGWGESYLSCPNKEYTPLEGNRSNLVHTSWATMALIQSGQGERDPTPLHRAAKLLINSQMESGDFPQQEITGVFMKNCMLHYAAYRNIYPLWALAEYRRNVPFPSKRV
gene-ENSIDEG00000030611	MDVRRRLVKPDLPSAKSATAGEPLKSQHQHPKASDALPLPLYLTNGLFFTMFFSVMYFLLHRWREKIRNSTPLHLVTLSELAALVLLMASAIYLIGFFGIGFVRSVIRPSPEGWDILEDDHAILKEDSRLEPCAAAIECSLPPNPKIVHMLPQKQKQPAPAITTLTEEDEEIVKFVVNGKIPSYSLESKLGDCKKAAAIRREALQRITGKSLAGLPLDGFDYECILGQCCEMPVGYVQIPVGIAGPLLLNETEYSVPMATTEGCLVASTNRGCKAIYASGGATSVLLRDGMTRAPVVRFGTAKRAAELKFFLEDPMNYDTLAHVFNKSSRFGRLQGIKCAVAGKNLYMRFTCSTGDAMGMNMVSKGVQNVLDFLQNDFPDMDVMGISGNYCSDKKPAAVNWIEGRGKSVVCEAIVKEEVVKKVLKTNVAALVELNMLKNLTGSAMAGALGGFNAHASNIVSAVYIATGQDPAQNVESSHCITMMEAVNDGKDLHISVTMPSVEVGTVGGGTQLASQSACLNLLGVKGASKESPGSNSRLLASIVAGSVLAGELSLMSALAAGQLVKSHMKYNRSSKDITKLSS
gene-ENSIDEG00000030937	MWKLKIAEGGNPWLRSLNDHVGRQTWEFDPKVASPEELEKIEKVRETFRNHRFEKKHSSDLLMRIQFANENRGSILLPQVKVKDTEDISDNKVTVTLKRAISFHSTLQAQDGHWPGDYGGPMFLMPGLVITLSITGALNAVLSKEHKREICRYLYNHQNRDGGWGLHIEGPSTMFGTALNYVTLRLLGEGANDGQGAMEKGRQWILDHGGATAITSWGKMWLSVLGVFEWSGNNPLPPEIWLLPYILPFQPGRMWCHCRMVYLPMSYLYGKRFVGPITSTVLSLRKELFTVPYHEIDWNQARSLCAKEDLYYPHPLIQDILWASIDKVLEPIFMRWPGKKLREKSLRTVMEHIHYEDENTRYICIGPVNKVLNMLCCWSEDPNSEAFKLHLPRLNDFLWLAEDGMKMQGYNGSQLWDSAFAVQAIISTELTDEFGPTLRKAHMFIKNSQVLDDCPGNLDFWYRHISKGAWPFSTADHGWPISDCTAEGFKAVLLLSKLPSELVGEPLDAKRLYDTVNVILSLQNSDGGYATYELTRSYSWLELINPAETFGDIVIDYPYVECTSAAIQALSAFKKLYPDHRREEIQLSIEKAALFIEKIQASDGSWYGSWGVCFTYGTWFGVKGLIAAGRTFSSCSSIRKACDFLLSKQLASGGWGESYLSCQNKVYTNLEGNRSHVVNTGWALLALIDAGQAKRDPAPLHRAARLLINSQMENGDFPQQEITGVFNKNCMITYAAYRNIFPIWALGEYRCRVLR

gene-ENSIDEG00000031109	MKSPSLSDLMATTLVENQELLMLLTTSVAILIGCVVVLVWRRSSSQRSAKSLEPAKLVIPKIKPEEEVDDGKKKVTIFFGTQTGTAEGFAKAFSEEAKARYEKTKFKVIDLDDYAADDDEYESKLKKESFAFFFLATYGDGEPTDNAARFYKWFSEGKENREWLSNLQYGVFGLGNRQYEHFNKIAKVVDDGLAEQGAKRLVQVGMGDDDQCIEDDFTAWRELVWPELDKLLLDDEAAVATPYTAAVLEYRVVVHDRTDTPLLDRSLSMQNGHAVFDAQHPCRSNVAVKRELHTPVSDRSCIHLEFDISQTGLAYETGDHVGVYCENLIETVEEAEKLLGISPATYFSIHTDKEDGTPLSGGSLPPPFPPCTLRTALTRYADLLSSPKKSALLALAAHASDPSEADRLRFLASPAGKDEYAQWLVANQRSLLEVLAEFPSAKSPLGVFFASVAPRLLPRYYSISSSPRMAPSRIHVTCALVYEKTPTGRIHKGVCSTWMKNAVSLEESHDCSWTPIFVRQSNFKLPSDTKVPIIMIGPGTGLAPFRGFLQERLALKDAGAEMGPAVLYFGCRNRKLDFIYEDELNNFVDSGAISELVVAFSREGPTKEYVQHKMSQKASEVWNMISEGAYIYVCGDAKGMARDVHRMLHTIAQEQGVLDSSKAESLVKNLQMTGRYLRDVW
gene-ENSIDEG00000031356	MDLSMAMLFENRELLMLLTTSIAILIGCVVVLVWRRSSSQGSAKSLEPPKLVISKIEPEEEVDDGKKKVTIFFGTQTGTAEGFAKAFAEEAKARYEKAKFKVIDLDDYAEDDDEYEAKLKKESLAFFFLATYGDGEPTDNAARFYKWFSEGEEKDEWLKNLQYGVFGLGNRQYEHFNKIAKVVDDGLAEQGAKRLVPVGMGDDDQCIEDDFTAWRELVWPELDQLLLDEDAAVATPYTAAVLEYRVVVHDQIDTSLLDRNLSTLNGHTVYDAQHPCRSNVAVKRELHTPASDRSCIHLEFDISHTGLSYETGDHVGVYCENLIEIVEEAERLLGIASATYFSVHTDKEDGTPLSGGSLPPPFPPCTLRTALTRYADLLSSPKKSALLALAAHASDPSEADRLRFLASPAGKDEYAQWLVANQRSLLEVMAEFPSAKPPLGVFFASVAPRLQPRYYSISSSPRMAPSRIHVTCALVYEKTPTGRIHKGVCSTWMKNAVSLEENNDCCWAPIFVRQSNFKLPSDTKVPIIMIGPGTGLAPFRGFLQERLALKEAGAELGSAVFYFGCRNRKLDLIYEDELNNFVETGAISELVLAFSREGPTKEYVQHKMSQKASEVWNMISQGAYIYVCGDAKGMARDVHRMLHTIAQEQGALDSSKAESLVKNLQMTGRYLRDVW
gene-ENSIDEG00000032600	MENSWIVLVAAAIFFSSSVAILILFQNYGRSSSSSSKYRKSSSRSSVLPLGTMGWPFLGETIEFISCAYTDHPESFMDKRRRMYGKVFKSHIFGSATIVSTDEEVSRCILQSDSNTFVPSYPKSLTVLMGKSSILLINGYLQRRIHGLIGSFLKSPHLKAQITRDMQKYVQQSMQTWKEDRIIYIQDQTKKIAFQVLVKALISLDPGEEMEFLRKQFQEFIAGLMSLPINIPGSRLYRSLQAKKKMVKLVHKIIEAKKKNESRSRVQKDVADVLLSENLGDDIISDNMIDLMIPGEDSVPVLITLAIKYLSDSPAALHQLTEENMKLKKLKDEVGEQLCWSDYLSLPFTQTVITESLRMGNIINGVMRKAMKDVKIKGGYVIPKGWCVFTYFRSVHLDQNLYDSPYQFNPWRWQDRETSGCSFSPFGGGQRLCPGLDLARLEASIFIHHFVTQFRWVAEDDSIVNFPTVRMRNRLPIWVKRRERPLSILF
gene-ENSIDEG00000032972	MNPKNVGILAMEIYFPPTCIQQEVLESHDGASKGKYTIGLGQECMGFCTELEDVISMSLTAVTSLLDKFGIDRKQIGRLEVGSETVIDKSKSIKTFLMQIFEDCGNTDIEGVDSTNACYGGTAALFNCVNWVESSSWDGRYGLVVCTDSAVYAEGPARPTGGAAAIVMLIGPDAPIAFESKFRGSHMSHAYDFYKPNLASEYPVVDGKLSQTCYLMALDSCYKRFCTKFEKWEGKPFSISEADYYVFHSPYNKLVQKSFARLCYDEFLRNPSFVDEATREKLAPFSALSGDESYQSRDLEKATQQVAKQIYEEKVQPTTLIPKQVGNMYTASLYAAFASLLHNKHSSLAGKRVIMFSYGSGLTATMFSFQLRECEHPFSLSNIATVMNVSEKLKSRHEFSAEKFIETMKLMEHRYGTKDFVTSKDTSLLSPGTCYLTEVDSMFRRFYERKSVDITTSIE

gene-ENSIDEG00000033049	MPPSLAKLFENRELLMLLTTSVAVLIGCVVVLMWRRSSQKPAKSLEPPKLVIPRTEPEEEVDDGKKKVTIFFGTQTGTAEGFAKALAEEAKARYEKATFNVIDLDDYAADDDEYETKLKKESITFFFLATYGDGEPTDNAARFYKWFSEGKEKGEWLNNLQYGVFGLGNRQYEHFNKIAKVVDDGLAEQGGKRLVPVGMGDDDQCIEDDFTAWRELVWPELDQLLRDEEDTTIATPYTAAVLEYRVEFHDRTDTSSLLDWSLSKLNGHAVYDAQHPCRANVAVKRELHTPASDRSCTHLEFDISSTGLIYETGDHVGVYTENLIEIVKEAERLLNISPDTYFSVHTDKEDGTPLSGGSLPPPFPPCTLRTALTKYADLLSSPKKSALLALAAHASDPSEADRLKFLASPAGKDEYAQWLVANQRSLLEVLAEFPSAKPPLGVLFASVAPRLQPRYYSISSSPRMAPSRIHVTCALVYEKTPAGRIHKGVCSTWMKNAASLDESHDCSWAPIFVRQSNFKLPADSKVPIIMIGPGTGLAPFRGFLQERLALKEAGAELGPAVLYFGCRNRKLDFIYEDELNNFIETGAMSEMVVAFSREGPTKEYVQHKMSQKALEIWDMISQGAYIYVCGDAKGMARDVHRMLHTIVQEQGALDSSKAESFVKNLQMSGRYLRDVW
gene-ENSIDEG00000033089	MEISRRLPTKPSPPHRQPQRHPLPLYLTNGIFFTLFFSVAYYLLHRWRDKIRSSTPLHVVTLTELAAIVSLIASFIYLLGFFGIDFVQSFISRADVEIDAETDILEADRRPCPKLMDHTSRPPIMISSAEDEEIVNSVVSGKTPSYSLESKLGDCYRAASIRREAVERTMERSLVGLPLEGFDYESILGQCCEMPIGYVQIPVGIAGPLLLNGCEYLVPMATTEGCLVASTNRGCKAIYASGGATGILLKDGMTRAPVVRFATAKRASDLKFFLEDPLNFDTLAVVFNKSSRFGRLQSIQCSMAGKNLYIRFSCSTGDAMGMNMVSKGVQNVLEFLQSDFPDMDVIGISGNFCSDKKPAAVNWIEGRGKSVVCEAIITEDVVKKVLKTTVPALVELNMLKNLAGSAVAGALGGFNAHAANIVSAVFIATGQDPAQNIESSHCITMMEAINDGKDLHISVTMPSIEVGTVGGGTQLASQSACLNLLGVKGANKQSPGSNSRLLATIVAGSVLAGELSLMSAIAAGQLVKSHMKYNRSSRDISKIVCKV
gene-ENSIDEG00000033704	MGIMMDTYYYIAGTLFASLLGFSFLYILGHHNSTIANSKKKNQQQKTTAARSVVTRNDPNNPRGNGSDADIIIVGAGVAGAALAHTLGKDEQRVLVIERDLTEPDRIVGELLQPGGYLKLIELGLEDCVEEIDAQRVIGYALFKDGKNAKLSYPLEKFHSDVSGRSFHNGRFIQRMREKAATLPNVRLEQGTVSSLLEENGTIKGVQYKTKTGQKVKAYAPLTIVCDGCFSNLRRSLCKPNVDVPSCFVGLILENCKLPHPNHGHVILADPSPILFYPISSTEVRCLVDIPGQKLPSLANGEMATYLKTIVAPQIPPELHDAFIAAIKKGNIRTMPNRSMPAAPYPTPGALLMGDAFNMRHPLTGGGMTVALSDIVVLRNLLKPLRDMNDADSLCKYLESFYTLRKPVASTINTLAGALYKVFCASPDQARKEMREACFDYLSLGGVCSTGPMALLSGLNPQPLSLVLHFFAVAIFGVGRLLLPFPSPKRLWNGVRLISVASGIIFPIIQAEGVRKMFFPATVPAYYRAPPVDVIKF

gene-ENSIDEG00000033798	MWKLKIAEGDGCWLTSTNNHVGRQHWEFDPQAGTPEERAQVQTFRQQFKTNRFQIKQSSDLLMRMQFAKENKTRREMPVAVKVKETEEVTEEAATTTVRRAINFYSTLQGHDGHWPAESAGPLFFLPPLVIALYVTGALSAILSPPHQREIIRYIYNHQNEDGGWGLHIEGHSTVFGSTLNYIALRLLGEGTEDGEDMAMARARKWILDHGGALGIPSWGKVLASSQILSHSSRLINFTIATIRLAYLLNERSLYIILYLIYDLQYCIELMNEIAGKMLCYCRLVYMPMSYLYGKRFVGRITGLVRSLRQELYIQPYHEINWNKARSTCAKEDLYYPHPLVQDVLWGFLHNIGEPILTRWPFNTFRKKALKVAMEHVHYEDQSSRYLCIGCVEKVLCLLACWVEDPNSEAYKRHLARIPDYLWLAEDGMKMQSFGSQLWDAAFAVQAILSSNLAVEYGSTLQKAHDFIKASQVQDDPPGNFHEMYRHTSKGAWTFSMQDHGWQVSDCTAEGLKAALLLSQNPKLIGERIETQRLHDSKNGGFPAWEPQRAYSWIEKFNPTEFFEDVLIEREYVECTSSAIQALTLFTKLHPGHRRKEIKSCISKAIQYVEDTQNPDGSWYGCWGVCFTNGTWFAVEALVACGMKIQNSPTLSKACEFLLSKQLPDGGWGESYLSSSNKVYTNLKGNRSNLVQTSWALLSLIKAGQADIDPTPIHRGVRALINSQMEDGDFPQQELTGAFMKNCTLNYSSYRNIFPIWALGEYRQHSLLKMDDS
gene-ENSIDEG00000035336	MENSWIVLVTAAIFIFSAAILVLFQNYGRSSSSSSKYRKSSSRSSVLPLGTMGWPFLGETIEFISCAYTDHPESFMDKRRRMYGKVFKSHIFGSATIVSTDEEVSRCILQSDSNTFVPFYPKSLTVLMGKSSILLINGYLQRRIHGLIGSFLKSPDLKAQITRDMQKYVQQSMQTWKEDRIIYIQDQTKKIAFQVLVKALISLDPGEEMEFLRKQFQEFIAGLMSLPINIPGSRLYRSLQAKKKMVKLVHKIIEAKKKNESSSRVQKDVADVLLNEKLGDDIISDNMIDLMIPGEDSVPVLVTLAIKYLSDSPVALHQLTEENMKLKKLKDEVGEQLCWSDYLSLPFTQTVITESLRMGNIINGVMRKAMKDVKIKGGYVIPKGWCVFTYFRSVHLDQNLYDSPYQFNPWRWQDRETSGCSFSPFGGGQRLCPGLDLARLEVSIFIHHFVTQFRWVAEHDSIVNFPTVRMKNRLPVWVKRRRDP
gene-ENSIDEG00000035347	MMMAWTWAWSFSSIGLLLSGITITMWWCFYININTQNTLMRRRRRKYWNNLEEQNTSSNIPRGSSGWPFIGETLDFIASGYSSRPVSFMDKRKSLYGKVFKTHILGRPIIVSTDPDVNRTVLQNHGNVFIPCYPKSVTELLGKSSILHMNGNLQKRLHGLIGGFLRSPQLKSRIARDIENSVKLSLSNWNRKKQPIYLQDETKRITFEVLVRVLTSVGPGEDMDFLKREFEEFIKGLICIPIKFPGTRLYKSLEAKKRLLKMVTKIVEERKLGMDKTGEKGSPNDAIDVLLRDTGESSHETQQQRLPLDFISGNIIEMMIPGEDSVPMVMTLAVKYLADSPVALSRVVEENRALKRHKAESTGGYAWTDYMSLQFTQNVISETLRMANIINAVWRKALKDVEIKGYLIPKDWCVMASFSSVHMDAENYENPYQFNPWRWEKTGGGVNNSNTFTPFGGGQRLCPGLELSRLEISIFLHHFVTTYRWVVEADEIVTFPTVKMKRKLPITIMPMVQQHY
gene-ENSIDEG00000036201	MDVRRRQVTKRATAGEPLKSQNQQSSDALPLPLNLTNGLFFTMFFSVMYFLLHRWREKIRNSVPLHVVTLSELAALVSLVASVIYLLGFFGIGFVESVIRSSLDSWDILEDDNAILEEDSRPEPCAAAIDCSLPSNPKIVHMVPQKPKSAFADMVVEQKQSASATITQEEDEEIIKSVVAGTTPSYSLESKLRDCLKAVAIRREALQRITGKSLAGLPLDGFDYESILGQCCEMPVGYVQIPVGIAGPLLLNETEYSVPMATTEGCLVASTNRGCKAIYASGGATSVLLRDGMTRAPVVRFGTAKRAAELKFFLEDPMNFETLALVFNKSSRFGRLQGIKCAIAGKNLYMRFTCSTGDAMGMNMVSKGVQNVLDFLQNDFPDMDVMGISGNYCSDKKAAAVNWIEGRGKSVVCEAIIKEEVVKKVLKTNVAALVELNMLKNLTGSAVAGALGGFNAQAGNIVSAVYIATGQDPAQNIESSHCITMMEAVNDGKDLHISVTMPSIEVGTVGGGTQLASQSACLSLLGVKGASKESPGSNSRLLASIVAGSVLAGELSLMSAIAAGQLVTSHMKYNRSSKDVSKLSS

gene-ENSIDEG00000036465	MDVRRRTVKPLPSAKSATSGEPLKSQHQHPKASDALPLPLYLTNGLFFTM<br>FFSVTYFLLHRWREKIRNSTPLHVVTLSELAALVLLMASVIYLIGFFGIAFVR<br>SVIRPSPEGWDILEDDNAIPEEDTRREPCVAAIESFSDASAITTLTEEDEEIV<br>KSVVNGKIPSYSLESKVGDCQKAAAIRREALQRITGKSLAGLPLDGFDYESI<br>LGQCCEMPVGYVQIPVGIAGPLLLNETEYSVPMATTEGCLVASTNRGCKA<br>IYASGGATSVLLRDGMTRAPVVRFGTAKRAAELKFFLEDPMNYDTLAHV<br>FNKSSRFGRLQGIKCAVAGKNLYMRFTCSTGDAMGMNMVSKGVQNVL<br>DFLHNDFPDMDVMGISGTSCNCKGGSSEEGVKDQCGSLGGAEHAQKSN<br>RLSCGRHRSGPCSKCGEICNLFLLFFKKNTNFFLMNLSGGDSRWWDPTSIP<br>VSLPEPTRSEGCKQRVTRVKLKALGQHSSWFSPGCRAAFH
gene-ENSIDEG00000036537	MAPAAATDCSESIKARDVCIVGAARTPMGGFLGTLSSLSATKLGYIAIQSA<br>LKRANVDPALVQEVFFGNVLSANLGQAPARQAALGAGIPNSVVCTTINK<br>VCASGMKATMLAAQSIQLGINDIVVAGGMESMSNVPKYLAEARKGSRLG<br>HDSLVDGMLKDGLWDVYNDYGMGVCAEICAEQHGVSRVEQDNYAIQS<br>FERGIAAQNSGAFAWEIVPVEVSGGRGKPSTIVDKDEGLGKFDVAKLRKL<br>RPSFKETGGTVTAGNASSISDGGAALVLVSGETAVKLGLQVLAKISGYGD<br>AAQSSELFTTSPALAIPKAISSAGIEASQVDFYEINEAFAVVALANQKLLGL<br>NPEKVNRHGGAVSLGHPLGCSGARILVTLLGVLRQNKGKYGVGGVCNG<br>GGGASAVVVELV
gene-ENSIDEG00000036903	MWRLKIAEGGNDPYLYSTNNYVGRQVWEFDPEYGTPEERAKVEEARLHF<br>WNNRYQVKPSGDLLWRMQFLQEKNFKQTVPQVKVEDGEEITYETASTTL<br>RRAVHFFAALQADDGHWPAENAGPLFFLPPLVNLLEQSINYHSCLFQNK<br>KFQVMCLYITGHLNTVFPAEHRKEILRYIYCHQNEDGGWGLHIEGHSTM<br>FCTALSYICMRILGEGLDDGENNACARSRKWILDHGSVTAIPSWGKTWLS<br>ILGVFDWSGTNPMPPEFWILPPFLPMHPAKMWCYCRMVYMPMSYLYGK<br>RFVGPITPLILQLREELYAQPYDKINWRKARHNCAKEDLYYPHPLIQDLM<br>WDSLYIFTEPFLTHWPFNKLREKALQTTMKHIHYEDENSRYITIGCVEKVL<br>CMLACWVEDPNGDYFKKHLARIPDYIWVAEDGMKMQSFGSQEWDTGF<br>AIQALLASDLTDEIQPILMKGHDFIKKSQVTENPSGDFKSMHRHISKGSWT<br>FSDQDHGWQVSDCTAEGLKCCLLFSRMPTEIVGNKMEDSQLFDAVNVLL<br>SLQSKNGGLAAWEPAGSSEWLELLNPTEFFADIVIEHEYVECTSSAIQAMV<br>LFKKLYPGHRKKEIEVAISNAVRYLEDIQMPDGSWYGNWGVCFTYGTWF<br>AVGGLTAAGKTHNNCQSICKAVDFLLKSQRSDGGWGESYLSCPNKEYTP<br>LEGNRSNLVHTSWATMALIHSGQAERDPTPLHRAAKLLINSQMESGDFP<br>QQEITGVFMKNCMLHYAAYRNIYPLWALAEYRRNVSLPSKSVSTLTTAA
gene-ENSIDEG00000039246	MADELHKWVVMVTAQTPTNIAVIKYWGKRDETLILPINDSISVTLDPDHL<br>CTTTTVAVSPSFEQDRMWLNGMEISLLGGRFQSCLREIRSRARDLEDEKKG<br>IKIKKMDWEKLHLHIASYNNFPTAAGLASSAAGLACFVFALAKLMNLKE<br>DNGQLSAIARRGSGSACRSLYGGFVKWIMGKEENGSDSIAVQLVDEKHW<br>DDLVIVIAVVSARQKETSSTTGMQDSCKTSMLIQHRAKEVVPKRIIQMED<br>AIEKRDFPSFARLGCADSNQFHAVCLDTSPPIFYMNDTSHKIISCVEKWNR<br>SEGTPQVAYTFDAGPNAVLIARNRKAAALLLRRLLFHFPPHSNTDLNNY<br>VIGDKSILQDVGIQDLKDVESLPPPPEIKDNIPAQKSKGDVSYFICTRPGRG<br>PVVLPDSQALLNPETGFPK

gene-ENSIDEG00000040554	MRSLGEILKHPDDFYPLLKLKFAARHAEKQIPPEPHWAFCYSMLHKVSRS<br>FGLVIQQLGPQLRDAVCIFYLVLRALDTVEDDTSIPTEVKVPILMAFHRHIY<br>DKDWHFSCGTKEYKVLMDEFHHVSNAFLELGSGYQEAIEDITMRMGAG<br>MAKFICKEVETIDDYDEYCHYVAGLVGLGLSKLFHASGAEDLATDSVSNS<br>MGLFLQKTNIIRDYLEDINEIPKSRMFWPRQIWSKYVDKLEDLKYEENSAK<br>AVQCLNDMVTNALLHAQDCLKYMSDLRDPAIFRFCAIPQIMAIGTLALC<br>FNNIQVFRGVVKMRRGLTAKVIDRTKTMSDVYGAFFDFSCLLKSKVDNN<br>DPNATKTLSRLEAIQKTCKESGTLSKRKSYIIESESGHNSALIAIIFIILAILYA<br>YLSSNLPNNQ

Table S6 The FPKM value of HhNACs and triterpenoid saponin biosynthetic genes.

Name	A0-1	A0-2	A0-3	A6-1	A6-2	A6-3	A12-1	A12-2	A12-3
HhNAC2	5.24	4.23	3.92	1.81	2.96	3.17	3.98	4.71	3.50
HhNAC38	4.77	5.94	5.53	12.35	11.40	13.20	3.41	3.38	4.07
HhNAC11	9.11	9.21	9.45	25.90	26.44	30.85	7.39	6.93	8.06
HhNAC110	25.45	24.95	22.74	14.51	12.34	9.72	9.89	9.42	9.30
HhNAC181	3.10	3.25	2.26	6.44	10.19	7.36	5.01	3.96	5.44
HhNAC132	3.31	2.71	2.49	1.59	1.40	1.94	0.88	1.14	1.07
HhNAC78	0.30	0.00	0.35	1.99	2.48	2.09	0.44	0.38	0.55
HhNAC53	1.21	1.39	1.11	2.52	3.27	2.99	0.87	1.04	1.25
HhNAC47	34.71	32.81	35.95	16.61	11.98	14.23	17.09	21.13	21.0
HhNAC104	8.60	4.99	7.20	1.49	1.94	1.50	11.30	12.76	13.2
HhNAC91	3.06	1.90	1.51	3.56	4.46	5.32	4.33	3.91	5.00
HhNAC93	13.13	16.89	14.02	30.26	28.31	29.26	6.30	7.30	5.59
HhNAC67	0.96	1.01	1.22	7.19	6.01	6.35	0.54	1.05	1.10
HhNAC136	5.25	5.10	5.74	11.04	8.93	7.31	6.65	6.63	7.79
HhNAC108	20.91	20.01	18.90	12.67	12.70	13.72	14.17	14.85	12.5
HhNAC164	0.56	0.90	0.66	2.73	1.98	2.34	0.45	2.04	0.96
HhNAC161	1.95	2.01	2.17	0.77	1.01	0.36	0.48	1.15	1.09
HhNAC61	0.17	0.34	0.26	1.27	1.25	0.64	0.16	0.88	0.00
HhNAC140	43.66	42.06	39.34	29.28	28.15	25.49	33.02	34.36	35.47
HhNAC25	2.33	2.77	3.18	3.77	5.93	6.28	3.84	5.03	3.47
HhNAC86	1.81	1.36	1.95	0.52	1.00	0.23	0.90	0.71	0.99
HhNAC178	5.07	5.01	4.44	0.40	0.40	0.54	2.78	2.99	2.22
HhNAC125	3.33	3.16	3.68	6.58	6.88	7.86	3.91	4.77	4.60
HhNAC66	0.47	0.12	0.20	1.26	1.01	1.55	0.00	0.07	0.12
gene-ENSIDEG00000006287	64.41	68.37	68.96	43.32	41.91	39.13	59.41	60.92	62.86
gene-ENSIDEG00000022401	38.63	46.17	37.66	39.51	38.49	37.52	27.12	28.17	28.15
gene-ENSIDEG00000036537	8.69	11.25	8.32	15.32	18.92	16.54	9.59	8.09	9.62
gene-ENSIDEG00000032972	0.29	0.00	0.22	0.31	0.32	0.13	0.45	0.39	0.22
gene-ENSIDEG00000005830	5.01	4.94	3.83	10.12	10.84	10.18	6.29	5.38	6.00
gene-ENSIDEG00000002084	1.00	0.78	1.14	1.55	1.21	1.18	1.38	1.41	1.82
gene-ENSIDEG00000000716	15.17	14.11	13.52	18.56	18.48	16.74	16.14	14.58	16.45
gene-ENSIDEG00000007770	0.52	0.30	0.23	1.82	1.09	1.39	0.54	0.14	0.15
gene-ENSIDEG00000033089	3.74	4.50	3.67	12.05	11.10	12.44	4.23	4.14	4.93
gene-ENSIDEG00000020689	6.74	7.23	6.45	4.08	4.17	3.88	5.67	5.08	5.43
gene-ENSIDEG00000030611	0.03	0.00	0.38	0.00	0.22	0.99	0.00	0.25	0.00
gene-ENSIDEG00000018481	1.29	0.78	0.99	2.12	2.10	1.76	1.66	1.07	1.22
gene-ENSIDEG00000028100	6.04	6.02	6.17	2.26	1.58	2.36	1.05	0.74	0.62
gene-ENSIDEG00000036465	1.04	0.88	0.99	1.96	2.01	2.01	9.39	9.84	10.65
gene-ENSIDEG00000036201	0.05	0.18	0.14	0.07	0.36	0.32	0.26	0.44	0.16

gene-ENSIDEG00000014823	1.81	1.36	2.19	5.53	4.45	4.00	1.89	1.71	1.45
gene-ENSIDEG00000023236	6.36	5.70	4.93	8.60	9.54	7.97	7.61	8.04	7.34
gene-ENSIDEG00000002405	2.93	3.01	3.26	4.13	4.46	2.73	4.97	4.44	2.36
gene-ENSIDEG00000008611	5.58	5.05	4.45	7.27	10.78	7.97	7.52	6.75	7.69
gene-ENSIDEG00000039246	17.28	16.95	15.91	14.01	18.78	18.10	23.67	22.82	23.77
gene-ENSIDEG00000008720	5.70	5.58	5.20	9.44	9.90	7.63	5.01	4.50	5.42
gene-ENSIDEG00000040554	14.96	16.02	16.58	17.59	21.06	19.10	16.43	18.26	16.79
gene-ENSIDEG00000008386	18.23	16.85	19.43	26.79	35.70	30.03	15.95	17.18	19.24
gene-ENSIDEG00000024263	10.12	10.93	10.02	5.31	5.42	6.13	4.90	5.64	5.97
gene-ENSIDEG00000019086	6.90	6.89	7.29	19.78	21.40	20.09	7.70	7.59	7.07
gene-ENSIDEG00000013564	11.07	7.43	7.43	13.09	14.39	10.91	10.33	8.34	8.28
gene-ENSIDEG00000033704	5.90	4.82	4.40	4.21	4.38	3.78	7.68	5.79	7.78
gene-ENSIDEG00000027362	0.10	0.00	0.40	0.00	0.33	0.80	0.00	0.18	0.69
gene-ENSIDEG00000020977	0.00	0.17	0.00	0.42	0.35	0.00	0.71	0.00	0.62
gene-ENSIDEG00000024014	0.15	0.00	0.40	0.17	0.00	0.12	0.00	0.00	0.02
gene-ENSIDEG00000012525	0.50	0.35	0.72	6.56	5.50	4.10	0.48	0.84	0.40
gene-ENSIDEG00000026974	14.58	16.86	14.92	14.46	12.97	15.73	18.94	20.65	18.38
gene-ENSIDEG00000028976	4.40	3.88	3.54	2.52	2.78	2.24	0.97	0.72	1.28
gene-ENSIDEG00000017676	7.67	7.17	7.65	1.44	1.20	1.18	5.72	5.05	5.88
gene-ENSIDEG00000007403	1.44	1.14	1.01	3.03	2.02	3.07	1.68	1.20	1.45
gene-ENSIDEG00000006820	0.39	0.71	0.79	0.00	0.27	0.81	0.11	0.00	0.02
gene-ENSIDEG00000005299	0.17	0.22	0.10	0.18	0.36	0.42	0.27	0.18	0.07
gene-ENSIDEG00000017952	0.32	0.27	0.38	0.62	0.95	0.57	0.87	1.14	1.35
gene-ENSIDEG00000030937	2.68	2.04	2.16	6.69	6.52	7.73	5.22	5.08	5.24
gene-ENSIDEG00000005981	5.27	4.62	4.83	11.04	10.51	9.04	2.49	2.06	2.57
gene-ENSIDEG00000014201	0.16	0.71	0.18	0.00	0.48	0.72	0.20	0.00	0.92
gene-ENSIDEG00000033798	0.40	0.72	0.52	0.69	0.62	0.68	0.20	0.40	0.62
gene-ENSIDEG00000036903	0.06	0.00	0.03	0.00	0.07	0.05	0.22	0.09	0.01
gene-ENSIDEG00000000378	8.36	7.28	7.52	9.58	8.65	9.27	8.57	9.93	9.86
gene-ENSIDEG00000003422	26.86	26.30	25.43	24.33	26.32	21.57	12.00	13.79	11.15
gene-ENSIDEG00000030213	0.07	0.14	0.18	0.63	0.43	0.49	0.21	0.35	0.15
gene-ENSIDEG00000033049	39.87	36.82	38.80	30.26	30.59	32.47	24.35	26.53	24.51
gene-ENSIDEG00000020820	67.20	67.16	69.67	76.58	76.85	77.72	66.45	71.89	70.56
gene-ENSIDEG00000032600	0.54	0.36	0.51	0.73	0.79	0.45	0.62	0.47	0.61
gene-ENSIDEG00000031109	7.13	6.74	6.75	6.97	5.74	6.16	7.80	8.42	8.04
gene-ENSIDEG00000031356	28.75	24.29	28.31	20.54	22.55	23.53	27.34	29.11	29.05
gene-ENSIDEG00000022420	21.26	18.23	21.53	19.32	21.45	21.29	27.15	27.69	29.48
gene-ENSIDEG00000035347	24.71	23.37	22.98	17.24	20.92	21.48	18.11	18.45	18.82
gene-ENSIDEG00000017517	12.34	13.08	15.78	19.00	20.05	18.14	11.29	11.52	13.31
gene-ENSIDEG00000028760	2.93	2.35	3.21	3.36	2.64	3.67	2.39	3.68	2.59
gene-ENSIDEG00000035336	0.32	0.06	0.39	0.59	0.16	0.26	0.08	0.18	0.25

Table S7 Primers used for quantitative real-time PCR.

ID	Forward primer（5′→3′）	Reverse primer（5′→3′）
HhNAC2	CAGTTATGCTACCTTCCCAG	TTTGACTTGCCAGAGTCATGC
HhNAC38	AGTTCGATGATGTGATGGAGTC	ATTTCTGAGTCCACTCTCTACC
HhNAC11	CATGTCTGGCCAAATAGCTACA	TACCTTTTGGAAGATCCCAAGG
HhNAC110	ATACGCACCCTTGCAAACATTTC	ATTCCGCCTTTTCGGGTAATTC
HhNAC181	CATCTACAACACTGCCTCTTC	TCCACTCCCACAGTTTATCC
HhNAC132	GAGACTGAACAAGCTCCAATGG	GACCAGCCATAACCTTCATGT
HhNAC78	AGTGATCCGAAAGAGATGAGG	TCCATCACATTATCACTGGTAGG
HhNAC53	GTTTTATCTTCGCCGGAAGG	GCACTTCCGCAATAGTAAACC
HhNAC47	TTGGTGAGAAACGGGAATTTGG	AGGATGAGCTTTGGACTTGG
HhNAC104	AAATCCTTCCTTCCCAAACGTG	TTTGAGCTGAAATCCCCATGG
HhNAC91	AAAGGGATCCGAACAAGTTGG	TAGAGCCAGTGTTGTAGATGG
HhNAC93	CTCTTGTTTTCTACGAAGGCAG	GACGGTCTGCATTGAAATTGG
HhNAC67	CCATTAATGTTACACCTGCTGC	TCACAACTGGGCATAATCACC
HhNAC136	TGGTGGAAGCAATGTCTTTGTG	TTGCGATCCAAACTCAAGTTGG
HhNAC108	TGATCCTTGGGATTTGCCAG	AATCAGTCCTGGAACCATGG
HhNAC164	CGGAAATGAAATACGCACACC	TTGGCGCCGTTATTAGGTTTTG
HhNAC161	TAGGAAAGATACGCACCCAC	ACGACAGAGTACAAAGGCAC
HhNAC61	GAATTCAATACCTTCCCAACCCTG	GATCGTTCGGATCAAACTTCAC
HhNAC140	AAGAGCTCCGAGTCAAGTATTC	GCTGATGGTATGAACTAAGCAC
HhNAC25	ATACGCACTGGAGAACTTCC	GATCCAACTACTTCGGATCC
HhNAC86	AAAGATTGGGGAGCTTCCTTC	GTTGAAGATGTCTCCTTGAGC
HhNAC178	TGGAGTGGTTTTTCTTCTGTCC	CATAACCGTGCGTATCAATCTG
HhNAC125	AGAGACATTGGAGCTGCACT	ACTTGCGATCTCGAAAGCTG
HhNAC66	CAAGTGCAAGACTTTCCTCAG	GCCGTGCGTATTTGAATTTGG

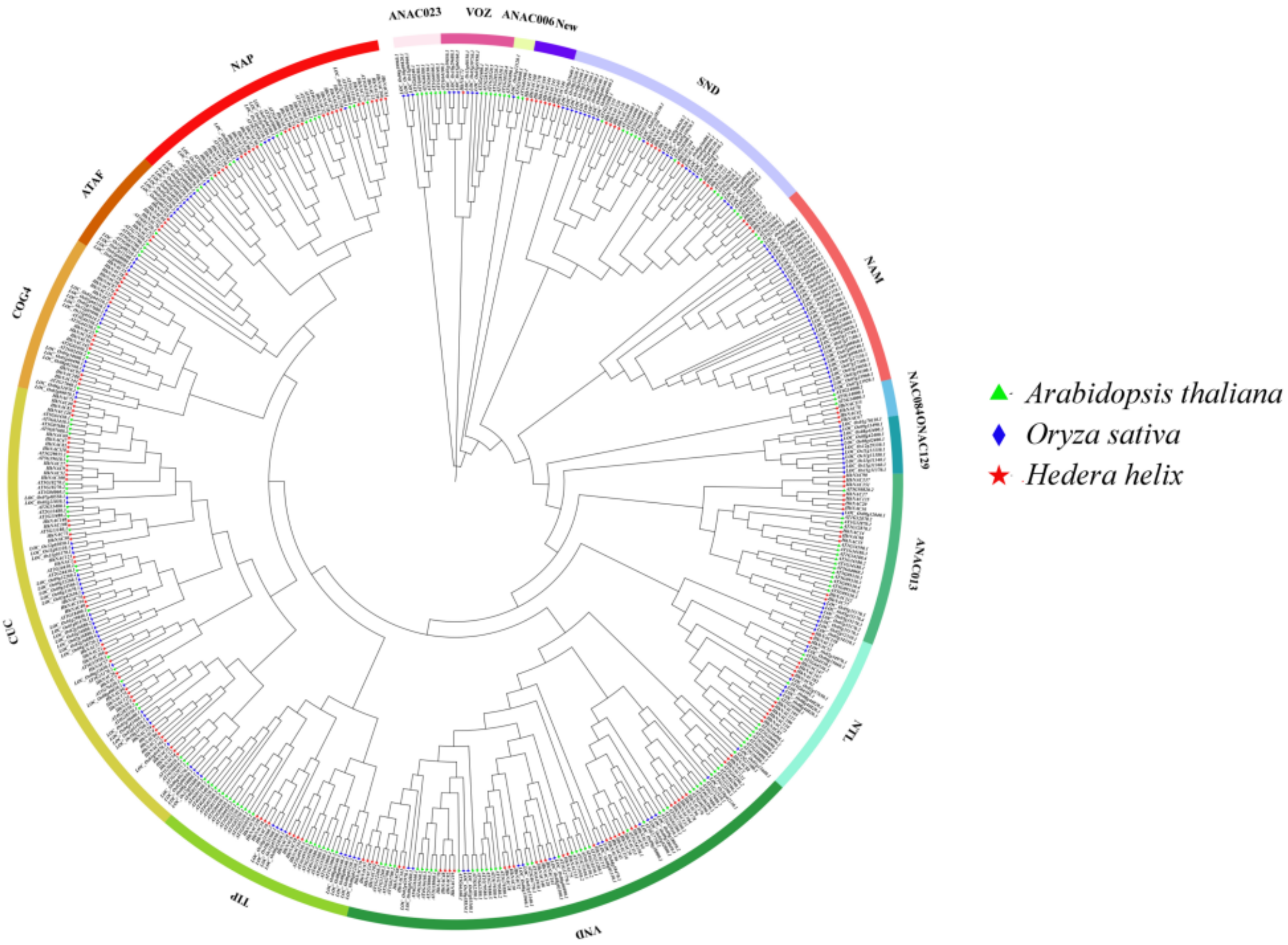

Figure S1 Phylogenetic analysis of NAC members in *H. helix*, *A. thaliana* and *O. sativa*.

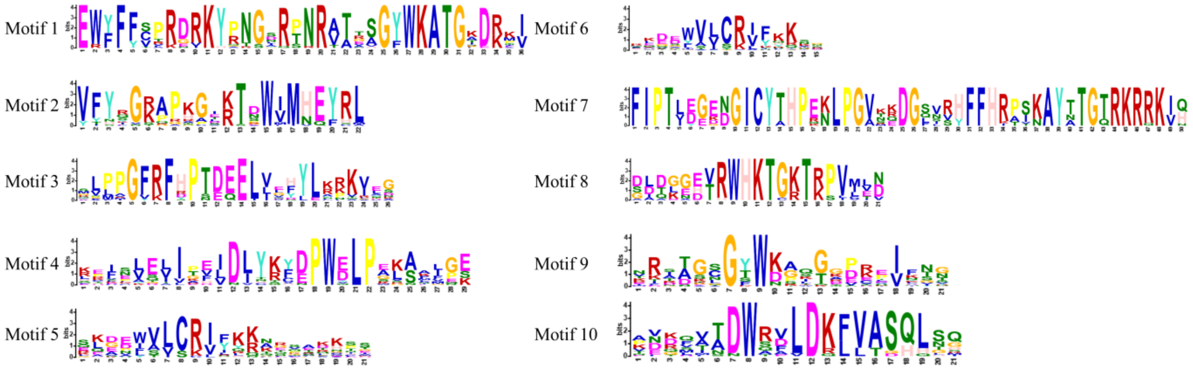

Figure S2 The information of motif 1-10 using MEME server.

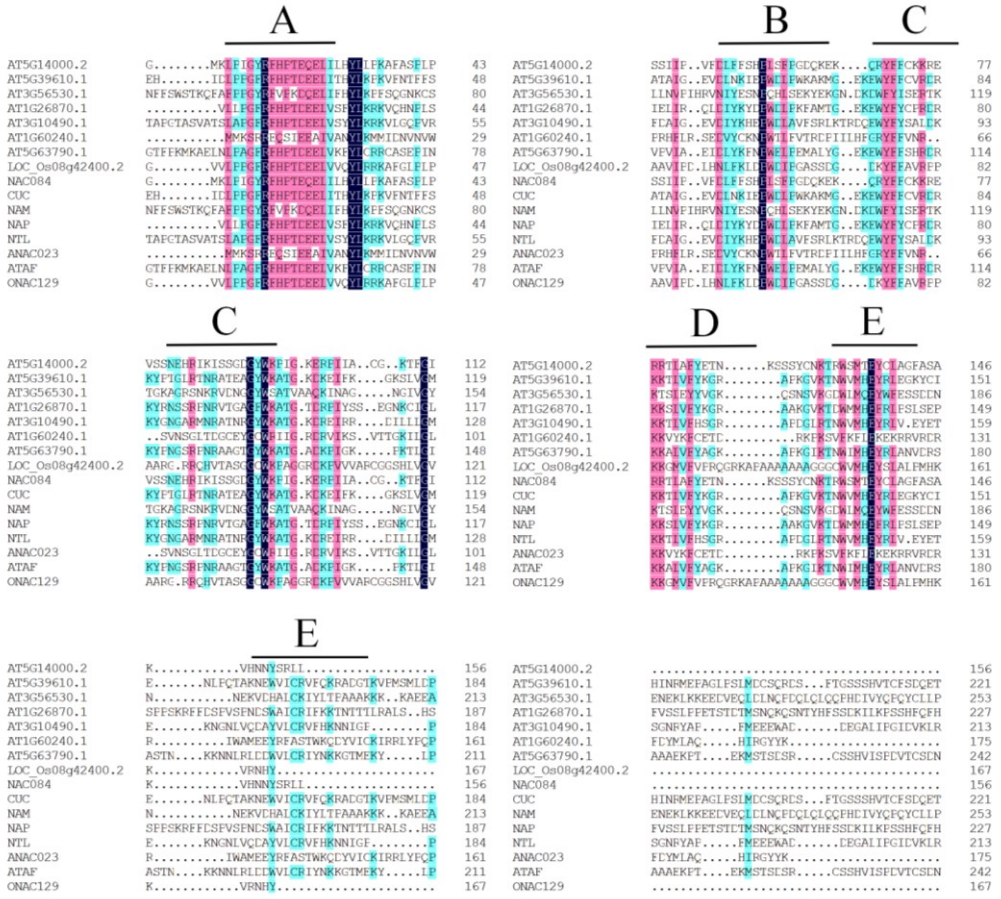

Figure S3 Sequence alignment of NAC domains.
